# *tert*-Butyl Carbonate as a Nucleophile in Epoxide Ring Opening: A Cascade Synthesis of 5‑(Hydroxymethyl)oxazolidin-2-one

**DOI:** 10.1021/acs.joc.5c00537

**Published:** 2025-06-04

**Authors:** Kelly Lee, Xsingyi Lee, Ci-Yi Zhou, Yung-Lan Chu, Cheng-Kun Lin

**Affiliations:** Department of Chemistry, 34916 National Chung Hsing University, 145 Xingda Rd., South Dist., Taichung City 402, Taiwan

## Abstract

We report an efficient and scalable synthesis of 5-(hydroxymethyl)­oxazolidin-2-ones using *tert*-butyl carbonate as a nucleophile in a Boc_2_O-mediated epoxide ring-opening cascade. Optimization identified Et_3_N as key to high yields, with electron-withdrawing aryl groups further enhancing efficiency (up to 91%). Mechanistic studies support the nucleophilic role of *tert*-butyl carbonate. This versatile method enables access to pharmaceutically relevant compounds, including intermediates for Linezolid, highlighting its value in medicinal chemistry.

## Graphical abstract

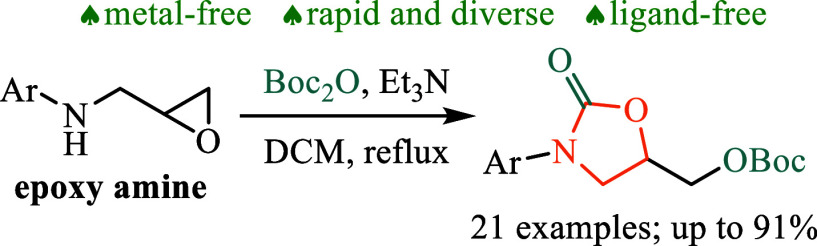

Molecules containing an oxazolidinone ring substituted with a hydroxymethyl group at the C5 position represent a significant class of compounds in medicinal chemistry, recognized for their diverse pharmacological properties and extensive applications, particularly as antidepressants and antibacterial agents.^1^ Notable examples (Figure 1) include Toloxatone (Humoryl),^2^ an antidepressant that functions via monoamine oxidase inhibition, with its effectiveness enhanced by the functional group at this position. Another prominent molecule is Delpazolid,^3^ closely related to Linezolid but exhibiting potential improvements in pharmacokinetics or reduced side effects due to structural modifications at this site.

**Figure 1:**
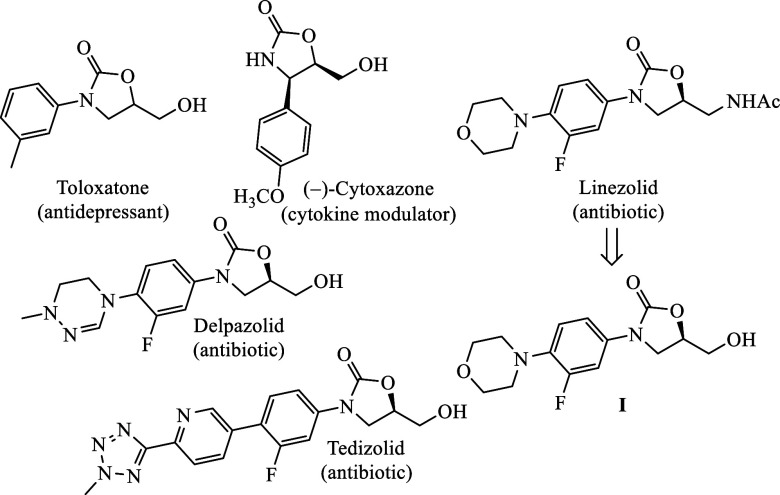
Examples of molecules containing an oxazolidinone ring with a C5-hydroxymethyl substituent.

Tedizolid,^4^ a next-generation antibacterial agent, builds upon the oxazolidinone structure of Linezolid (Zyvox),^5^ offering enhanced binding affinity to bacterial ribosomal targets and an improved safety profile. Linezolid itself is widely utilized for treating Gram-positive bacterial infections, with the substituent on its oxazolidinone ring crucial for enhancing its antibacterial mechanism. Linezolid can occasionally be synthesized from compound **I** as a key intermediate,^6^ highlighting an essential synthetic route for its production. Another notable compound, Cytoxazone,^7^ isolated from Streptomyces, contains a 4,5-disubstituted oxazolidin-2-one ring and functions as a cytokine modulator by influencing Th2 cell signaling, which is integral to cell growth and differentiation.

Compounds bearing a C5-hydroxymethyl-substituted oxazolidinone ring have attracted sustained research interest, particularly aimed at developing more efficient synthetic methodologies due to their critical role in medicinal chemistry. For instance, in 2014,^8^ Liu reported a palladium-catalyzed intramolecular aminohydroxylation of alkenes using hydrogen peroxide as an oxidant, resulting in alkyl alcohols with good diastereoselectivity (Figure 2a). This reaction likely proceeds via an S_N_2-type nucleophilic attack of water on a high-valent palladium intermediate, enabling the synthesis of 2-amino-1,3-diols and 3-ol amino acids. In 2018,^9^ Kim described the divergent coupling of *N*-aryl epoxy amines and CO_2_ to produce either cyclic carbonates or oxazolidinones (Figure 2b). These reactions were catalyzed by an Al­(III) complex, with product selectivity determined by the cocatalyst choice. When combined with 4-(dimethylamino)­pyridine (DMAP), oxazolidinones were selectively formed. Subsequently, Kleij and Pericàs developed a similar catalytic system utilizing TBD@PS as a highly recyclable catalyst for converting epoxy amines into 2-oxazolidone scaffolds using CO_2_ as a C1 source in a continuous-flow setup (Figure 2c).^10^ In 2019,^11^ Toda and Suga introduced a phosphonium salt-catalyzed reaction between glycidols and isocyanates for oxazolidinone synthesis (Figure 2d). Enhanced by electron-rich aryl substituents, tetraarylphosphonium salts facilitated high yields of 4-hydroxymethyl-substituted oxazolidinones, including enantio-enriched forms. They also reported a method for synthesizing five-membered cyclic carbamates using atmospheric CO_2_ as the carbon source, facilitated by a base promoter (Figure 2e).^12^ Recently, Feng and Pan used very close conditions (CO_2_ and Cs_2_CO_3_) in the presence of phosphine but starting from primary amines to access oxazolidinones.^13^ Although these methods significantly advanced oxazolidinone synthesis, they often necessitate elevated temperatures, pressures, specific catalysts, and controlled reaction conditions, limiting their scalability and practicality for large-scale industrial applications.

**Figure 2:**
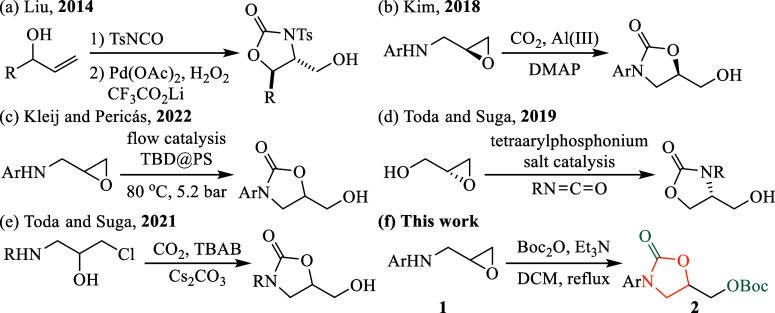
Recent advances in the synthesis of oxazolidinones bearing a C5-hydroxymethyl group and related compounds.

Herein, we report a straightforward and efficient synthetic approach for oxazolidinone preparation, building upon our previous work on *N*-aryl-5-substituted-2-oxazolidinones (Figure 2f). This method employs Boc_2_O to simultaneously introduce the carbonyl functionality of the oxazolidinone and the hydroxymethyl group (post-Boc removal), underscoring its dual role. Boc_2_O,^14^ widely utilized for amine protection in peptide, pharmaceutical, and cosmetic syntheses, provides several advantages, including low toxicity, affordability, and ease of removal, making it particularly suitable for scalable synthetic processes.

Previously, we attempted the synthesis of oxazolidinones via nucleophilic epoxide ring opening and intramolecular acyl substitution of epoxy carbamates.^15^ However, the preparation of epoxy carbamate **3** from epoxy amine **1a** yielded unsatisfactory results (Scheme 1a). Specifically, when the reaction was conducted in THF with DMAP under reflux conditions, oxazolidinone **2a** was isolated in only 17% yield, bearing a C5-*tert*-butoxycarboxymethyl group, clearly indicating suboptimal conditions.

**Scheme 1:**
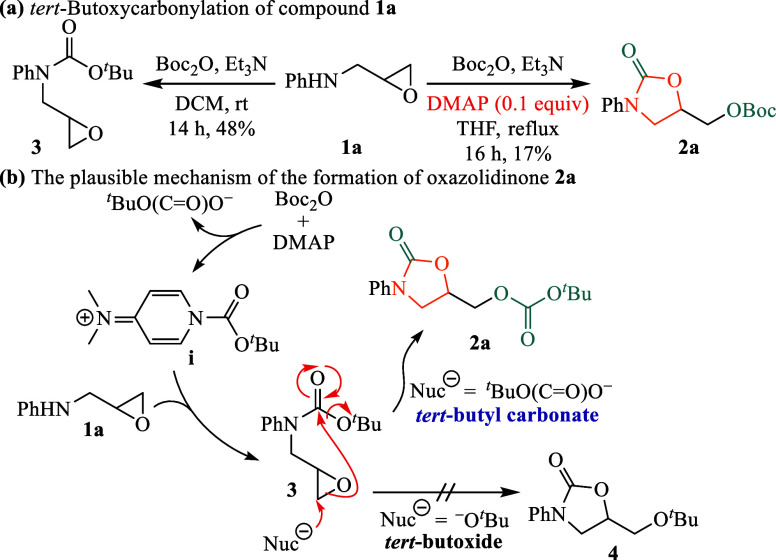
Preparation of Epoxy Carbamate **3**

The structure of compound **2a** was confirmed by ^13^C NMR analysis, which displayed a quaternary carbon signal at 154.1 ppm, corresponding to the Boc group. This observation implies that compound **3** initially forms during the reaction (Scheme 1b), subsequently undergoing epoxide ring opening by *tert*-butyl carbonate (^*t*^BuO­(C=O)­O^–^) and intramolecular cyclization to yield the oxazolidinone product featuring a C5-*tert*-butoxycarboxymethyl substituent. To our knowledge, this is the first report demonstrating *tert*-butyl carbonate functioning as a nucleophile in an epoxide opening reaction.

In contrast, when *tert*-butoxide (^*t*^BuO^–^) was used as the nucleophile, the reaction yielded ether **4**. The ^13^C NMR spectrum exhibited no carbonyl carbon signal from the Boc group, indirectly supporting the proposed reaction mechanism. This finding underscores the significant impact of reaction conditions on product formation, indicating the need for further optimization to enhance both yield and efficiency of the desired products.

This study systematically examines the Boc_2_O-mediated synthesis of oxazolidinones, emphasizing the effects of solvent selection, reagent equivalents, and additives to optimize yield while minimizing side product formation, as summarized in Table 1.

**Table 1:**
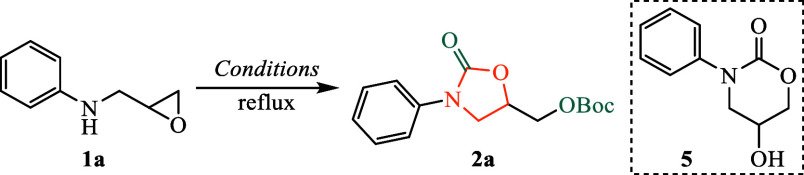
Optimization of Reaction Conditions

entry	conditions					product (% yield)^a^
	equiv of Boc_2_O	equiv of Et_3_N	additive (equiv)	solvent	time (h)	
1	1.5	1.1	DMAP (0.1)	THF	16	**2a** (17)
2	1.5	1.1		THF	16	**2a** (messy)
3	1.5	1.1	DMAP (0.1)	DCM	16	**2a** (29)
4	1.5	1.1		DCM	16	**2a** (74)
5	2	2		DCM	16	**2a** (78)
6	2			DCM	16	**3** (85)
7	2		NH_4_Cl (2)	DCM	14	**3** (81)
8	2		TBAB (2)	DCM	14	**2a** (45)
9	1.5	2		DCM	14	**2a** (77)
10	1.5	2	TBAB (2)	DCM	14	**2a** (78)
11	1.1	3		DCM	14	**2a** (77)
12	1.1		TBAB (3)	DCM	14	**5** (72)
13	1.3	3		DCM	14	**2a** (78)
14	1.3	3		MTBE	14	**2a** (69)

a Isolated yield.

Solvent selection was found to critically influence reaction efficiency. THF (entries 1–2) resulted in poor yields (17%) or complicated reaction mixtures, confirming its unsuitability for this transformation. Conversely, dichloromethane (DCM) consistently delivered superior yields (entries 3–5, 8–11, 13), with entry 5 (2.0 equiv Boc_2_O, 2.0 equiv Et_3_N in DCM) achieving a notable 78% yield. Further investigations explored variations in Boc_2_O and Et_3_N stoichiometry, showing that increasing Boc_2_O from 1.5 to 2.0 equiv enhanced the yield from 74% to 78% (entry 5). Interestingly, reducing Boc_2_O to 1.1 equiv while increasing Et_3_N to 3.0 equiv (entry 11) maintained the yield at 77%, demonstrating that lower Boc_2_O loading can be compensated by increased Et_3_N, providing a cost-effective alternative.

Comparative analysis of entries 9 (1.5 equiv Boc_2_O, 2 equiv Et_3_N, DCM, 77%), 11 (1.1 equiv Boc_2_O, 3 equiv Et_3_N, DCM, 77%), and 13 (1.3 equiv Boc_2_O, 3 equiv Et_3_N, DCM, 78%) highlights the impact of reagent ratio optimization on reaction efficiency. Entry 9 indicates that increased Et_3_N with moderate Boc_2_O loading yields satisfactory results. Entry 11 demonstrates that significantly lowering Boc_2_O to 1.1 equiv while maximizing Et_3_N maintains comparable yields (77%), emphasizing the crucial role of the base. Entry 13, featuring a modest adjustment in Boc_2_O equivalents, shows only marginal improvement (78%), suggesting that additional Boc_2_O provides minimal incremental benefits. Collectively, these results demonstrate that higher Et_3_N loadings effectively offset reduced Boc_2_O amounts, representing a more sustainable approach without yield compromise.

The influence of quaternary ammonium salts (NH_4_Cl and TBAB) on reaction selectivity was also assessed. NH_4_Cl (entry 7, 2 equiv) exclusively yielded intermediate **3** (protection product, 81%), suggesting that ammonium salts stabilize *tert*-butyl carbonate, preventing further conversion. TBAB (entry 8, 2 equiv) increased yield to 45%, and entry 10 (1.5 equiv Boc_2_O, 2.0 equiv Et_3_N, 2.0 equiv TBAB, DCM) further improved the yield to 78%, confirming its catalytic role in enhancing conversion. However, excess TBAB (entry 12, 1.1 equiv Boc_2_O, no Et_3_N, 3 equiv TBAB, DCM) resulted in formation of a six-membered cyclic carbamate (compound **5**, 72%), indicating a shift in reaction mechanism away from oxazolidinone formation. Furthermore, the absence of Et_3_N (entry 6, 2.0 equiv Boc_2_O, DCM) produced intermediate **3** exclusively (85% yield), emphasizing Et_3_N’s critical role in facilitating cyclization to oxazolidinone **2a**. Thus, balancing reagent equivalents and additive choice is essential for optimizing reaction selectivity and yield. Ultimately, entry 11 (1.1 equiv Boc_2_O, 3.0 equiv Et_3_N, DCM) emerged as the most efficient reaction conditions.

With the optimized reaction conditions established, we found that *N*-aryl-5-substituted-2-oxazolidinones containing a *tert*-butoxycarboxymethyl side chain could be efficiently synthesized by directly treating the starting epoxy amine **1** with Boc_2_O. To further understand how various aryl substituents influence reaction outcomes, we explored the substrate scope comprehensively, as summarized in Table 2. This investigation provided valuable insights into how electronic and steric effects from electron-withdrawing groups (EWGs) and electron-donating groups (EDGs) at different positions on the aryl ring significantly impacted the reaction yields.

**Table 2:**
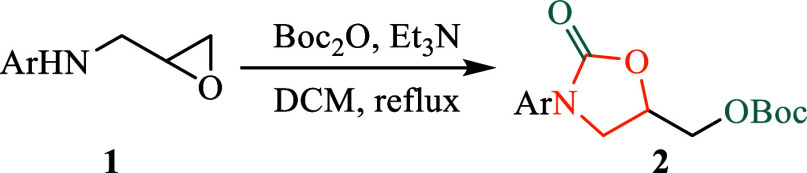

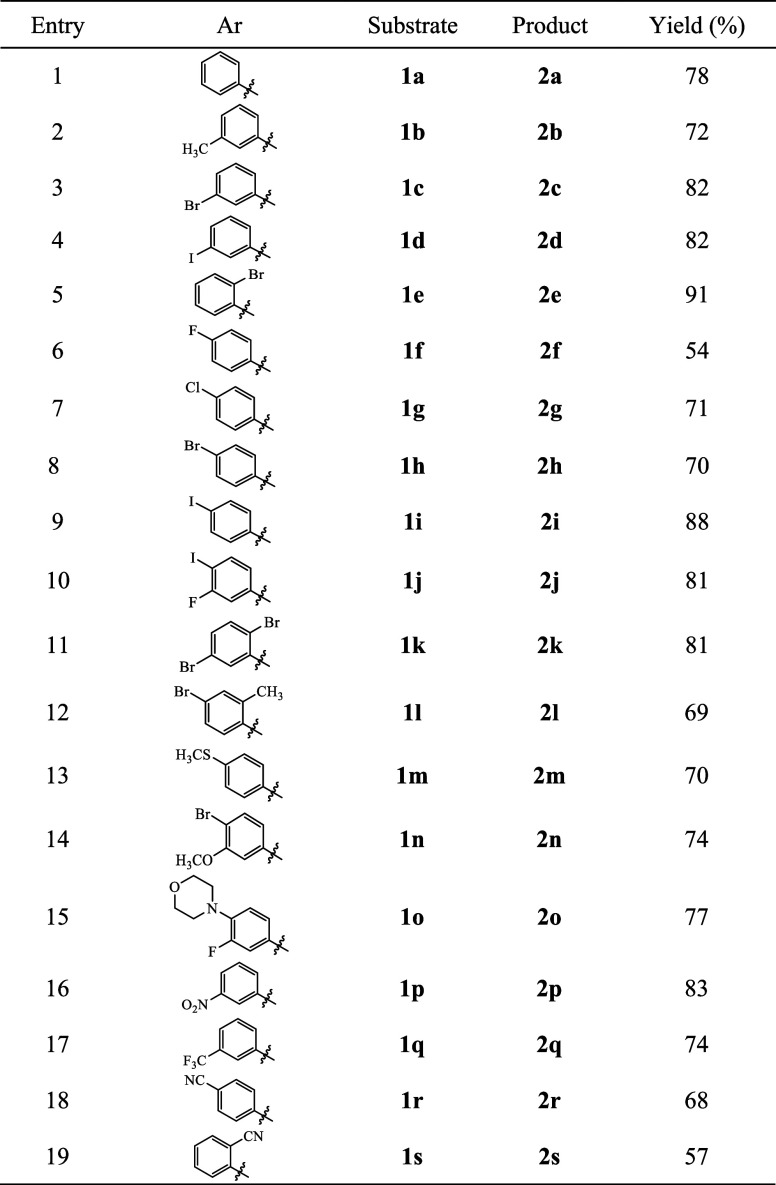
Substrate Scope^a^

a The reaction was conducted with 0.5 mmol of the starting material in DCM as the solvent, using 1.1 equiv of Boc_2_O and 3 equiv of Et_3_N.

*Meta*-substituted groups exhibited distinct trends: a *meta*-methyl electron-donating substituent (entry 2, **1b**) yielded 72%, whereas *meta*-electron-withdrawing substituents such as bromo and iodo (entries 3–4, **1c** and **1d**) achieved higher yields (82%). *Ortho*-substituted groups showed the most pronounced effects, with the *ortho*-bromo substituent (entry 5, **1e**) giving the highest yield of 91%, likely due to increased electrophilicity enhancing nucleophilic attack. *Para*-substituted groups displayed varied outcomes; *para*-fluoro substituents (entry 6, **1f**) resulted in the lowest yield (54%), whereas larger *para*-halogen substituents like iodo (entry 9, **1i**) achieved significantly higher yields (88%), suggesting that steric bulk and polarizability may positively affect reaction efficiency.

We also investigated the synergistic effects of multiple substituents. A combination of *para*-iodo and *meta*-fluoro substituents (entry 10, **1j**) yielded 81%, while combined *ortho*- and *meta*-bromo substituents (entry 11, **1k**) also yielded 81%, indicating a stabilizing electronic effect from dual EWGs. Conversely, introducing an *ortho*-methyl EDG alongside a *para*-bromo EWG (entry 12, **1l**) reduced the yield to 69%, highlighting potential steric hindrance from *ortho*-EDGs. The effect of EDGs was further assessed: a *para*-methylthio substituent (entry 13, **1m**) provided a moderate yield of 70%, indicating minimal interference from sulfur-containing EDGs. Additionally, a combination of *para*-bromo and *meta*-methoxy substituents (entry 14, **1n**) modestly improved the yield to 74%, suggesting mild EDGs at the *meta* position do not significantly hinder reaction efficiency. Interestingly, the combination of a strong *para*-amino EDG with a *meta*-fluoro EWG (entry 15, **1o**) delivered a relatively high yield of 77%, indicating a beneficial balance between EDG and EWG effects. A strongly electron-withdrawing *meta*-nitro group (entry 16, **1p**) resulted in a high yield of 83%. Similarly, the *meta*-trifluoromethyl group (entry 17) gave a solid yield of 74%, while *para*-cyano (entry 18) gave a moderate 68%, and *ortho*-cyano (entry 19) reduced the yield to 57%.

The results depicted in Scheme 2 provide compelling evidence supporting the role of *tert*-butyl carbonate as an effective nucleophile in the reaction. Under various reaction conditions, the yield of cyclic carbonate **7** showed considerable variation, highlighting the sensitivity of the reaction to external parameters.

**Scheme 2:**
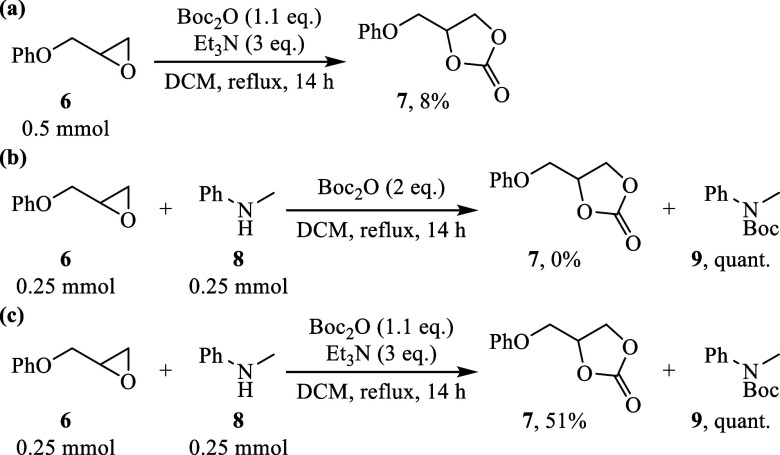
Reactions Designed to Demonstrate that *tert*-Butyl Carbonate Functions as a Nucleophile

Scheme 2a illustrates that only a minor amount of product **7** was formed, indicating suboptimal conditions for nucleophilic activation. In contrast, Scheme 2b shows that, in the presence of secondary amine **8**, the reaction predominantly yielded the Boc-protected product **9**, with no detectable formation of **7**, suggesting that the amine presence alters the reaction pathway. However, Scheme 2c demonstrates that the addition of Et_3_N significantly enhances the yield of **7**, confirming that Et_3_N is critical for promoting the nucleophilic activity of *tert*-butyl carbonate. Collectively, these results clearly validate *tert*-butyl carbonate as a viable nucleophile capable of directly participating in the transformation leading to cyclic carbonate formation.

The proposed mechanism for cyclic carbonate **7** formation, shown in Scheme 3a, involves protonation of Et_3_N to form an ammonium ion, stabilizing *tert*-butyl carbonate and preventing its decomposition into CO_2_ and *tert*-butoxide. This stabilization enables the effective nucleophilic attack of *tert*-butyl carbonate on epoxide **6**, forming an intermediate that subsequently undergoes intramolecular acyl substitution to yield cyclic carbonate **7**. Thus, ammonium ion stabilization of *tert*-butyl carbonate is essential for the reaction’s efficiency and success.

**Scheme 3:**
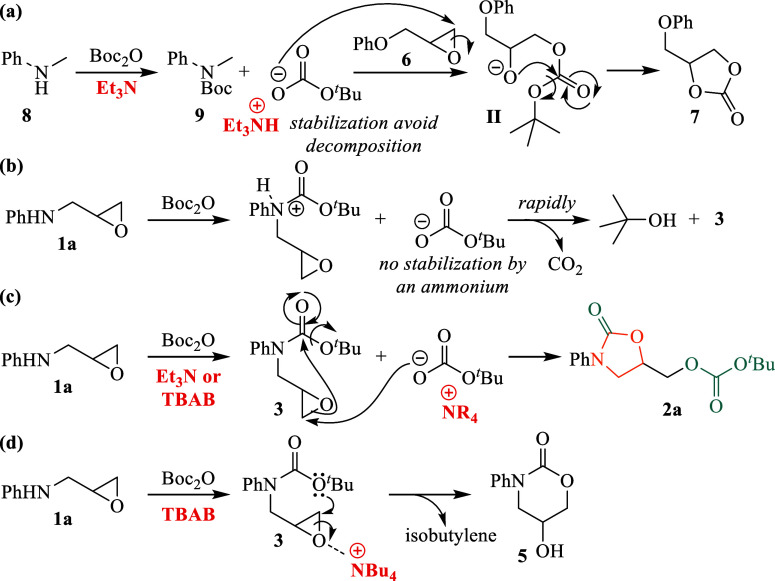
Proposed Mechanism for the Formation of Cyclic Carbonate **7**, Oxazolidinone **2a**, and Cyclic Carbamate **5**

Moreover, the reaction pathways leading to *tert*-butyl carbamate **3** and oxazolidinone **2a** were also examined. Without ammonium ion stabilization, *tert*-butyl carbonate rapidly decomposes to *tert*-butoxide, which then reacts to exclusively yield carbamate **3** (Scheme 3b; entry 6, Table 1). Conversely, in the presence of Et_3_N or an external ammonium source such as TBAB, *tert*-butyl carbonate remains stable, enabling intermolecular nucleophilic epoxide ring-opening followed by intramolecular acyl substitution, resulting in the formation of oxazolidinone **2a** (Scheme 3c). When excess TBAB is present, the reaction favors intramolecular nucleophilic epoxide ring-opening due to epoxide activation, producing cyclic carbamate **5** instead (Scheme 3d; entry 12, Table 1).

Scheme 4 highlights key synthetic applications that demonstrate the efficiency, scalability, and versatility of the developed cascade synthesis method. First, Scheme 4a illustrates the successful gram-scale synthesis of Toloxatone, emphasizing that the method is not only highly efficient but also readily scalable, reinforcing its practical applicability in pharmaceutical production.

**Scheme 4:**
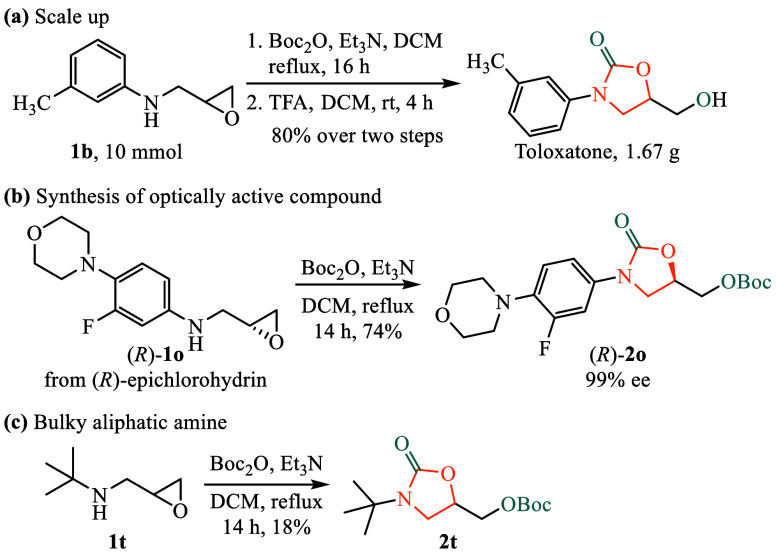
Synthetic Applications

Second, Scheme 4b showcases the method’s capability to produce optically active intermediates, specifically compound (*R*)-**2o**. Following the removal of the Boc protecting group, this intermediate plays a crucial role in the synthesis of Linezolid, a widely used antibacterial agent. Notably, the chirality of the starting material is preserved, ensuring the retention of optical purity, which is essential for biological activity. The synthesis of (*R*)-**2o** further underscores the versatility of this cascade approach, particularly its significance in medicinal chemistry for the production of chiral pharmaceutical intermediates.

In Scheme 4c, the use of a bulky *tert*-butylamine resulted in the formation of the desired oxazolidinone product, albeit in 18% yield. This lower efficiency is likely due to steric hindrance, which may interfere with the nucleophilic attack on the epoxide or hinder effective cyclization. Nonetheless, the successful formation of the product highlights the tolerance of the method to nonaryl amines, suggesting its potential applicability to broader structural classes.

In conclusion, we have successfully demonstrated *tert*-butyl carbonate as a nucleophile in epoxide ring opening, providing a practical cascade synthetic route for efficiently synthesizing *N*-aryl-5-substituted-2-oxazolidinones, compounds critical in medicinal chemistry. Through careful optimization of reaction conditions, we achieved excellent yields, particularly with electron-withdrawing substituents on the aryl rings. Detailed mechanistic studies confirm the novel use of *tert*-butyl carbonate, significantly expanding its synthetic utility in epoxide ring-opening reactions. The method’s robustness and versatility are validated by gram-scale production and successful synthesis of optically active intermediates essential for pharmaceutical agents like Toloxatone and Linezolid. Overall, this cascade synthesis offers an efficient, scalable, and versatile synthetic platform suitable for broad adoption in both research and industrial pharmaceutical production.

## Supplementary Material



## Data Availability

The data underlying this study are available in the published article and its Supporting Information.

## Notes

Experimental procedures, ^1^H, ^13^C NMR spectra, and characterization data for the synthesized compounds (PDF)
