# Supplementary material for: tert-Butyl Carbonate as a Nucleophile in Epoxide Ring Opening: A Cascade Synthesis of 5‑(Hydroxymethyl)oxazolidin-2-one
Source: J Org Chem. 2025 Jun 4;90(23):7976–81. doi: 10.1021/acs.joc.5c00537 (PMC12172036; doi:10.1021/acs.joc.5c00537)
Supplement: Supplementary file 1 [file jo5c00537_si_001.pdf]

# Supporting Information

## *tert*-Butyl Carbonate as a Nucleophile in Epoxide Ring Opening: A Cascade Synthesis of 5-(Hydroxymethyl)oxazolidin-2-one

Kelly Lee, Xsingyi Lee, Yung-Lan Chu, Ci-Yi Zhou, and Cheng-Kun Lin*

*Department of Chemistry, National Chung Hsing University, Taichung 402, Taiwan*

*E-mail: cklin93@nchu.edu.tw*

| | Table of Content | Page |
|---|---|---|
| A. | General information | S2 |
| B. | Experimental section | S2 |
| | Typical Procedure A for the synthesis of chlorohydrin **S1** | S2 |
| | Typical Procedure B for the synthesis of epoxy amine **1** | S10 |
| | Table S1. Optimization experiment | S17 |
| | Typical Procedure C for the synthesis of compound **2** | S19 |
| | Reactions designed to demonstrate that *tert*-butyl carbonate functions as a nucleophile | S27 |
| | Synthetic Applications | S27 |
| | Table S2. Epoxidation and Subsequent Oxazolidinone Formation for Selected Aliphatic Substrates | S29 |
| C. | NMR Spectra for the synthesized compounds | S31 |
| | References and Notes | S101 |

## A. General Information

Unless otherwise specified, all reagents and solvents were commercially sourced and used without additional purification. The $^{1}$H and $^{13}$C, and $^{19}$F NMR spectra were recorded on a JEOL-400 MHz spectrometer, with TMS (δ = 0.00) in chloroform-*d* or acetone-$d_6$ (δ = 2.05) serving as an internal standard for $^{1}$H NMR spectra. For $^{13}$C NMR spectra, the central peak of chloroform-*d* (δ = 77.0) or acetone-$d_6$ (δ = 29.9) was employed as the internal standard. For $^{19}$F NMR spectra, chemical shifts were calibrated with $^{1}$H NMR data as the standard through X-nucleus calibration method. JEOL Delta 6.3.0 software was used for processing NMR data. High-resolution mass spectrometry (HRMS) analyses were conducted on a Thermo Scientific Orbitrap LTQ XL mass spectrometer or on a Bruker Daltonics BioTOF III spectrometer (ESI-MS). Optical rotations were measured in $CH_2Cl_2$ solution using a cuvette of 1 dm length on a Rudolph Autopol IV automatic polarimeter at λ = 589 nm (Na). Melting points were measured on an Electrothermal 1101D Mel-Temp digital melting point apparatus with a capillary melting point tube. Thin-layer chromatography (TLC) plates were visualized by exposure to ultraviolet light at 254 nm and/or by immersion in a staining solution (phosphomolybdic acid, potassium permanganate, ninhydrin, or *p*-anisaldehyde), followed by heating on a hot plate. For flash chromatography, silica gel 60 with a mesh size of 70-230 ASTM was utilized. These comprehensive analytical techniques ensure the reliability and accuracy of the experimental results, contributing to the robustness of the reported findings in the study. The epoxy amine **1** was prepared according to the following methods.

## B. Experimental section

**The epoxy amines 1 listed in Table 2 were synthesized using the following procedures.**

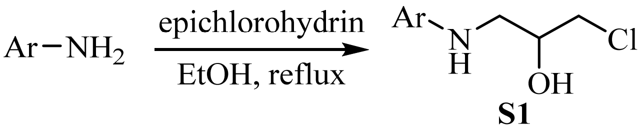


**S1**

**Typical Procedure A** for the synthesis of chlorohydrin **S1**.

To a solution of aniline (5 mmol) in EtOH (10 mL) was added epichlorohydrin (392 μL, 5 mmol, 1 equiv) and was stirred at reflux for 12 hours. Upon completion of the reaction, as determined by TLC analysis, the mixture was concentrated under reduced pressure and purified by column chromatography to afford compound **S1**.

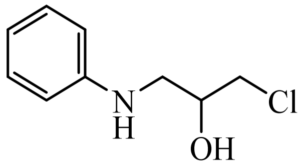


**S1a**

**1-Chloro-3-(phenylamino)propan-2-ol (S1a).**[S1] The preparation was carried out according on **Typical**

**Procedure A**. Starting from aniline (457 μL, 5 mmol), after purified by column chromatography [silica gel, *n*-hexane/ethyl acetate = 4/1 (v/v)], the title compound was obtained as a colorless liquid (624 mg, 67%). R*f* = 0.5 [*n*-hexane/ethyl acetate = 2/1 (v/v)]; $^1$H NMR (400 MHz, $CDCl_3$) δ 7.21–7.17 (m, 2H), 6.75 (t, *J* = 7.3 Hz, 1H), 6.66 (dd, *J* = 8.6, 1.0 Hz, 2H), 4.10–4.05 (m, 1H), 3.71–3.61 (m, 2H), 3.38 (dd, *J* = 13.3, 4.4 Hz, 1H), 3.23 (dd, *J* = 13.3, 7.2 Hz, 1H); $^{13}$C{$^1$H} NMR (101 MHz, $CDCl_3$) δ 147.7, 129.4, 118.3, 113.3, 69.8, 47.7, 47.1; HRMS (EI): calculated for $C_9H_{12}ClNO$ ($M^+$), 185.0607, found 185.0600.

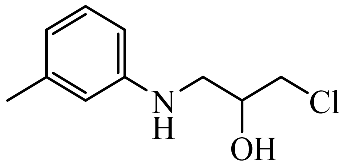


**S1b**

**1-Chloro-3-(*m*-tolylamino)propan-2-ol (S1b).**[S1] The preparation was carried out according on **Typical Procedure A**. Starting from *m*-toluidine (547 μL, 5 mmol), the round-bottom flask was wrapped in aluminum foil to prevent the *m*-toluidine from decomposing due to light exposure, after purified by column chromatography [silica gel, *n*-hexane/ethyl acetate = 4/1 (v/v)], the title compound was obtained as an orange liquid (520 mg, 52%). R*f* = 0.4 [*n*-hexane/ethyl acetate = 2/1 (v/v)]; $^1$H NMR (400 MHz, $CDCl_3$) δ 7.10–7.06 (m, 1H), 6.59–6.57 (m, 1H), 6.48 (d, *J* = 7.8 Hz, 2H), 4.10–4.05 (m, 1H), 3.71–3.62 (m, 2H), 3.37 (dd, *J* = 13.3, 4.4 Hz, 1H), 3.23 (dd, *J* = 13.3, 7.0 Hz, 1H), 2.28 (s, 3H); $^{13}$C{$^1$H} NMR (101 MHz, $CDCl_3$) δ 147.7, 139.2, 129.2, 119.2, 114.1, 110.4, 69.8, 47.7, 47.1, 21.6; HRMS (EI): calculated for $C_{10}H_{14}ClNO$ ($M^+$), 199.0764, found 199.0760.

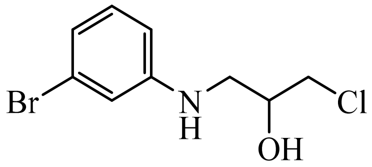


**S1c**

**1-((3-Bromophenyl)amino)-3-chloropropan-2-ol (S1c).**[S2] The preparation was carried out according on **Typical Procedure A**. Starting from 3-bromoaniline (544 μL, 5 mmol), after purified by column chromatography [silica gel, *n*-hexane/ethyl acetate = 4/1 (v/v)], the title compound was obtained as an orange liquid (954 mg, 72%). R*f* = 0.5 [*n*-hexane/ethyl acetate = 2/1 (v/v)]; $^1$H NMR (400 MHz, $CDCl_3$) δ 7.03 (t, *J* = 8.0 Hz, 1H), 6.87 (dq, *J* = 7.8, 0.9 Hz, 1H), 6.81 (t, *J* = 2.1 Hz, 1H), 6.58 (ddd, *J* = 8.3, 2.3, 0.8 Hz, 1H), 4.11–4.06 (m, 1H), 3.71–3.61 (m, 2H), 3.36 (dd, *J* = 13.2, 4.4 Hz, 1H), 3.22 (dd, *J* = 13.2, 7.3 Hz, 1H); $^{13}$C{$^1$H} NMR (101 MHz, $CDCl_3$) δ 148.9, 130.6, 123.3, 121.0, 115.8, 112.1, 69.7, 47.6, 46.8; HRMS (EI): calculated for $C_9H_{11}BrClNO$ ($M^+$), 262.9713, found 262.9718.

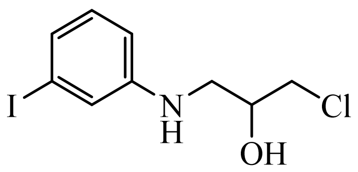

**S1d**

**1-Chloro-3-((3-iodophenyl)amino)propan-2-ol (S1d).** The preparation was carried out according on **Typical Procedure A**. Starting from 3-iodoaniline (601 μL, 5 mmol), after purified by column chromatography [silica gel, *n*-hexane/ethyl acetate = 4/1 (v/v)], the title compound was obtained as a yellow liquid (1.107 g, 71%). $R_f$ = 0.5 [*n*-hexane/ethyl acetate = 2/1 (v/v)]; $^1$H NMR (400 MHz, $CDCl_3$) δ 7.06 (ddd, *J* = 7.8, 1.4, 1.0 Hz, 1H), 6.99–6.98 (m, 1H), 6.88 (t, *J* = 7.9 Hz, 1H), 6.59 (ddd, *J* = 8.2, 2.3, 0.8 Hz, 1H), 4.07–4.02 (m, 1H), 3.68–3.58 (m, 2H), 3.31 (dd, *J* = 13.3, 4.3 Hz, 1H), 3.17 (dd, *J* = 13.1, 7.3 Hz, 1H); $^{13}$C{$^1$H} NMR (101 MHz, $CDCl_3$) δ 148.8, 130.7, 127.0, 121.8, 112.6, 95.2, 69.6, 47.5, 46.7; HRMS (EI): calculated for $C_9H_{11}ClINO$ ($M^+$), 310.9574, found 310.9570.

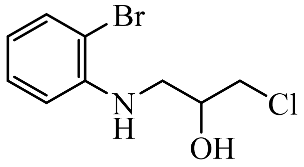

**S1e**

**1-((2-Bromophenyl)amino)-3-chloropropan-2-ol (S1e).**[S3] The preparation was carried out according on **Typical Procedure A**. Starting from 2-bromoaniline (566 μL, 5 mmol), after purified by column chromatography [silica gel, *n*-hexane/ethyl acetate = 8/1 (v/v)], the title compound was obtained as a yellow liquid (768 mg, 58%). $R_f$ = 0.6 [*n*-hexane/ethyl acetate = 3/1 (v/v)]; $^1$H NMR (400 MHz, $CDCl_3$) δ 7.44 (dd, *J* = 7.9, 1.5 Hz, 1H), 7.21–7.17 (m, 1H), 6.71 (dd, *J* = 8.2, 1.5 Hz, 1H), 6.64–6.60 (m, 1H), 4.15–4.09 (m, 1H), 3.72–3.63 (m, 2H), 3.43 (dd, *J* = 13.3, 4.6 Hz, 1H), 3.30 (dd, *J* = 13.3, 7.0 Hz, 1H); $^{13}$C{$^1$H} NMR (101 MHz, $CDCl_3$) δ 144.4, 132.6, 128.6, 118.8, 111.8, 110.4, 69.6, 47.5, 47.0; HRMS (EI): calculated for $C_9H_{11}BrClNO$ ($M^+$), 262.9713, found 262.9705.

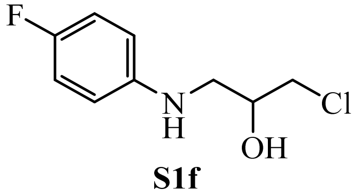

**S1f**

**1-Chloro-3-((4-fluorophenyl)amino)propan-2-ol (S1f).**[S4] The preparation was carried out according on **Typical Procedure A**. Starting from 4-fluoroaniline (474 μL, 5 mmol), after purified by column chromatography [silica gel, *n*-hexane/ethyl acetate = 6/1 (v/v)], the title compound was obtained as a violet liquid (550 mg, 54%). $R_f$ = 0.4 [*n*-hexane/ethyl acetate = 2/1 (v/v)]; $^1$H NMR (400 MHz, $CDCl_3$) δ 6.93–6.87 (m, 2H), 6.62–6.57 (m, 2H), 4.09–4.03 (m, 1H), 3.70–3.61 (m, 2H), 3.33 (dd, *J* = 13.1, 4.2 Hz, 1H),

3.18 (dd, $J$ = 13.1, 7.2 Hz, 1H), 2.78 (s, 1H); $^{13}C\{^1H\}$ NMR (101 MHz, $CDCl_3$) δ 156.3 (d, $J_{C\text{-}F}$ = 236.0 Hz), 144.1 (d, $J_{C\text{-}F}$ = 1.5 Hz), 115.8 (d, $J_{C\text{-}F}$ = 22.6 Hz), 114.3 (d, $J_{C\text{-}F}$ = 7.7 Hz), 69.8, 47.8, 47.6; $^{19}F$ NMR (376 MHz, $CDCl_3$) δ –125.8; HRMS (EI): calculated for $C_9H_{11}ClFNO$ ($M^+$), 203.0513, found 203.0510.

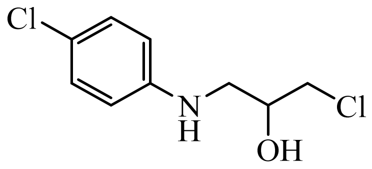

**S1g**

**1-Chloro-3-((4-chlorophenyl)amino)propan-2-ol (S1g).**[S2] The preparation was carried out according on **Typical Procedure A**. Starting from 4-chloroaniline (638 mg, 5 mmol), after purified by column chromatography [silica gel, *n*-hexane/ethyl acetate = 4/1 (v/v)], the title compound was obtained as a pale yellow solid (836 mg, 76%). $R_f$ = 0.4 [*n*-hexane/ethyl acetate = 2/1 (v/v)]; $^1H$ NMR (400 MHz, $CDCl_3$) δ 7.15–7.12 (m, 2H), 6.60–6.56 (m, 2H), 4.10–4.04 (m, 1H), 3.71–3.61 (m, 2H), 3.35 (dd, $J$ = 13.1, 4.4 Hz, 1H), 3.20 (dd, $J$ = 13.1, 7.2 Hz, 1H), 2.42 (s, 1H); $^{13}C\{^1H\}$ NMR (101 MHz, $CDCl_3$) δ 146.3, 129.2, 122.8, 114.3, 69.8, 47.7, 47.1; HRMS (EI): calculated for $C_9H_{11}Cl_2NO$ ($M^+$), 219.0218, found 219.0210; m.p. = 56–57 °C.

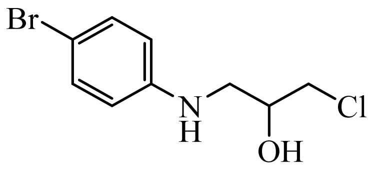

**S1h**

**1-((4-Bromophenyl)amino)-3-chloropropan-2-ol (S1h).**[S1] The preparation was carried out according on **Typical Procedure A**. Starting from 4-bromoaniline (860 mg, 5 mmol), after purified by column chromatography [silica gel, *n*-hexane/ethyl acetate = 4/1 (v/v)], the title compound was obtained as a yellow solid (715 mg, 54%). $R_f$ = 0.4 [*n*-hexane/ethyl acetate = 2/1 (v/v)]; $^1H$ NMR (400 MHz, $CDCl_3$) δ 7.27–7.24 (m, 2H), 6.54–6.50 (m, 2H), 4.07–4.01 (m, 1H), 3.68–3.58 (m, 2H), 3.32 (dd, $J$ = 13.1, 4.3 Hz, 1H), 3.17 (dd, $J$ = 13.2, 7.3 Hz, 1H); $^{13}C\{^1H\}$ NMR (101 MHz, $CDCl_3$) δ 146.7, 132.0, 114.8, 109.7, 69.7, 47.6, 47.0; HRMS (EI): calculated for $C_9H_{11}BrClNO$ ($M^+$), 262.9713, found 262.9715; m.p. = 51–52 °C.

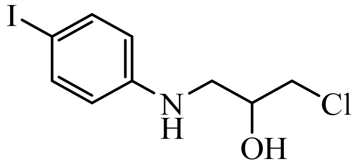

**S1i**

**1-Chloro-3-((4-iodophenyl)amino)propan-2-ol (S1i).**[S2] The preparation was carried out according on **Typical Procedure A**. Starting from 4-iodoaniline (1.10 g, 5 mmol), after purified by column

chromatography [silica gel, *n*-hexane/ethyl acetate = 4/1 (v/v)], the title compound was obtained as a brown solid (964 mg, 62%). $R_f$ = 0.4 [*n*-hexane/ethyl acetate = 2/1 (v/v)]; $^1$H NMR (400 MHz, $CDCl_3$) δ 7.45–7.42 (m, 2H), 6.46–6.42 (m, 2H), 4.09–4.04 (m, 1H), 3.70–3.60 (m, 2H), 3.35 (dd, *J* = 13.3, 4.4 Hz, 1H), 3.20 (dd, *J* = 13.3, 7.2 Hz, 1H), 2.40 (s, 1H); $^{13}C\{^1H\}$ NMR (101 MHz, $CDCl_3$) δ 147.4, 137.9, 115.4, 78.9, 69.7, 47.6, 46.8; HRMS (EI): calculated for $C_9H_{11}ClINO$ ($M^+$), 310.9574, found 310.9570; m.p. = 64–65 °C.

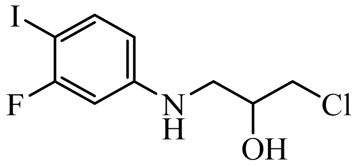


**S1j**

**1-Chloro-3-((3-fluoro-4-iodophenyl)amino)propan-2-ol (S1j).** The preparation was carried out according on **Typical Procedure A**. Starting from 3-fluoro-4-iodoaniline (1.19 g, 5 mmol), after purified by column chromatography [silica gel, *n*-hexane/ethyl acetate = 5/1 (v/v)], the title compound was obtained as a brown solid (967 mg, 59%). $R_f$ = 0.3 [*n*-hexane/ethyl acetate = 4/1 (v/v)]; $^1$H NMR (400 MHz, $CDCl_3$) δ 7.41 (dd, *J* = 8.6, 7.2 Hz, 1H), 6.38 (dd, *J* = 10.5, 2.6 Hz, 1H), 6.24 (dd, *J* = 8.6, 2.6 Hz, 1H), 4.08–4.03 (m, 1H), 3.68–3.58 (m, 2H), 3.37–3.30 (m, 1H), 3.23–3.15 (m, 1H); $^{13}C\{^1H\}$ NMR (101 MHz, $CDCl_3$) δ 162.4 (d, $J_{C\text{-}F}$ = 242.8 Hz), 149.8 (d, $J_{C\text{-}F}$ = 10.1 Hz), 139.0 (d, $J_{C\text{-}F}$ = 3.4 Hz), 111.3 (d, $J_{C\text{-}F}$ = 2.4 Hz), 100.4 (d, $J_{C\text{-}F}$ = 27.9 Hz), 69.6, 64.8 (d, $J_{C\text{-}F}$ = 26.0 Hz), 47.4, 46.7; $^{19}$F NMR (376 MHz, $CDCl_3$) δ –93.7; HRMS (EI): calculated for $C_9H_{10}ClFINO$ ($M^+$), 328.9480, found 328.9487; m.p. = 66–67 °C.

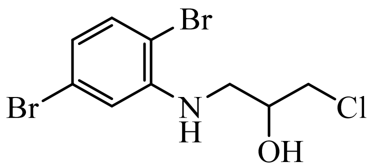


**S1k**

**1-Chloro-3-((2,5-dibromophenyl)amino)propan-2-ol (S1k).** The preparation was carried out according on **Typical Procedure A**. Starting from 2,5-dibromoaniline (1.26 g, 5 mmol), after purified by column chromatography [silica gel, *n*-hexane/ethyl acetate = 6/1 (v/v)], the title compound was obtained as a colorless liquid (560 mg, 33%). $R_f$ = 0.4 [*n*-hexane/ethyl acetate = 4/1 (v/v)]; $^1$H NMR (400 MHz, $CDCl_3$) δ 7.28–7.26 (m, 1H), 6.79 (d, *J* = 2.1 Hz, 1H), 6.72 (dd, *J* = 8.3, 2.2 Hz, 1H), 4.71 (s, 1H), 4.12 (td, *J* = 11.2, 4.8 Hz, 1H), 3.73–3.63 (m, 2H), 3.40 (qd, *J* = 6.5, 4.7 Hz, 1H), 3.30–3.24 (m, 1H), 2.44 (d, *J* = 5.2 Hz, 1H); $^{13}C\{^1H\}$ NMR (101 MHz, $CDCl_3$) δ 145.7, 133.5, 122.3, 121.2, 114.3, 108.7, 69.6, 47.5, 46.6; HRMS (EI): calculated for $C_9H_{10}Br_2ClNO$ ($M^+$), 340.8818, found 340.8821.

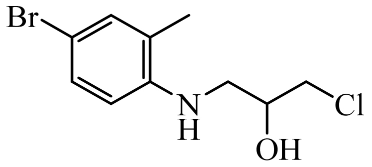

**S1l**

**1-((4-Bromo-2-methylphenyl)amino)-3-chloropropan-2-ol (S1l).** The preparation was carried out according on **Typical Procedure A**. Starting from 4-bromo-2-methylaniline (930 mg, 5 mmol), after purified by column chromatography [silica gel, *n*-hexane/ethyl acetate = 4/1 (v/v)], the title compound was obtained as a colorless liquid (766 mg, 55%). $R_f$ = 0.5 [*n*-hexane/ethyl acetate = 2/1 (v/v)]; $^1$H NMR (400 MHz, $CDCl_3$) δ 7.22–7.17 (m, 2H), 6.50 (d, $J$ = 8.6 Hz, 1H), 4.13–4.08 (m, 1H), 3.72–3.61 (m, 2H), 3.38 (dd, $J$ = 13.1, 4.3 Hz, 1H), 3.23 (dd, $J$ = 13.1, 7.3 Hz, 1H), 2.48 (s, 1H), 2.13 (s, 3H); $^{13}C\{^1H\}$ NMR (101 MHz, $CDCl_3$) δ 144.7, 132.7, 129.7, 124.8, 111.6, 109.5, 69.7, 47.8, 47.0, 17.2; HRMS (EI): calculated for $C_{10}H_{13}BrClNO$ ($M^+$), 276.9869, found 276.9864.

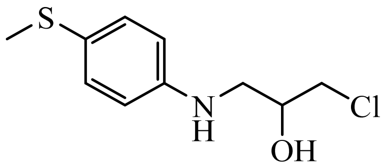

**S1m**

**1-Chloro-3-((4-(methylthio)phenyl)amino)propan-2-ol (S1m).** The preparation was carried out according on **Typical Procedure A**. Starting from 4-(methylthio)aniline (622 μL, 5 mmol), after purified by column chromatography [silica gel, *n*-hexane/ethyl acetate = 6/1 (v/v)], the title compound was obtained as a brick red liquid (428 mg, 37%). $R_f$ = 0.3 [*n*-hexane/ethyl acetate = 4/1 (v/v)]; $^1$H NMR (400 MHz, $CDCl_3$) 7.24–7.20 (m, 2H), 6.63–6.59 (m, 2H), 4.10–4.05 (m, 1H), 3.71–3.61 (m, 2H), 3.37 (dd, $J$ = 13.3, 4.4 Hz, 1H), 3.22 (dd, $J$ = 13.2, 7.1 Hz, 1H), 2.41 (s, 3H); $^{13}C\{^1H\}$ NMR (101 MHz, $CDCl_3$) δ 146.5, 131.2, 125.4, 113.9, 69.8, 47.6, 47.1, 18.8; HRMS (EI): calculated for $C_{10}H_{14}ClNOS$ ($M^+$), 231.0485, found 231.0480.

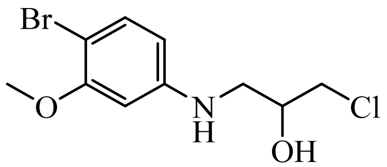

**S1n**

**1-((4-Bromo-3-methoxyphenyl)amino)-3-chloropropan-2-ol (S1n).** The preparation was carried out according on **Typical Procedure A**. Starting from 4-bromo-3-methoxyaniline (1.01 g, 5 mmol), after purified by column chromatography [silica gel, *n*-hexane/ethyl acetate = 5/1 (v/v)], the title compound was obtained as a brown solid (767 mg, 52%). $R_f$ = 0.4 [*n*-hexane/ethyl acetate = 3/1 (v/v)]; $^1$H NMR (400 MHz, $CDCl_3$) δ 7.28 (d, $J$ = 8.6 Hz, 1H), 6.24 (d, $J$ = 2.6 Hz, 1H), 6.16 (dd, $J$ = 8.6, 2.6 Hz, 1H), 4.11–4.05 (m,

1H), 3.85 (s, 3H), 3.71–3.62 (m, 2H), 3.37 (dd, $J$ = 13.1, 4.3 Hz, 1H), 3.22 (dd, $J$ = 13.1, 7.0 Hz, 1H); $^{13}C\{^1H\}$ NMR (101 MHz, $CDCl_3$) δ 156.5, 148.5, 133.4, 106.4, 99.3, 98.1, 69.9, 56.0, 47.6, 47.1; HRMS (EI): calculated for $C_{10}H_{13}BrClNO_2$ ($M^+$), 292.9818, found 292.9821; m.p. = 93–94 °C.

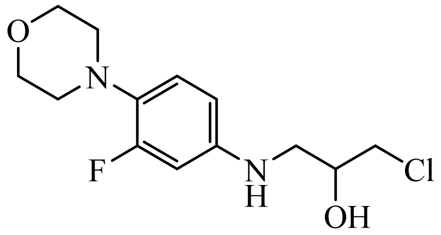

**S1o**

**1-Chloro-3-((3-fluoro-4-morpholinophenyl)amino)propan-2-ol (S1o).**[S5] The preparation was carried out according on **Typical Procedure A**. Starting from 3-fluoro-4-morpholinoaniline (981 mg, 5 mmol), after purified by column chromatography [silica gel, *n*-hexane/ethyl acetate = 4/1 (v/v)], the title compound was obtained as a pale brown liquid (814 mg, 56%). $R_f$ = 0.2 [*n*-hexane/ethyl acetate = 2/1 (v/v)]; $^1H$ NMR (400 MHz, $CDCl_3$) δ 6.86–6.77 (m, 1H), 6.45–6.37 (m, 2H), 4.09–4.03 (m, 1H), 3.85 (t, $J$ = 4.6 Hz, 4H), 3.71–3.61 (m, 2H), 3.32 (dd, $J$ = 13.1, 4.3 Hz, 1H), 3.18 (dd, $J$ = 13.1, 7.2 Hz, 1H), 2.97 (t, $J$ = 4.7 Hz, 4H); $^{13}C\{^1H\}$ NMR (101 MHz, $CDCl_3$) δ 156.8 (d, $J_{C\text{-}F}$ = 245.2 Hz), 144.5 (d, $J_{C\text{-}F}$ = 10.1 Hz), 131.0 (d, $J_{C\text{-}F}$ = 9.6 Hz), 120.2 (d, $J_{C\text{-}F}$ = 4.3 Hz), 108.7 (d, $J_{C\text{-}F}$ = 2.4 Hz), 101.9 (d, $J_{C\text{-}F}$ = 24.6 Hz), 69.6, 67.0, 51.6, 47.4, 47.3; $^{19}F$ NMR (376 MHz, $CDCl_3$) δ –125.8; HRMS (EI): calculated for $C_{13}H_{18}ClFN_2O_2$ ($M^+$), 288.1041, found 288.1031.

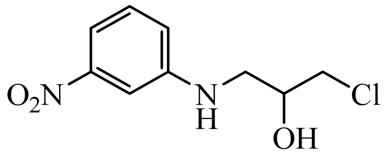

**S1p**

**1-Chloro-3-((3-nitrophenyl)amino)propan-2-ol (S1p).**[S6] The preparation was carried out according on **Typical Procedure A**. Starting from 3-nitroaniline (691 mg, 5 mmol), after purified by column chromatography [silica gel, *n*-hexane/ethyl acetate = 4/1 (v/v)], the title compound was obtained as a yellow liquid (669 mg, 58%). $R_f$ = 0.2 [*n*-hexane/ethyl acetate = 2/1 (v/v)]; $^1H$ NMR (400 MHz, $CDCl_3$) δ 7.55–7.52 (m, 1H), 7.43 (t, $J$ = 2.2 Hz, 1H), 7.30–7.26 (m, 1H), 6.94–6.92 (m, 1H), 4.16–4.10 (m, 1H), 3.74–3.63 (m, 2H), 3.44 (dd, $J$ = 13.1, 4.1 Hz, 1H), 3.28 (dd, $J$ = 13.1, 7.2 Hz, 1H); $^{13}C\{^1H\}$ NMR (101 MHz, $CDCl_3$) δ 149.2, 148.6, 129.8, 119.2, 112.5, 106.6, 69.7, 47.4, 46.6; HRMS (EI): calculated for $C_9H_{11}ClN_2O_3$ ($M^+$), 230.0458, found 230.0450.

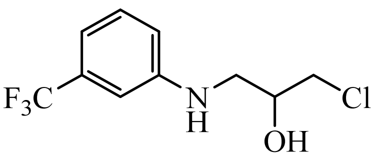

**S1q**

**1-Chloro-3-((3-(trifluoromethyl)phenyl)amino)propan-2-ol (S1q).**[S7]] The preparation was carried out according on **Typical Procedure A**. Starting from 3-(trifluoromethyl)aniline (624 μL, 5 mmol), after purified by column chromatography [silica gel, *n*-hexane/ethyl acetate = 4/1 (v/v)], the title compound was obtained as a colorless liquid (862 mg, 68%). $R_f$ = 0.5 [*n*-hexane/ethyl acetate = 2/1 (v/v)]; $^1$H NMR (400 MHz, $CDCl_3$) δ 7.29–7.25 (m, 1H), 6.98 (d, *J* = 7.6 Hz, 1H), 6.85–6.78 (m, 2H), 4.12–4.06 (m, 1H), 3.72–3.60 (m, 2H), 3.40 (dd, *J* = 13.1, 4.3 Hz, 1H), 3.25 (dd, *J* = 13.1, 7.3 Hz, 1H); $^{13}C\{^1H\}$ NMR (101 MHz, $CDCl_3$) δ 148.0, 131.6 (q, $J_{C-F}$ = 31.8 Hz), 129.7, 124.2 (q, $J_{C-F}$ = 272.5 Hz), 116.2, 114.5 (d, $J_{C-F}$ = 3.9 Hz), 109.3 (d, $J_{C-F}$ = 3.9 Hz), 69.7, 47.5, 46.7; $^{19}$F NMR (376 MHz, $CDCl_3$) δ –62.6; HRMS (ESI-TOF): calculated for $C_{10}H_{11}ClF_3NO+H^+$ [M+H$^+$] 254.0554, found 254.0556.

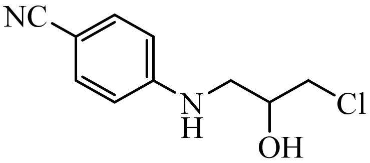

**S1r**

**4-((3-Chloro-2-hydroxypropyl)amino)benzonitrile (S1r).**[S8]] The preparation was carried out according on **Typical Procedure A**. Starting from 4-aminobenzonitrile (591 mg, 5 mmol), after purified by column chromatography [silica gel, *n*-hexane/ethyl acetate = 2/1 (v/v)], the title compound was obtained as a pale yellow liquid (506 mg, 48%). $R_f$ = 0.2 [*n*-hexane/ethyl acetate = 2/1 (v/v)]; $^1$H NMR (400 MHz, $CDCl_3$) δ 7.40 (d, *J* = 8.7 Hz, 2H), 6.61 (d, *J* = 8.7 Hz, 2H), 4.11–4.05 (m, 1H), 3.68–3.59 (m, 2H), 3.43 (dd, *J* = 13.4, 4.1 Hz, 1H), 3.29–3.22 (m, 1H); $^{13}C\{^1H\}$ NMR (101 MHz, $CDCl_3$) δ 151.1, 133.7, 120.3, 112.5, 98.9, 69.5, 47.1, 45.9; HRMS (ESI-TOF): calculated for $C_{10}H_{11}ClN_2O+H^+$ [M+H$^+$] 211.0633, found 211.0635.

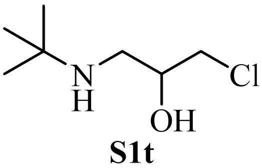

**S1t**

**1-(*tert*-Butylamino)-3-chloropropan-2-ol (S1t).**[S3] To a solution of *tert*-butylamine (1.3 mL, 12 mmol) in isopropanol (30 mL) was added epichlorohydrin (784 μL, 10 mmol, 1 equiv), and the mixture was stirred at room temperature for 24 hours. Upon completion, the reaction mixture was concentrated under reduced pressure to afford the crude compound **S1t** as a colorless liquid (954 mg, 48%). $^1$H NMR (400 MHz, $CDCl_3$) δ 3.85–3.79 (m, 1H), 3.61–3.53 (m, 2H), 2.84 (dd, *J* = 12.1, 4.0 Hz, 1H), 2.64 (dd, *J* = 12.1, 7.8 Hz, 1H),

1.14 (s, 9H); $^{13}C\{^1H\}$ NMR (101 MHz, $CDCl_3$) δ 69.4, 51.2, 47.1, 44.9, 28.7; HRMS (ESI-TOF): calculated for $C_7H_{16}ClNO+H^+$ [$M+H^+$] 166.0993, found 166.0995.

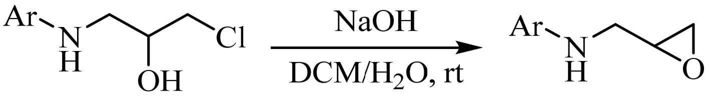


**S1** **1**

**Typical Procedure B** for the synthesis of epoxy amine **1**.

To a solution of **S1** (1 mmol) in DCM (3.3 mL) was added 0.3 N aqueous sodium hydroxide (6.6 mL) and left to be stirred for 24 hours at room temperature. Upon completion of the reaction, as determined by TLC analysis, the mixture was extracted with DCM (10 mL × 3), dried over anhydrous $MgSO_4$, and concentrated under reduced pressure to afford compound **1**.

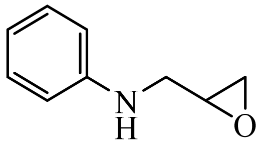


**1a**

***N*-(Oxiran-2-ylmethyl)aniline (1a).**[S1] The preparation was carried out according on **Typical Procedure B**. Starting from **S1a** (186 mg, 1 mmol), the title compound was obtained as a colorless liquid (148 mg, 99%). $R_f$ = 0.5 [*n*-hexane/ethyl acetate = 4/1 (v/v)]; $^1H$ NMR (400 MHz, $CDCl_3$) δ 7.21–7.16 (m, 2H), 6.74 (t, $J$ = 7.3 Hz, 1H), 6.65 (dd, $J$ = 8.5, 0.8 Hz, 2H), 3.86 (s, 1H), 3.56–3.51 (m, 1H), 3.27–3.20 (m, 2H), 2.83–2.81 (m, 1H), 2.70 (q, $J$ = 2.4 Hz, 1H); $^{13}C\{^1H\}$ NMR (101 MHz, $CDCl_3$) δ 147.8, 129.3, 117.9, 112.9, 51.0, 45.3, 45.0; HRMS (EI): calculated for $C_9H_{11}NO$ ($M^+$), 149.0841, found 149.0847.

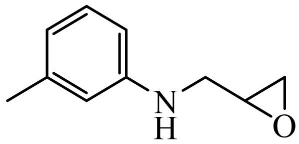


**1b**

**3-Methyl-*N*-(oxiran-2-ylmethyl)aniline (1b).**[S1] The preparation was carried out according on **Typical Procedure B**. Starting from **S1b** (199 mg, 1 mmol), the title compound was obtained as a pale yellow liquid (160 mg, 98%). $R_f$ = 0.5 [*n*-hexane/ethyl acetate = 2/1 (v/v)]; $^1H$ NMR (400 MHz, $CDCl_3$) δ 7.09–7.05 (m, 1H), 6.57–6.55 (m, 1H), 6.46 (d, $J$ = 7.5 Hz, 2H), 3.81 (s, 1H), 3.53–3.49 (m, 1H), 3.25–3.18 (m, 2H), 2.82–2.80 (m, 1H), 2.69 (q, $J$ = 2.5 Hz, 1H), 2.28 (s, 3H); $^{13}C\{^1H\}$ NMR (101 MHz, $CDCl_3$) δ 147.8, 139.1, 129.2, 118.8, 113.8, 110.1, 51.0, 45.4, 45.0, 21.6; HRMS (EI): calculated for $C_{10}H_{13}NO$ ($M^+$), 163.0997, found 163.0990.

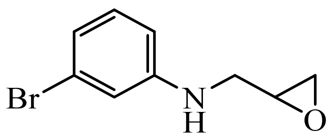

**1c**

**3-Bromo-*N*-(oxiran-2-ylmethyl)aniline (1c).**[S3] The preparation was carried out according on **Typical Procedure B**. Starting from **S1c** (228 mg, 1 mmol), the title compound was obtained as an orange liquid (224 mg, 98%). $R_f$ = 0.4 [*n*-hexane/ethyl acetate = 4/1 (v/v)]; $^1$H NMR (400 MHz, $CDCl_3$) δ 7.02 (t, $J$ = 8.0 Hz, 1H), 6.84 (d, $J$ = 7.2 Hz, 1H), 6.77 (d, $J$ = 1.8 Hz, 1H), 6.55 (d, $J$ = 8.1 Hz, 1H), 3.95 (s, 1H), 3.55–3.51 (m, 1H), 3.19 (d, $J$ = 5.3 Hz, 2H), 2.82 (t, $J$ = 4.2 Hz, 1H), 2.68 (t, $J$ = 2.3 Hz, 1H); $^{13}C\{^1H\}$ NMR (101 MHz, $CDCl_3$) δ 149.1, 130.5, 123.3, 120.6, 115.4, 111.8, 50.7, 45.2, 44.7; HRMS (EI): calculated for $C_9H_{10}BrNO$ ($M^+$), 226.9946, found 226.9940.

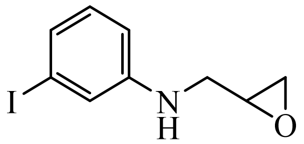

**1d**

**3-Iodo-*N*-(oxiran-2-ylmethyl)aniline (1d).** The preparation was carried out according on **Typical Procedure B**. Starting from **S1d** (312 mg, 1 mmol), the title compound was obtained as a yellow liquid (261 mg, 95%). $R_f$ = 0.6 [*n*-hexane/ethyl acetate = 2/1 (v/v)]; $^1$H NMR (400 MHz, $CDCl_3$) δ 7.04 (dq, $J$ = 7.8, 0.8 Hz, 1H), 6.98–6.97 (m, 1H), 6.87 (t, $J$ = 8.0 Hz, 1H), 6.58 (ddd, $J$ = 8.2, 2.3, 0.8 Hz, 1H), 3.91 (s, 1H), 3.54–3.49 (m, 1H), 3.20–3.15 (m, 2H), 2.83–2.80 (m, 1H), 2.67 (q, $J$ = 2.4 Hz, 1H); $^{13}C\{^1H\}$ NMR (101 MHz, $CDCl_3$) δ 149.0, 130.7, 126.7, 121.4, 112.3, 95.2, 50.7, 45.2, 44.6; HRMS (EI): calculated for $C_9H_{10}INO$ ($M^+$), 274.9807, found 274.9800; m.p. = 67–68 °C.

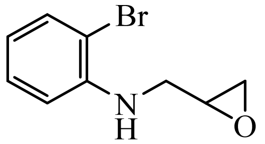

**1e**

**2-Bromo-*N*-(oxiran-2-ylmethyl)aniline (1e).** The preparation was carried out according on **Typical Procedure B**. Starting from **S1e** (265 mg, 1 mmol), the title compound was obtained as a yellow liquid (228 mg, 99%). $R_f$ = 0.7 [*n*-hexane/ethyl acetate = 3/1 (v/v)]; $^1$H NMR (400 MHz, $CDCl_3$) δ 7.43–7.40 (m, 1H), 7.18–7.14 (m, 1H), 6.68 (dd, $J$ = 8.3, 1.4 Hz, 1H), 6.60–6.56 (m, 1H), 4.51 (s, 1H), 3.54 (ddd, $J$ = 14.2, 5.5, 3.1 Hz, 1H), 3.27 (dt, $J$ = 14.1, 5.3 Hz, 1H), 3.20–3.17 (m, 1H), 2.80 (dd, $J$ = 4.8, 4.1 Hz, 1H), 2.66 (q, $J$ = 2.5 Hz, 1H); $^{13}C\{^1H\}$ NMR (101 MHz, $CDCl_3$) δ 144.5, 132.4, 128.4, 118.2, 111.3, 109.8, 50.7, 45.1, 44.8; HRMS (EI): calculated for $C_9H_{10}BrNO$ ($M^+$), 226.9946, found 226.9950.

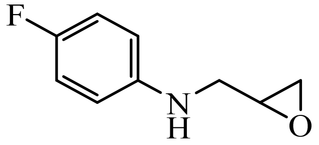

**1f**

**4-Fluoro-*N*-(oxiran-2-ylmethyl)aniline (1f).**[S1] The preparation was carried out according on **Typical Procedure B**. Starting from **S1f** (204 mg, 1 mmol), the title compound was obtained as a colorless liquid (167 mg, 99%). $R_f$ = 0.5 [*n*-hexane/ethyl acetate = 4/1 (v/v)]; $^1$H NMR (400 MHz, $CDCl_3$) δ 6.92–6.86 (m, 2H), 6.60–6.55 (m, 2H), 3.76 (s, 1H), 3.53–3.49 (m, 1H), 3.22–3.13 (m, 2H), 2.82 (dd, *J* = 4.8, 4.1 Hz, 1H), 2.69 (q, *J* = 2.5 Hz, 1H); $^{13}$C{$^1$H} NMR (101 MHz, $CDCl_3$) δ 156.1 (d, $J_{C\text{-}F}$ = 235.5 Hz), 144.1, 115.7 (d, $J_{C\text{-}F}$ = 22.6 Hz), 113.9 (d, $J_{C\text{-}F}$ = 7.2 Hz), 50.9, 45.7, 45.3; $^{19}$F NMR (376 MHz, $CDCl_3$) δ –127.4; HRMS (EI): calculated for $C_9H_{10}FNO$ ($M^+$), 167.0746, found 167.0740.

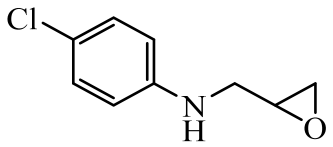

**1g**

**4-Chloro-*N*-(oxiran-2-ylmethyl)aniline (1g).**[S1] The preparation was carried out according on **Typical Procedure B**. Starting from **S1g** (220 mg, 1 mmol), the title compound was obtained as a yellow liquid (182 mg, 99%). $R_f$ = 0.3 [*n*-hexane/ethyl acetate = 3/1 (v/v)]; $^1$H NMR (400 MHz, $CDCl_3$) δ 7.14–7.11 (m, 2H), 6.58–6.54 (m, 2H), 3.88 (s, 1H), 3.56–3.50 (m, 1H), 3.22–3.15 (m, 2H), 2.83–2.81 (m, 1H), 2.68 (q, *J* = 2.4 Hz, 1H); $^{13}$C{$^1$H} NMR (101 MHz, $CDCl_3$) δ 146.4, 129.1, 122.5, 114.0, 50.8, 45.3, 45.1; HRMS (EI): calculated for $C_9H_{10}ClNO$ ($M^+$), 183.0451, found 183.0456.

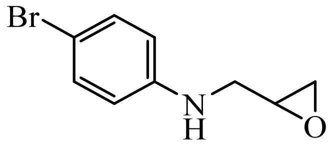

**1h**

**4-Bromo-*N*-(oxiran-2-ylmethyl)aniline (1h).**[S1] The preparation was carried out according on **Typical Procedure B**. Starting from **S1h** (265 mg, 1 mmol), the title compound was obtained as a pale yellow solid (216 mg, 95%). $R_f$ = 0.4 [*n*-hexane/ethyl acetate = 2/1 (v/v)]; $^1$H NMR (400 MHz, $CDCl_3$) δ 7.27–7.23 (m, 2H), 6.53–6.50 (m, 2H), 3.55–3.50 (m, 1H), 3.21–3.15 (m, 2H), 2.83–2.81 (m, 1H), 2.67 (q, *J* = 2.3 Hz, 1H); $^{13}$C{$^1$H} NMR (101 MHz, $CDCl_3$) δ 146.8, 132.0, 114.5, 109.5, 50.8, 45.2, 44.9; HRMS (EI): calculated for $C_9H_{10}BrNO$ ($M^+$), 226.9946, found 226.9940; m.p. = 50–51 °C.

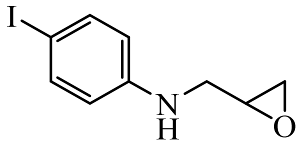

**1i**

**4-Iodo-*N*-(oxiran-2-ylmethyl)aniline (1i).**[S9] The preparation was carried out according on **Typical Procedure B**. Starting from **S1i** (312 mg, 1 mmol), the title compound was obtained as a green solid (267 mg, 97%). $R_f$ = 0.5 [*n*-hexane/ethyl acetate = 2/1 (v/v)]; $^{1}$H NMR (400 MHz, $CDCl_3$) 7.43–7.40 (m, 2H), 6.43–6.40 (m, 2H), 3.92 (s, 1H), 3.54–3.48 (m, 1H), 3.19–3.14 (m, 2H), 2.82–2.80 (m, 1H), 2.66 (q, *J* = 2.4 Hz, 1H); $^{13}$C{$^{1}$H} NMR (101 MHz, $CDCl_3$) δ 147.4, 137.8, 115.1, 78.5, 50.7, 45.2, 44.7; HRMS (EI): calculated for $C_9H_{10}INO$ ($M^+$), 274.9807, found 274.9800; m.p. = 88–90 °C.

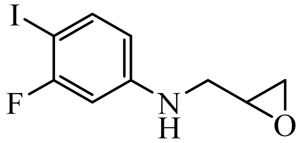

**1j**

**3-Fluoro-4-iodo-*N*-(oxiran-2-ylmethyl)aniline (1j).** The preparation was carried out according on **Typical Procedure B**. Starting from **S1j** (330 mg, 1 mmol), the title compound was obtained as a white solid (284 mg, 97%). $R_f$ = 0.4 [*n*-hexane/ethyl acetate = 2/1 (v/v)]; $^{1}$H NMR (400 MHz, $CDCl_3$) δ 7.41 (dd, *J* = 8.6, 7.2 Hz, 1H), 6.38 (dd, *J* = 10.5, 2.7 Hz, 1H), 6.23 (dd, *J* = 8.6, 2.6 Hz, 1H), 4.08 (s, 1H), 3.56–3.51 (m, 1H), 3.20–3.14 (m, 2H), 2.83–2.81 (m, 1H), 2.66 (q, *J* = 2.4 Hz, 1H); $^{13}$C{$^{1}$H} NMR (101 MHz, $CDCl_3$) δ 162.5 (d, $J_{C\text{-}F}$ = 242.3 Hz), 149.9 (d, $J_{C\text{-}F}$ = 9.6 Hz), 139.0 (d, $J_{C\text{-}F}$ = 3.4 Hz), 111.1 (d, $J_{C\text{-}F}$ = 2.4 Hz), 100.1 (d, $J_{C\text{-}F}$ = 27.9 Hz), 64.5 (d, $J_{C\text{-}F}$ = 26.0 Hz), 50.6, 45.1, 44.7; $^{19}$F NMR (376 MHz, $CDCl_3$) δ –93.8; HRMS (EI): calculated for $C_9H_9FINO$ ($M^+$), 292.9713, found 292.9720; m.p. = 61–62 °C.

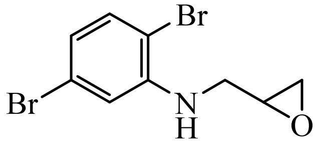

**1k**

**2,5-Dibromo-*N*-(oxiran-2-ylmethyl)aniline (1k).** The preparation was carried out according on **Typical Procedure B**. Starting from **S1k** (343 mg, 1 mmol), the title compound was obtained as a colorless liquid (289 mg, 94%). $R_f$ = 0.6 [*n*-hexane/ethyl acetate = 4/1 (v/v)]; $^{1}$H NMR (400 MHz, $CDCl_3$) δ 7.27–7.25 (m, 1H), 6.80 (d, *J* = 2.0 Hz, 1H), 6.71 (dd, *J* = 8.3, 2.2 Hz, 1H), 4.57 (s, 1H), 3.62–3.57 (m, 1H), 3.30–3.23 (m, 2H), 2.87–2.84 (m, 1H), 2.69 (q, *J* = 2.3 Hz, 1H); $^{13}$C{$^{1}$H} NMR (101 MHz, $CDCl_3$) 145.7, 133.4, 122.3, 121.0, 114.2, 108.4, 50.5, 45.1, 44.7; HRMS (EI): calculated for $C_9H_9Br_2NO$ ($M^+$), 304.9051, found 304.9056.

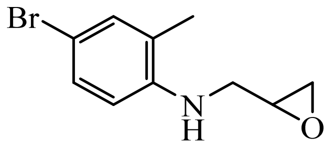

**1l**

**4-Bromo-2-methyl-*N*-(oxiran-2-ylmethyl)aniline (1l).** The preparation was carried out according on **Typical Procedure B**. Starting from **S1l** (279 mg, 1 mmol), the title compound was obtained as a colorless liquid (231 mg, 95%). $R_f$ = 0.6 [*n*-hexane/ethyl acetate = 4/1 (v/v)]; $^1$H NMR (400 MHz, $CDCl_3$) 7.22–7.16 (m, 2H), 6.50 (d, $J$ = 8.6 Hz, 1H), 3.72 (s, 1H), 3.60–3.56 (m, 1H), 3.25–3.20 (m, 2H), 2.85–2.83 (m, 1H), 2.69 (q, $J$ = 2.4 Hz, 1H), 2.12 (s, 3H); $^{13}$C{$^1$H} NMR (101 MHz, $CDCl_3$) δ 144.8, 132.6, 129.6, 124.5, 111.3, 109.2, 50.8, 45.3, 44.9, 17.2; HRMS (EI): calculated for $C_{10}H_{12}BrNO$ ($M^+$), 241.0102, found 241.0109; m.p. = 36–37 °C.

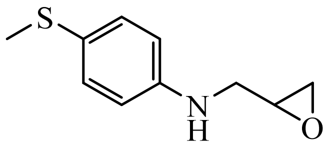

**1m**

**4-(Methylthio)-*N*-(oxiran-2-ylmethyl)aniline (1m).** The preparation was carried out according on **Typical Procedure B**. Starting from **S1m** (232 mg, 1 mmol), the title compound was obtained as a pink liquid (186 mg, 95%). $R_f$ = 0.5 [*n*-hexane/ethyl acetate = 1/1 (v/v)]; $^1$H NMR (400 MHz, $CDCl_3$) δ 7.23–7.20 (m, 2H), 6.61–6.57 (m, 2H), 3.90 (s, 1H), 3.55–3.50 (m, 1H), 3.24–3.18 (m, 2H), 2.82 (dd, $J$ = 4.8, 4.1 Hz, 1H), 2.68 (q, $J$ = 2.4 Hz, 1H), 2.41 (s, 3H); $^{13}$C{$^1$H} NMR (101 MHz, $CDCl_3$) δ 146.6, 131.3, 125.0, 113.6, 50.9, 45.3, 45.0, 18.9; HRMS (EI): calculated for $C_{10}H_{13}NOS$ ($M^+$), 195.0718, found 195.0710.

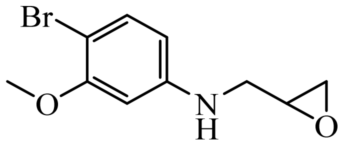

**1n**

**4-Bromo-3-methoxy-*N*-(oxiran-2-ylmethyl)aniline (1n).** The preparation was carried out according on **Typical Procedure B**. Starting from **S1n** (295 mg, 1 mmol), the title compound was obtained as a pale brown liquid (256 mg, 99%). $R_f$ = 0.4 [*n*-hexane/ethyl acetate = 1/1 (v/v)]; $^1$H NMR (400 MHz, $CDCl_3$) δ 7.28 (q, $J$ = 4.2 Hz, 1H), 6.22 (d, $J$ = 2.6 Hz, 1H), 6.14 (dd, $J$ = 8.5, 2.5 Hz, 1H), 3.95 (s, 1H), 3.85 (s, 3H), 3.58–3.51 (m, 1H), 3.22–3.16 (m, 2H), 2.83–2.82 (m, 1H), 2.69 (q, $J$ = 2.4 Hz, 1H); $^{13}$C{$^1$H} NMR (101 MHz, $CDCl_3$) δ 156.5, 148.6, 133.3, 106.0, 98.9, 97.8, 56.0, 50.8, 45.2, 45.1; HRMS (EI): calculated for $C_{10}H_{12}BrNO_2$ ($M^+$), 257.0051, found 257.0059.

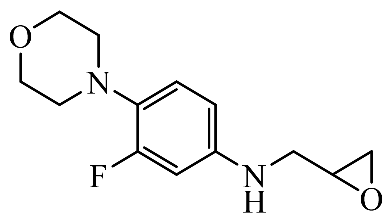

**1o**

**3-Fluoro-4-morpholino-*N*-(oxiran-2-ylmethyl)aniline (1o).**[S8] The preparation was carried out according on **Typical Procedure B**. Starting from **S1o** (289 mg, 1 mmol), the title compound was obtained as a yellow liquid (246 mg, 98%). $R_f$ = 0.2 [*n*-hexane/ethyl acetate = 2/1 (v/v)]; $^1$H NMR (400 MHz, $CDCl_3$) δ 6.83 (t, *J* = 9.1 Hz, 1H), 6.42–6.35 (m, 2H), 3.85 (t, *J* = 4.6 Hz, 4H), 3.50 (d, *J* = 11.2 Hz, 1H), 3.21–3.13 (m, 2H), 2.97 (t, *J* = 4.7 Hz, 4H), 2.83–2.81 (m, 1H), 2.68 (q, *J* = 2.4 Hz, 1H); $^{13}$C{$^1$H} NMR (101 MHz, $CDCl_3$) δ 156.9 (d, $J_{C\text{-}F}$ = 245.2 Hz), 144.6 (d, $J_{C\text{-}F}$ = 10.1 Hz), 131.0 (d, $J_{C\text{-}F}$ = 9.6 Hz), 120.3 (d, $J_{C\text{-}F}$ = 4.3 Hz), 108.5 (d, $J_{C\text{-}F}$ = 2.4 Hz), 101.6 (d, $J_{C\text{-}F}$ = 24.6 Hz), 67.1, 51.7, 50.8, 45.3, 45.2; $^{19}$F NMR (376 MHz, $CDCl_3$) δ –122.3; HRMS (EI): calculated for $C_{13}H_{17}FN_2O_2$ ($M^+$), 252.1274, found 252.1266.

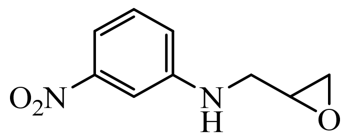

**1p**

**3-Nitro-*N*-(oxiran-2-ylmethyl)aniline (1p).** The preparation was carried out according on **Typical Procedure B**. Starting from **S1p** (231 mg, 1 mmol), the title compound was obtained as a yellow liquid (181 mg, 93%). $R_f$ = 0.2 [*n*-hexane/ethyl acetate = 2/1 (v/v)]; $^1$H NMR (400 MHz, $CDCl_3$) δ 7.56 (ddd, *J* = 8.1, 2.1, 0.8 Hz, 1H), 7.44 (t, *J* = 2.3 Hz, 1H), 7.31–7.26 (m, 1H), 6.92 (ddd, *J* = 8.2, 2.4, 0.8 Hz, 1H), 4.24 (s, 1H), 3.68–3.62 (m, 1H), 3.30–3.22 (m, 2H), 2.87–2.85 (m, 1H), 2.71 (q, *J* = 2.4 Hz, 1H); $^{13}$C{$^1$H} NMR (101 MHz, $CDCl_3$) δ 149.4, 148.6, 129.8, 119.1, 112.5, 106.4, 50.5, 45.2, 44.7; HRMS (EI): calculated for $C_9H_{10}N_2O_3$ ($M^+$), 194.0691, found 194.0685.

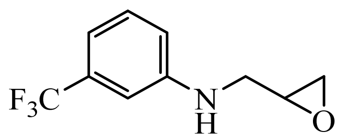

**1q**

***N*-(Oxiran-2-ylmethyl)-3-(trifluoromethyl)aniline (1q).** The preparation was carried out according on **Typical Procedure B**. Starting from **S1q** (254 mg, 1 mmol), the title compound was obtained as a colorless liquid (202 mg, 93%). $R_f$ = 0.6 [*n*-hexane/ethyl acetate = 2/1 (v/v)]; $^1$H NMR (400 MHz, $CDCl_3$) δ 7.28–7.24 (m, 1H), 6.96 (d, *J* = 7.6 Hz, 1H), 6.83 (s, 1H), 6.78 (d, *J* = 8.3 Hz, 1H), 4.09 (s, 1H), 3.61–3.56 (m,

1H), 3.27–3.20 (m, 2H), 2.85–2.82 (m, 1H), 2.70 (q, $J$ = 2.4 Hz, 1H); $^{13}C\{^1H\}$ NMR (101 MHz, $CDCl_3$) δ 148.0, 131.6 (q, $J_{C\text{-}F}$ = 31.8 Hz), 129.7, 124.2 (q, $J_{C\text{-}F}$ = 272.3 Hz), 116.0, 114.3 (d, $J_{C\text{-}F}$ = 3.9 Hz), 108.9 (d, $J_{C\text{-}F}$ = 3.9 Hz), 50.7, 45.2, 44.7; $^{19}F$-NMR (376 MHz, $CDCl_3$) δ –62.8; HRMS (ESI-TOF): calculated for $C_{10}H_{10}F_3NO+H^+$ [$M+H^+$] 218.0787, found 218.0790.

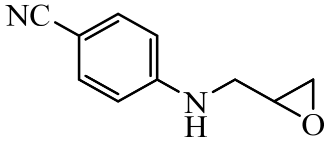

**1r**

**4-((Oxiran-2-ylmethyl)amino)benzonitrile (1r).**[S8] The preparation was carried out according on **Typical Procedure B**. Starting from **S1r** (211 mg, 1 mmol), the title compound was obtained as a pale yellow liquid (153 mg, 88%). $R_f$ = 0.2 [*n*-hexane/ethyl acetate = 2/1 (v/v)]; $^1H$ NMR (400 MHz, $CDCl_3$) δ 7.44 (dt, $J$ = 9.2, 2.2 Hz, 2H), 6.62 (dt, $J$ = 9.2, 2.2 Hz, 2H), 3.63 (dd, $J$ = 14.1, 2.4 Hz, 1H), 3.28–3.19 (m, 2H), 2.85 (t, $J$ = 4.4 Hz, 1H), 2.67 (q, $J$ = 2.4 Hz, 1H); $^{13}C\{^1H\}$ NMR (101 MHz, $CDCl_3$) δ 151.0, 133.7, 120.2, 112.4, 99.4, 50.4, 45.1, 44.1; HRMS (ESI-TOF): calculated for $C_{10}H_{10}N_2O+H^+$ [$M+H^+$] 175.0866, found 175.0867.

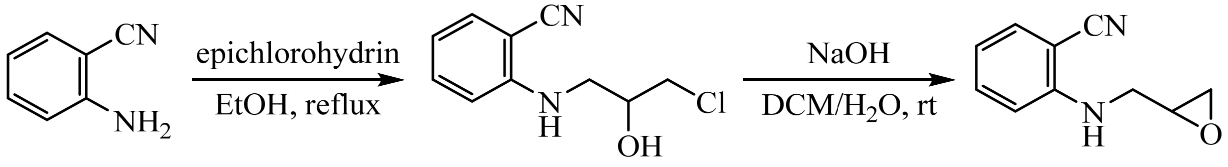


**S1s** + inseparable **1s** **1s**

**2-((Oxiran-2-ylmethyl)amino)benzonitrile (1s).** The preparation was carried out according on **Typical Procedure B**. To a solution of 2-aminobenzonitrile (591 mg, 5 mmol) in EtOH (10 mL) was added epichlorohydrin (392 μL, 5 mmol, 1 equiv) and was stirred at reflux for 12 hours. Upon completion of the reaction, as determined by TLC analysis, the mixture was concentrated under reduced pressure and purified by column chromatography [silica gel, *n*-hexane/ethyl acetate = 5/1 (v/v)] to afford compound **S1s** (colorless liquid, maximum 261 mg, 25%), along with inseparable **1s**. $R_f$ = 0.4 [*n*-hexane/ethyl acetate = 2/1 (v/v)]; $^1H$ NMR (400 MHz, $CDCl_3$) δ 7.43–7.39 (m, 2H), 6.78–6.70 (m, 2H), 4.13–4.09 (m, 1H), 3.73–3.59 (m, 2H), 3.56–3.46 (m, 1H), 3.38–3.31 (m, 1H); $^{13}C\{^1H\}$ NMR (101 MHz, $CDCl_3$) δ 149.9, 134.3, 132.9, 117.7, 117.3, 110.9, 96.6, 69.7, 47.3, 46.2; HRMS (ESI-TOF): calculated for $C_{10}H_{11}ClN_2O+H^+$ [$M+H^+$] 211.0633, found 211.0634.

Starting from **S1s** (211 mg, 1 mmol), the title compound was obtained as a colorless liquid (159 mg, 91%). $R_f$ = 0.4 [*n*-hexane/ethyl acetate = 2/1 (v/v)]; $^1H$ NMR (400 MHz, $CDCl_3$) δ 7.43–7.38 (m, 2H), 6.76 (t, $J$ = 4.4 Hz, 1H), 6.72 (td, $J$ = 7.6, 0.8 Hz, 1H), 4.78 (s, 1H), 3.63 (ddd, $J$ = 14.4, 5.9, 3.2 Hz, 1H), 3.38–3.31 (m, 1H), 3.22–3.19 (m, 1H), 2.85 (dd, $J$ = 4.7, 4.1 Hz, 1H), 2.69 (q, $J$ = 2.4 Hz, 1H); $^{13}C\{^1H\}$ NMR (101

MHz, $CDCl_3$) δ 149.9, 134.3, 132.8, 117.7, 117.2, 110.9, 96.2, 50.6, 45.1, 44.6; HRMS (ESI-TOF): calculated for $C_{10}H_{10}N_2O+H^+$ [$M+H^+$] 175.0866, found 175.0865.

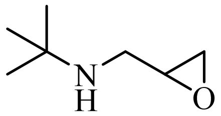

**1t**

**2-Methyl-*N*-(oxiran-2-ylmethyl)propan-2-amine (1t).**[S10] To a solution of **S1t** (495 mg, 3 mmol) in DCM (9 mL) was added 4 N aqueous sodium hydroxide (18 mL), and the mixture was stirred at room temperature for 16 hours. Upon completion, the reaction mixture was extracted with DCM (10 mL × 3), dried over anhydrous $MgSO_4$, and purified by distillation to afford compound **1t** as a pale yellow liquid (167 mg, 43%). $^1$H NMR (400 MHz, $CDCl_3$) δ 3.10 (td, $J$ = 6.4, 3.7 Hz, 1H), 2.87–2.84 (m, 1H), 2.79 (t, $J$ = 4.5 Hz, 1H), 2.62–2.57 (m, 2H), 1.10 (s, 9H); $^{13}$C{$^1$H} NMR (101 MHz, $CDCl_3$) δ 52.3, 50.2, 45.9, 45.0, 28.9.

**Table S1.** Optimization experiment

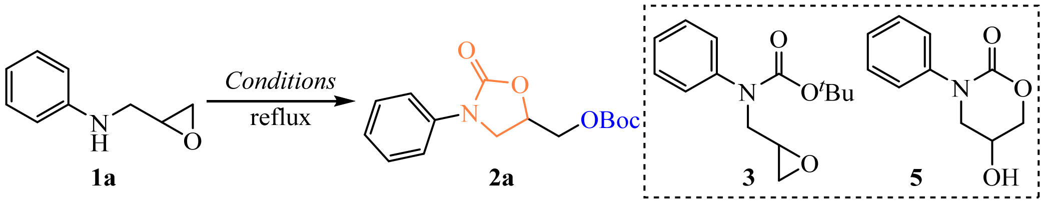


| Entry | Equiv of Boc$_2$O | Equiv of Et$_3$N | Additive (equiv) | Solvent | Time (h) | Product (% yield)$^a$ |
|---|---|---|---|---|---|---|
| **1** | 1.5 | 1.1 | DMAP (0.1) | THF | 16 | **2a** (17) |
| **2** | 1.5 | 1.1 | – | THF | 16 | **2a** (messy) |
| **3** | 1.5 | 1.1 | DMAP (0.1) | DCM | 16 | **2a** (29) |
| **4** | 1.5 | 1.1 | – | DCM | 16 | **2a** (74) |
| **5** | 2 | 2 | – | DCM | 16 | **2a** (78) |
| **6** | 2 | 2 | – | DCM | 24 | **2a** (78) |
| **7** | 2 | – | – | DCM | 16 | **3** (85) |
| **8** | 2 | – | $NH_4Cl$ (2) | DCM | 14 | **3** (81) |
| **9** | 2 | – | TBAB (2) | DCM | 14 | **2a** (45) |

| | | | | | | |
|---|---|---|---|---|---|---|
| | | | | | | **5** (18) |
| **10** | 2 | – | TBAI (2) | DCM | 14 | **2a** (42)<br>**5** (15) |
| **11** | 2 | – | $BnNEt_3Cl$ (2) | DCM | 14 | **2a** (messy) |
| **12** | 1.5 | 2 | – | DCM | 14 | **2a** (77)<br>**3** (messy) |
| **13** | 1.5 | 0 | TBAB (2) | DCM | 14 | **2a** (25)<br>**5** (30) |
| **14** | 1.5 | 2 | TBAB (2) | DCM | 14 | **2a** (78) |
| **15** | 1.1 | 3 | – | DCM | 14 | **2a** (77) |
| **16** | 1.1 | – | TBAB (3) | DCM | 14 | **5** (72) |
| **17** | 1.3 | 3 | – | DCM | 14 | **2a** (78)<br>**3** (messy) |
| **18** | 1.3 | 3 | | EtOAc | 14 | **2a** (42) |
| **19** | 1.3 | 3 | | MTBE | 14 | **2a** (69) |
| **20** | 1.3 | | $K_2CO_3$ | DCM | 14 | **3** (85) |
| **21** | 1.3 | | $NaHCO_3$ | DCM | 14 | **3** (62) |

[a]Isolated yield.

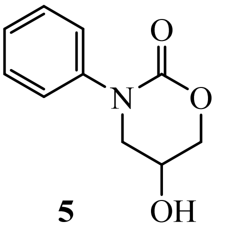


**5**

**5-Hydroxy-3-phenyl-1,3-oxazinan-2-one (5).**[S3] To a solution of **1a** (75 mg, 0.5 mmol) in DCM (1.0 mL) was added di-*tert*-butyl dicarbonate ($Boc_2O$, 126 µL, 0.55 mmol, 1.1 equiv), TBAB (484 mg, 1.5 mmol, 3 equiv) and was stirred at reflux for 14 hours. Upon completion of the reaction, as determined by TLC analysis, the mixture was concentrated under reduced pressure, and purified by column chromatography [silica gel, *n*-hexane/ethyl acetate = 4/1 (v/v)] to afford the title compound (70 mg, 72%) as a colorless liquid. $R_f$ = 0.17 [*n*-hexane/ethyl acetate = 2/1 (v/v)]; $^1$H NMR (400 MHz, $CDCl_3$) δ 7.19–7.12 (m, 2H),

6.72 (t, $J$ = 7.3 Hz, 1H), 6.58 (d, $J$ = 7.9 Hz, 2H), 4.90–4.84 (m, 1H), 4.48 (t, $J$ = 8.3 Hz, 1H), 4.22 (dd, $J$ = 8.4, 6.9 Hz, 1H), 3.90 (s, 1H), 3.47 (ddd, $J$ = 14.2, 6.6, 4.5 Hz, 1H), 3.40–3.33 (m, 1H); $^{13}C\{^1H\}$ NMR (101 MHz, $CDCl_3$) (101 MHz, $CDCl_3$) δ 154.7, 146.8, 129.5, 118.9, 113.2, 75.2, 67.0, 45.8; HRMS (EI): calculated for $C_{10}H_{11}NO_3$ ($M^+$), 193.0739, found 193.0734.

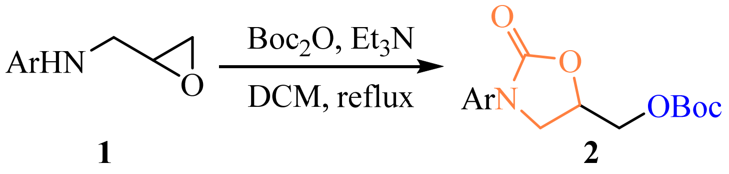

**Typical Procedure C** for the synthesis of compound **2**.

To a solution of **1** (0.5 mmol) in DCM (1.0 mL) was added $Boc_2O$ (126 µL, 0.55 mmol, 1.1 equiv), triethylamine (209 µL, 1.5 mmol, 3 equiv) and was stirred at reflux for 14 hours. Upon completion of the reaction, as determined by TLC analysis, the mixture was concentrated under reduced pressure, and purified by column chromatography to afford compound **2**.

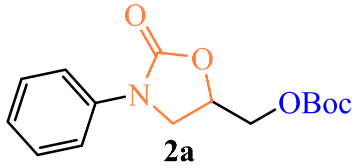

**2a**

***N*-(Oxiran-2-ylmethyl)aniline (1a).** The preparation was carried out according on **Typical Procedure C**. Starting from **S1a** (75 mg, 0.5 mmol), after purified by column chromatography [silica gel, *n*-hexane/ethyl acetate = 4/1 (v/v)], the title compound was obtained as a white solid (114 mg, 78%). $R_f$ = 0.3 [*n*-hexane/ethyl acetate = 4/1 (v/v)]; $^1H$ NMR (400 MHz, $CDCl_3$) δ 7.55–7.52 (m, 2H), 7.40–7.36 (m, 2H), 7.15 (t, $J$ = 7.4 Hz, 1H), 4.89–4.83 (m, 1H), 4.32 (d, $J$ = 4.9 Hz, 2H), 4.13 (t, $J$ = 8.9 Hz, 1H), 3.89 (dd, $J$ = 9.0, 6.4 Hz, 1H), 1.48 (s, 9H); $^{13}C\{^1H\}$ NMR (101 MHz, $CDCl_3$) δ 154.1, 152.9, 137.9, 129.1, 124.2, 118.3, 83.2, 69.8, 66.0, 47.1, 27.6; HRMS (EI): calculated for $C_{15}H_{19}NO_5$ ($M^+$), 293.1263, found 293.1258. m.p. = 132–134 °C.

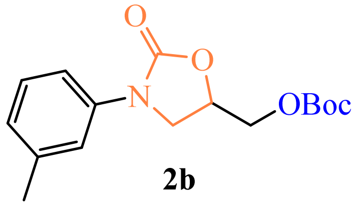

**2b**

***tert*-Butyl ((2-oxo-3-(*m*-tolyl)oxazolidin-5-yl)methyl) carbonate (2b).** The preparation was carried out according on **Typical Procedure C**. Starting from **1b** (82 mg, 0.5 mmol), after purified by column chromatography [silica gel, *n*-hexane/ethyl acetate = 4/1 (v/v)], the title compound was obtained as a pale

yellow solid (111 mg, 72%). R$_f$ = 0.3 [*n*-hexane/ethyl acetate = 4/1 (v/v)]; $^1$H NMR (400 MHz, $CDCl_3$) δ 7.39 (d, *J* = 0.5 Hz, 1H), 7.32–7.26 (m, 2H), 6.97 (dd, *J* = 7.2, 0.6 Hz, 1H), 4.88–4.82 (m, 1H), 4.31 (d, *J* = 4.9 Hz, 2H), 4.11 (t, *J* = 9.0 Hz, 1H), 3.88 (dd, *J* = 9.2, 6.4 Hz, 1H), 2.37 (s, 3H), 1.48 (s, 9H); $^{13}$C{$^1$H} NMR (101 MHz, $CDCl_3$) δ 154.1, 152.9, 139.1, 137.9, 128.9, 125.1, 119.1, 115.4, 83.2, 69.7, 66.1, 47.2, 27.6, 21.6; HRMS (EI): calculated for $C_{16}H_{21}NO_5$ ($M^+$), 307.1420, found 307.1423. m.p. = 98–100 °C.

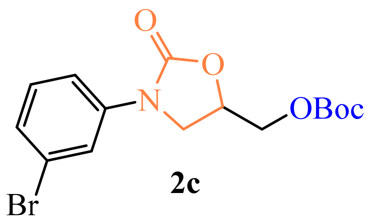


**2c**

**(3-(3-Bromophenyl)-2-oxooxazolidin-5-yl)methyl *tert*-butyl carbonate (2c).** The preparation was carried out according on **Typical Procedure C**. Starting from **1c** (114 mg, 0.5 mmol), after purified by column chromatography [silica gel, *n*-hexane/ethyl acetate = 4/1 (v/v)], the title compound was obtained as a pale orange solid (153 mg, 82%). R$_f$ = 0.3 [*n*-hexane/ethyl acetate = 4/1 (v/v)]; $^1$H NMR (400 MHz, $CDCl_3$) δ 7.71 (t, *J* = 1.9 Hz, 1H), 7.52 (ddd, *J* = 7.9, 2.2, 1.4 Hz, 1H), 7.29–7.22 (m, 2H), 4.90–4.84 (m, 1H), 4.36–4.28 (m, 2H), 4.10 (t, *J* = 8.9 Hz, 1H), 3.88 (dd, *J* = 9.0, 6.4 Hz, 1H), 1.48 (s, 9H); $^{13}$C{$^1$H} NMR (101 MHz, $CDCl_3$) δ 153.8, 152.9, 139.2, 130.4, 127.2, 122.8, 121.1, 116.6, 83.3, 69.9, 65.9, 46.9, 27.6; HRMS (EI): calculated for $C_{15}H_{18}BrNO_5$ ($M^+$), 371.0368, found 371.0363; m.p. = 131–132 °C.

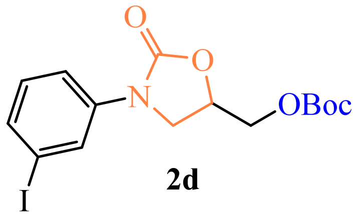


**2d**

***tert*-Butyl ((3-(3-iodophenyl)-2-oxooxazolidin-5-yl)methyl) carbonate (2d).** The preparation was carried out according on **Typical Procedure C**. Starting from **1d** (138 mg, 0.5 mmol), after purified by column chromatography [silica gel, *n*-hexane/ethyl acetate = 4/1 (v/v)], the title compound was obtained as a pale brown solid (155 mg, 82%). R$_f$ = 0.5 [*n*-hexane/ethyl acetate = 2/1 (v/v)]; $^1$H NMR (400 MHz, $CDCl_3$) δ 7.85 (s, 1H), 7.59 (dd, *J* = 8.3, 1.5 Hz, 1H), 7.49 (d, *J* = 7.9 Hz, 1H), 7.10 (t, *J* = 8.1 Hz, 1H), 4.90–4.83 (m, 1H), 4.36–4.28 (m, 2H), 4.09 (t, *J* = 8.9 Hz, 1H), 3.87 (dd, *J* = 8.9, 6.5 Hz, 1H), 1.48 (s, 9H); $^{13}$C{$^1$H} NMR (101 MHz, $CDCl_3$) δ 153.8, 152.9, 139.1, 133.2, 130.5, 126.8, 117.4, 94.3, 83.4, 69.9, 65.9, 46.9, 27.6; HRMS (EI): calculated for $C_{15}H_{18}INO_5$ ($M^+$), 419.0230, found 419.0235; m.p. = 138–140 °C.

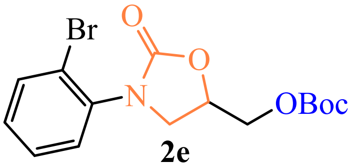

2e

**(3-(2-Bromophenyl)-2-oxooxazolidin-5-yl)methyl *tert*-butyl carbonate (2e).** The preparation was carried out according on **Typical Procedure C**. Starting from **1e** (114 mg, 0.5 mmol), after purified by column chromatography [silica gel, *n*-hexane/ethyl acetate = 4/1 (v/v)], the title compound was obtained as a yellow solid (169 mg, 91%). $R_f$ =0.2 [*n*-hexane/ethyl acetate = 3/1 (v/v)]; $^1$H NMR (400 MHz, $CDCl_3$) δ 7.65 (dd, $J$ = 8.0, 1.1 Hz, 1H), 7.43–7.36 (m, 2H), 7.26–7.22 (m, 1H), 4.96–4.90 (m, 1H), 4.36 (ddd, $J$ = 18.9, 11.8, 4.8 Hz, 2H), 4.12 (t, $J$ = 8.8 Hz, 1H), 3.81 (dd, $J$ = 8.8, 5.7 Hz, 1H), 1.50 (s, 9H); $^{13}C\{^1H\}$ NMR (101 MHz, $CDCl_3$) δ 155.6, 152.9, 136.0, 133.7, 130.0, 129.7, 128.7, 122.5, 83.0, 71.0, 66.2, 48.8, 27.6; HRMS (EI): calculated for $C_{15}H_{18}BrNO_5$ ($M^+$), 371.0368, found 371.0360; m.p. = 83–84 °C.

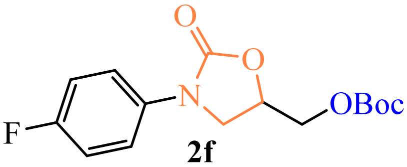

2f

***tert*-Butyl ((3-(4-fluorophenyl)-2-oxooxazolidin-5-yl)methyl) carbonate (2f).** The preparation was carried out according on **Typical Procedure C**. Starting from **1f** (84 mg, 0.5 mmol), after purified by column chromatography [silica gel, *n*-hexane/ethyl acetate = 4/1 (v/v)], the title compound was obtained as a white solid (84 mg, 54%). $R_f$ = 0.3 [*n*-hexane/ethyl acetate = 4/1 (v/v)]; $^1$H NMR (400 MHz, $CDCl_3$) δ 7.52–7.47 (m, 2H), 7.11–7.05 (m, 2H), 4.90–4.83 (m, 1H), 4.36–4.29 (m, 2H), 4.11 (t, $J$ = 8.9 Hz, 1H), 3.88 (dd, $J$ = 8.9, 6.4 Hz, 1H), 1.48 (s, 9H); $^{13}C\{^1H\}$ NMR (101 MHz, $CDCl_3$) δ 159.4 (d, $J_{C\text{-}F}$ = 244.2 Hz), 154.2, 152.9, 134.1 (d, $J_{C\text{-}F}$ = 3.0Hz), 120.1 (d, $J_{C\text{-}F}$ = 7.7 Hz) 115.8 (d, $J_{C\text{-}F}$ = 22.6 Hz), 83.3, 69.8, 66.0, 47.4, 27.6; $^{19}$F NMR (376 MHz, $CDCl_3$) δ –118.0; HRMS (EI): calculated for $C_{15}H_{18}FNO_5$ ($M^+$), 311.1169, found 311.1160; m.p.= 121–122 °C.

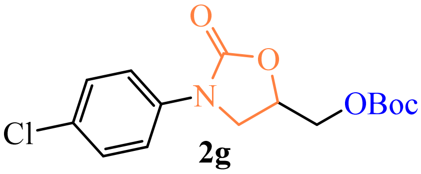

2g

***tert*-Butyl ((3-(4-chlorophenyl)-2-oxooxazolidin-5-yl)methyl) carbonate (2g).** The preparation was carried out according on **Typical Procedure C**. Starting from **1g** (92 mg, 0.5 mmol), after purified by column chromatography [silica gel, *n*-hexane/ethyl acetate = 4/1 (v/v)], the title compound was obtained as a pale yellow solid (116 mg, 71%). $R_f$ = 0.3 [*n*-hexane/ethyl acetate = 4/1 (v/v)]; $^1$H NMR (400 MHz, $CDCl_3$) δ 7.51–7.47 (m, 2H), 7.36–7.32 (m, 2H), 4.90–4.84 (m, 1H), 4.36–4.28 (m, 2H), 4.10 (t, $J$ = 8.9 Hz,

1H), 3.87 (dd, $J$ = 8.9, 6.4 Hz, 1H), 1.48 (s, 9H); $^{13}C\{^1H\}$ NMR (101 MHz, $CDCl_3$) δ 153.9, 152.9, 136.6, 129.5, 129.1, 119.4, 83.3, 69.8, 65.9, 47.0, 27.6; HRMS (EI): calculated for $C_{15}H_{18}ClNO_5$ ($M^+$), 327.0874, found 327.0870; m.p. = 134–136 °C.

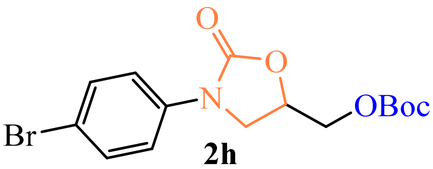


**2h**

**(3-(4-Bromophenyl)-2-oxooxazolidin-5-yl)methyl *tert*-butyl carbonate (2h).** The preparation was carried out according on **Typical Procedure C**. Starting from **1h** (114 mg, 0.5 mmol), after purified by column chromatography [silica gel, *n*-hexane/ethyl acetate = 4/1 (v/v)], the title compound was obtained as a yellow solid (130 mg, 70%). $R_f$ = 0.5 [*n*-hexane/ethyl acetate = 2/1 (v/v)]; $^1H$ NMR (400 MHz, $CDCl_3$) δ 7.49 (d, $J$ = 9.2 Hz, 2H), 7.44 (d, $J$ = 9.2 Hz, 2H), 4.90–4.84 (m, 1H), 4.36–4.28 (m, 2H), 4.10 (t, $J$ = 8.9 Hz, 1H), 3.87 (dd, $J$ = 8.9, 6.5 Hz, 1H), 1.48 (s, 9H); $^{13}C\{^1H\}$ NMR (101 MHz, $CDCl_3$) δ 153.9, 152.9, 137.1, 132.1, 119.7, 117.1, 83.4, 69.8, 65.9, 46.9, 27.6; HRMS (EI): calculated for $C_{15}H_{18}BrNO_5$ ($M^+$), 371.0368, found 371.0370; m.p. = 142–143 °C.

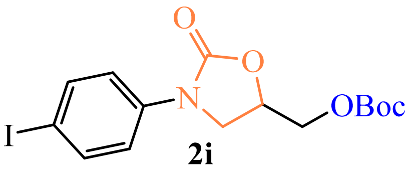


**2i**

***tert*-Butyl ((3-(4-iodophenyl)-2-oxooxazolidin-5-yl)methyl) carbonate (2i).** The preparation was carried out according on **Typical Procedure C**. Starting from **1i** (138 mg, 0.5 mmol), after purified by column chromatography [silica gel, *n*-hexane/ethyl acetate = 4/1 (v/v)], the title compound was obtained as a brown solid (184 mg, 88%). $R_f$ = 0.4 [*n*-hexane/ethyl acetate = 2/1 (v/v)]; $^1H$ NMR (400 MHz, $CDCl_3$) δ 7.70–7.66 (m, 2H), 7.34–7.30 (m, 2H), 4.90–4.83 (m, 1H), 4.36–4.28 (m, 2H), 4.09 (t, $J$ = 9.0 Hz, 1H), 3.86 (dd, $J$ = 8.9, 6.4 Hz, 1H), 1.48 (s, 9H); $^{13}C\{^1H\}$ NMR (101 MHz, $CDCl_3$) δ 153.8, 152.9, 138.0, 137.8, 120.0, 87.7, 83.4, 69.8, 65.9, 46.8, 27.6; HRMS (EI): calculated for $C_{15}H_{18}INO_5$ ($M^+$), 419.0230, found 419.0237; m.p. = 142–143 °C.

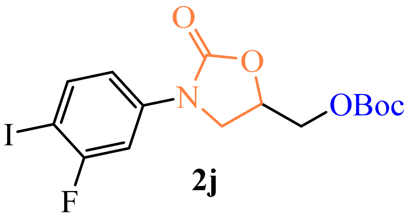


**2j**

***tert*-Butyl ((3-(3-fluoro-4-iodophenyl)-2-oxooxazolidin-5-yl)methyl) carbonate (2j).**[S 11] The

preparation was carried out according on **Typical Procedure C**. Starting from **1j** (147 mg, 0.5 mmol), after purified by column chromatography [silica gel, *n*-hexane/ethyl acetate = 4/1 (v/v)], the title compound was obtained as a white solid (177 mg, 81%). $R_f$ = 0.6 [*n*-hexane/ethyl acetate = 4/1 (v/v)]; $^1$H NMR (400 MHz, $CDCl_3$) δ 7.71 (dd, *J* = 8.7, 7.2 Hz, 1H), 7.47 (dd, *J* = 10.2, 2.5 Hz, 1H), 7.06 (dd, *J* = 8.7, 2.6 Hz, 1H), 4.91–4.85 (m, 1H), 4.32 (ddd, *J* = 17.9, 12.0, 4.6 Hz, 2H), 4.09 (t, *J* = 9.0 Hz, 1H), 3.87 (dd, *J* = 9.0, 6.4 Hz, 1H), 1.48 (s, 9H); $^{13}$C{$^1$H} NMR (101 MHz, $CDCl_3$) δ 161.9 (d, $J_{C\text{-}F}$ = 244.2 Hz), 153.6, 152.9, 139.8 (d, $J_{C\text{-}F}$ = 10.1 Hz), 139.3 (d, $J_{C\text{-}F}$ = 2.9 Hz), 115.0 (d, $J_{C\text{-}F}$ = 3.4 Hz), 106.0 (d, $J_{C\text{-}F}$ = 29.4 Hz), 83.4, 74.3 (d, $J_{C\text{-}F}$ = 26.0 Hz), 69.9, 65.8, 46.8, 27.6; HRMS (EI): calculated for $C_{15}H_{17}FINO_5$ ($M^+$), 437.0135, found 437.0130; m.p. = 124–125 °C.

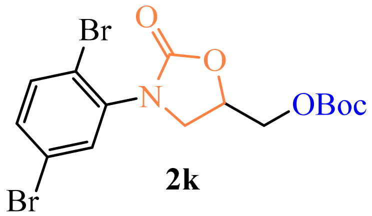

**2k**

***tert*-Butyl ((3-(2,5-dibromophenyl)-2-oxooxazolidin-5-yl)methyl) carbonate (2k).** The preparation was carried out according on **Typical Procedure C**. Starting from **1k** (153 mg, 0.5 mmol), after purified by column chromatography [silica gel, *n*-hexane/ethyl acetate = 4/1 (v/v)], the title compound was obtained as a colorless liquid (183 mg, 81%). $R_f$ = 0.3 [*n*-hexane/ethyl acetate = 4/1 (v/v)]; $^1$H NMR (400 MHz, $CDCl_3$) δ 7.58 (d, *J* = 2.3 Hz, 1H), 7.51 (d, *J* = 8.6 Hz, 1H), 7.37 (dd, *J* = 8.6, 2.4 Hz, 1H), 4.96–4.90 (m, 1H), 4.40 (dd, *J* = 11.9, 4.3 Hz, 1H), 4.31 (dd, *J* = 11.9, 4.6 Hz, 1H), 4.14 (t, *J* = 8.8 Hz, 1H), 3.80 (dd, *J* = 8.7, 5.5 Hz, 1H), 1.51 (s, 9H); $^{13}$C{$^1$H} NMR (101 MHz, $CDCl_3$) δ 155.2, 152.8, 137.2, 134.6, 132.9, 132.7, 121.4, 121.2, 83.0, 71.2, 66.1, 48.4, 27.5; HRMS (EI): calculated for $C_{15}H_{17}Br_2NO_5$ ($M^+$), 448.9473, found 448.9470.

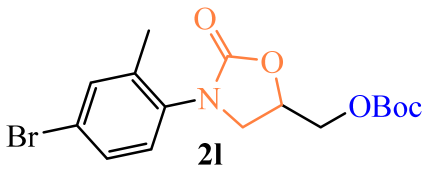

**2l**

**(3-(4-Bromo-2-methylphenyl)-2-oxooxazolidin-5-yl)methyl *tert*-butyl carbonate (2l).** The preparation was carried out according on **Typical Procedure C**. Starting from **1l** (121 mg, 0.5 mmol), after purified by column chromatography [silica gel, *n*-hexane/ethyl acetate = 4/1 (v/v)], the title compound was obtained as a colorless liquid (133 mg, 69%). $R_f$ = 0.3 [*n*-hexane/ethyl acetate = 4/1 (v/v)]; $^1$H NMR (400 MHz, $CDCl_3$) δ 7.44 (d, *J* = 1.7 Hz, 1H), 7.38–7.35 (m, 1H), 7.12 (d, *J* = 8.4 Hz, 1H), 4.89 (ddd, *J* = 13.1, 5.5, 4.1 Hz, 1H), 4.41 (dd, *J* = 11.9, 4.1 Hz, 1H), 4.26 (dd, *J* = 11.9, 4.1 Hz, 1H), 4.03 (t, *J* = 8.9 Hz, 1H), 3.75 (dd, *J* = 9.0, 5.5 Hz, 1H), 2.30 (s, 3H), 1.49 (s, 9H); $^{13}$C{$^1$H} NMR (101 MHz, $CDCl_3$) δ 155.3, 152.9, 138.4, 134.8,

134.2, 130.0, 128.1, 121.8, 83.0, 70.7, 66.3, 49.1, 27.6, 17.6; HRMS (EI): calculated for $C_{16}H_{20}BrNO_5$ ($M^+$), 385.0525, found 385.0520.

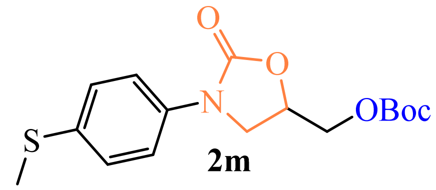

**2m**

***tert*-Butyl ((3-(4-(methylthio)phenyl)-2-oxooxazolidin-5-yl)methyl) carbonate (2m).** The preparation was carried out according on **Typical Procedure C**. Starting from **1m** (98 mg, 0.5 mmol), after purified by column chromatography [silica gel, *n*-hexane/ethyl acetate = 4/1 (v/v)], the title compound was obtained as a pale orange solid (119 mg, 70%). $R_f$ = 0.3 [*n*-hexane/ethyl acetate = 4/1 (v/v)]; $^1$H NMR (400 MHz, $CDCl_3$) δ 7.48–7.46 (m, 2H), 7.30–7.26 (m, 3H), 4.89–4.83 (m, 1H), 4.32 (dd, *J* = 16.7, 12.8 Hz, 2H), 4.10 (t, *J* = 8.9 Hz, 1H), 3.87 (dd, *J* = 8.9, 6.4 Hz, 1H), 2.48 (s, 3H), 1.48 (s, 9H); $^{13}C\{^1H\}$ NMR (101 MHz, $CDCl_3$) δ 154.0, 152.9, 135.5, 133.9, 127.9, 118.9, 83.3, 69.8, 66.0, 47.1, 27.6, 16.5; HRMS (EI): calculated for $C_{16}H_{21}NO_5S$ ($M^+$), 339.1140, found 339.1147; m.p. = 103–104 °C.

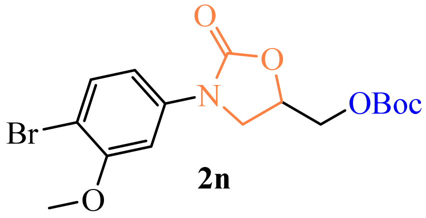

**2n**

**(3-(4-Bromo-3-methoxyphenyl)-2-oxooxazolidin-5-yl)methyl *tert*-butyl carbonate (2n).** The preparation was carried out according on **Typical Procedure C**. Starting from **1n** (129 mg, 0.5 mmol), after purified by column chromatography [silica gel, *n*-hexane/ethyl acetate = 4/1 (v/v)], the title compound was obtained as an off-white solid (149 mg, 74%). $R_f$ = 0.3 [*n*-hexane/ethyl acetate = 2/1 (v/v)]; $^1$H NMR (400 MHz, $CDCl_3$) δ 7.60 (d, *J* = 2.4 Hz, 1H), 7.49 (d, *J* = 8.7 Hz, 1H), 6.66 (dd, *J* = 8.6, 2.5 Hz, 1H), 4.90–4.84 (m, 1H), 4.32 (d, *J* = 4.9 Hz, 2H), 4.11 (t, *J* = 8.9 Hz, 1H), 3.92–3.86 (m, 5H), 1.48 (s, 9H); $^{13}C\{^1H\}$ NMR (101 MHz, $CDCl_3$) δ 156.3, 153.9, 152.9, 138.6, 133.0, 110.5, 106.5, 102.9, 83.4, 69.8, 65.9, 56.3, 47.1, 27.6; HRMS (EI): calculated for $C_{16}H_{20}BrNO_6$ ($M^+$), 401.0474, found 401.0470; m.p. = 152–153 °C.

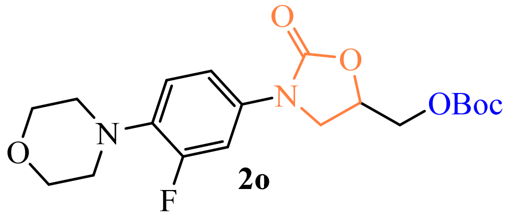

**2o**

***tert*-Butyl ((3-(3-fluoro-4-morpholinophenyl)-2-oxooxazolidin-5-yl)methyl) carbonate (2o).** The

preparation was carried out according on **Typical Procedure C**. Starting from **1o** (126 mg, 0.5 mmol), after purified by column chromatography [silica gel, *n*-hexane/ethyl acetate = 4/1 (v/v)], the title compound was obtained as an off-white solid (153 mg, 77%). $R_f$ = 0.2 [*n*-hexane/ethyl acetate = 4/1 (v/v)]; $^1$H NMR (400 MHz, $CDCl_3$) δ 7.44 (dd, *J* = 14.3, 2.5 Hz, 1H), 7.12 (dd, *J* = 8.8, 1.6 Hz, 1H), 6.93 (t, *J* = 9.1 Hz, 1H), 4.89–4.82 (m, 1H), 4.35–4.28 (m, 2H), 4.07 (t, *J* = 8.9 Hz, 1H), 3.88–3.83 (m, 5H), 3.06 (t, *J* = 4.7 Hz, 4H), 1.48 (s, 9H); $^{13}$C{$^1$H} NMR (101 MHz, $CDCl_3$) δ 155.5 (d, $J_{C\text{-}F}$ = 246.1 Hz),154.0, 152.9, 136.5 (d, $J_{C\text{-}F}$ = 9.2 Hz), 133.0 (d, $J_{C\text{-}F}$ = 10.6 Hz), 118.8 (d, $J_{C\text{-}F}$ = 4.3 Hz), 113.9 (d, $J_{C\text{-}F}$ = 3.4 Hz), 107.5 (d, $J_{C\text{-}F}$ = 26.5 Hz), 83.2, 69.8, 66.9, 65.9, 51.0, 47.1, 27.6; $^{19}$F NMR (376 MHz, $CDCl_3$) δ –120.1; HRMS (EI): calculated for $C_{19}H_{25}FN_2O_6$ ($M^+$), 396.1697, found 396.1690. m.p. = 167–169 °C.

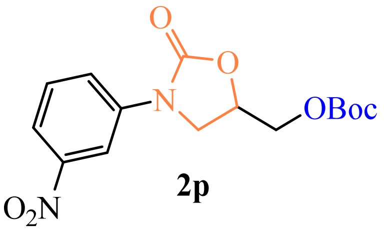


**2p**

***tert*-Butyl ((3-(3-nitrophenyl)-2-oxooxazolidin-5-yl)methyl) carbonate (2p).** The preparation was carried out according on **Typical Procedure C**. Starting from **1p** (97 mg, 0.5 mmol), after purified by column chromatography [silica gel, *n*-hexane/ethyl acetate = 4/1 (v/v)], the title compound was obtained as a yellow liquid (140 mg, 83%). $R_f$ = 0.1 [*n*-hexane/ethyl acetate = 3/1 (v/v)]; $^1$H NMR (400 MHz, $CDCl_3$) δ 8.27 (t, *J* = 2.1 Hz, 1H), 8.07 (ddd, *J* = 8.4, 2.3, 0.8 Hz, 1H), 7.98 (ddd, *J* = 8.2, 2.1, 0.8 Hz, 1H), 7.56 (t, *J* = 8.3 Hz, 1H), 5.00–4.94 (m, 1H), 4.41 (dd, *J* = 12.1, 4.3 Hz, 1H), 4.34 (dd, J = 12.1, 4.7 Hz, 1H), 4.23 (t, *J* = 8.9 Hz, 1H), 4.00 (dd, *J* = 8.9, 6.4 Hz, 1H), 1.48 (s, 9H); $^{13}$C{$^1$H} NMR (101 MHz, $CDCl_3$) δ 153.8, 152.8, 148.5, 139.1, 129.9, 123.7, 118.5, 112.3, 83.3, 70.2, 65.7, 46.6, 27.5; HRMS (EI): calculated for $C_{15}H_{18}N_2O_7$ ($M^+$), 338.1114, found 338.1120.

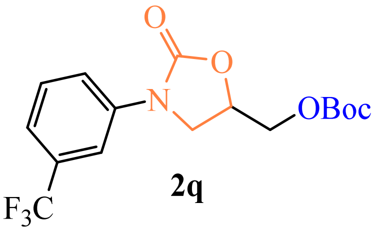


**2q**

***tert*-Butyl ((2-oxo-3-(3-(trifluoromethyl)phenyl)oxazolidin-5-yl)methyl) carbonate (2q).** The preparation was carried out according on **Typical Procedure C**. Starting from **1q** (109 mg, 0.5 mmol), after purified by column chromatography [silica gel, *n*-hexane/ethyl acetate = 4/1 (v/v)], the title compound was obtained as a pale yellow solid (134 mg, 74%). $R_f$ = 0.4 [*n*-hexane/ethyl acetate = 2/1 (v/v)]; $^1$H NMR (400 MHz, $CDCl_3$) δ 7.78–7.74 (m, 2H), 7.47 (t, *J* = 8.0 Hz, 1H), 7.37 (d, *J* = 7.8 Hz, 1H), 4.91–4.85 (m, 1H), 4.34 (dd, *J* = 12.1, 4.4 Hz, 1H), 4.29 (dd, *J* = 12.0, 4.7 Hz, 1H), 4.15 (t, *J* = 8.9 Hz, 1H), 3.92 (dd, *J* = 8.9,

6.3 Hz, 1H), 1.45 (s, 9H); $^{13}C\{^1H\}$ NMR (101 MHz, $CDCl_3$) δ 153.9, 152.8, 138.5, 131.4 (q, $J_{C\text{-}F}$ = 32.6 Hz), 129.6, 123.7 (q, $J_{C\text{-}F}$ = 272.5 Hz), 121.1, 120.6 (d, $J_{C\text{-}F}$ = 3.4 Hz), 114.6 (d, $J_{C\text{-}F}$ = 3.9 Hz), 83.2, 70.0, 65.8, 46.7, 27.5; $^{19}F$ NMR (376 MHz, $CDCl_3$) δ –62.6; HRMS (ESI-TOF): calculated for $C_{16}H_{18}F_3NO_5$+$Na^+$ [M+$Na^+$] 384.1029, found 384.1035; m.p. = 55–56 °C.

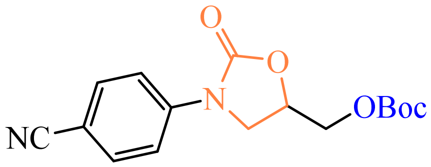


**2r**

***tert*-Butyl ((3-(4-cyanophenyl)-2-oxooxazolidin-5-yl)methyl) carbonate (2r).** The preparation was carried out according on **Typical Procedure C**. Starting from **1r** (87 mg, 0.5 mmol), after purified by column chromatography [silica gel, *n*-hexane/ethyl acetate = 1/1 (v/v)], the title compound was obtained as a yellow solid (108 mg, 68%). $R_f$ = 0.2 [*n*-hexane/ethyl acetate = 2/1 (v/v)]; $^1H$ NMR (400 MHz, $CDCl_3$) δ 7.68–7.62 (m, 4H), 4.93–4.87 (m, 1H), 4.35 (dd, $J$ = 12.1, 4.3 Hz, 1H), 4.29 (dd, $J$ = 12.1, 4.4 Hz, 1H), 4.14 (t, $J$ = 8.9 Hz, 1H), 3.92 (dd, $J$ = 8.8, 6.5 Hz, 1H), 1.45 (s, 9H); $^{13}C\{^1H\}$ NMR (101 MHz, $CDCl_3$) δ 153.5, 152.8, 141.7, 133.1, 118.5, 117.8, 107.0, 83.4, 70.1, 65.7, 46.5, 27.5; HRMS (ESI-TOF): calculated for $C_{16}H_{18}N_2O_5$+$NH_4^+$ [M+$NH_4^+$] 336.1554, found 336.1559; m.p. = 84–85 °C.

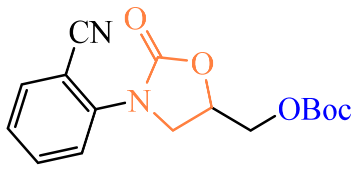


**2s**

***tert*-Butyl ((3-(2-cyanophenyl)-2-oxooxazolidin-5-yl)methyl) carbonate (2s).** The preparation was carried out according on **Typical Procedure C**. Starting from **1s** (87 mg, 0.5 mmol), after purified by column chromatography [silica gel, *n*-hexane/ethyl acetate = 1/1 (v/v)], the title compound was obtained as a pale white solid (91 mg, 57%). $R_f$ = 0.2 [*n*-hexane/ethyl acetate = 2/1 (v/v)]; $^1H$ NMR (400 MHz, $CDCl_3$) δ 7.71 (dd, $J$ = 7.8, 1.1 Hz, 1H), 7.69–7.65 (m, 1H), 7.61 (dd, $J$ = 8.3, 0.9 Hz, 1H), 7.40 (td, $J$ = 7.5, 1.3 Hz, 1H), 4.99–4.93 (m, 1H), 4.42–4.31 (m, 3H), 3.98 (dd, $J$ = 8.9, 6.1 Hz, 1H), 1.49 (s, 9H); $^{13}C\{^1H\}$ NMR (101 MHz, $CDCl_3$) δ 155.1, 152.9, 140.4, 133.9, 133.9, 127.5, 126.8, 116.5, 109.6, 83.3, 71.2, 65.9, 48.7, 27.6; HRMS (ESI-TOF): calculated for $C_{16}H_{18}N_2O_5$+$NH_4^+$ [M+$NH_4^+$] 336.1554, found 336.1560; m.p. = 96–97 °C.

**Reactions designed to demonstrate that *tert*-butyl carbonate functions as a nucleophile**

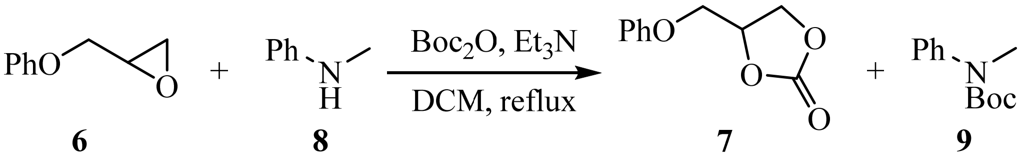

**4-(Phenoxymethyl)-1,3-dioxolan-2-one (7)**[S12] **and *tert*-butyl methyl(phenyl)carbamate (9).**[S13] To a solution of 2-(phenoxymethyl)oxirane (**6**, 38 mg, 0.25 mmol) and *N*-methylaniline (**8**, 27 mg, 0.25 mmol) in DCM (1 mL), $Boc_2O$ (126 µL, 0.55 mmol, 1.1 equiv) and triethylamine (209 µL, 1.5 mmol, 3 equiv) were added. The reaction mixture was stirred under reflux for 14 hours. Upon completion, as confirmed by TLC analysis, the mixture was concentrated under reduced pressure and purified by column chromatography (silica gel, *n*-hexane/ethyl acetate = 4:1, v/v) to afford the title compounds **7** (51%) and **9** (quantitative yield).

Compounds **7**. Colorless liquid; $R_f$ = 0.2 [*n*-hexane/ethyl acetate = 2/1 (v/v)]; $^1$H NMR (400 MHz, $CDCl_3$) δ 7.30 (t, *J* = 7.9 Hz, 3H), 7.00 (t, *J* = 7.3 Hz, 1H), 6.90 (d, *J* = 8.6 Hz, 2H), 5.04–4.99 (m, 1H), 4.59 (t, *J* = 8.5 Hz, 1H), 4.51 (dd, *J* = 8.6, 6.0 Hz, 1H), 4.22 (dd, *J* = 10.6, 3.9 Hz, 1H), 4.11 (dd, *J* = 10.7, 3.5 Hz, 1H); $^{13}C\{^1H\}$ NMR (101 MHz, $CDCl_3$) δ 157.6, 154.8, 129.6, 121.8, 114.4, 74.1, 66.7, 66.1; HRMS (EI): calculated for $C_{10}H_{10}O_4$ ($M^+$), 194.0579, found 194.0570.

Compounds **9**. Brown liquid; $R_f$ = 0.6 [*n*-hexane/ethyl acetate = 2/1 (v/v)]; $^1$H NMR (400 MHz, $CDCl_3$) δ 7.34–7.31 (m, 2H), 7.26–7.22 (m, 2H), 7.16 (t, *J* = 7.3 Hz, 1H), 3.26 (s, 3H), 1.45 (s, 9H); $^{13}C\{^1H\}$ NMR (101 MHz, $CDCl_3$) δ 154.8, 143.8, 128.5, 125.5, 125.3, 80.2, 37.3, 28.3; HRMS (EI): calculated for $C_{12}H_{17}NO_2$ ($M^+$), 207.1259, found 207.1255.

**Synthetic Applications**

**Gram scale preparation of Toloxatone**

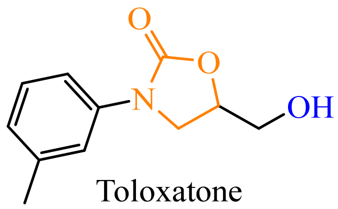

Toloxatone

**Toloxatone.**[S10,S14] The preparation was carried out according on **Typical Procedure C**. Starting from **1b** (1.63 g, 10 mmol), after purified by column chromatography [silica gel, *n*-hexane/ethyl acetate = 4/1 (v/v)], the oxazolidinone (8.2 mmol, 82%) were obtained. To a 250 mL round bottom flask were added the oxazolidinone (8.2 mmol) and TFA (21 mL) in DCM (8.2 mL). After stirring at room temperature for 4 h, the volatiles were removed under reduced pressure. The residue was purified by column chromatography [silica gel, *n*-hexane/ethyl acetate = 1/1 (v/v)] to afford Toloxatone as a white solid (1.67 g, 98%). $R_f$ = 0.1 [*n*-hexane/ethyl acetate = 1/1 (v/v)]; $^1$H NMR (400 MHz, acetone-$d_6$) δ 7.44 (d, *J* = 8.6 Hz, 2H), 7.23 (t, *J*

= 7.8 Hz, 1H), 6.91 (d, $J$ = 7.5 Hz, 1H), 4.76–4.70 (m, 1H), 4.33–4.32 (m, 1H), 4.12 (t, $J$ = 8.9 Hz, 1H), 3.95 (dd, $J$ = 8.9, 6.4 Hz, 1H), 3.86 (ddd, $J$ = 12.2, 5.7, 3.6 Hz, 1H), 3.77–3.71 (m, 1H), 2.32 (s, 3H); $^{13}C\{^1H\}$ NMR (101 MHz, acetone-$d_6$) δ 155.3, 140.0, 139.3, 129.4, 124.7, 119.2, 115.8, 73.9, 63.2, 47.0, 21.6; HRMS (EI): calculated for $C_{11}H_{13}NO_3$ ($M^+$), 207.0895, found 207.0905; m.p. = 77–79 °C.

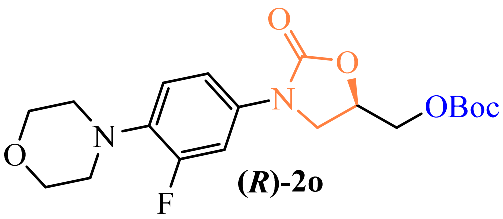


**(*R*)-2o**

**(*R*)-*tert*-Butyl ((3-(3-fluoro-4-morpholinophenyl)-2-oxooxazolidin-5-yl)methyl) carbonate ((*R*)-2o).** The preparation was carried out according on **Typical Procedure C**. Starting from (*R*)-**1o** (126 mg, 0.5 mmol), after purified by column chromatography [silica gel, *n*-hexane/ethyl acetate = 4/1 (v/v)], the title compound was obtained as an off-white solid (146 mg, 74%). $R_f$ = 0.2 [*n*-hexane/ethyl acetate = 4/1 (v/v)]; $[\alpha]_D^{22}$ +34.3 (*c* 0.6, $CH_2Cl_2$); $^1H$ NMR (400 MHz, $CDCl_3$) δ 7.44 (dd, $J$ = 14.4, 2.4 Hz, 1H), 7.14–7.11 (m, 1H), 6.93 (t, $J$ = 9.1 Hz, 1H), 4.89–4.83 (m, 1H), 4.35–4.27 (m, 2H), 4.10–4.05 (m, 1H), 3.88-3.83 (m, 5H), 3.07-3.06 (m, 4H), 1.49 (s, 9H); $^{13}C\{^1H\}$ NMR (101 MHz, $CDCl_3$) δ 155.6 (d, $J_{C–F}$ = 246.1 Hz), 154.1, 153.0, 136.6 (d, $J_{C–F}$ = 9.2 Hz), 133.1 (d, $J_{C–F}$ = 10.6 Hz), 118.9 (d, $J_{C–F}$ = 4.1 Hz), 114.0 (d, $J_{C–F}$ = 3.4 Hz), 107.6 (d, $J_{C–F}$ = 26.5 Hz), 83.3, 69.9, 67.0, 66.0, 51.1, 51.0, 47.2, 27.7; HRMS (EI): calculated for $C_{19}H_{25}FN_2O_6$ ($M^+$), 396.1697, found 396.1693. Enantiomeric excess value of (*R*)-**2o** was checked by HPLC analysis equipped with a chiral column. [column: CHIRALPAK® AD-H (4.6 mm × 250 mm); flow rate: 0.8 mL/min; eluent: 40% IPA in hexanes; UV detector: 254 nm (Hg lamp); $t_R$ (*R*)-**2o**: 11.44 min, (*S*)-**2o**: 10.04 min; ee 99%].

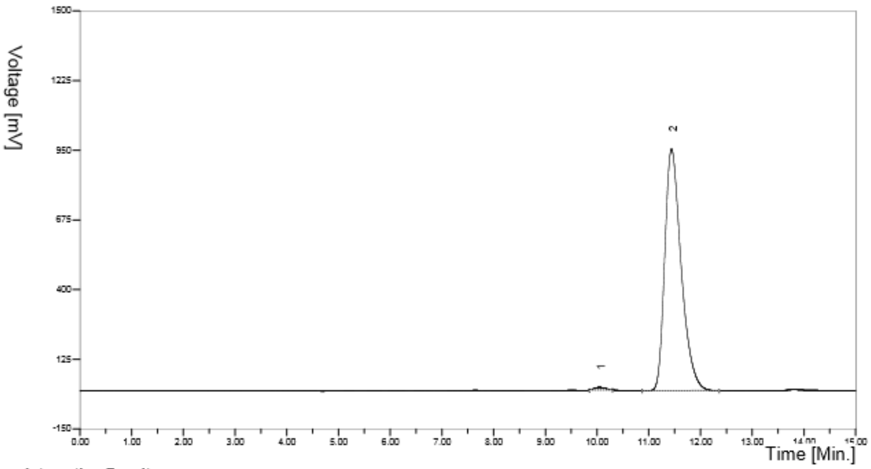


Integration Result

| # | Ret. Time(min) | Area(mv.sec) | Area Percentage(%) |
|---|---|---|---|
| 1 | 10.04 | 151.51 | 0.7158 |
| 2 | 11.44 | 21015.10 | 99.2842 |
| Total | | 21166.61 | 100 |

**Figure S1.** HPLC results for (*R*)-**2o**

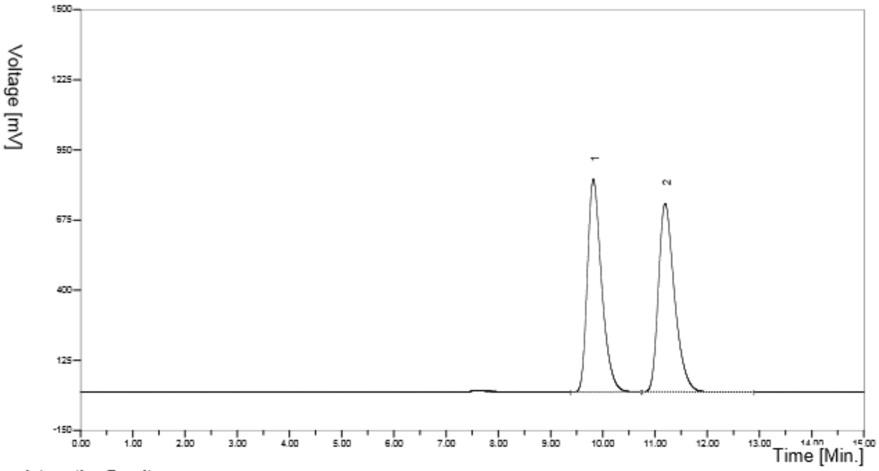


Integration Result

| # | Ret. Time(min) | Area(mv.sec) | Area Percentage(%) |
|---|---|---|---|
| 1 | 9.82 | 15783.13 | 49.9554 |
| 2 | 11.19 | 15811.31 | 50.0446 |
| Total | | 31594.44 | 100 |

**Figure S2.** HPLC results for (±)-**2o**

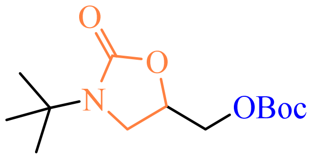


**2t**

***tert*-Butyl ((3-(*tert*-butyl)-2-oxooxazolidin-5-yl)methyl) carbonate (2t).** To a solution of **1t** (3 mmol) in DCM (6.0 mL) was added $Boc_2O$ (1.4 mL, 6 mmol, 2 equiv), triethylamine (836 µL, 6 mmol, 2 equiv) , and the mixture was stirred at reflux for 14 hours. Upon completion of the reaction, as determined by TLC analysis, the mixture was concentrated under reduced pressure, and purified by column chromatography [silica gel, *n*-hexane/ethyl acetate = 4/1 (v/v)] to afford compound **2t** as a colorless liquid (147 mg, 18%). $R_f$ = 0.2 [*n*-hexane/ethyl acetate = 2/1 (v/v)]; $^1$H NMR (400 MHz, $CDCl_3$) δ 4.59–4.53 (m, 1H), 4.20–4.11 (m, 2H), 3.67 (t, $J$ = 8.8 Hz, 1H), 3.38 (dd, $J$ = 8.9, 6.2 Hz, 1H), 1.45 (s, 9H), 1.36 (s, 9H); $^{13}$C{$^1$H} NMR (101 MHz, $CDCl_3$) δ 156.0, 153.0, 82.8, 69.0, 66.3, 53.4, 45.3, 27.6, 27.3; HRMS (ESI-TOF): calculated for $C_{13}H_{23}NO_5+Na^+$ [$M+Na^+$] 296.1468, found 296.1472.

**Table S2.** Epoxidation and Subsequent Oxazolidinone Formation for Selected Aliphatic Substrates

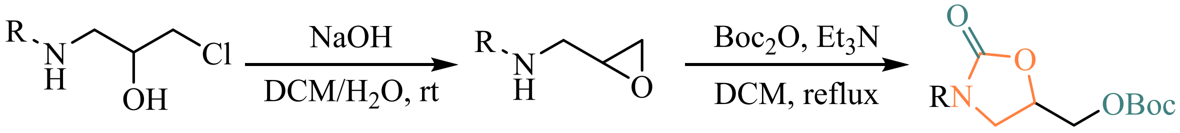


**S2** **S3** **S4**

| Entry | R | Epoxidation | Oxazolidinone | Comments |
|---|---|---|---|---|

| | | | | |
|---|---|---|---|---|
| | | (**S2** to **S3**) | formation (**S3** to **S4**) | |
| 1 | 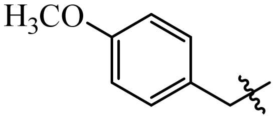 | Problematic | Not attempted | ● Since the key intermediate epoxide (S3) was not obtained cleanly or reliably, the team understandably did not proceed to ring closure to form the oxazolidinone (S4). ● This highlights that the epoxidation step is the bottleneck in these cases. |
| 2 | 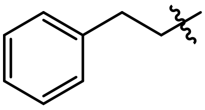 | Problematic | Not attempted | |
| 3 | 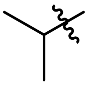 | Problematic | Not attempted | |
| 4 | 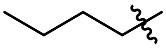 | Problematic | Not attempted | |
| 5 | 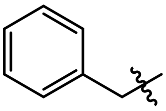 | | Problematic | |
| 6 | 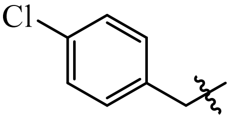 | | Problematic | |

## C. NMR Spectra for the synthesized compounds

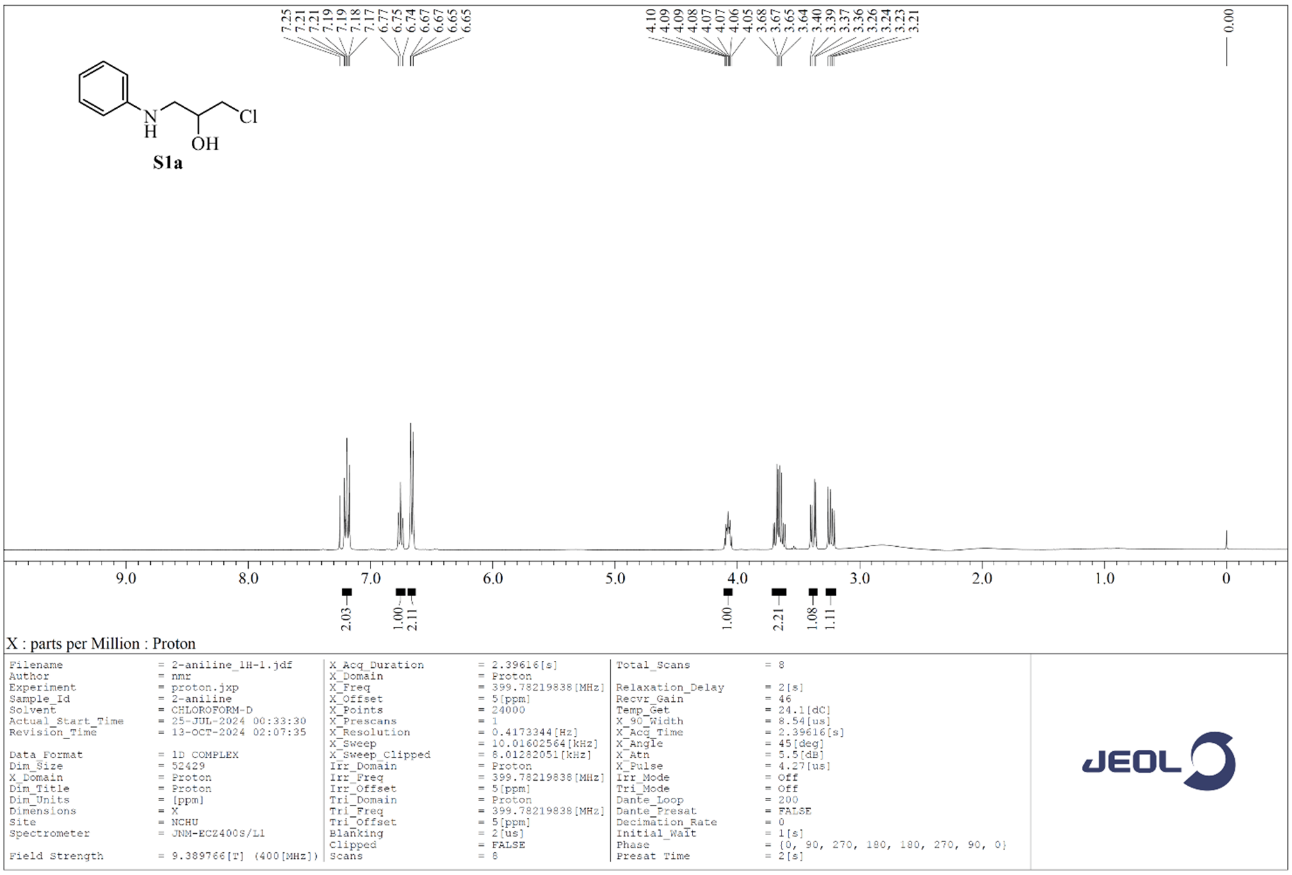


$^{1}H$ NMR spectrum of compound **S1a** (400 MHz, $CDCl_3$)

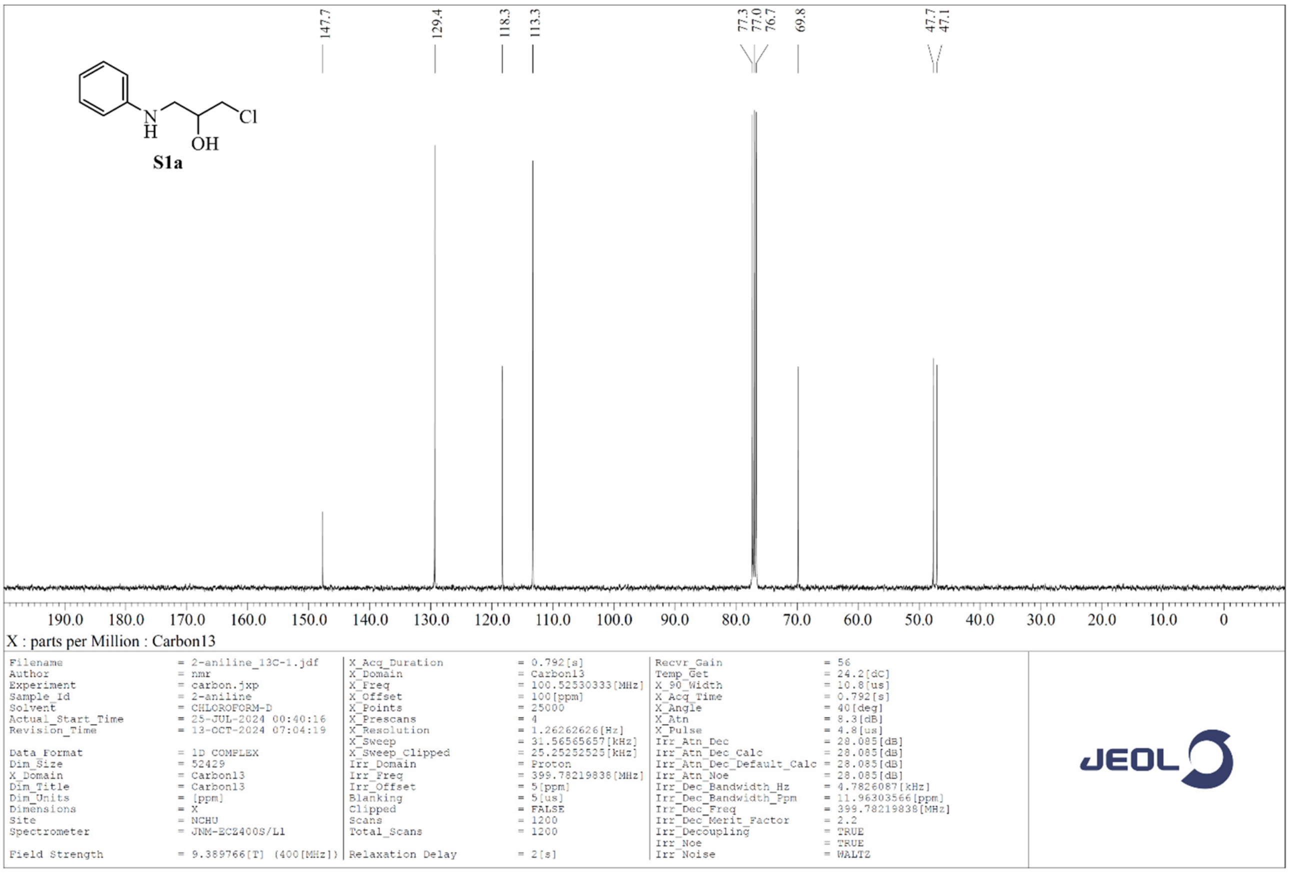


$^{13}C$ NMR spectrum of compound **S1a** (101 MHz, $CDCl_3$)

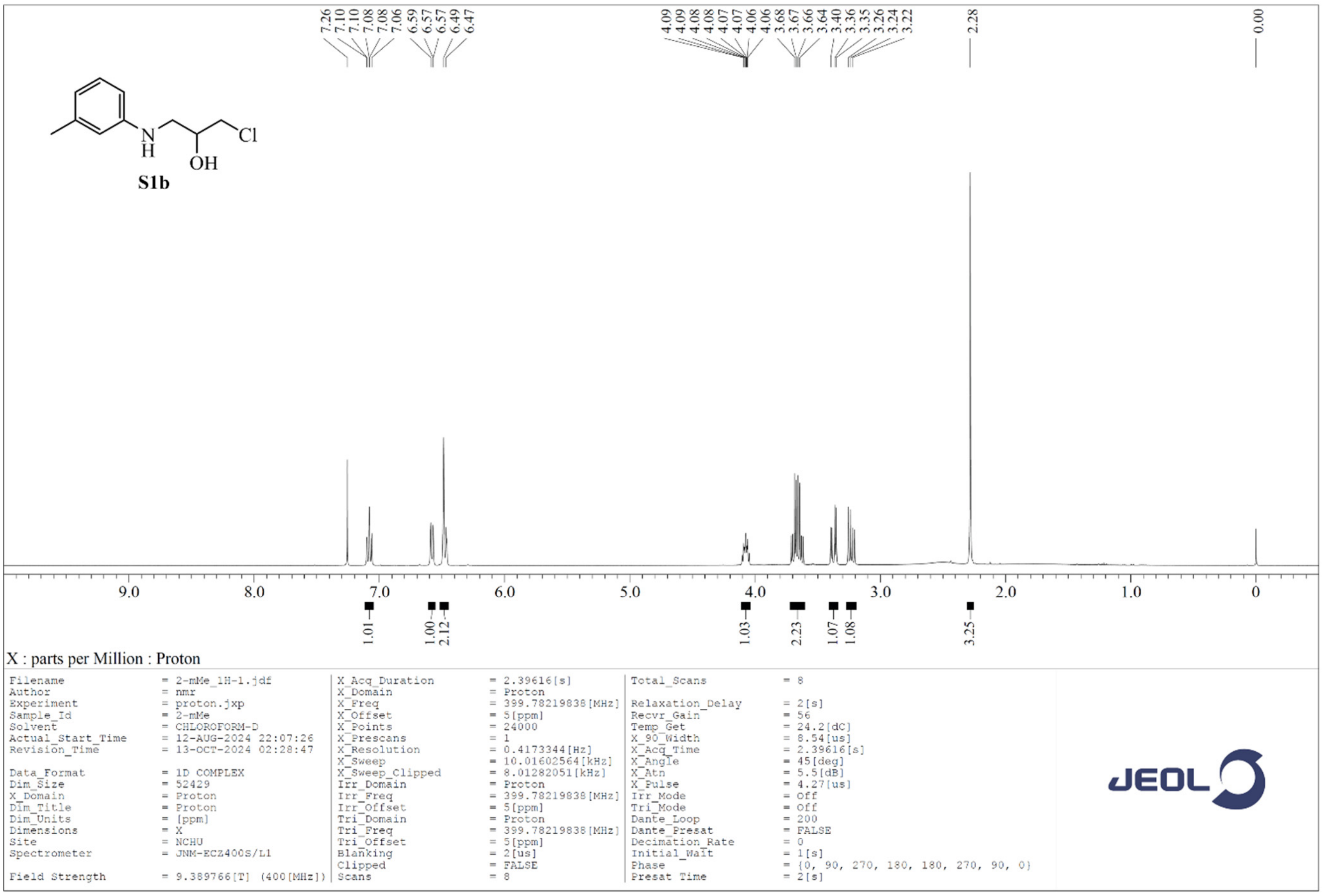


$^{1}H$ NMR spectrum of compound **S1b** (400 MHz, $CDCl_3$)

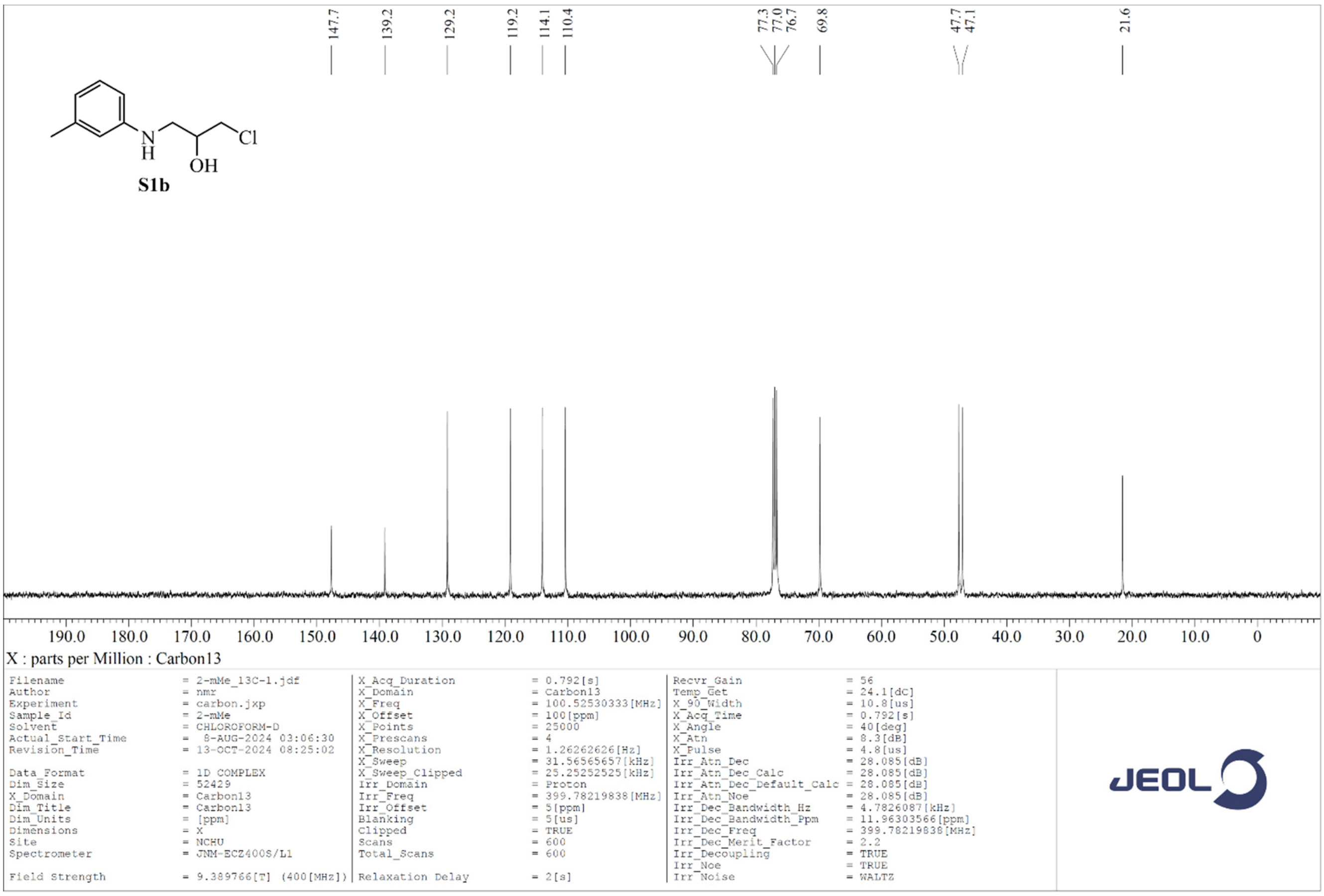


$^{13}C$ NMR spectrum of compound **S1b** (101 MHz, $CDCl_3$)

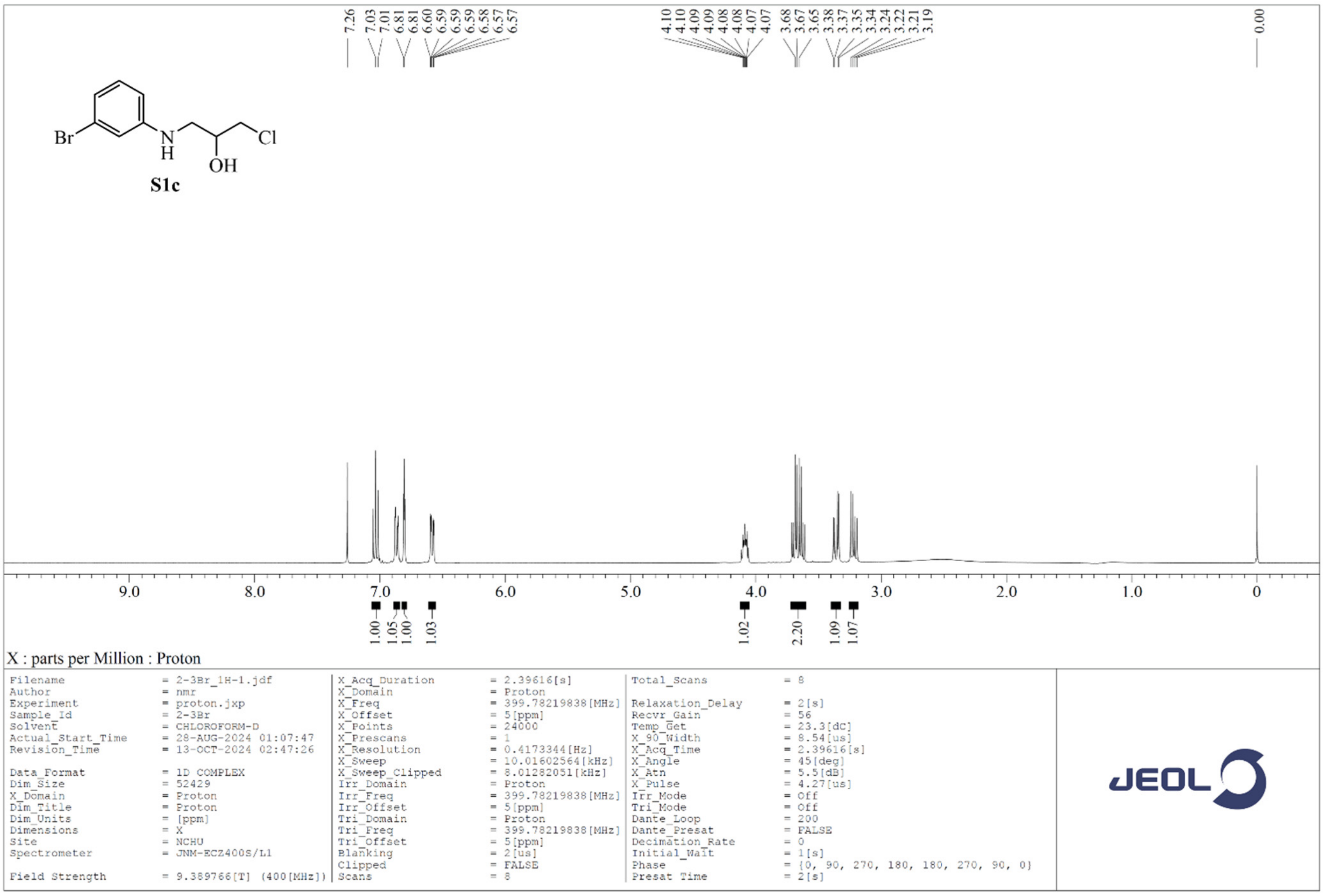


$^{1}H$ NMR spectrum of compound **S1c** (400 MHz, $CDCl_3$)

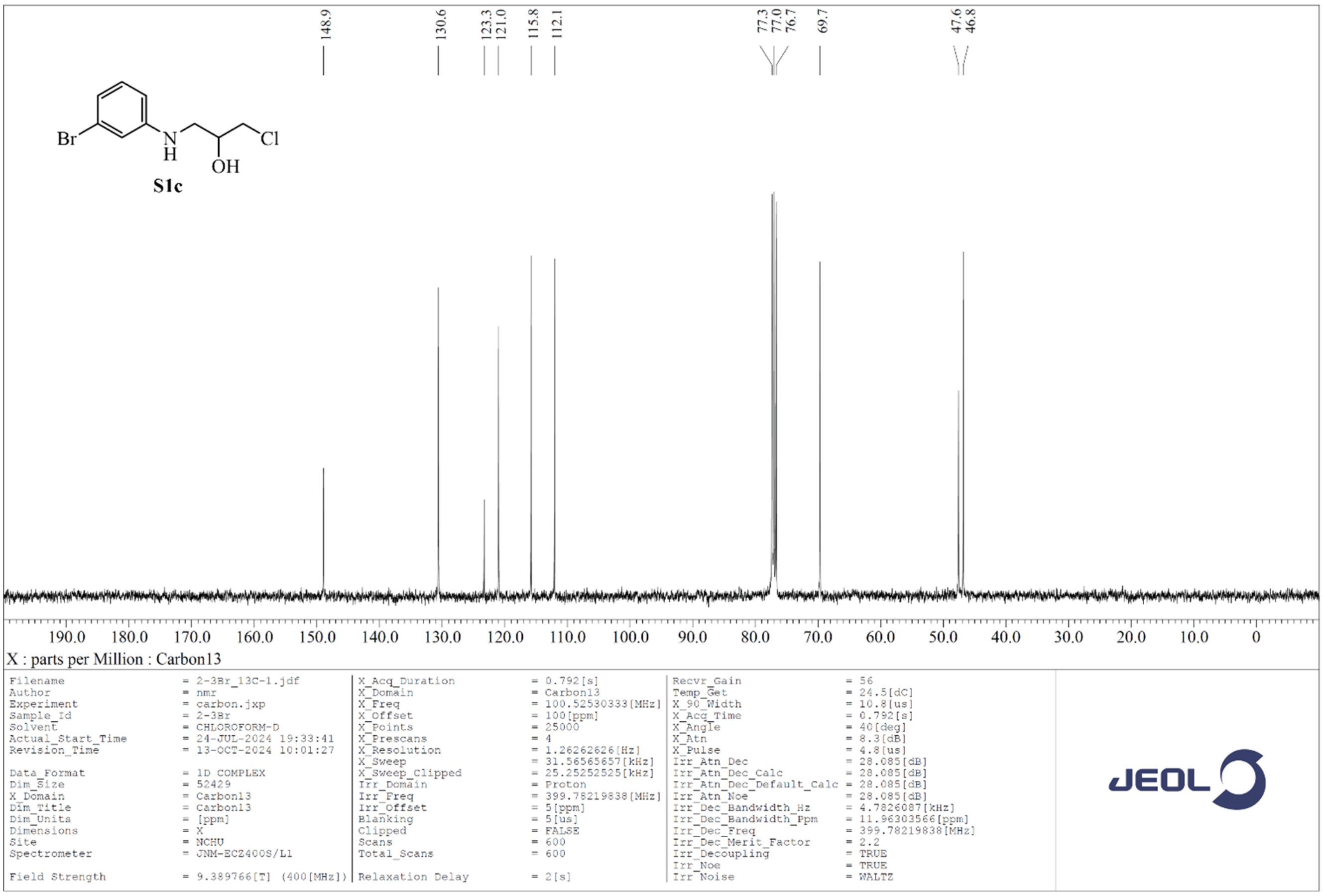


$^{13}C$ NMR spectrum of compound **S1c** (101 MHz, $CDCl_3$)

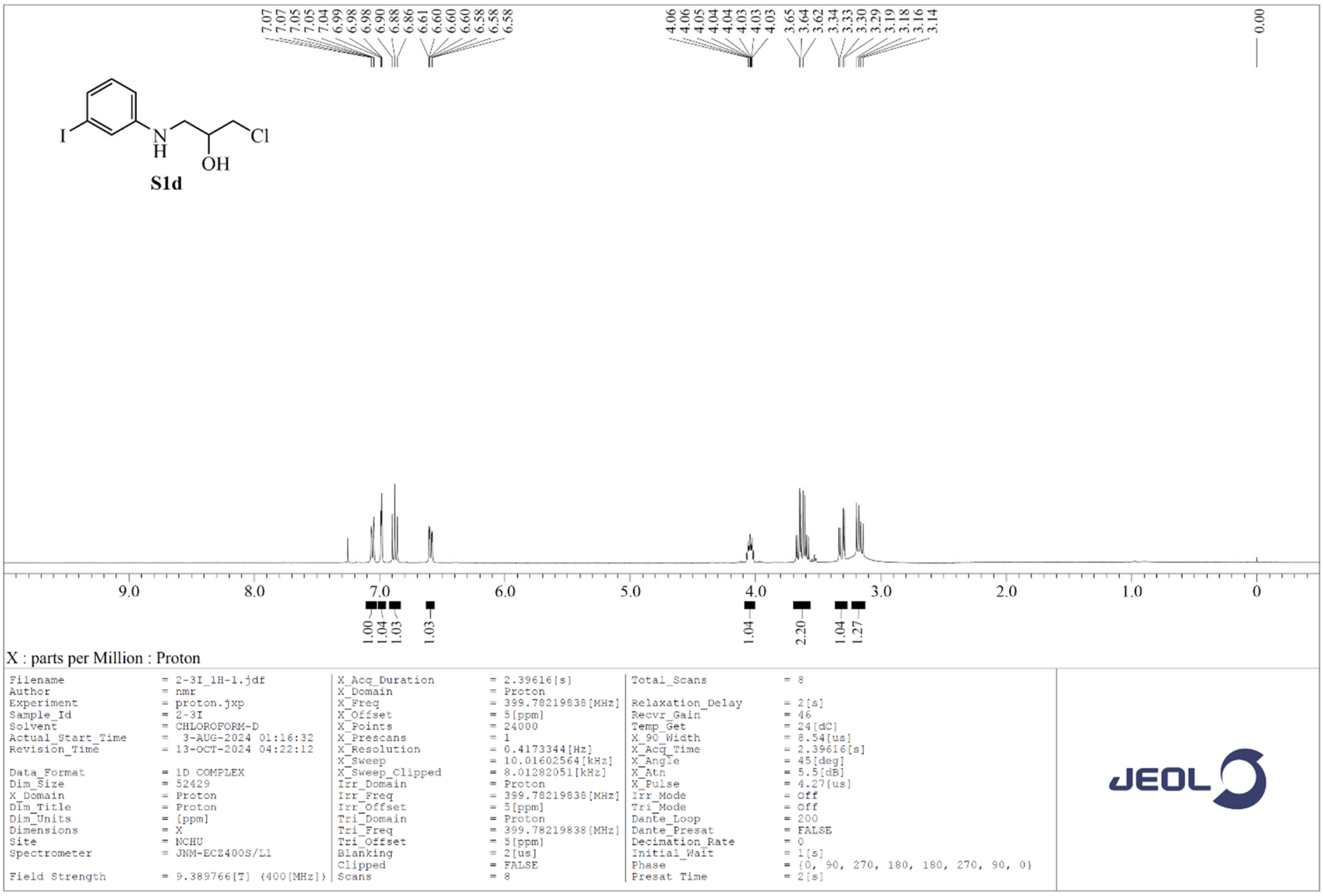


$^{1}$H NMR spectrum of compound **S1d** (400 MHz, $CDCl_3$)

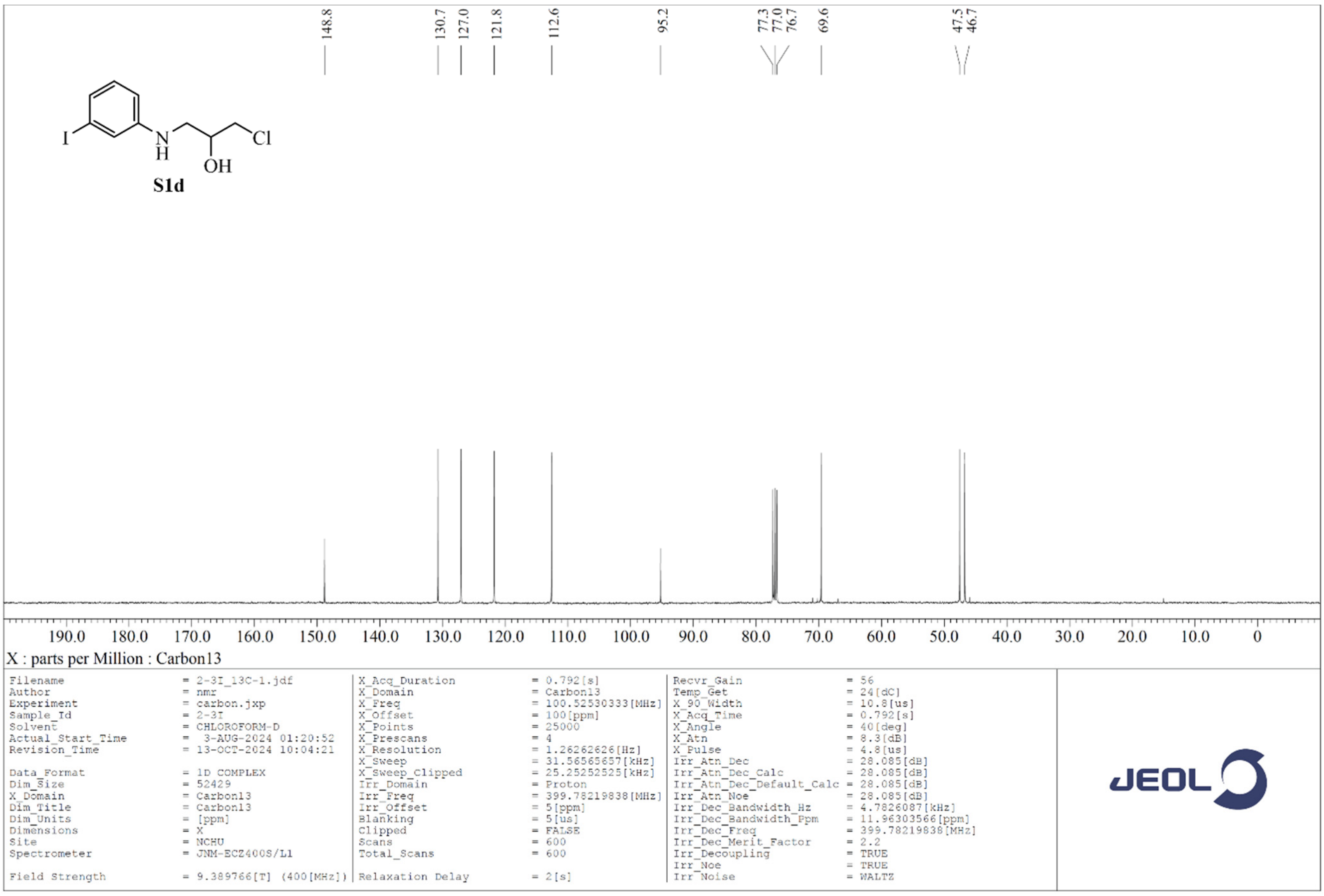


$^{13}$C NMR spectrum of compound **S1d** (101 MHz, $CDCl_3$)

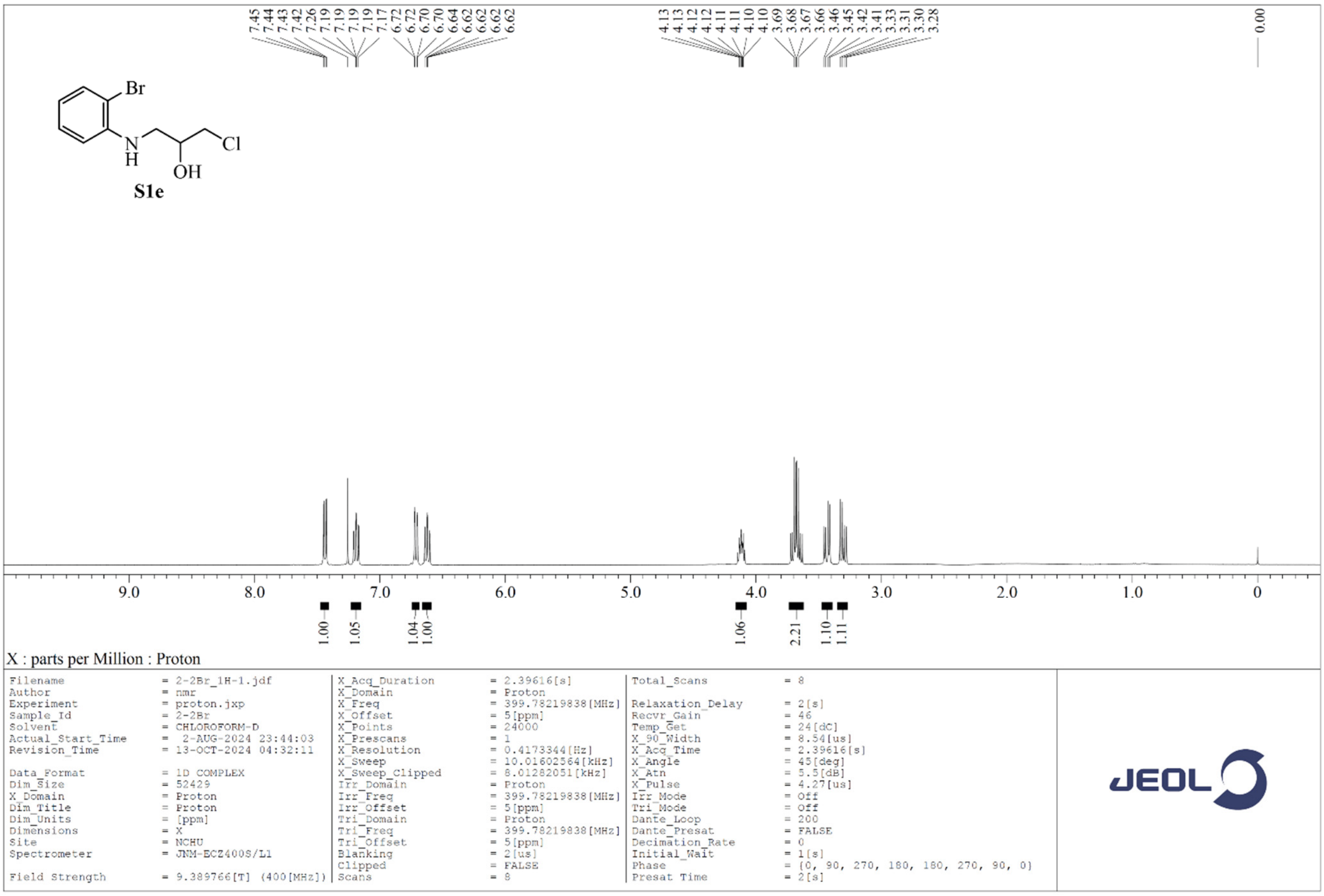


$^{1}H$ NMR spectrum of compound **S1e** (400 MHz, $CDCl_3$)

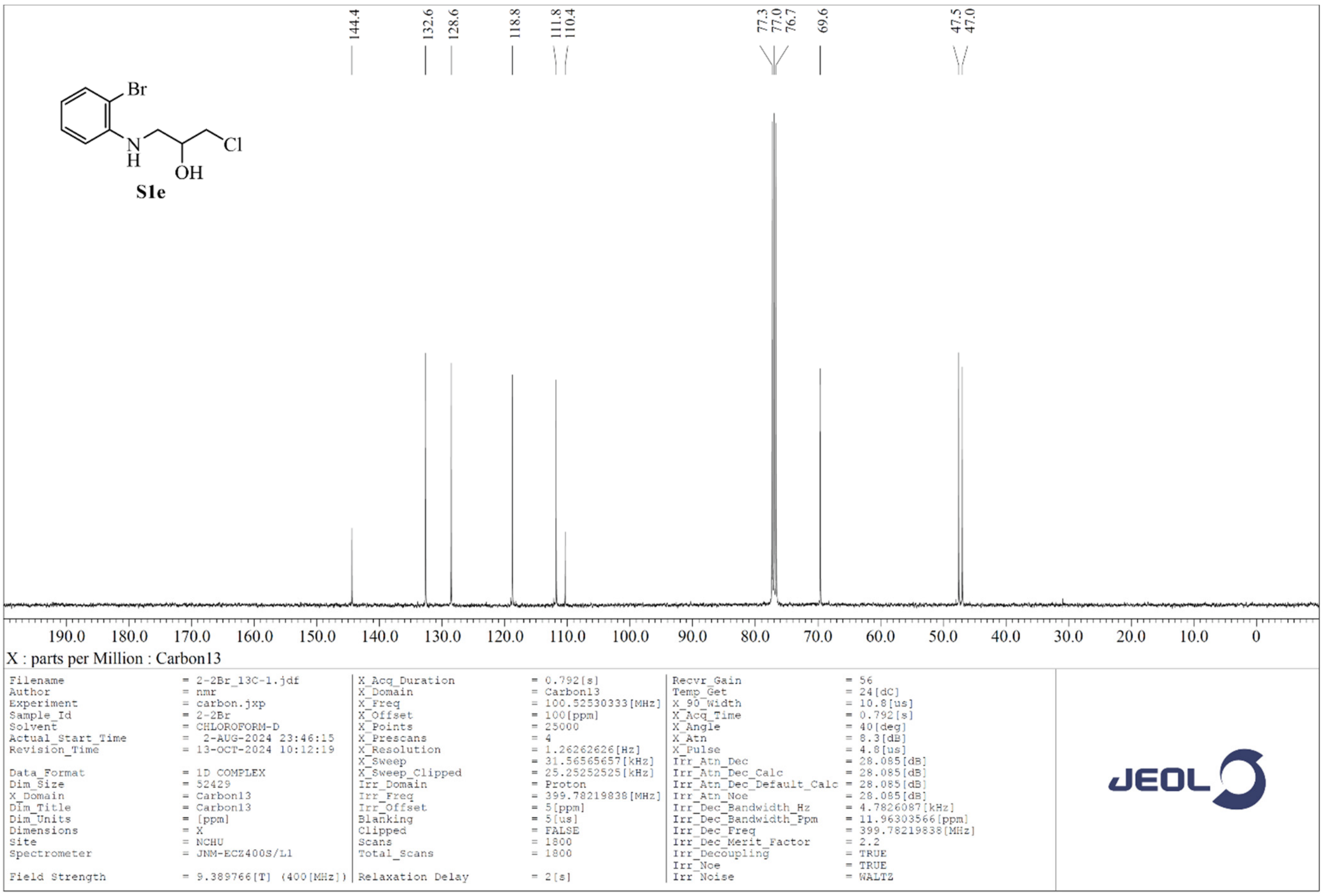


$^{13}C$ NMR spectrum of compound **S1e** (101 MHz, $CDCl_3$)

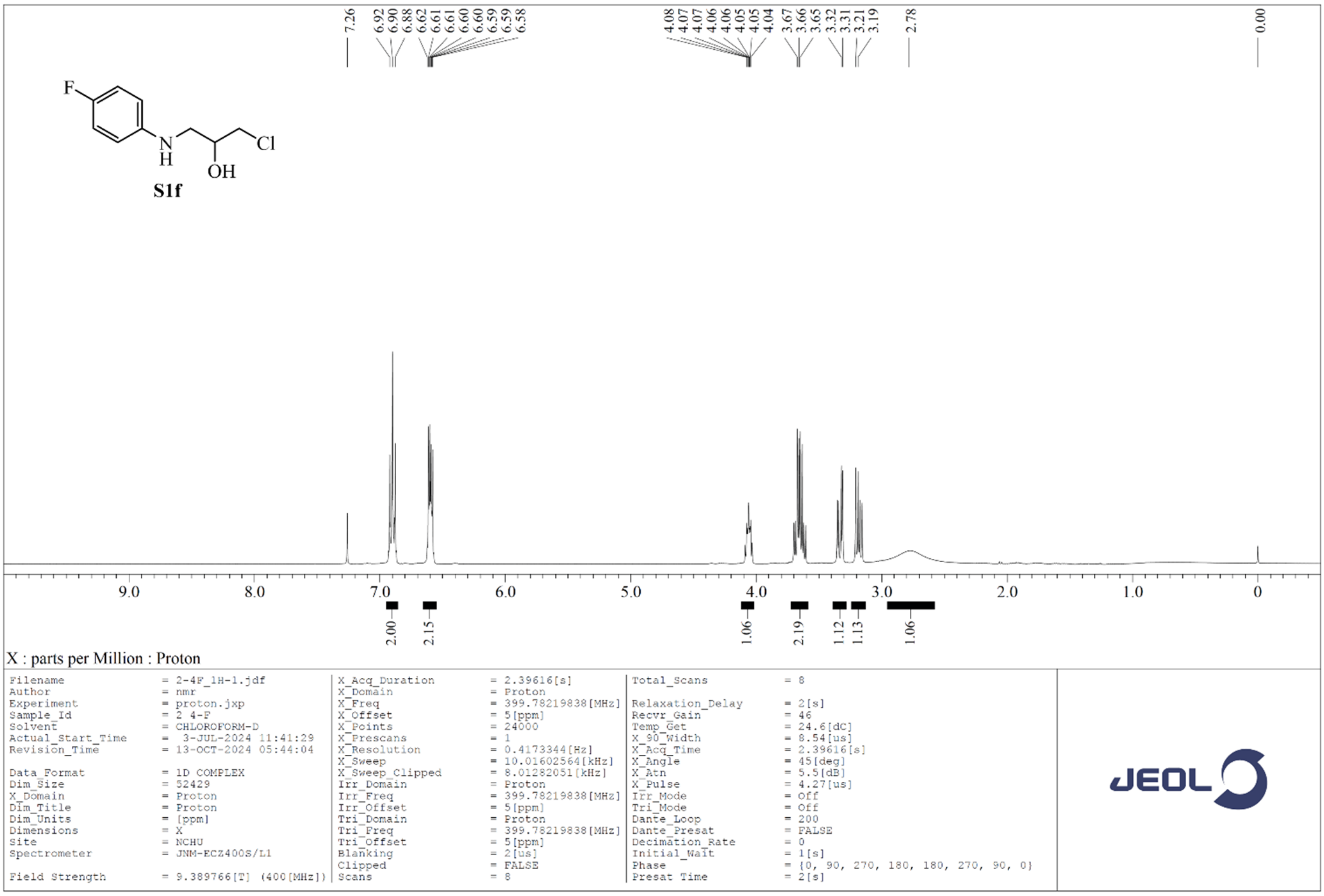


$^{1}H$ NMR spectrum of compound **S1f** (400 MHz, $CDCl_3$)

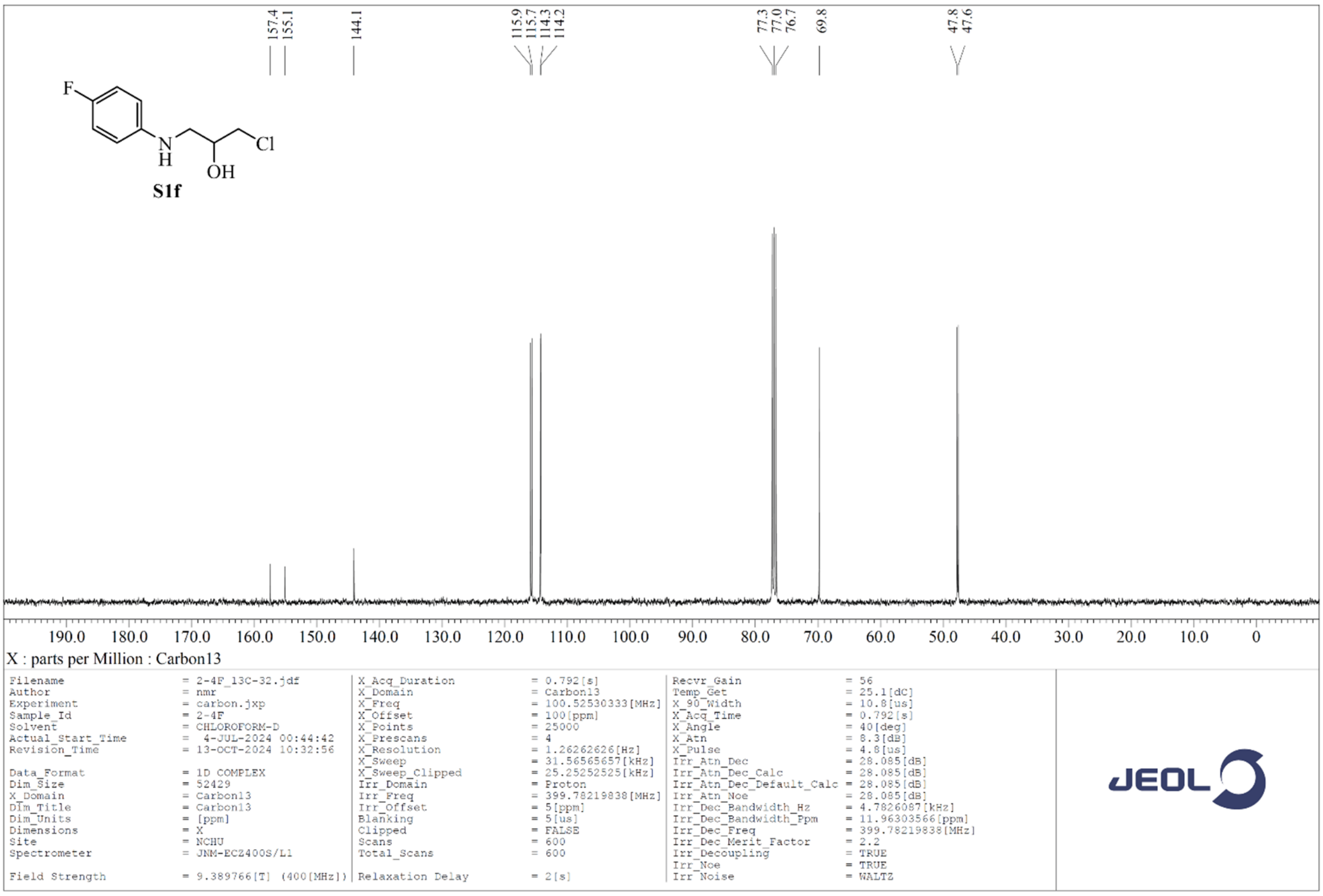


$^{13}C$ NMR spectrum of compound **S1f** (101 MHz, $CDCl_3$)

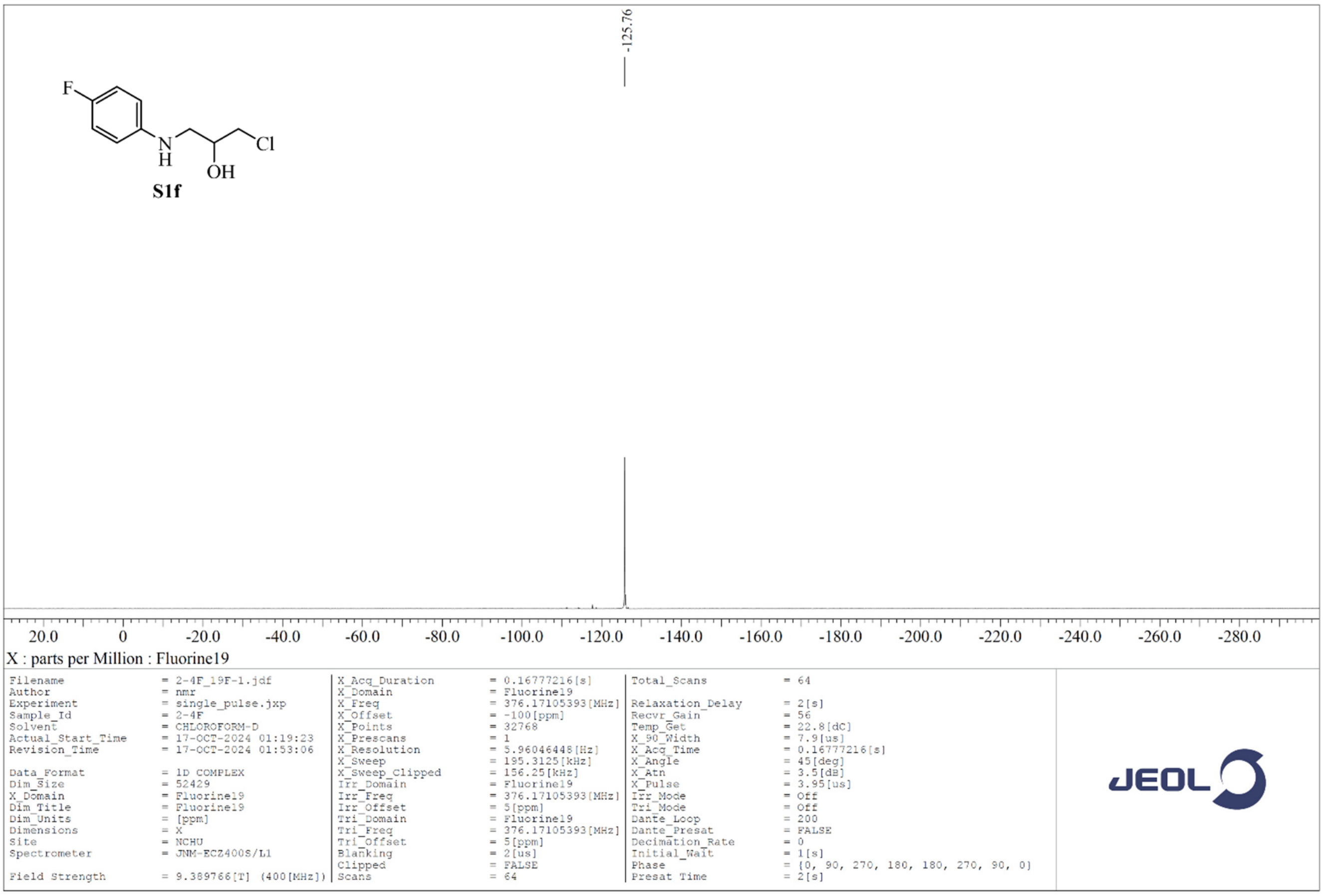


$^{19}$F NMR spectrum of compound **S1f** (376 MHz, $CDCl_3$)

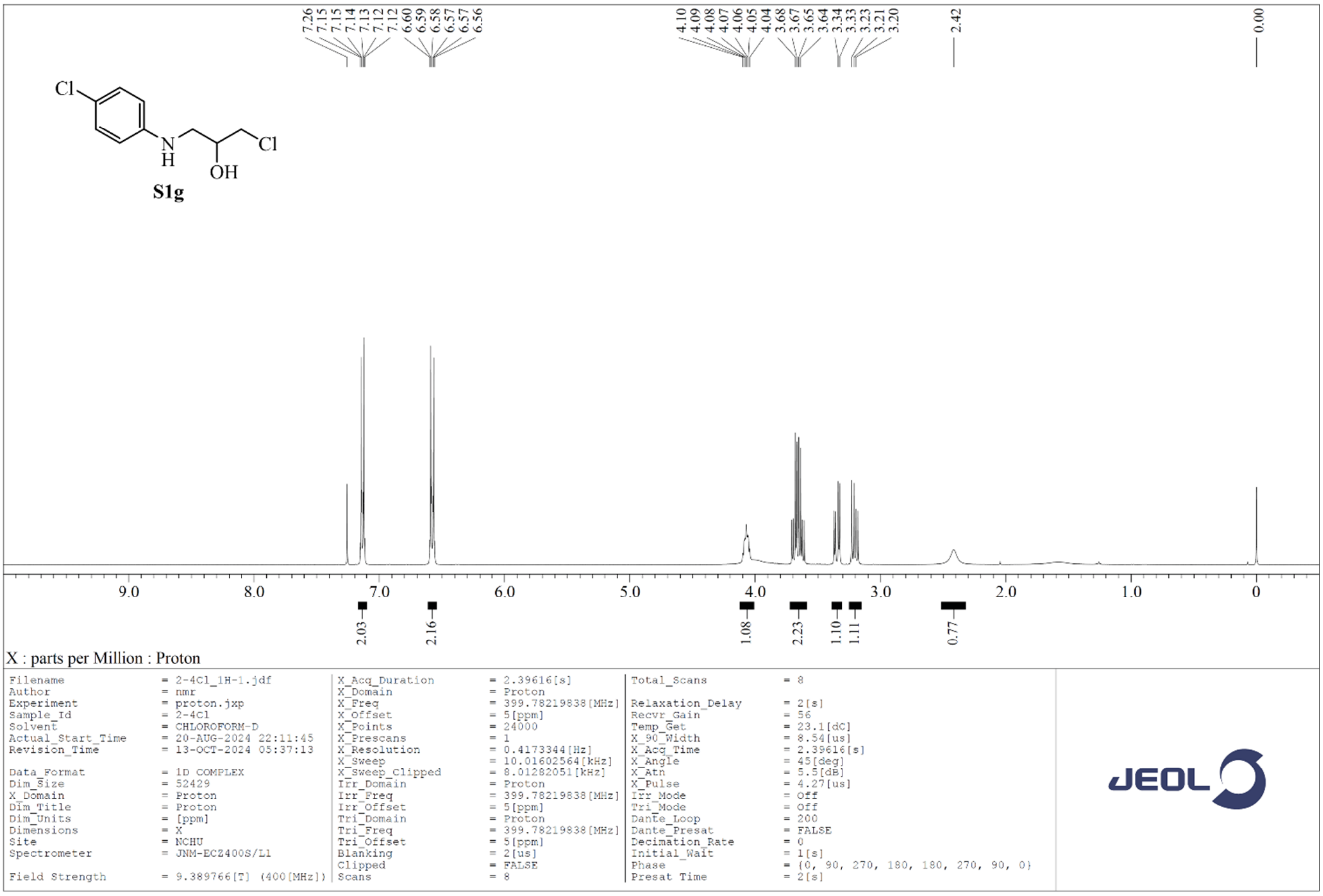


$^{1}$H NMR spectrum of compound **S1g** (400 MHz, $CDCl_3$)

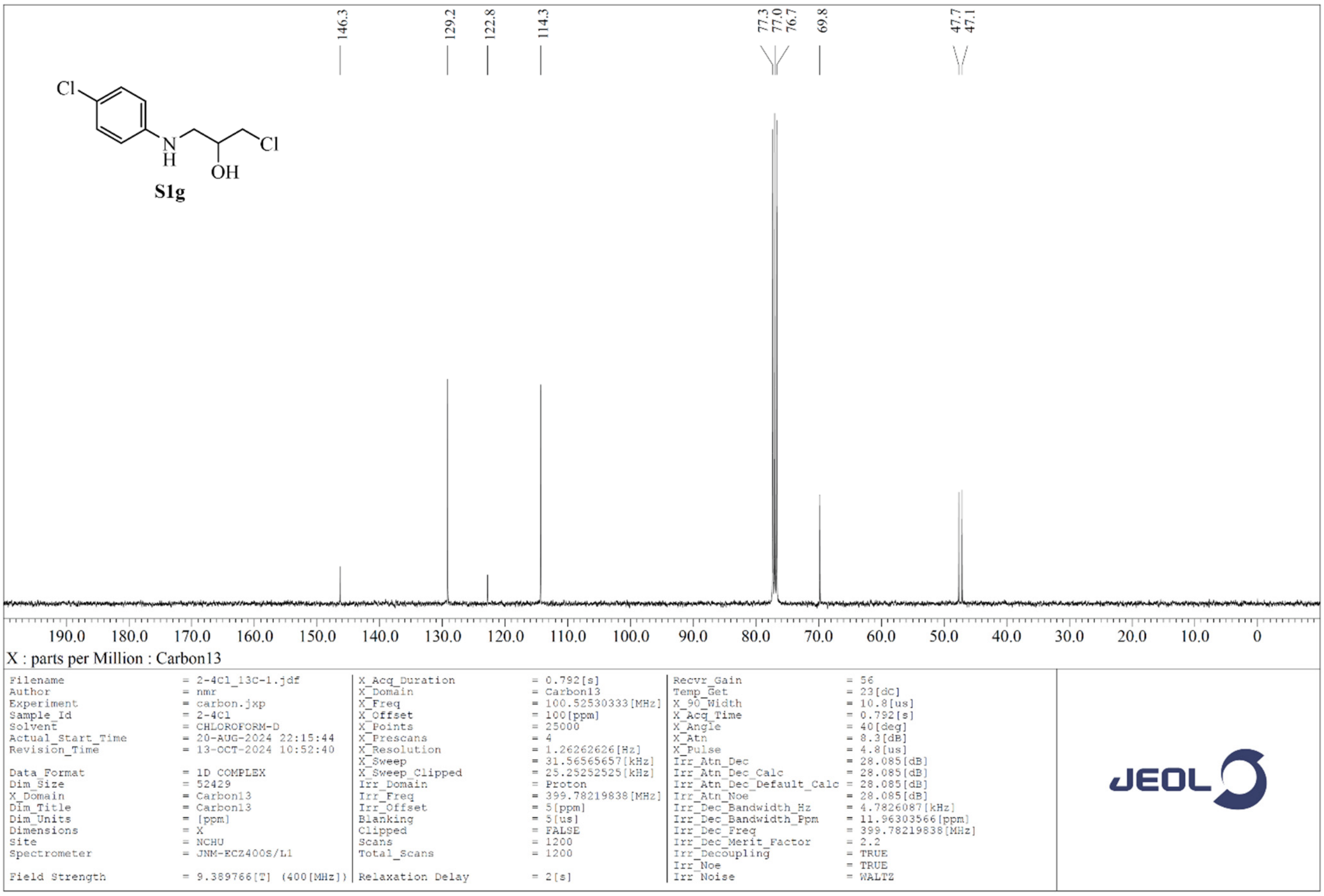


$^{13}$C NMR spectrum of compound **S1g** (101 MHz, $CDCl_3$)

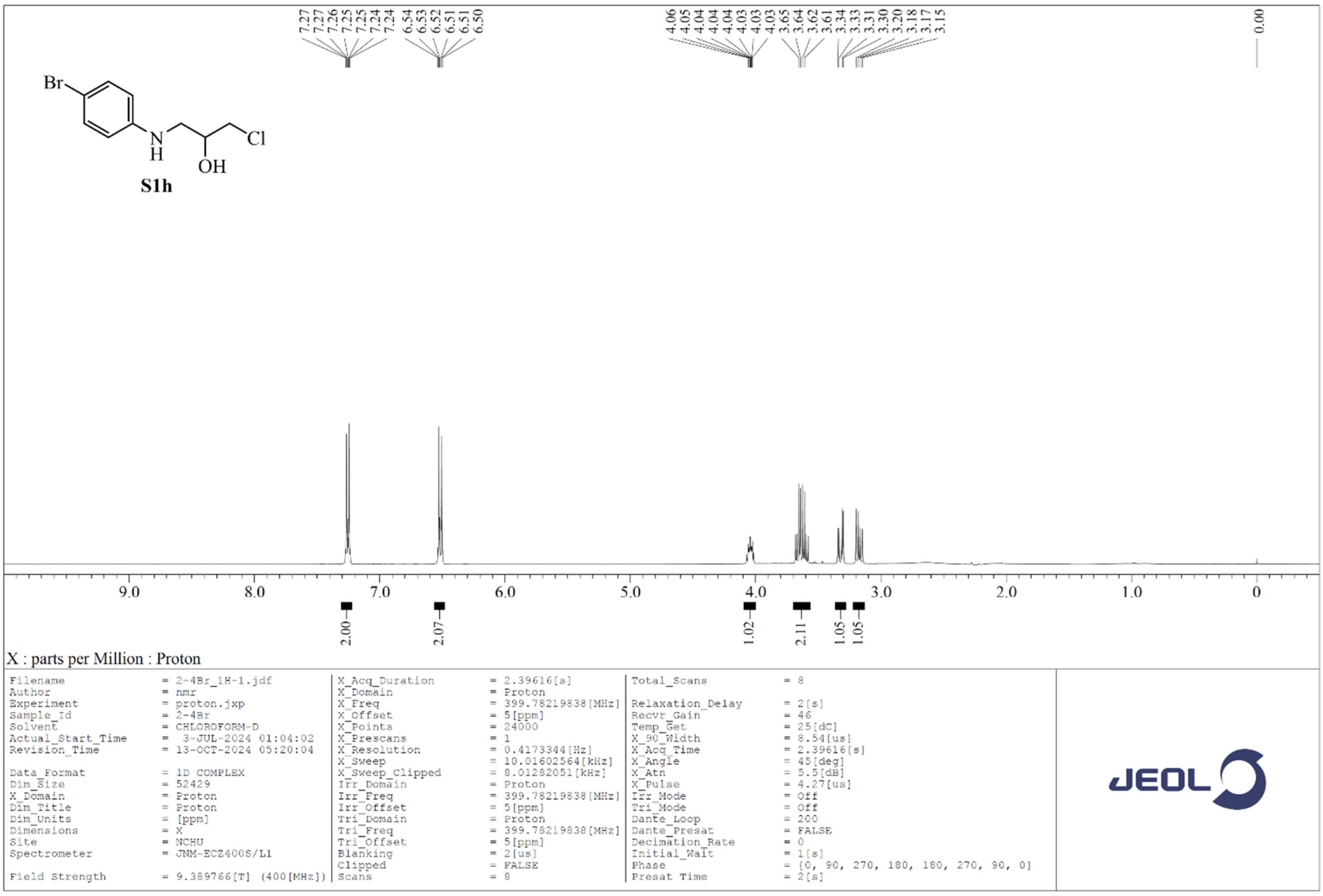


$^{1}$H NMR spectrum of compound **S1h** (400 MHz, $CDCl_3$)

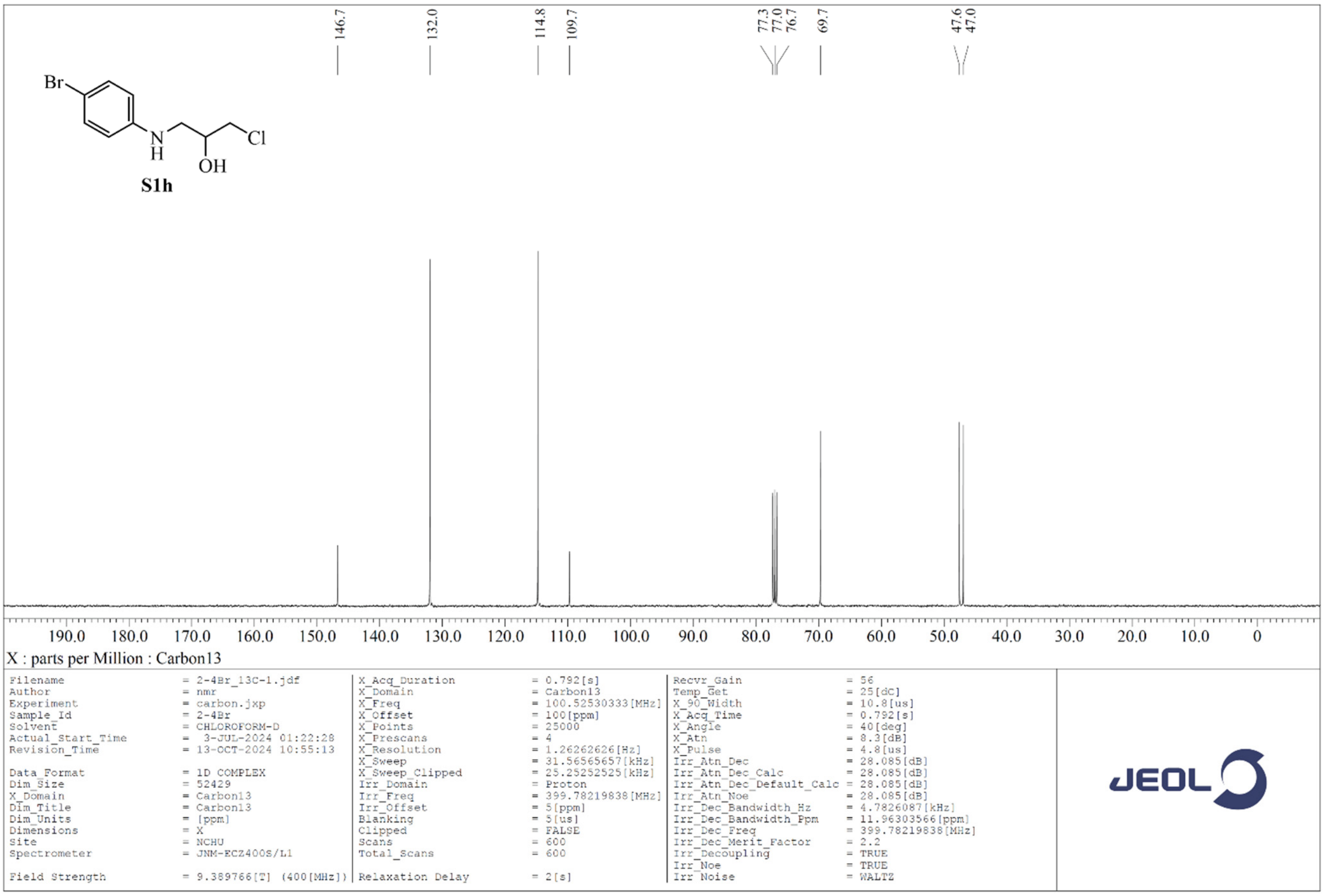


$^{13}C$ NMR spectrum of compound **S1h** (101 MHz, $CDCl_3$)

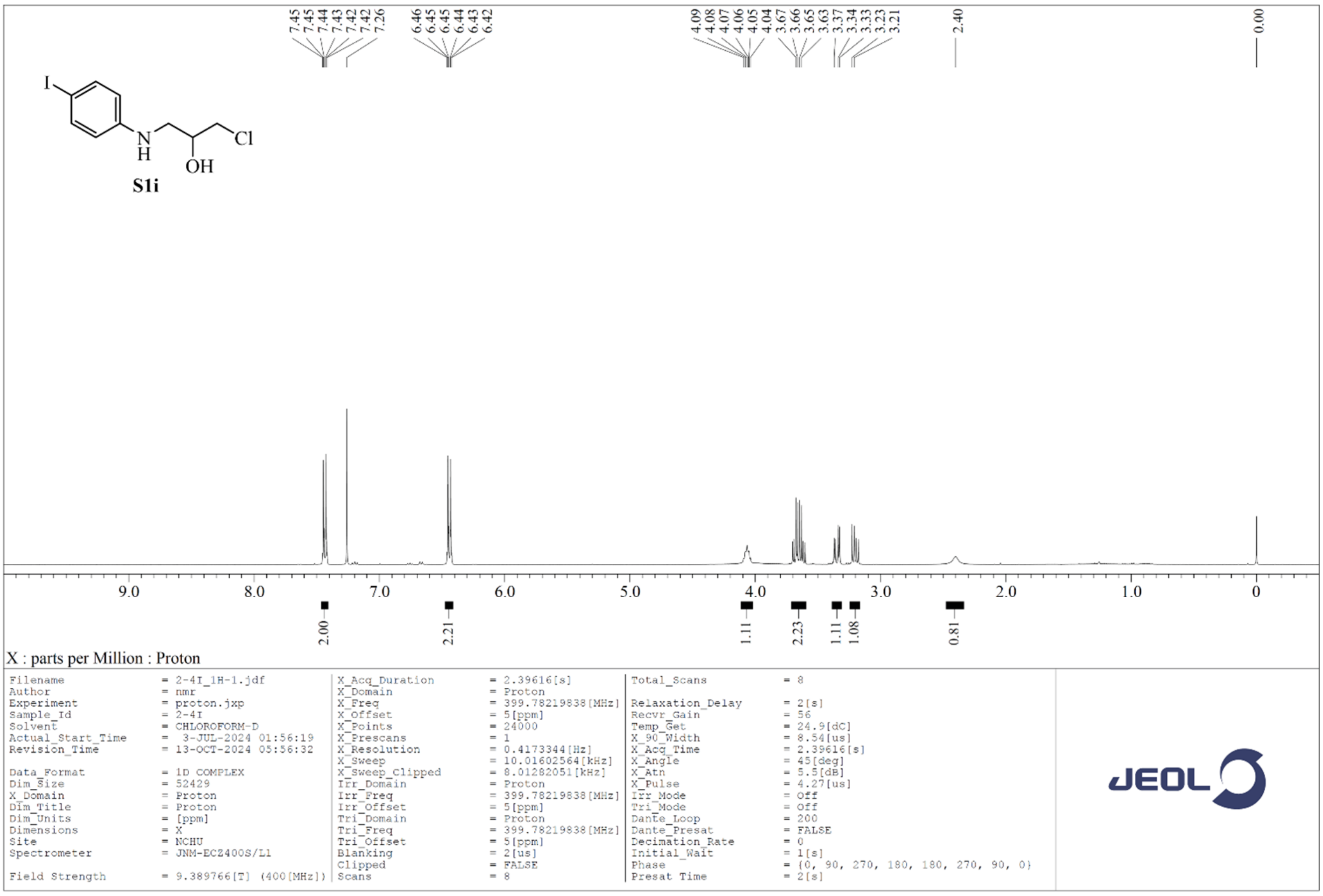


$^{1}H$ NMR spectrum of compound **S1i** (400 MHz, $CDCl_3$)

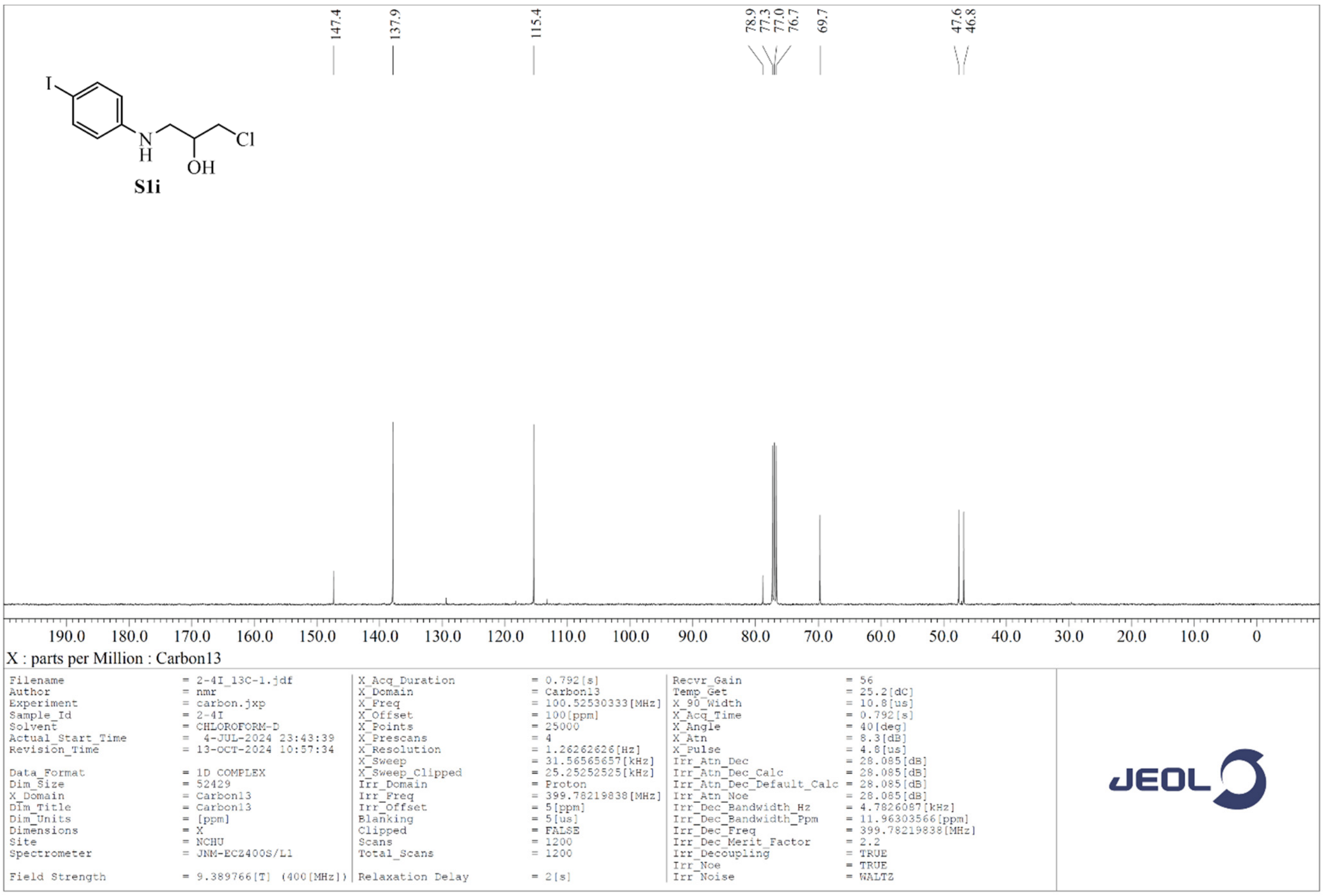


$^{13}C$ NMR spectrum of compound **S1i** (101 MHz, $CDCl_3$)

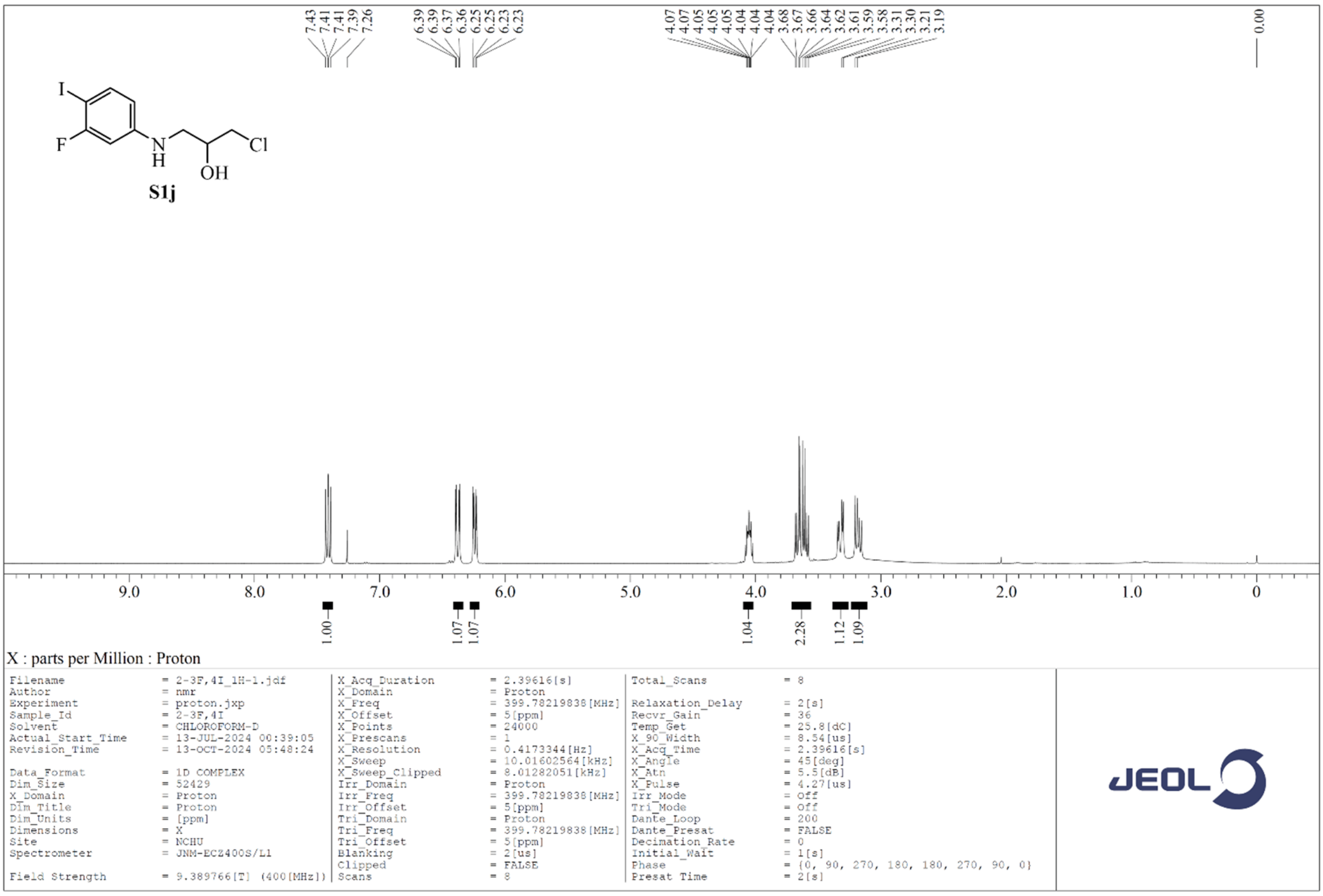


$^{1}H$ NMR spectrum of compound **S1j** (400 MHz, $CDCl_3$)

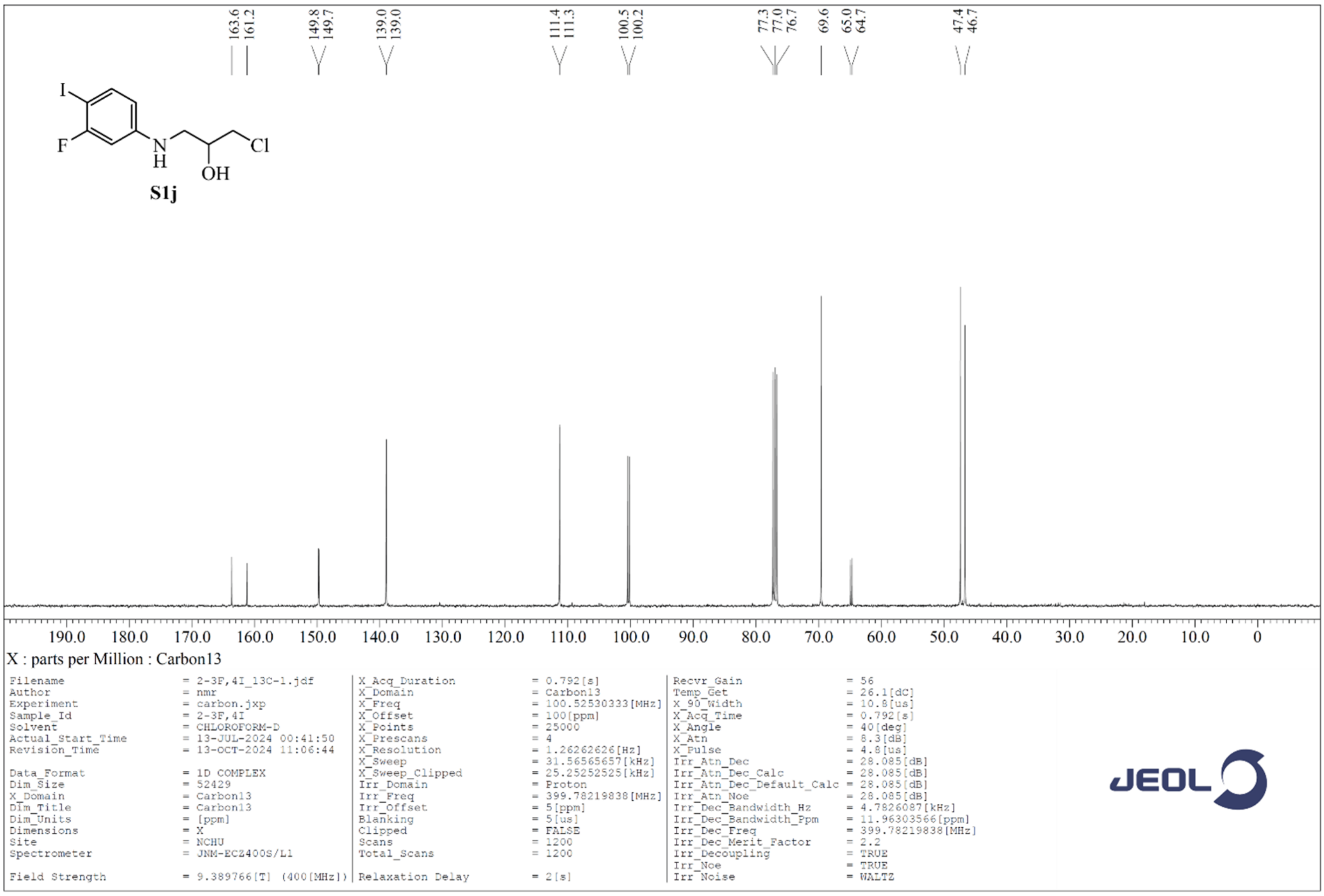


$^{13}$C NMR spectrum of compound **S1j** (101 MHz, $CDCl_3$)

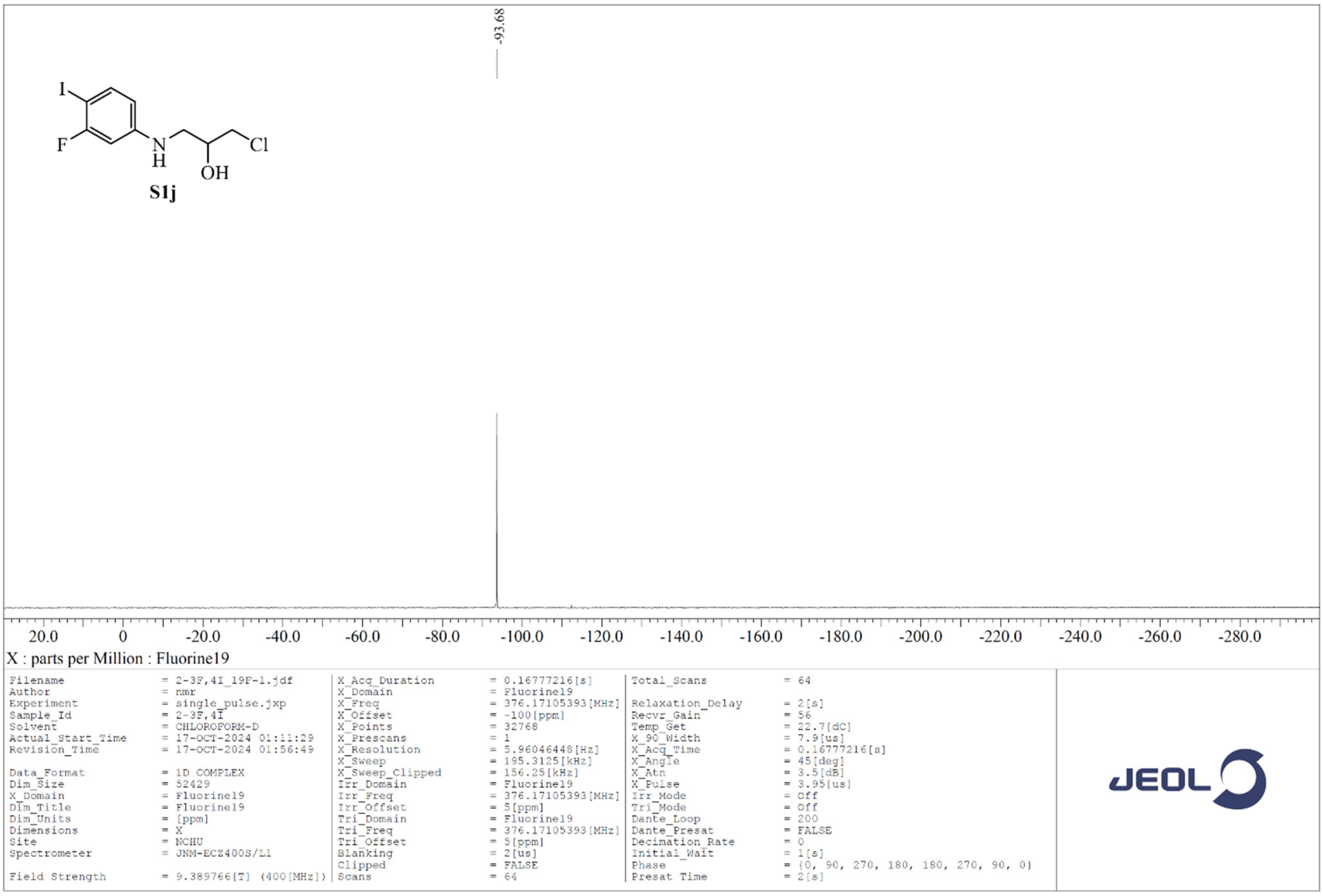


$^{19}$F NMR spectrum of compound **S1j** (376 MHz, $CDCl_3$)

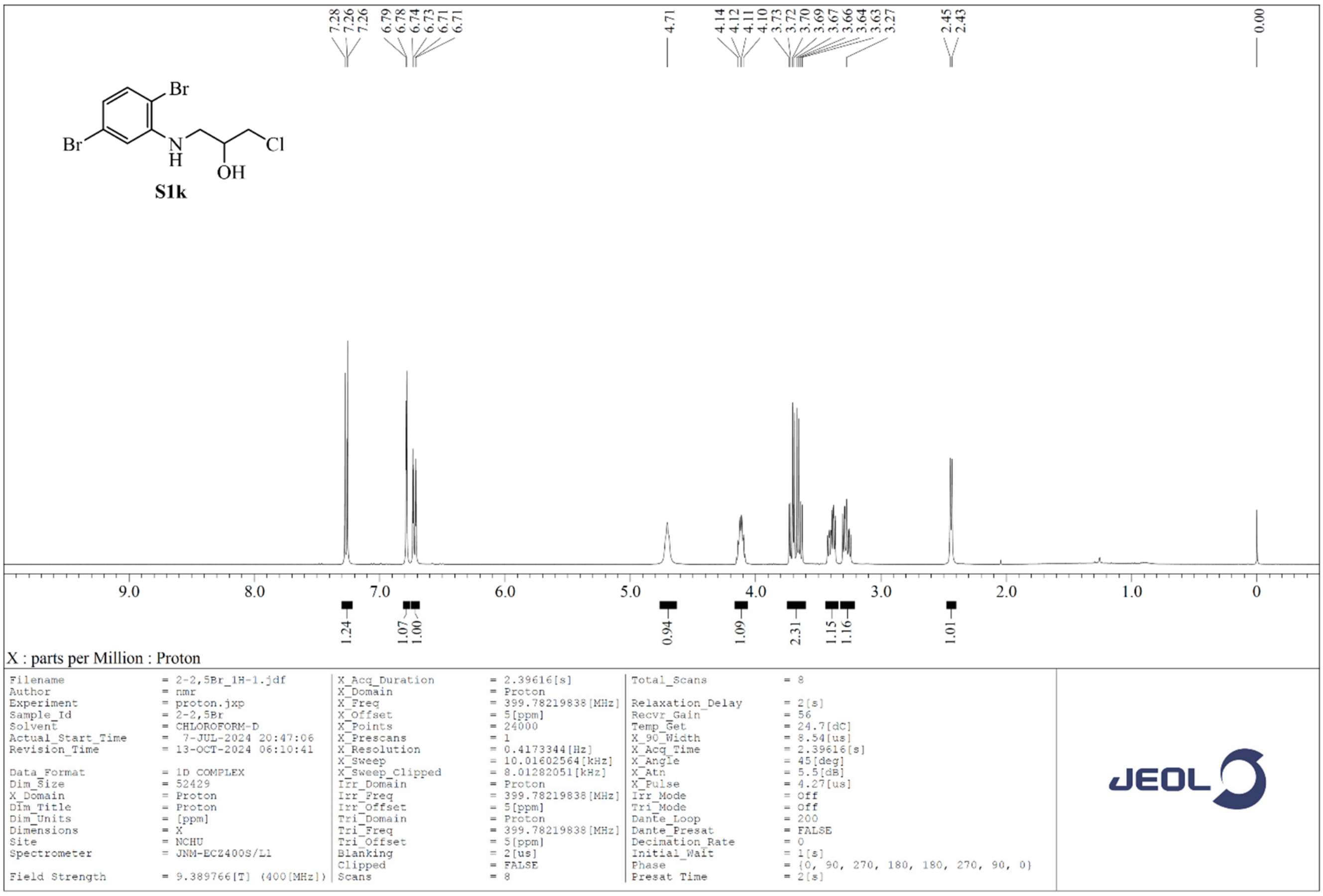


[1] H NMR spectrum of compound **S1k** (400 MHz, $CDCl_3$)

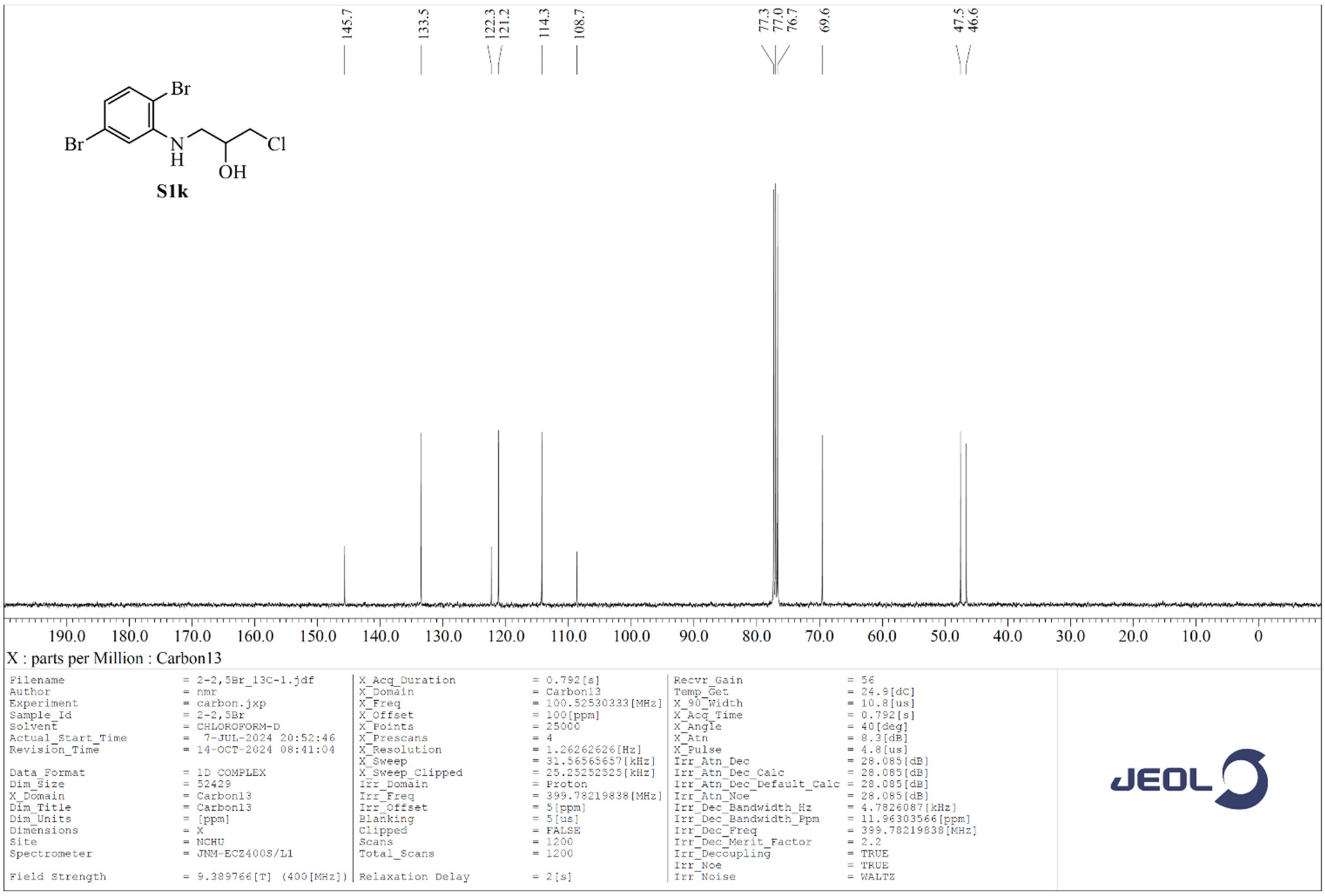


[13] C NMR spectrum of compound **S1k** (101 MHz, $CDCl_3$)

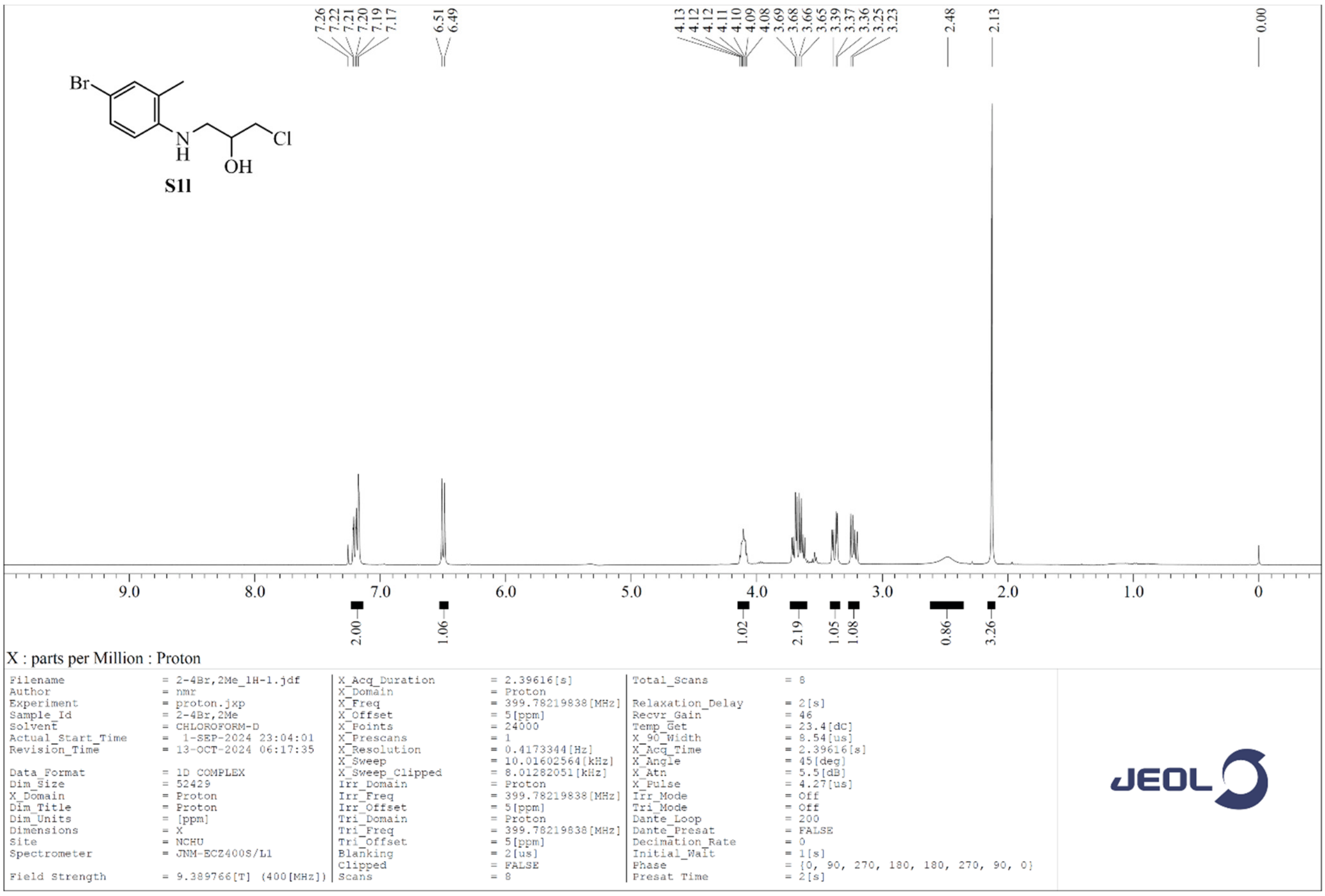


$^{1}$H NMR spectrum of compound **S11** (400 MHz, $CDCl_3$)

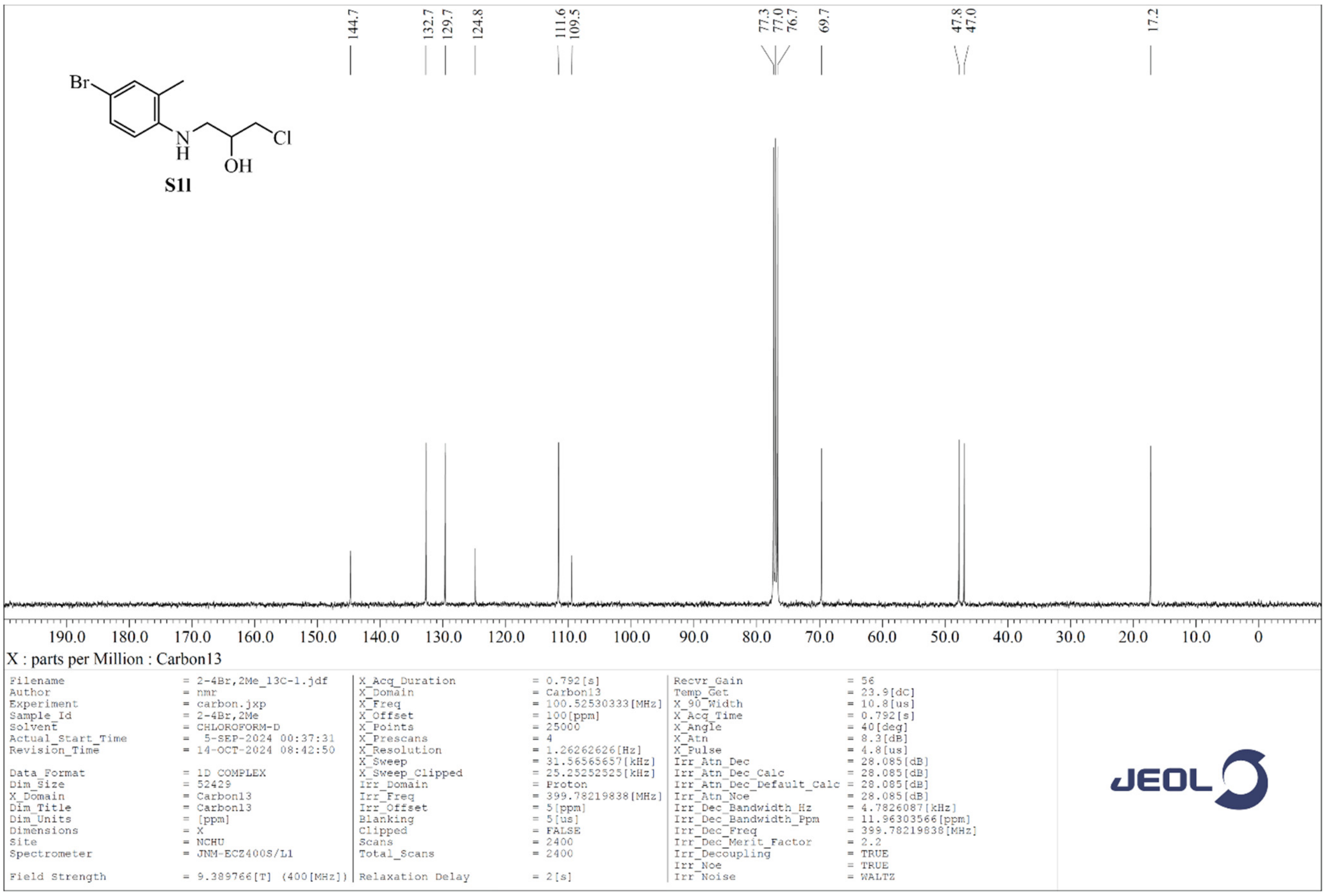


$^{13}$C NMR spectrum of compound **S11** (101 MHz, $CDCl_3$)

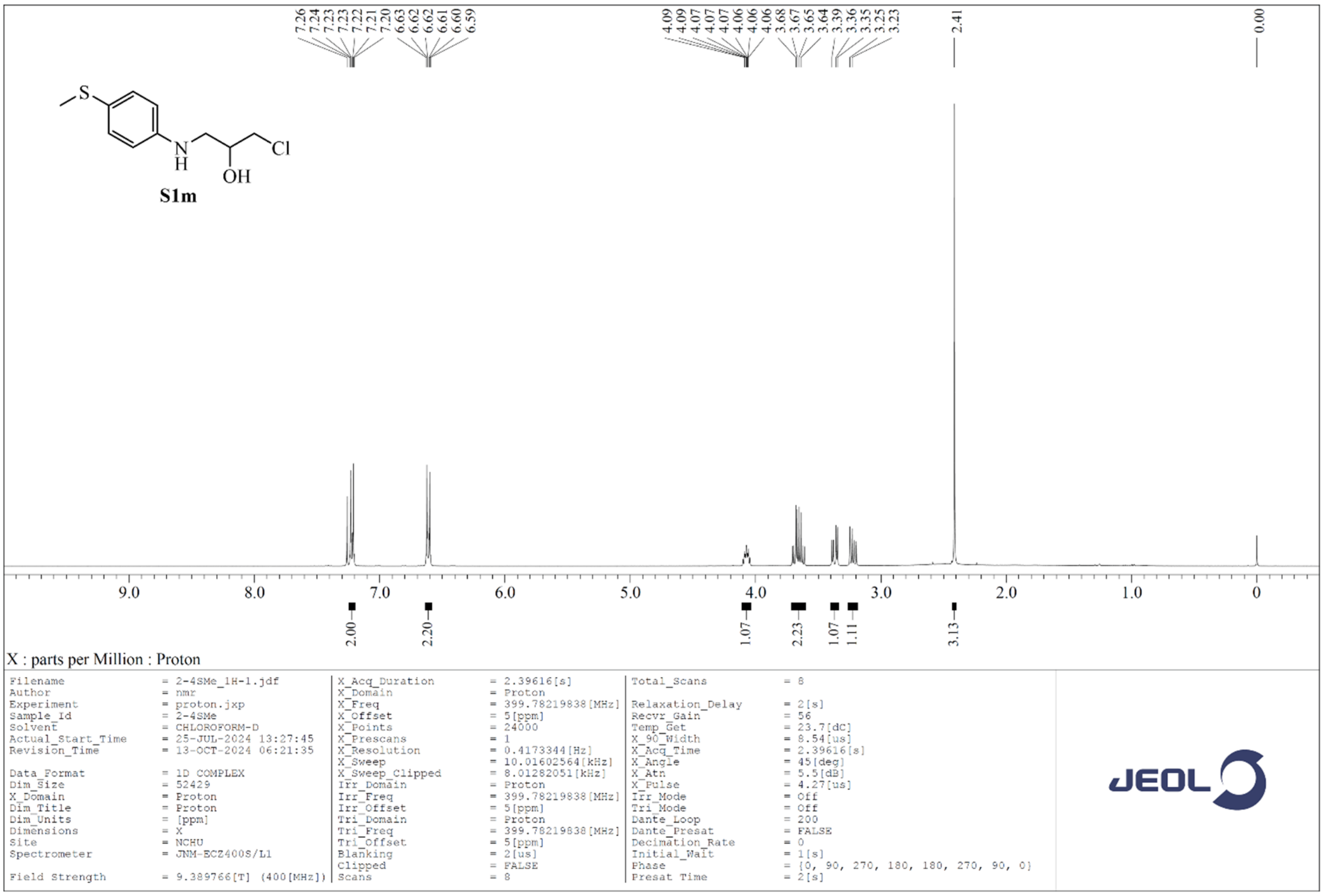


$^{1}$H NMR spectrum of compound **S1m** (400 MHz, $CDCl_3$)

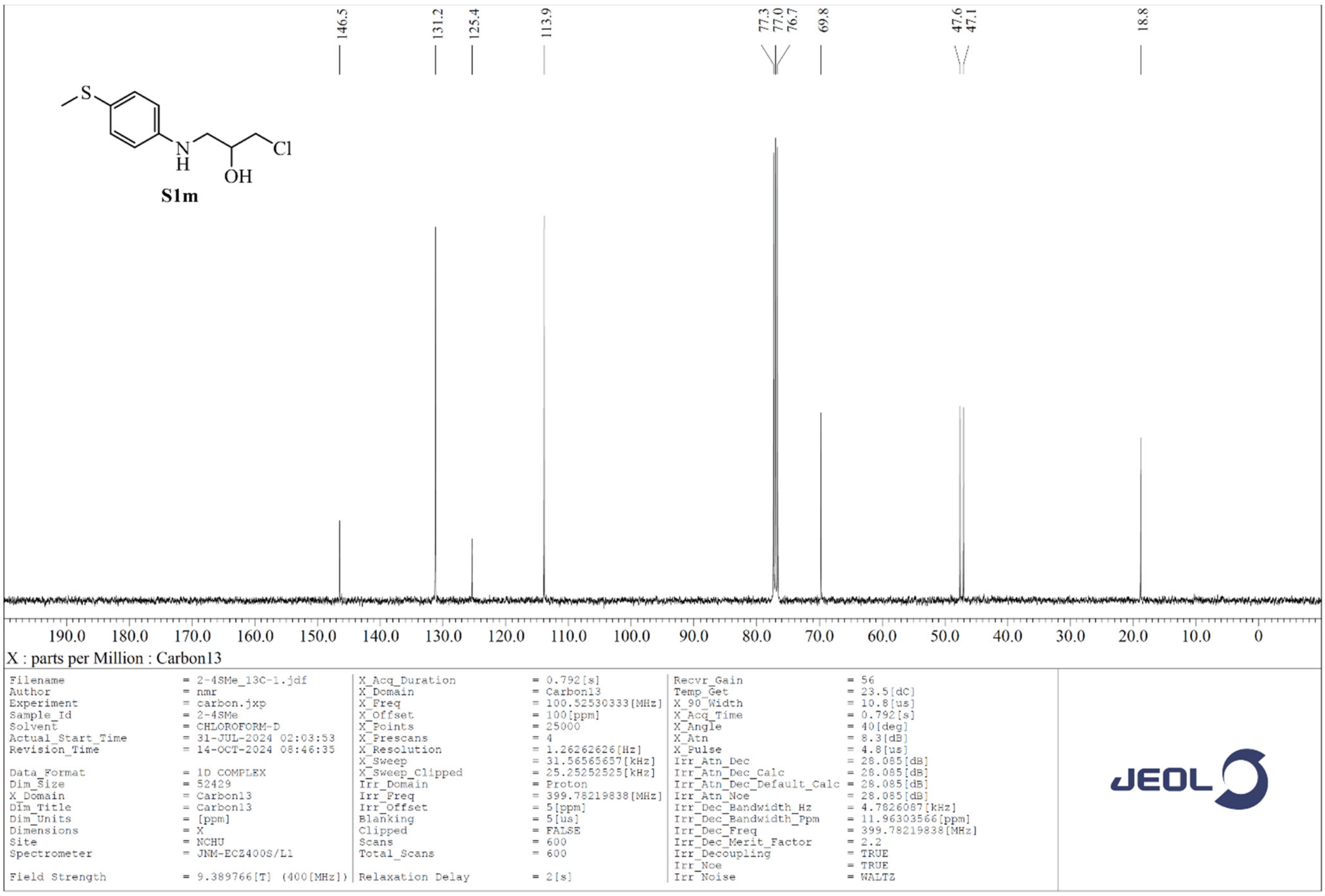


$^{13}$C NMR spectrum of compound **S1m** (101 MHz, $CDCl_3$)

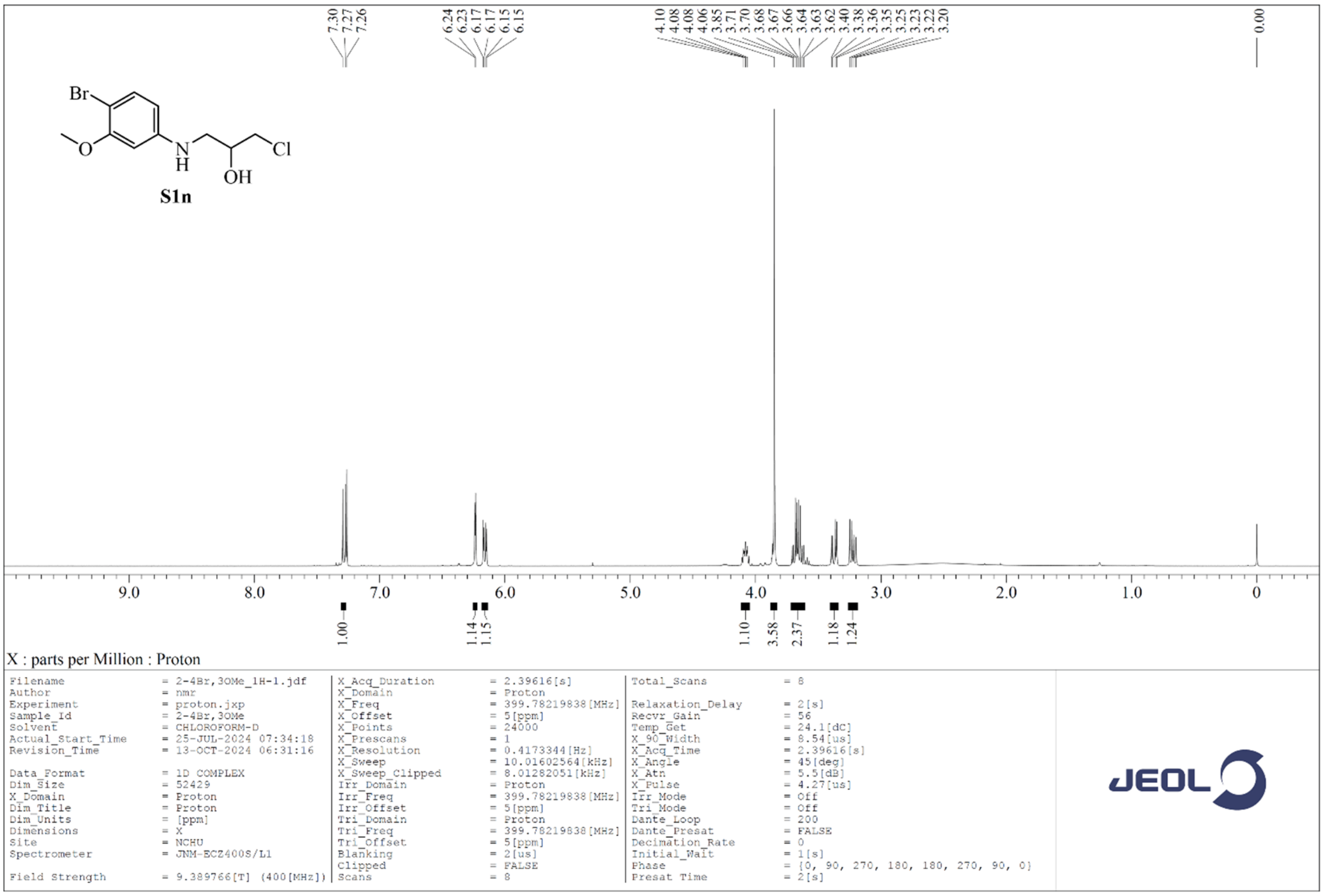


$^{1}H$ NMR spectrum of compound **S1n** (400 MHz, $CDCl_3$)

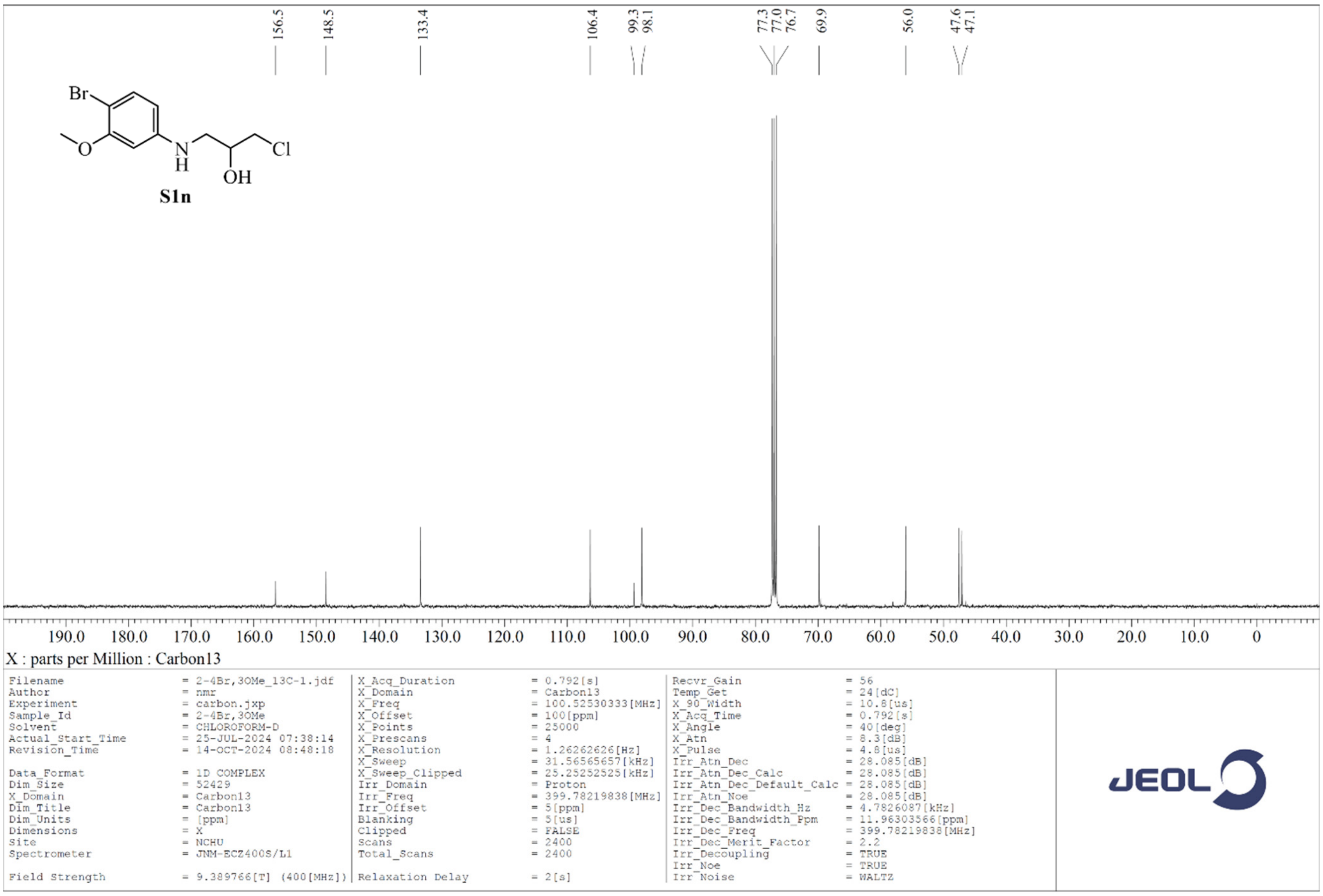


$^{13}C$ NMR spectrum of compound **S1n** (101 MHz, $CDCl_3$)

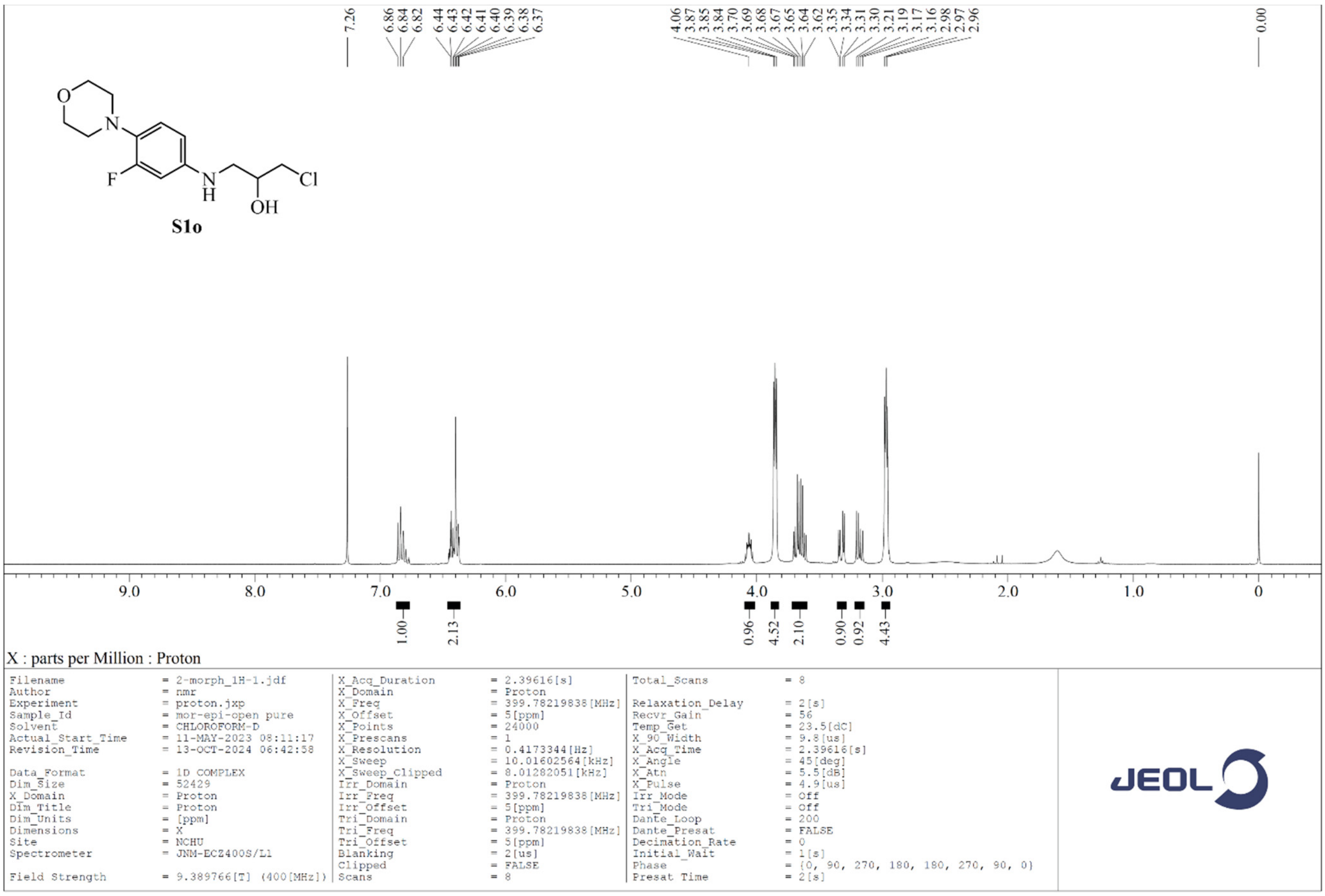


$^{1}$H NMR spectrum of compound **S1o** (400 MHz, $CDCl_3$)

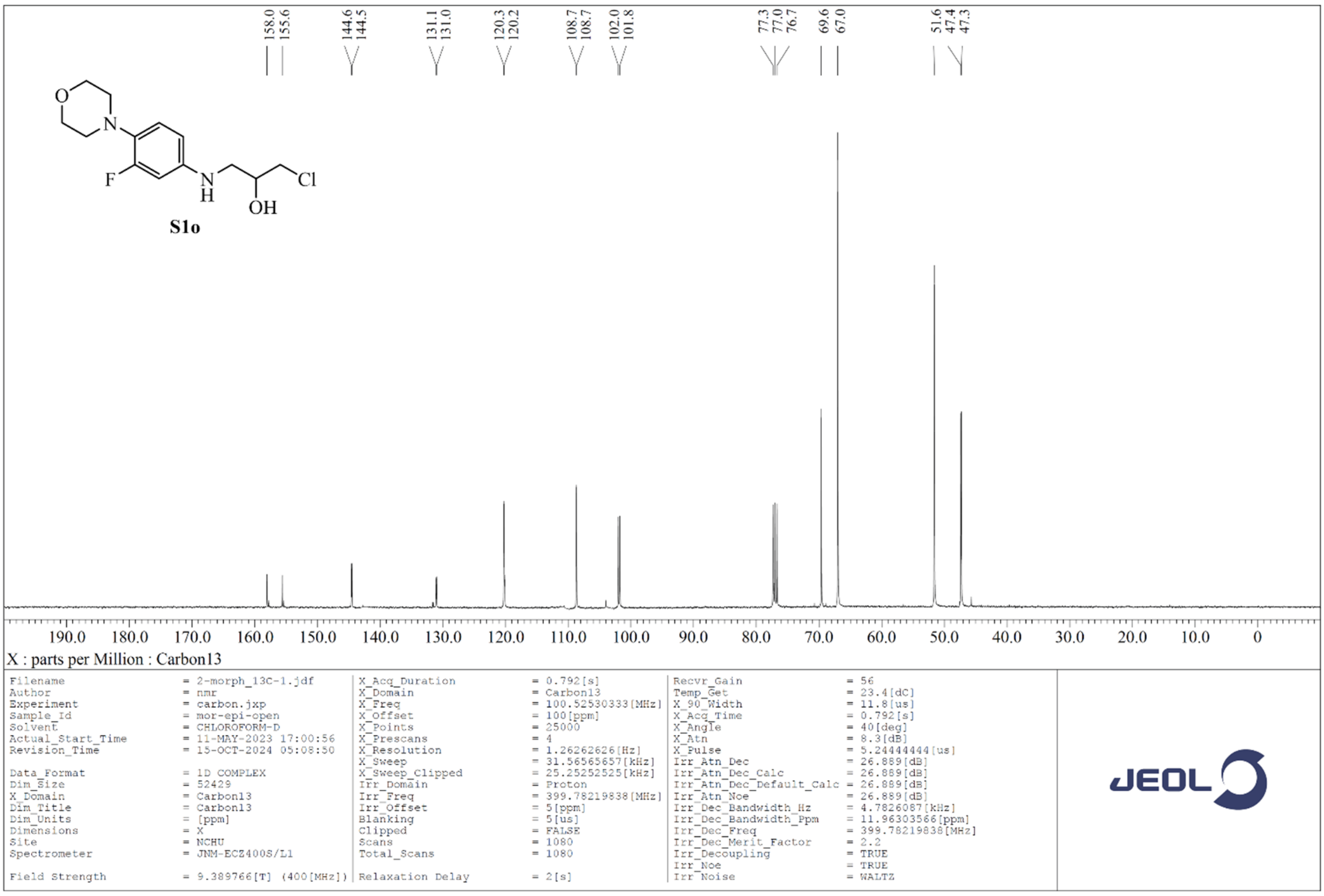


$^{13}$C NMR spectrum of compound **S1o** (101 MHz, $CDCl_3$)

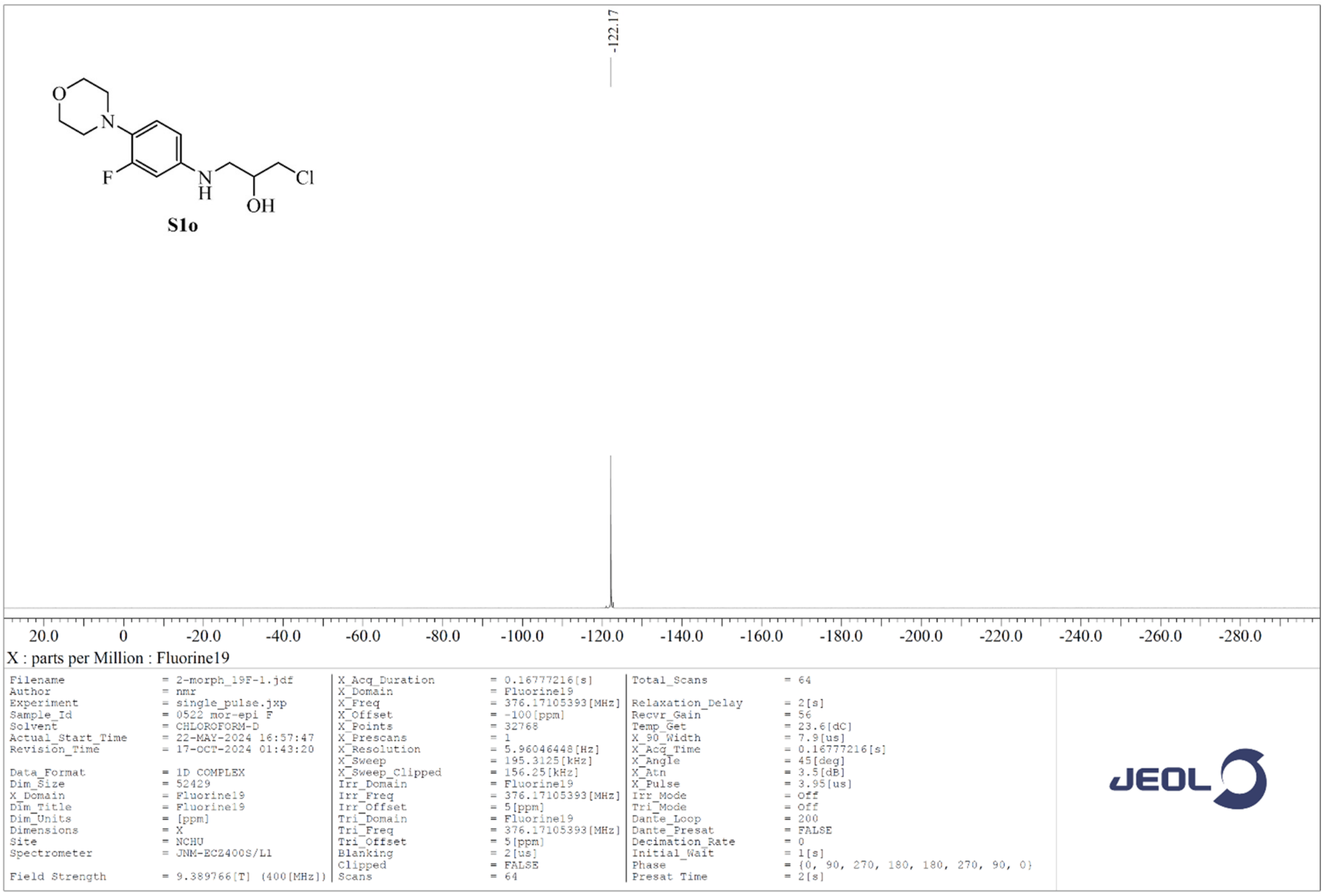


$^{19}$F NMR spectrum of compound **S1o** (376 MHz, $CDCl_3$)

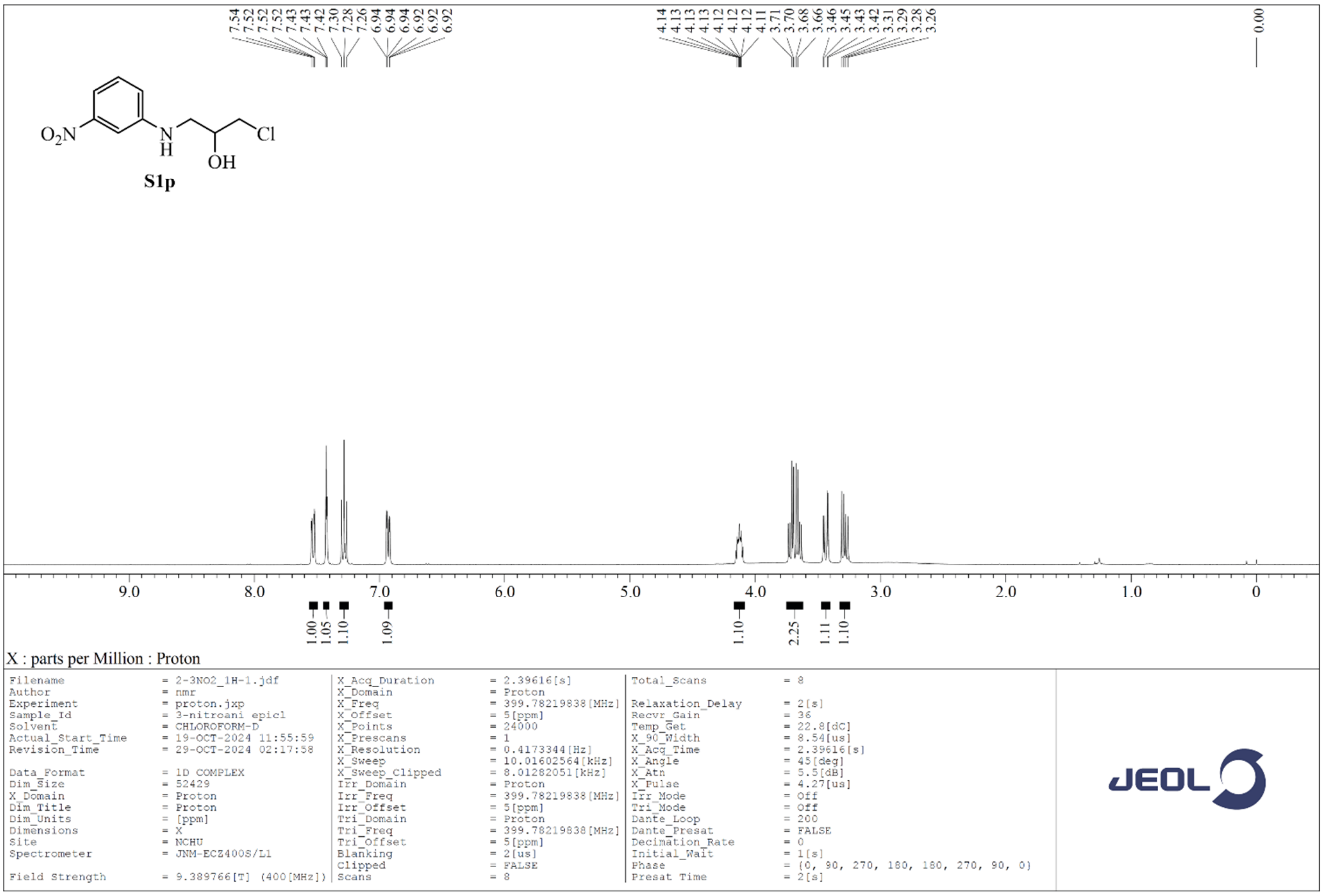


$^{1}$H NMR spectrum of compound **S1p** (400 MHz, $CDCl_3$)

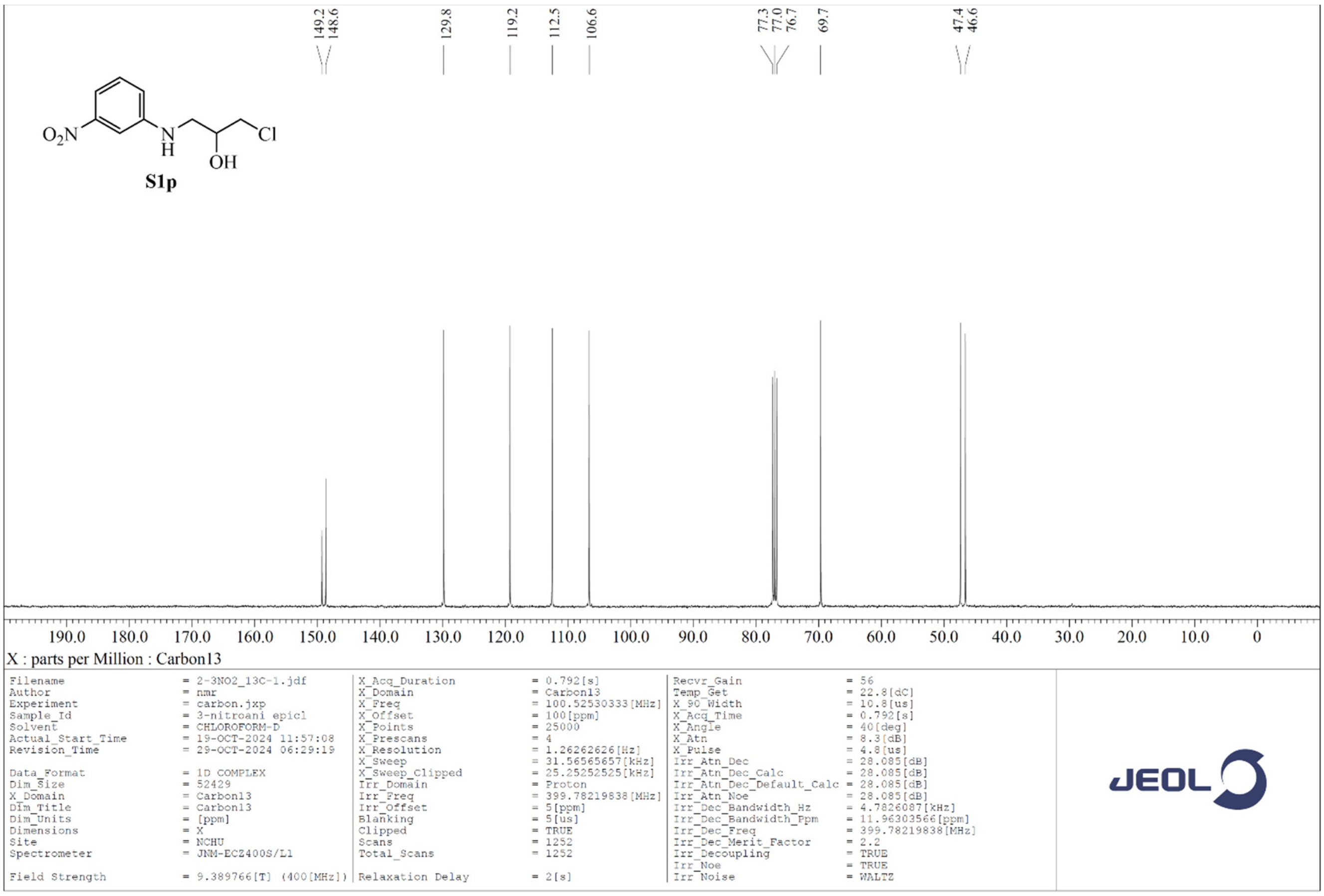


$^{13}C$ NMR spectrum of compound **S1p** (101 MHz, $CDCl_3$)

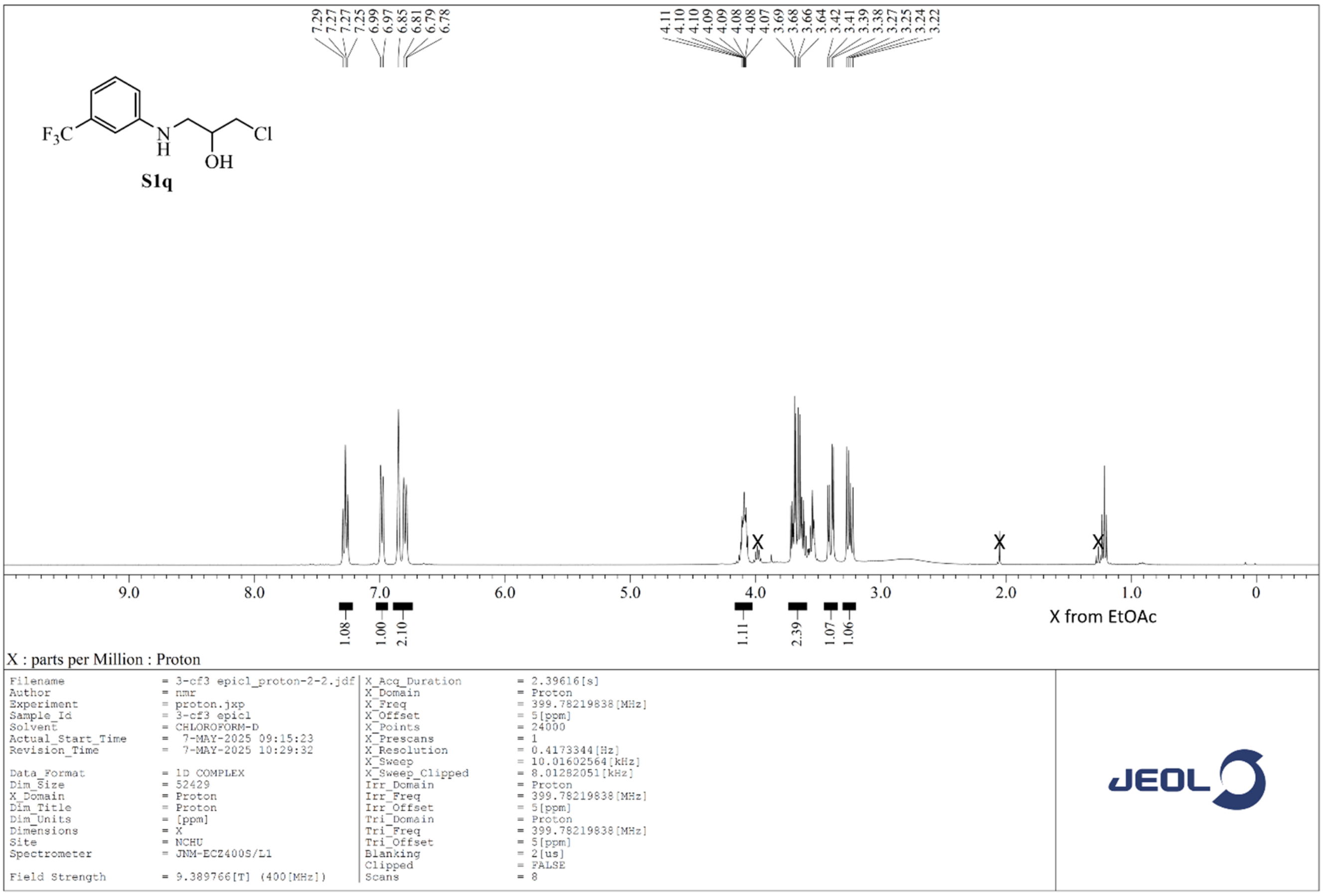


$^{1}H$ NMR spectrum of compound **S1q** (400 MHz, $CDCl_3$)

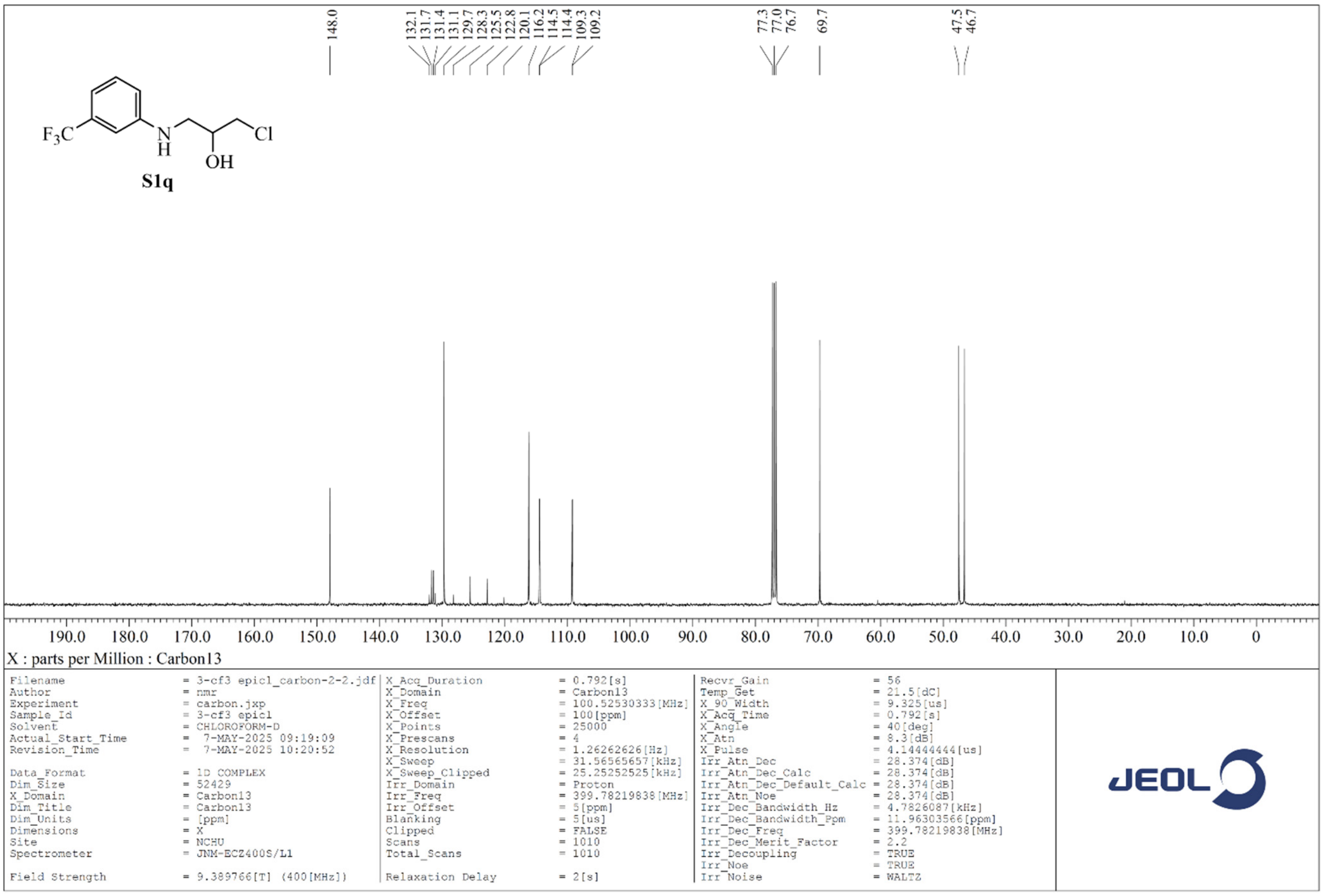


$^{13}$C NMR spectrum of compound **S1q** (101 MHz, $CDCl_3$)

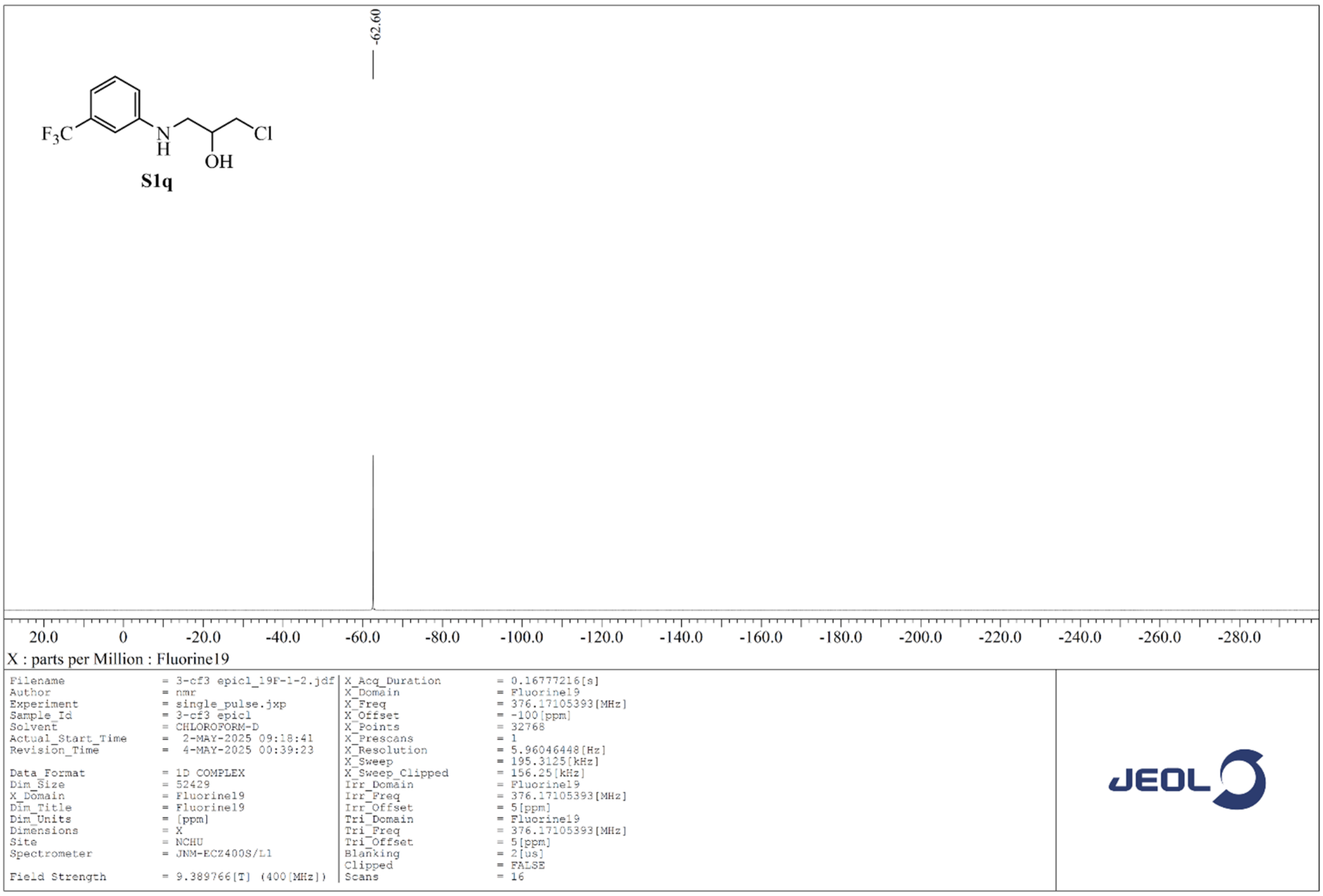


$^{19}$F NMR spectrum of compound **S1q** (376 MHz, $CDCl_3$)

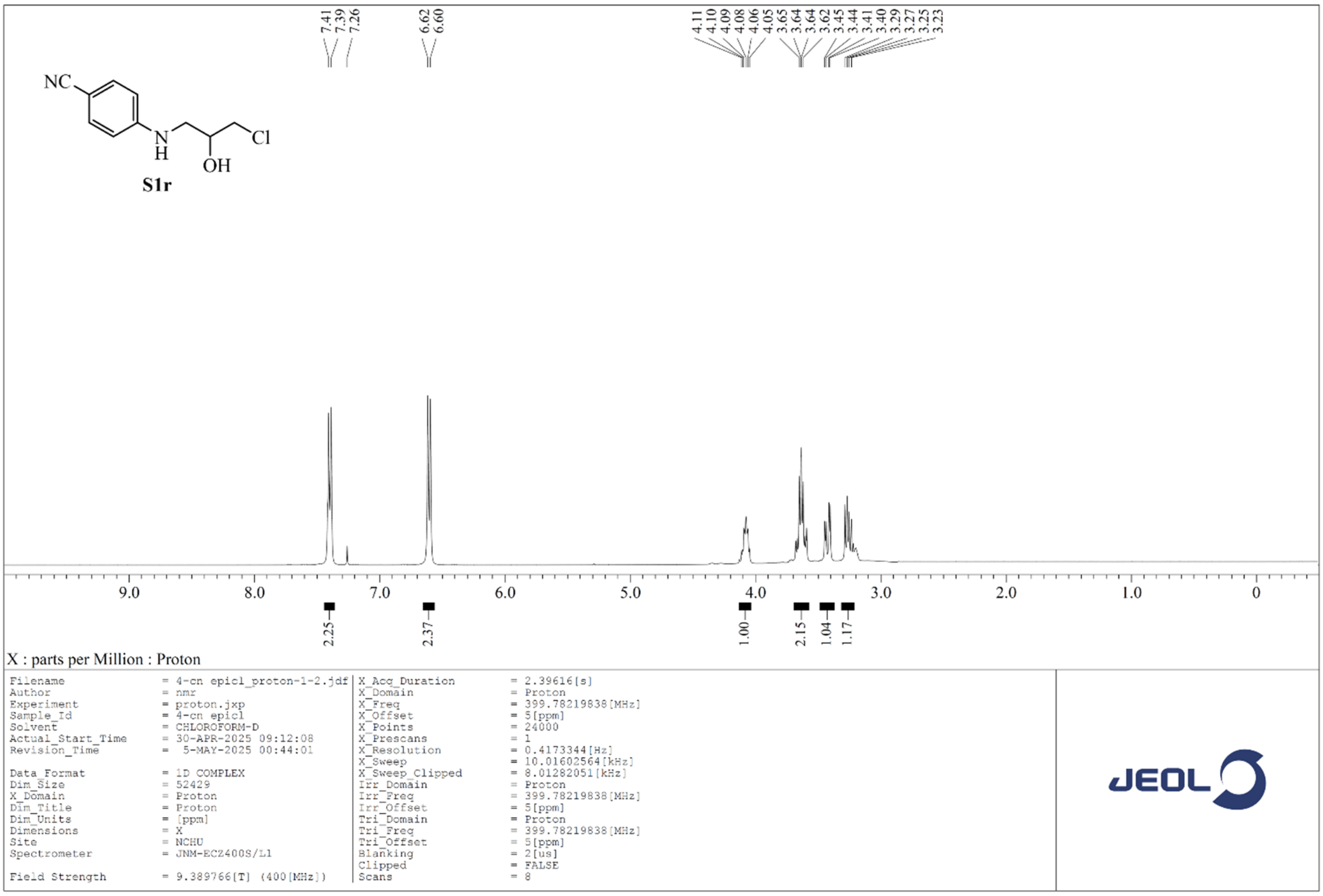


$^{1}$H NMR spectrum of compound **S1r** (400 MHz, $CDCl_3$)

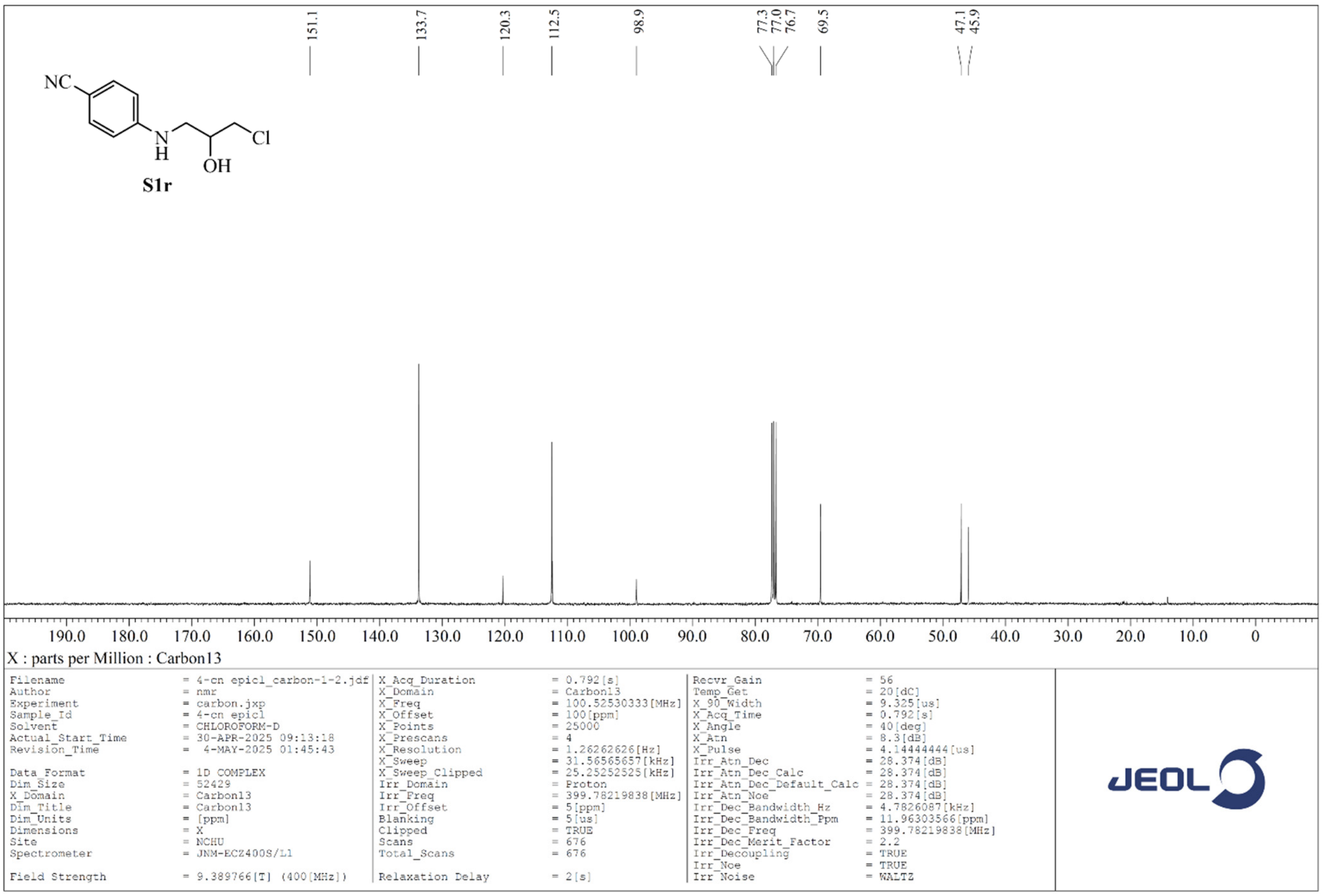


$^{13}$C NMR spectrum of compound **S1r** (101 MHz, $CDCl_3$)

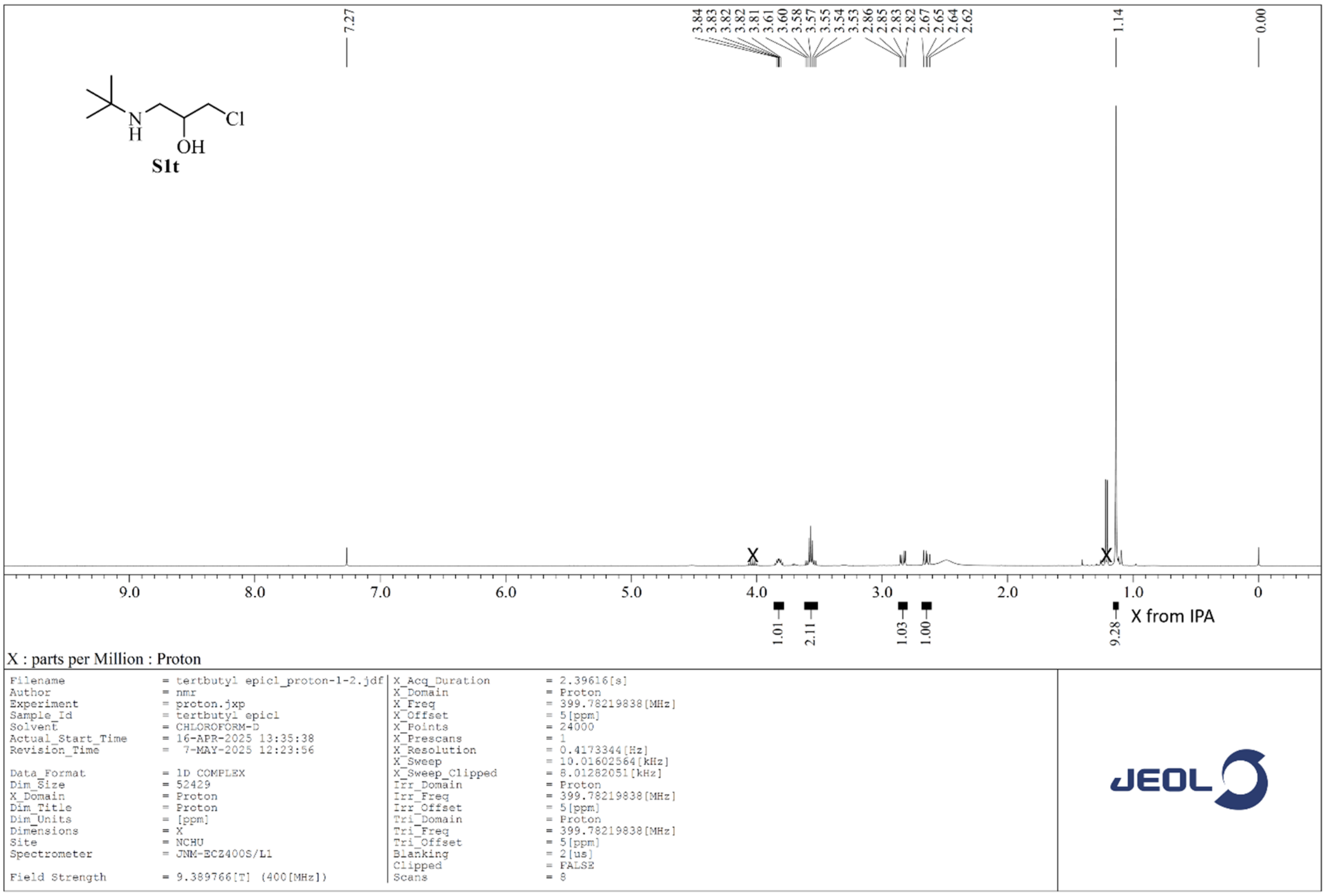


$^{1}$H NMR spectrum of compound **S1t** (400 MHz, $CDCl_3$)

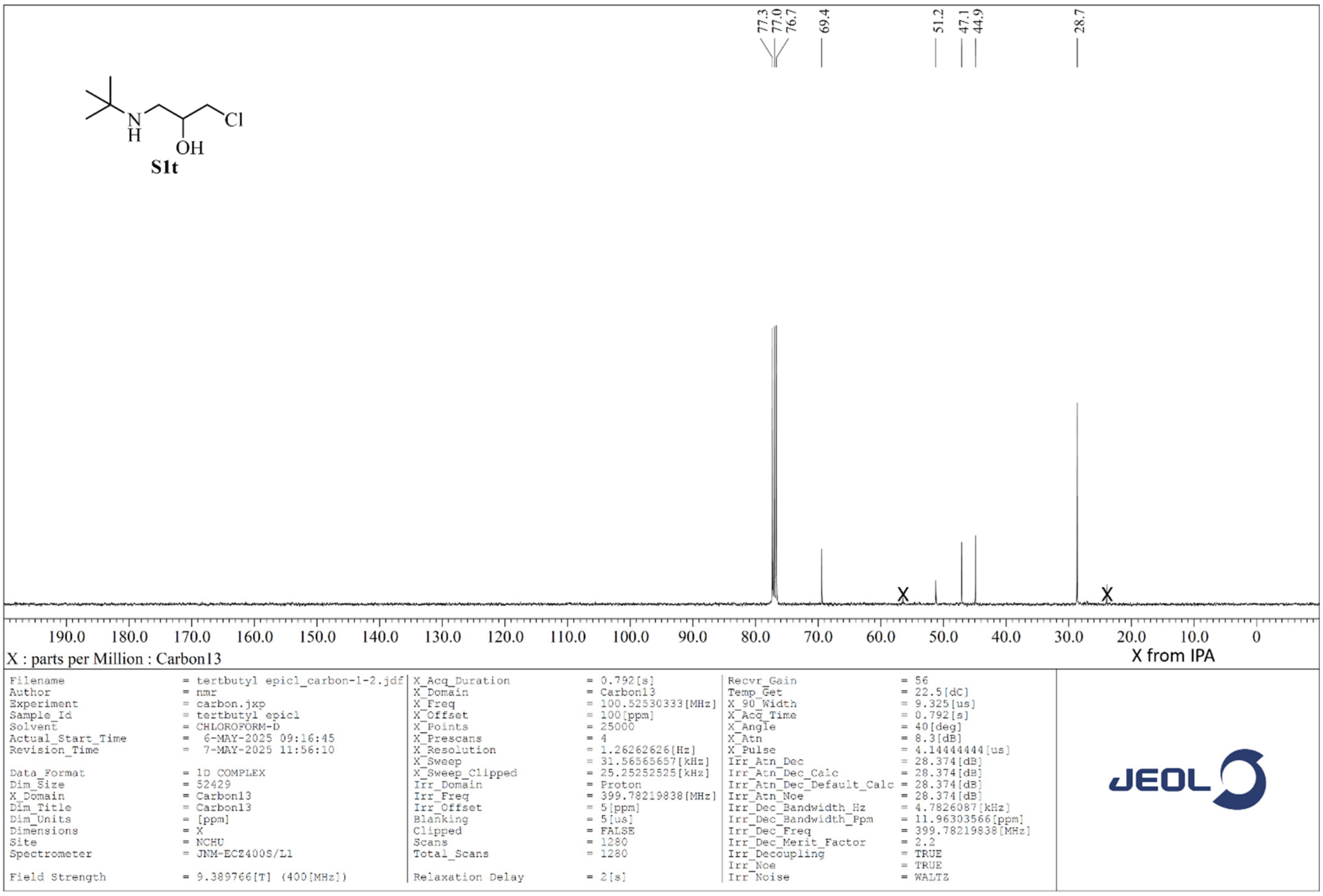


$^{13}$C NMR spectrum of compound **S1t** (101 MHz, $CDCl_3$)

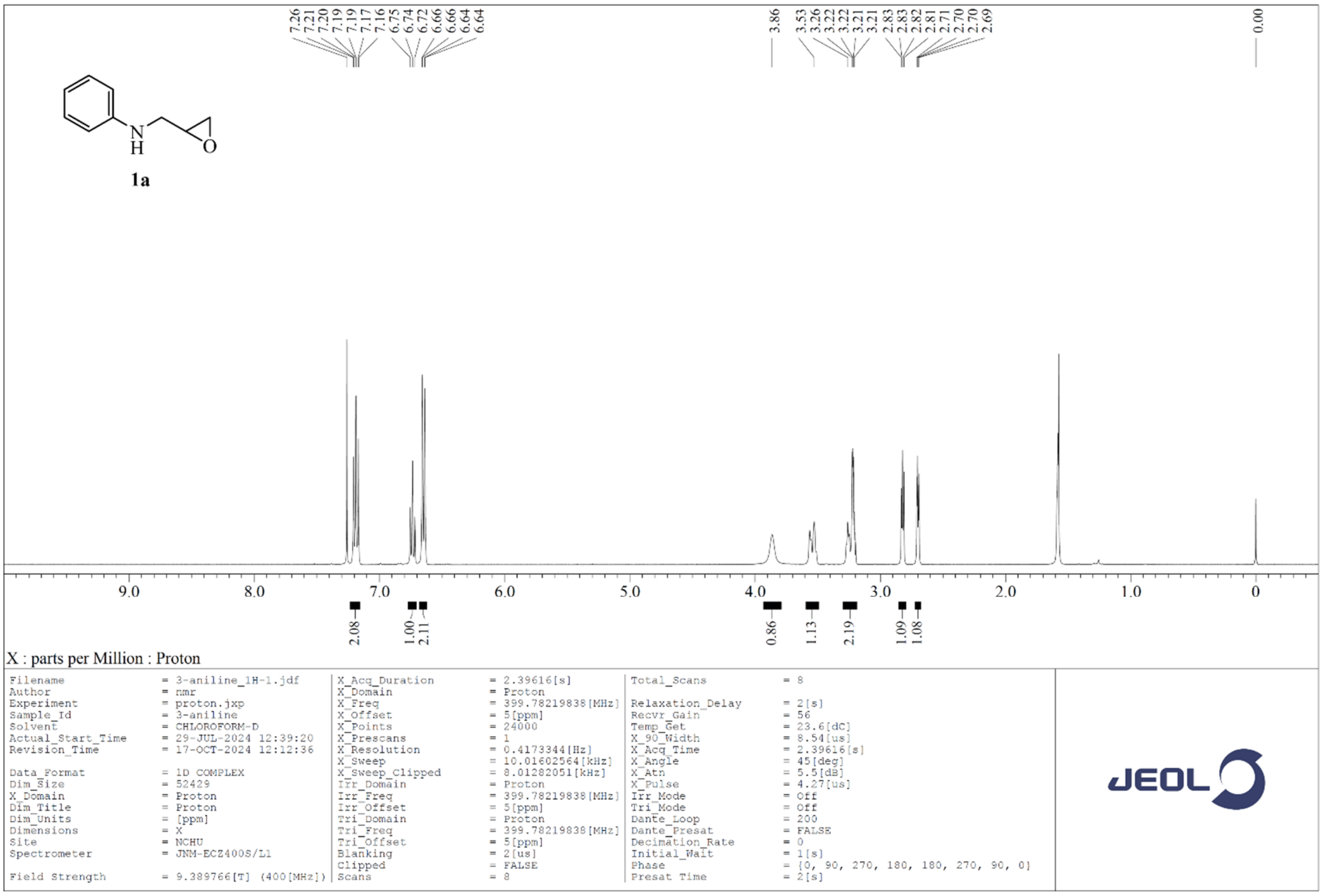


$^{1}$H NMR spectrum of compound **1a** (400 MHz, $CDCl_3$)

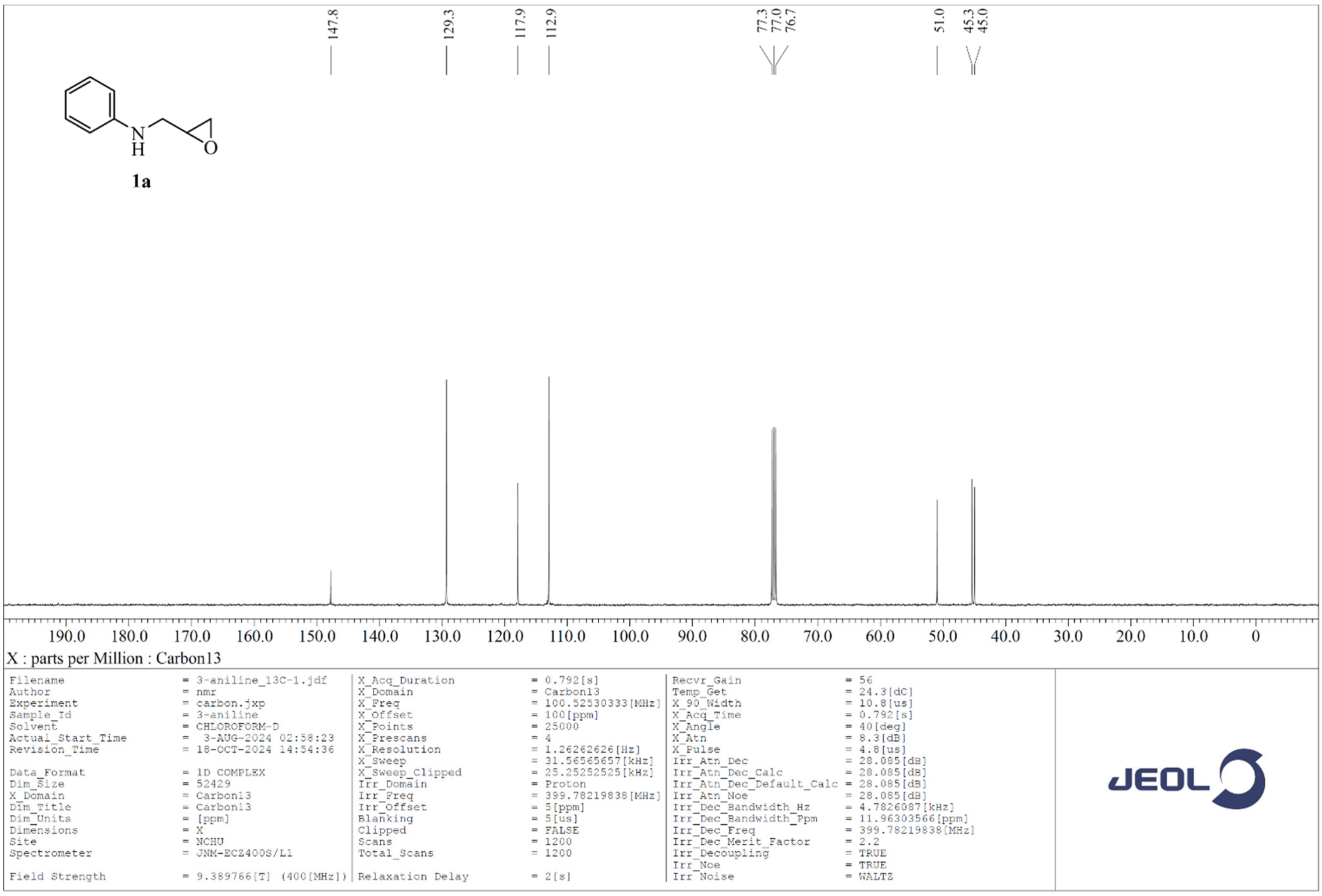


$^{13}$C NMR spectrum of compound **1a** (101 MHz, $CDCl_3$)

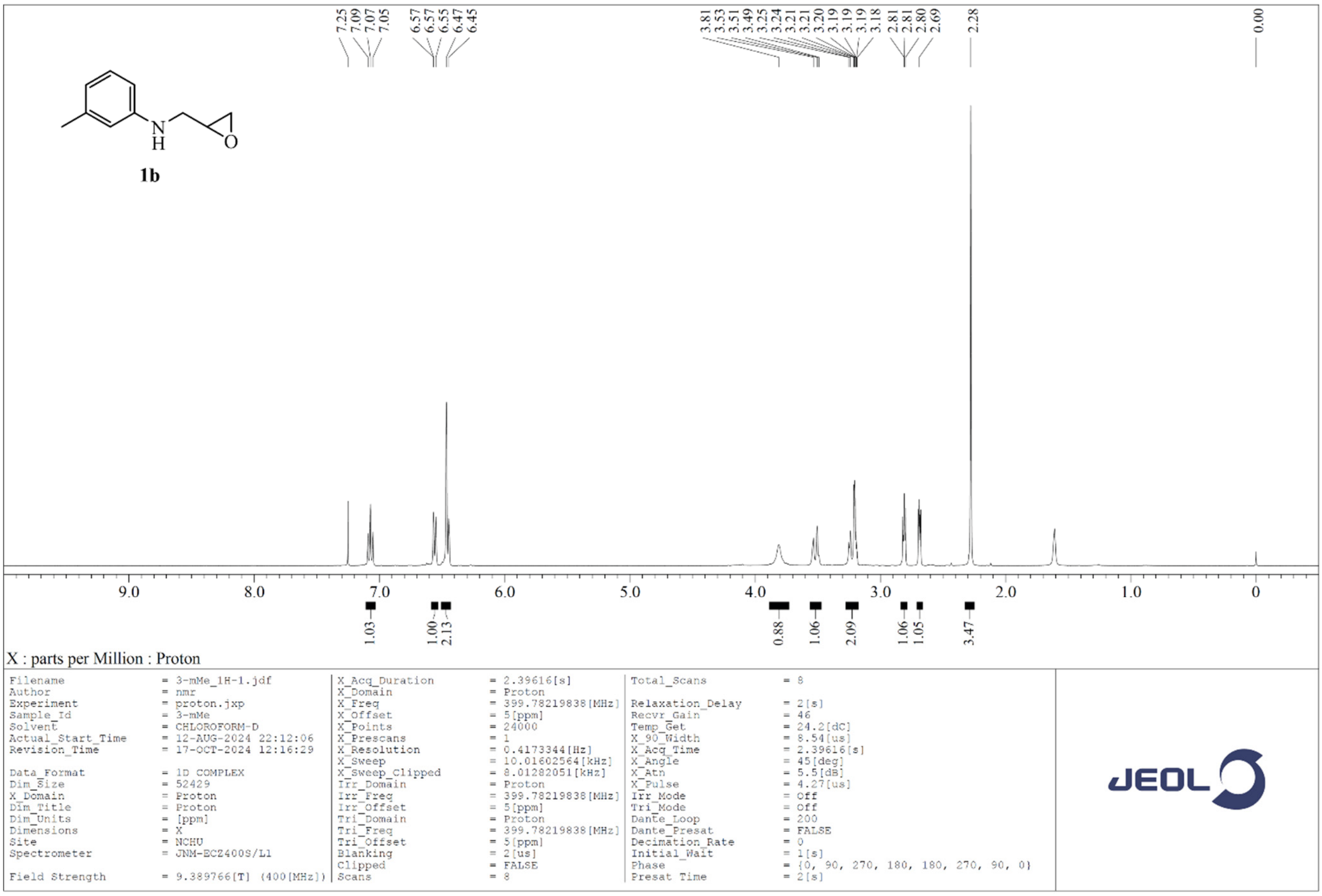


$^{1}$H NMR spectrum of compound **1b** (400 MHz, $CDCl_3$)

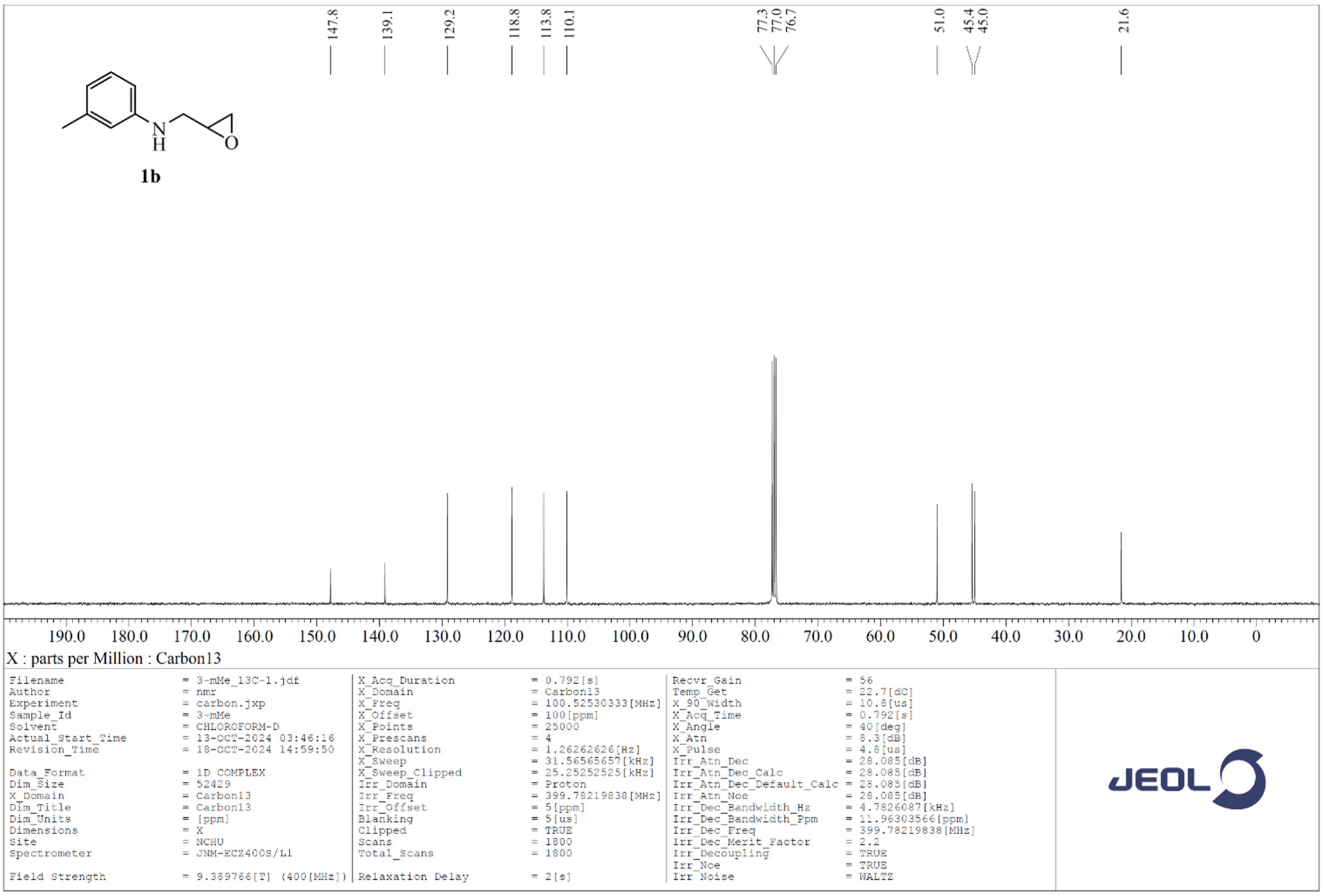


$^{13}$C NMR spectrum of compound **1b** (101 MHz, $CDCl_3$)

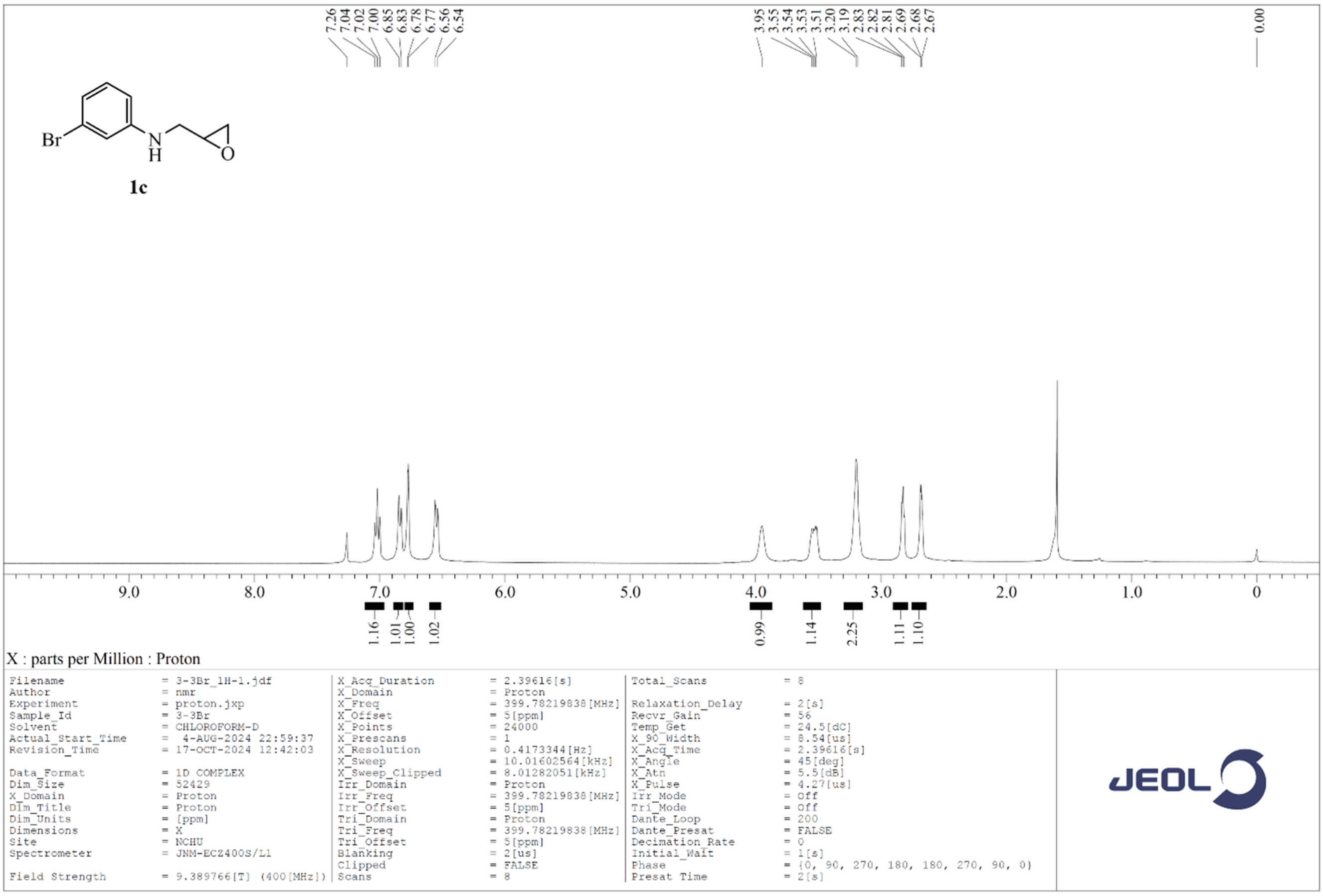


$^{1}$H NMR spectrum of compound **1c** (400 MHz, $CDCl_3$)

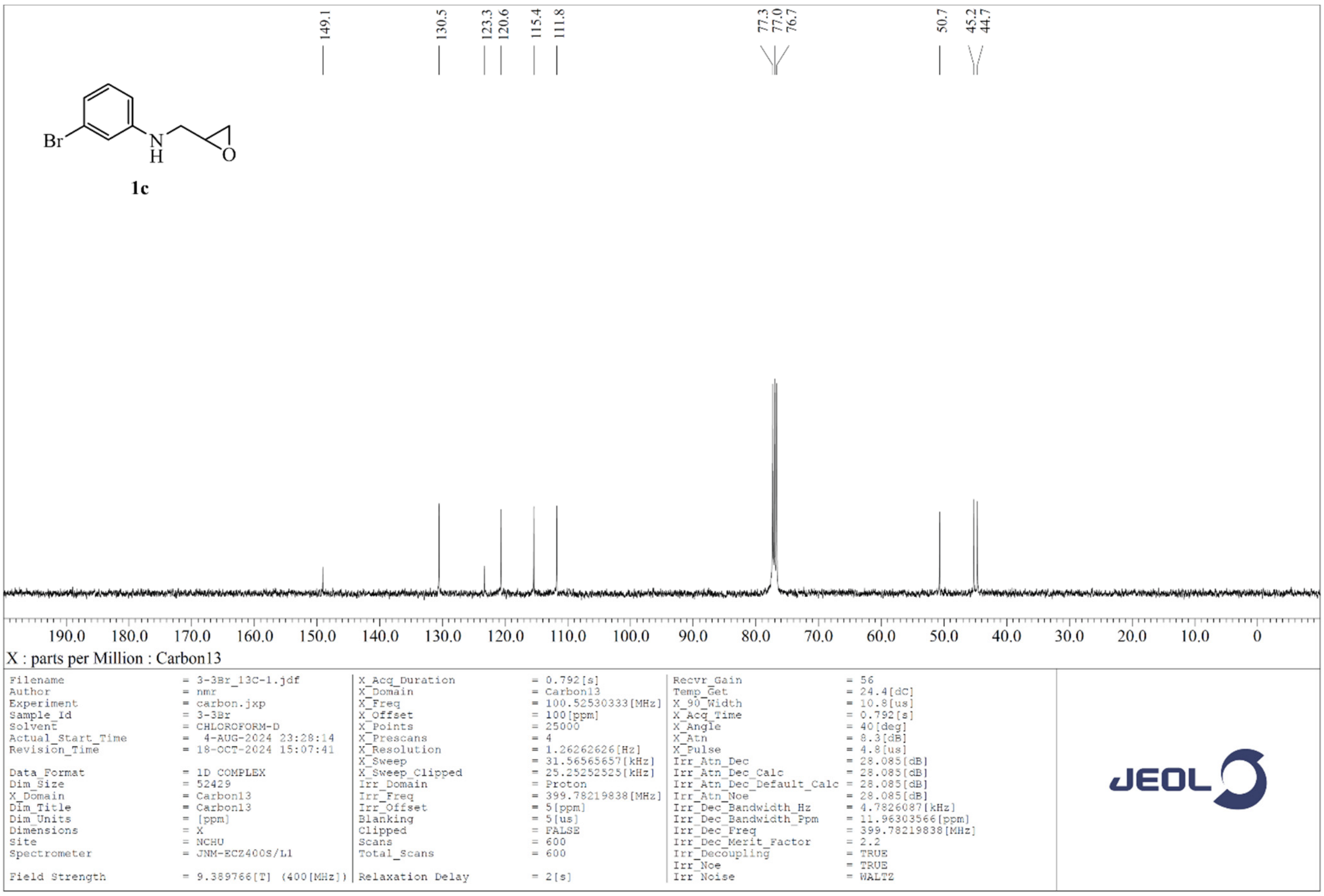


$^{13}$C NMR spectrum of compound **1c** (101 MHz, $CDCl_3$)

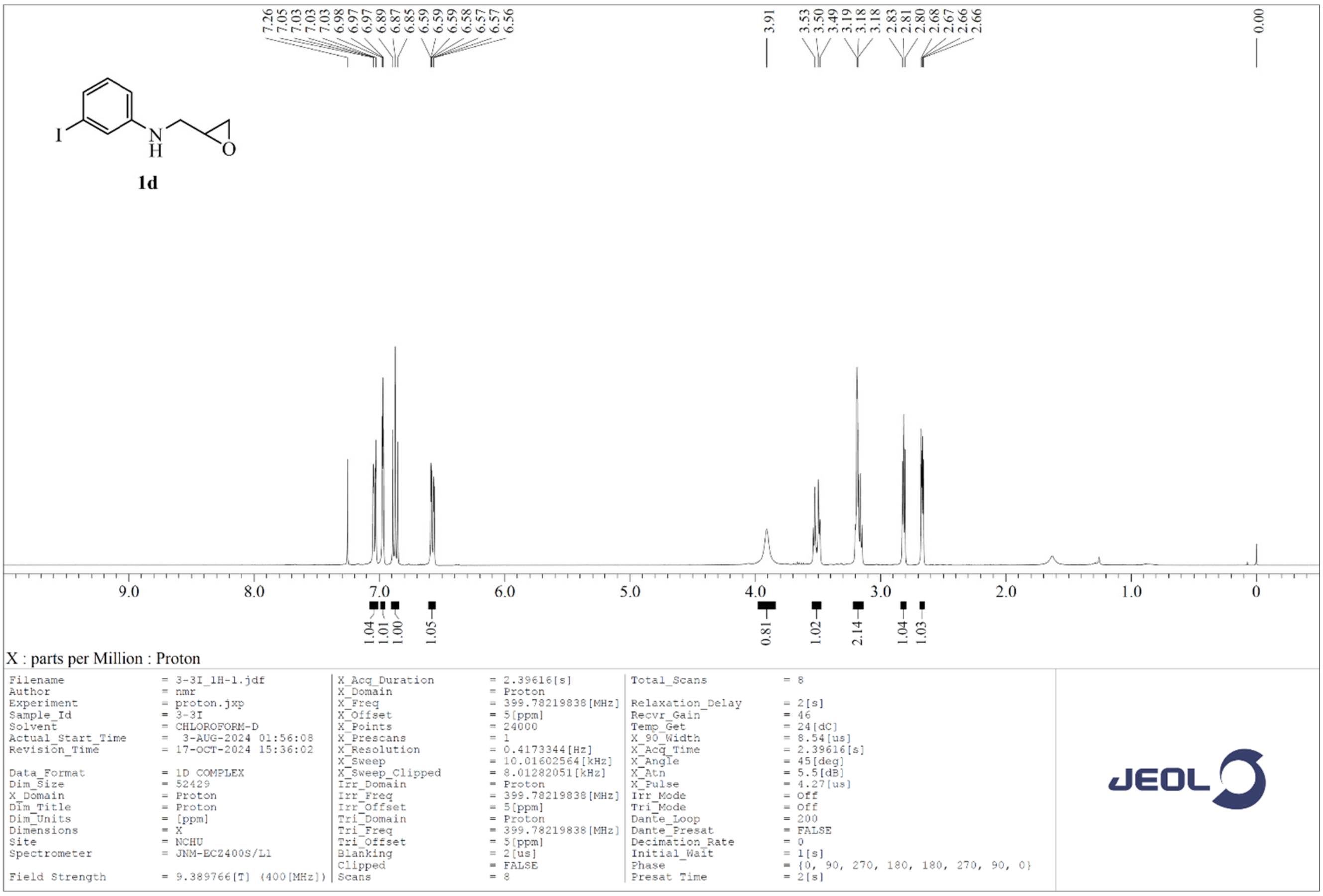


$^{1}H$ NMR spectrum of compound **1d** (400 MHz, $CDCl_3$)

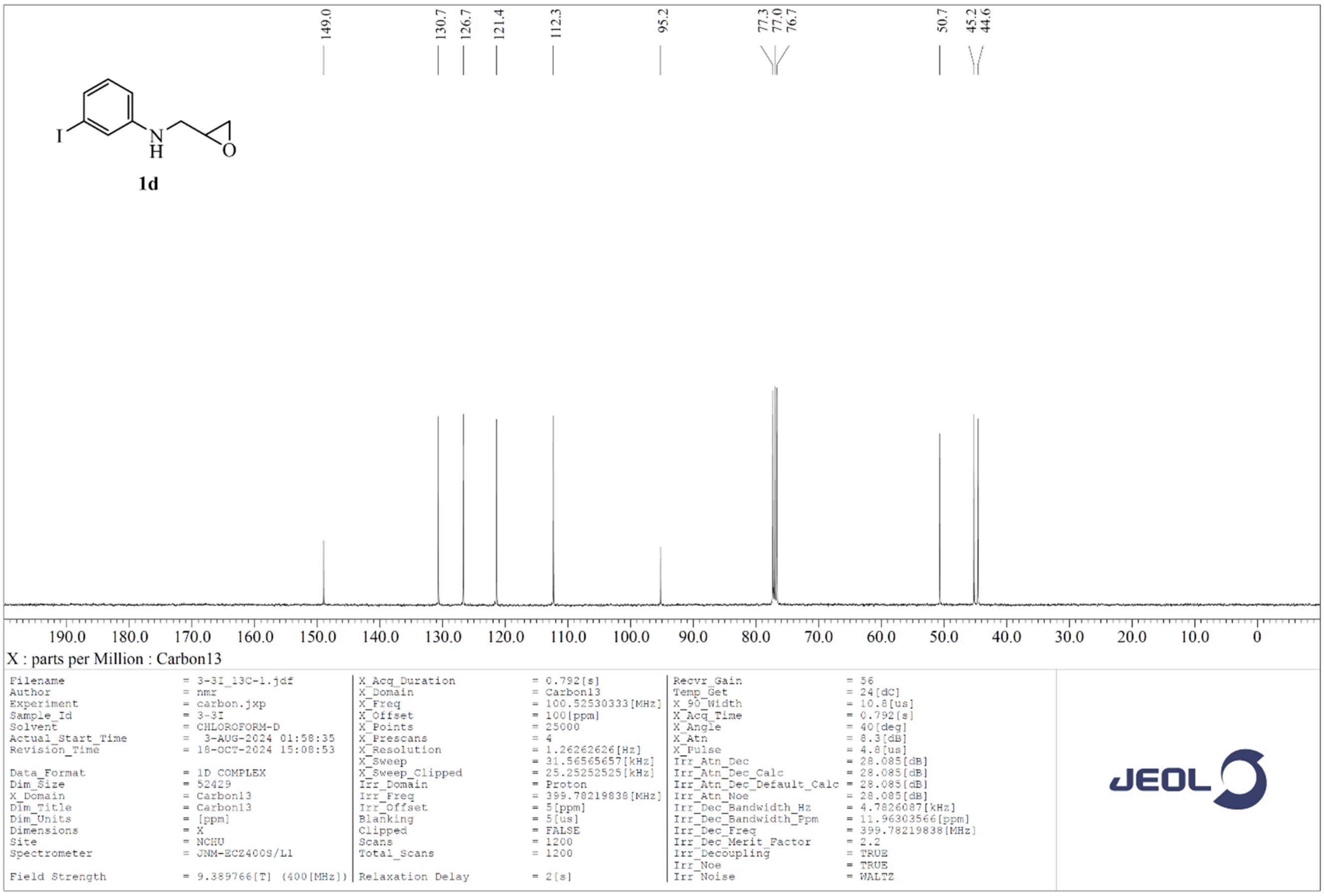


$^{13}C$ NMR spectrum of compound **1d** (101 MHz, $CDCl_3$)

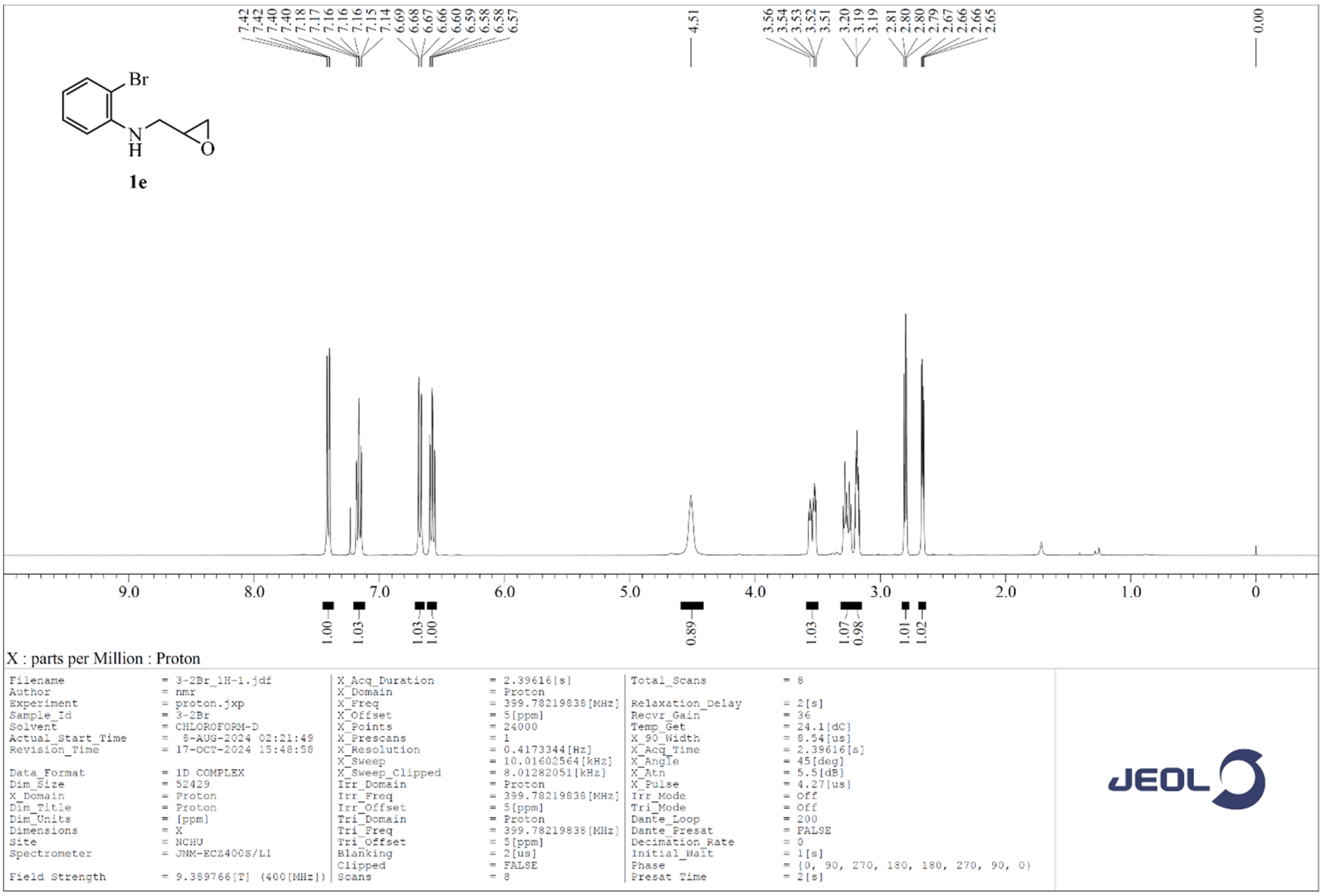


$^{1}$H NMR spectrum of compound **1e** (400 MHz, $CDCl_3$)

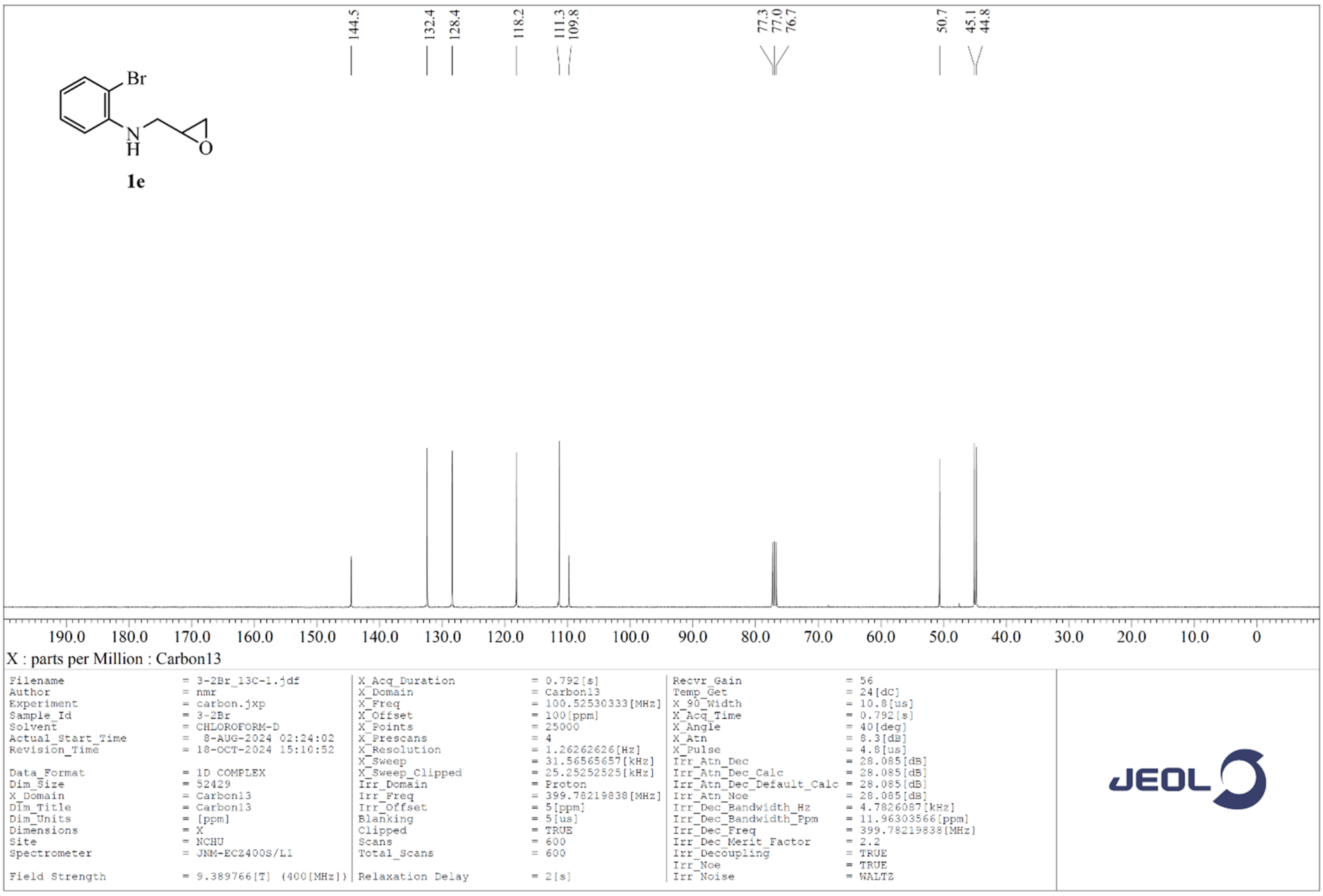


$^{13}$C NMR spectrum of compound **1e** (101 MHz, $CDCl_3$)

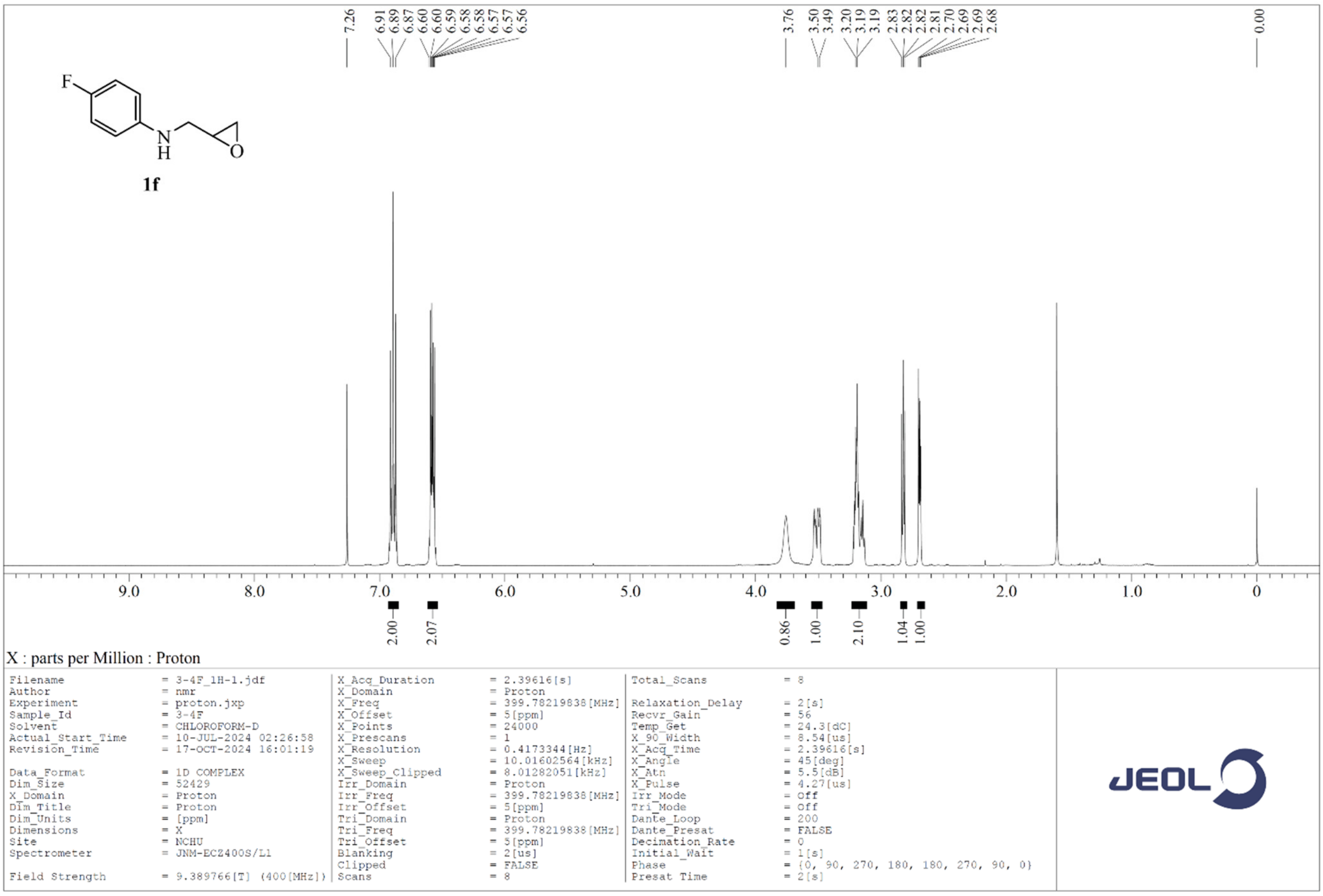


$^{1}H$ NMR spectrum of compound **1f** (400 MHz, $CDCl_3$)

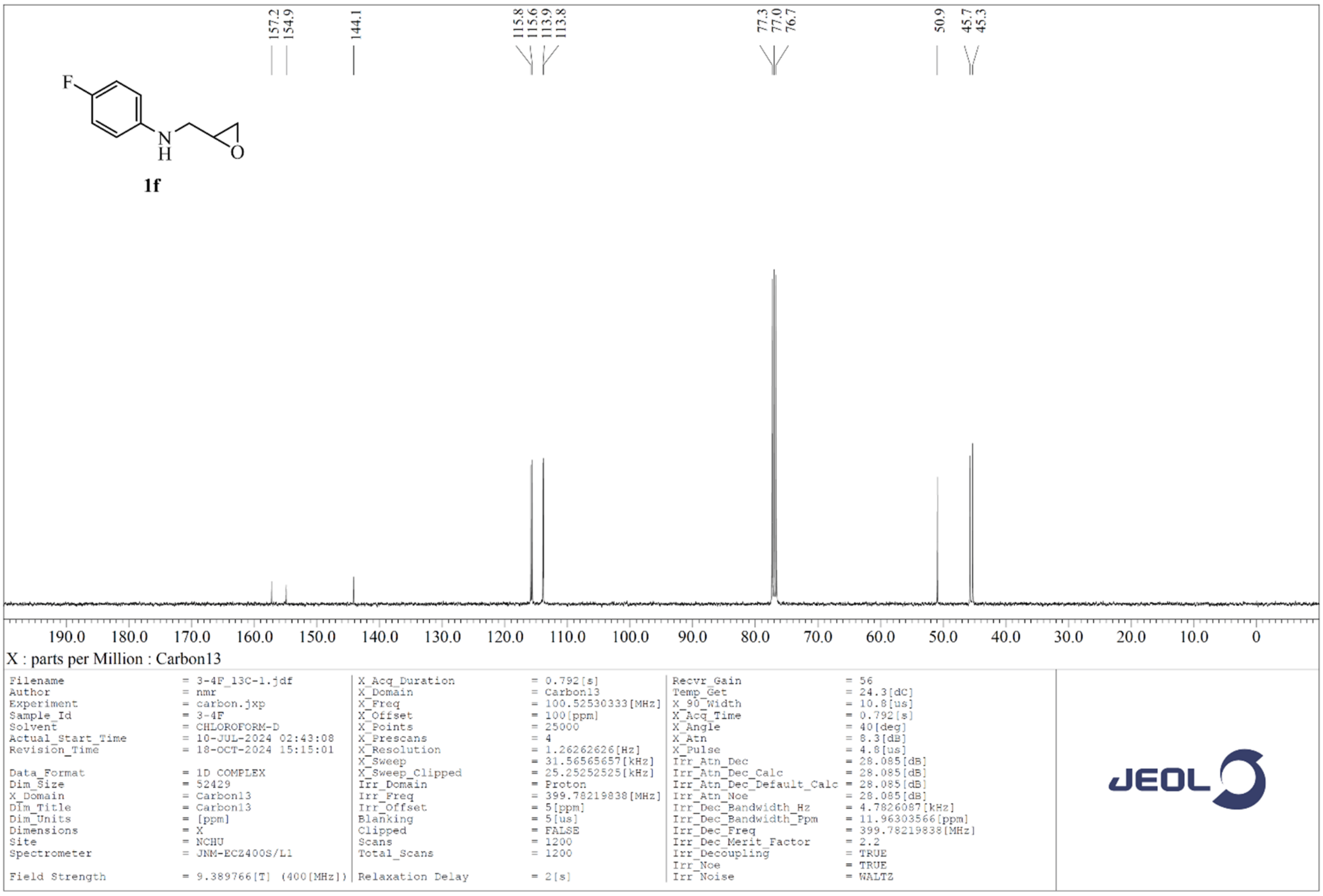


$^{13}C$ NMR spectrum of compound **1f** (101 MHz, $CDCl_3$)

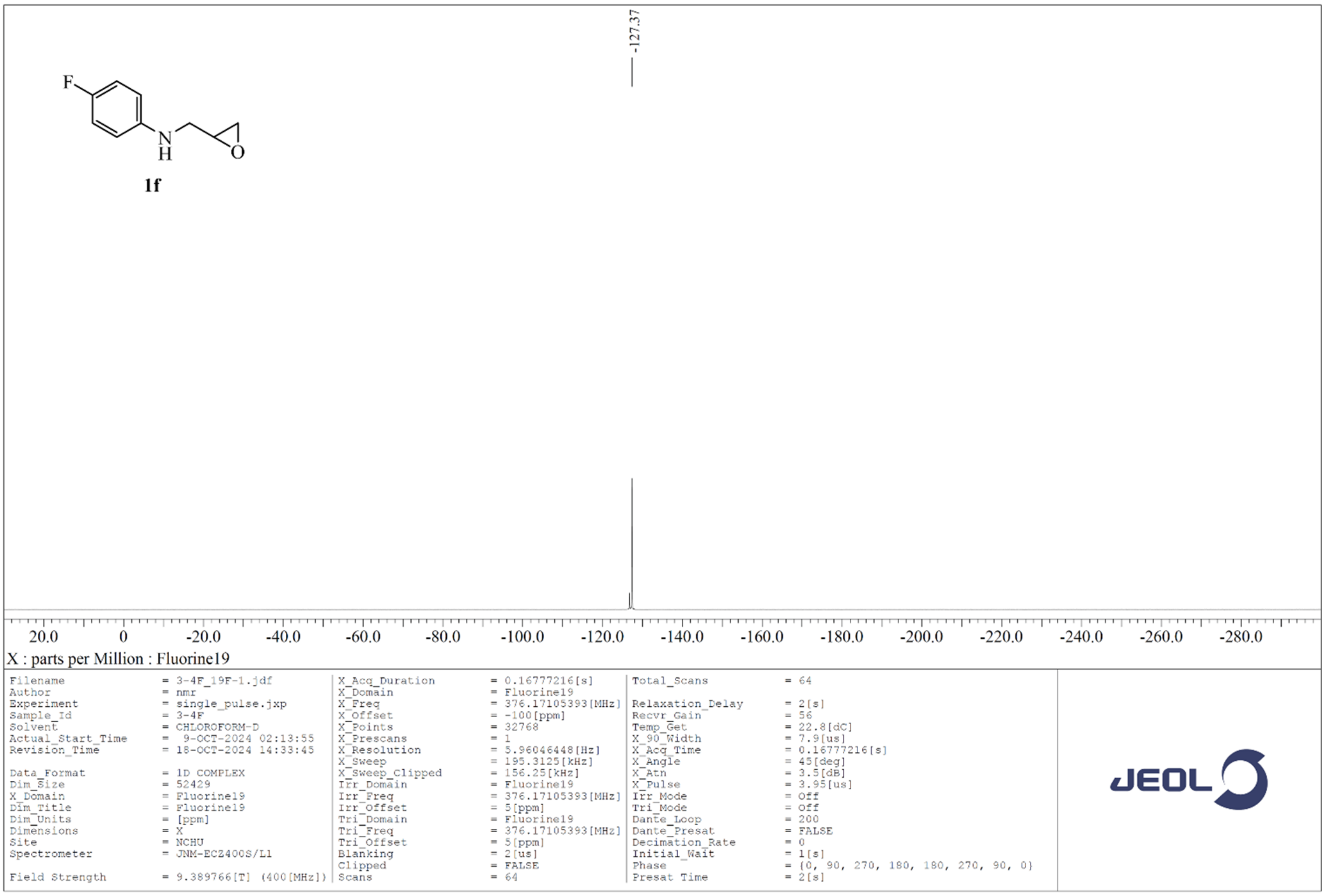


$^{19}$F NMR spectrum of compound **1f** (376 MHz, $CDCl_3$)

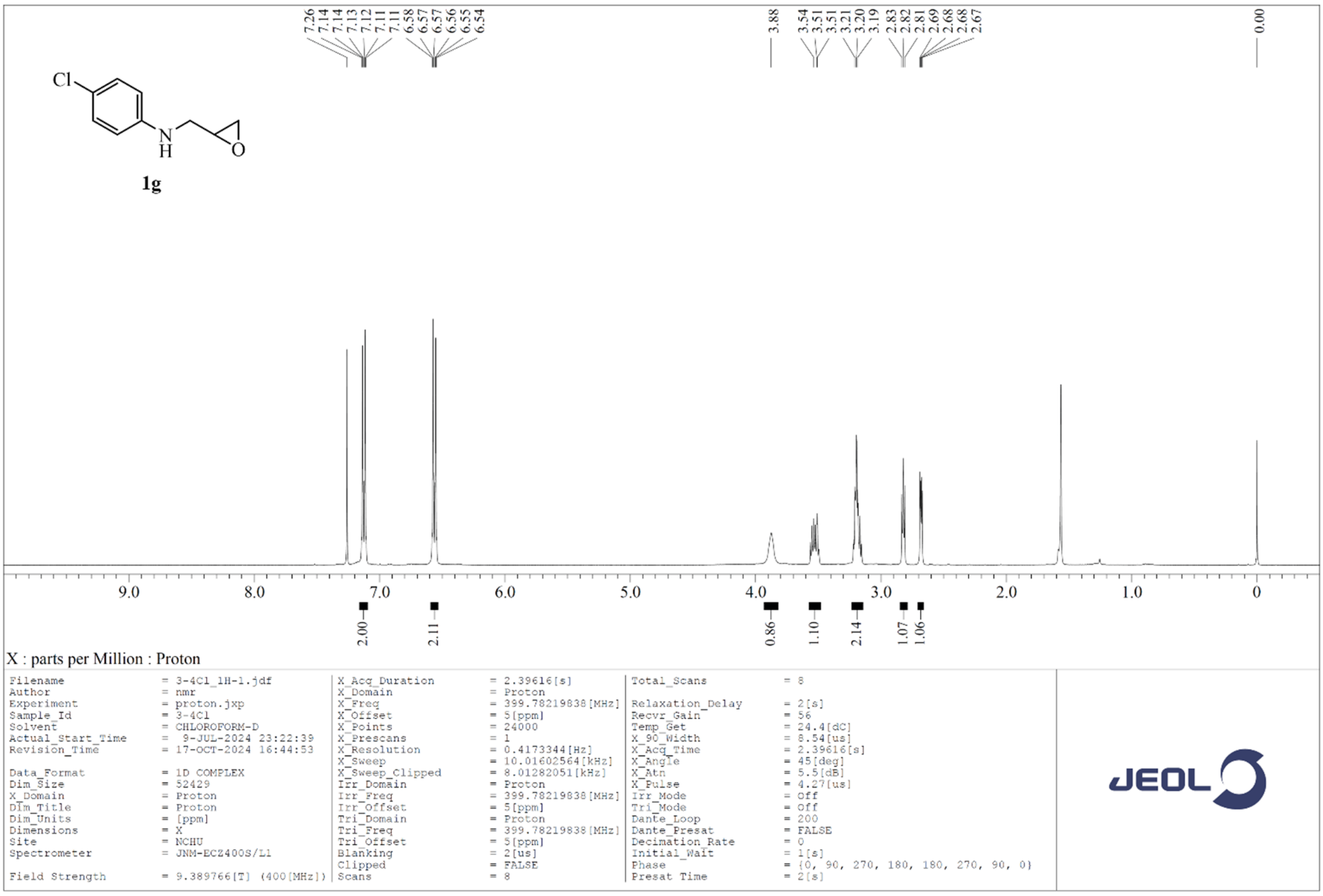


$^{1}$H NMR spectrum of compound **1g** (400 MHz, $CDCl_3$)

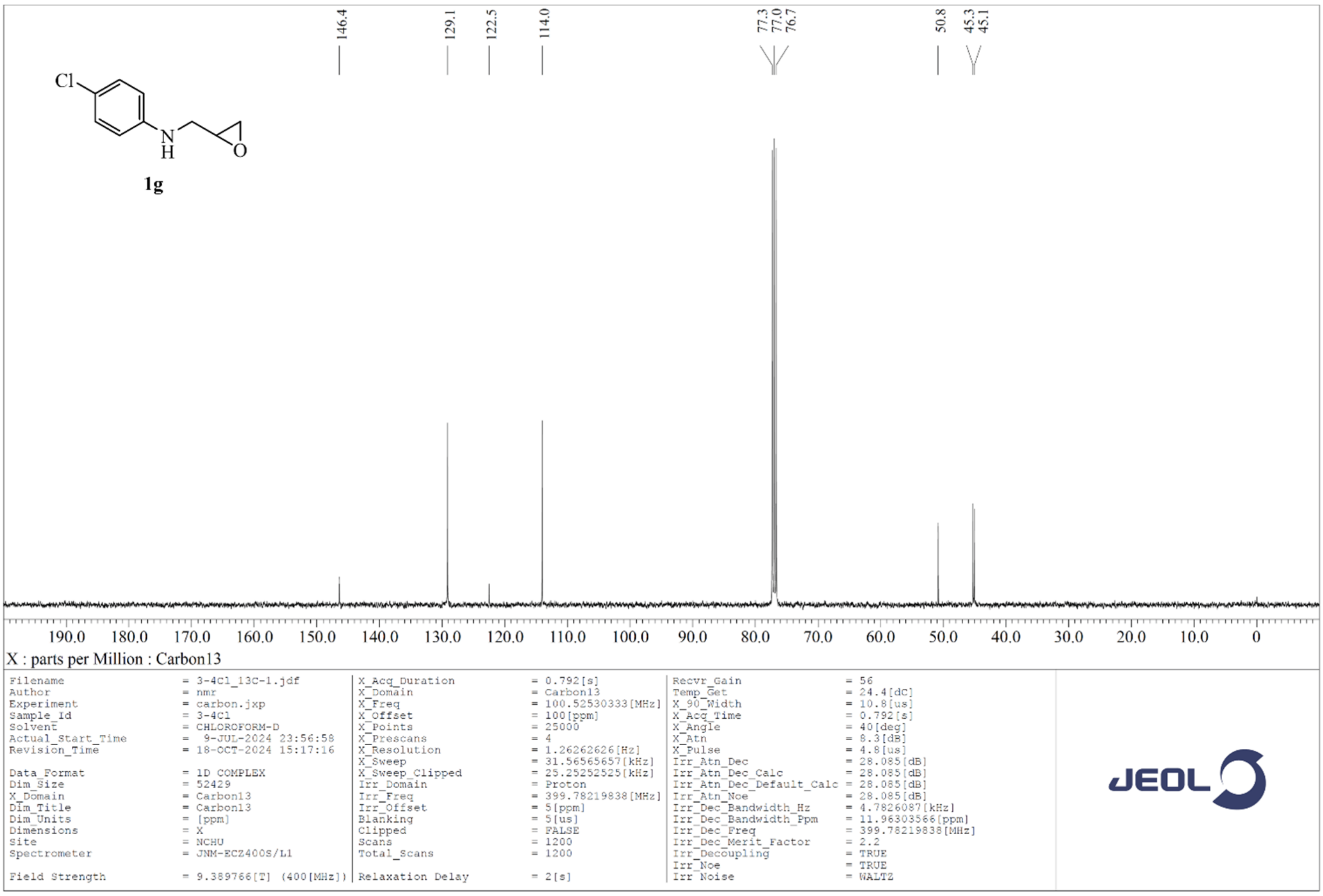


$^{13}C$ NMR spectrum of compound **1g** (101 MHz, $CDCl_3$)

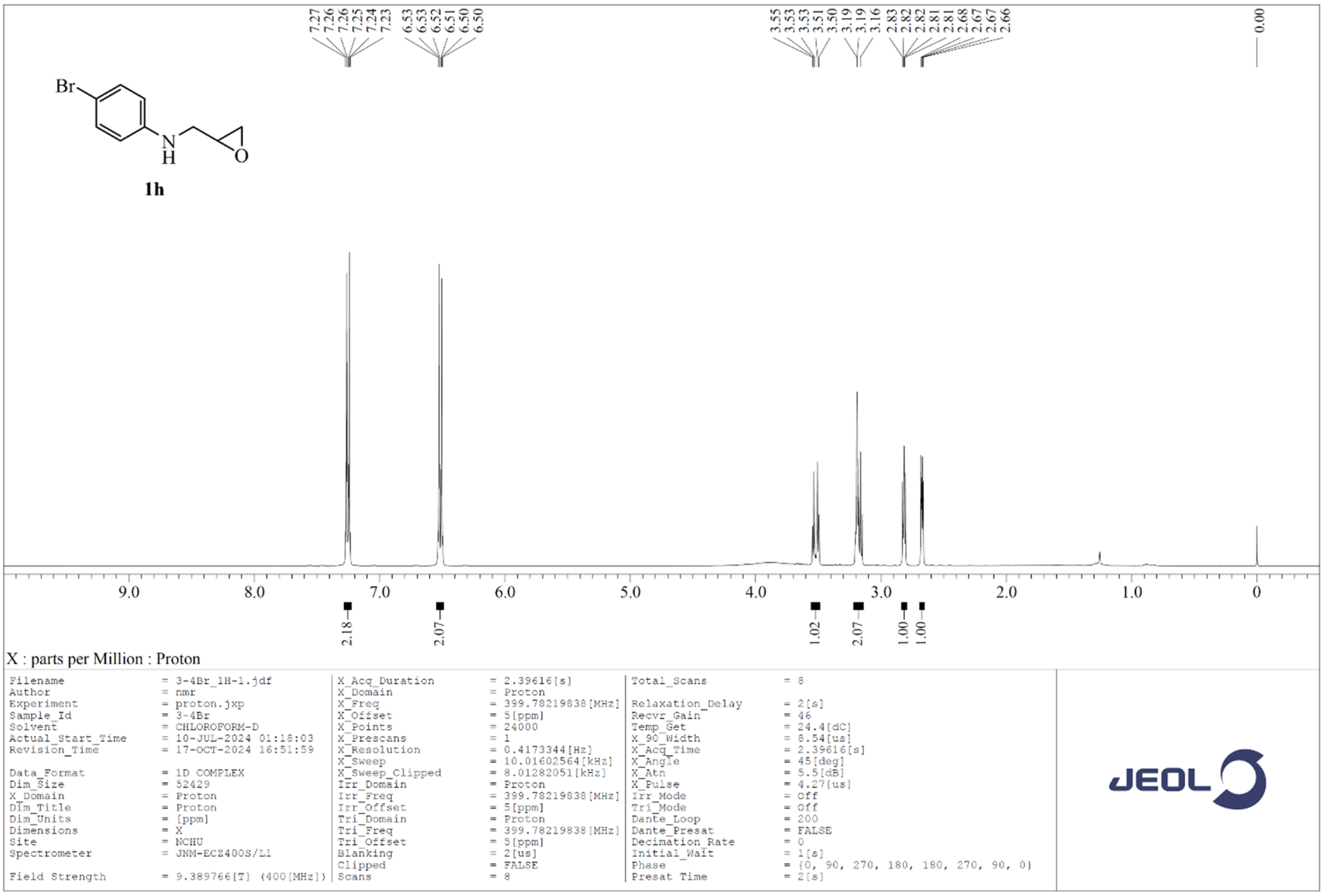


$^{1}H$ NMR spectrum of compound **1h** (400 MHz, $CDCl_3$)

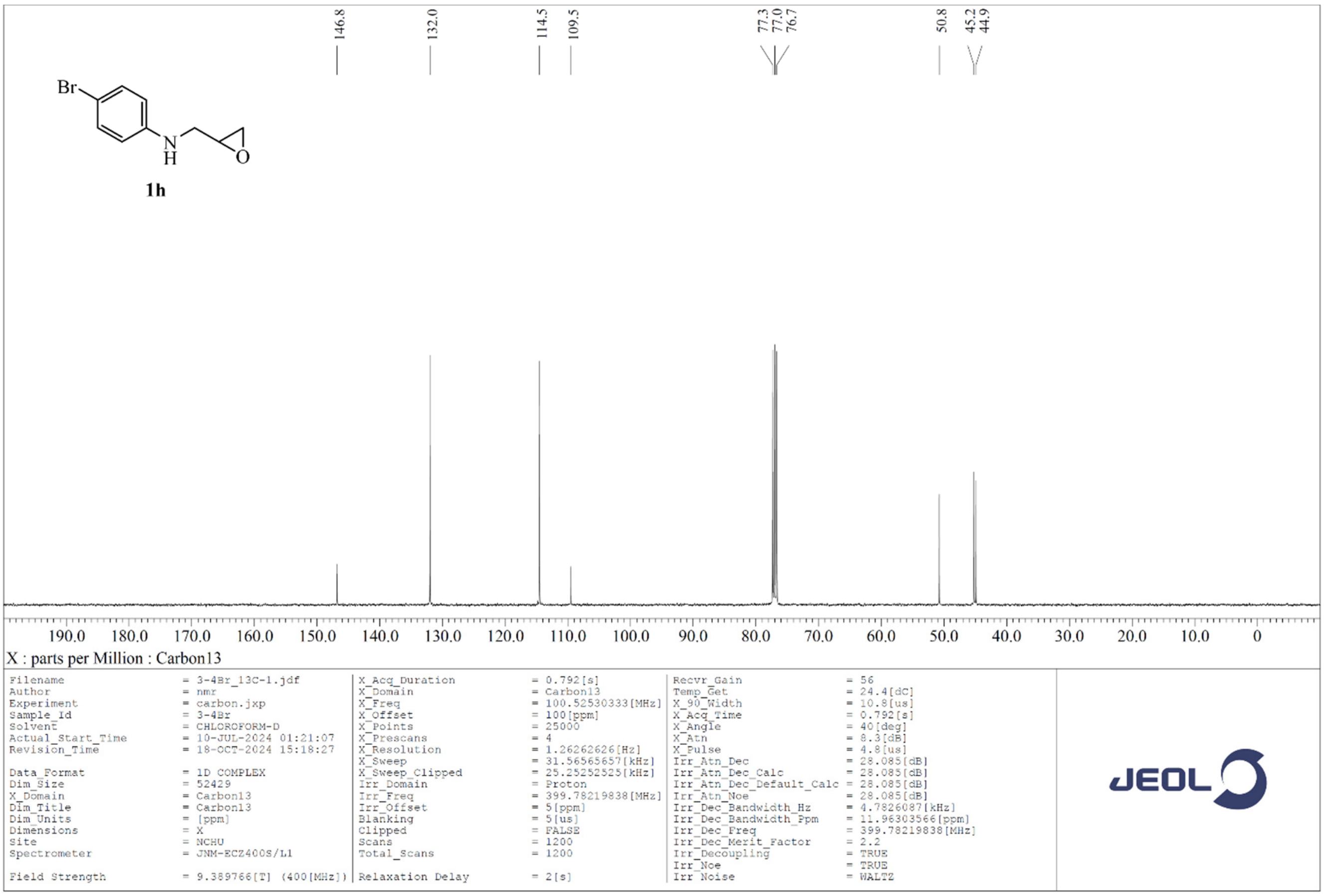


$^{13}$C NMR spectrum of compound **1h** (101 MHz, $CDCl_3$)

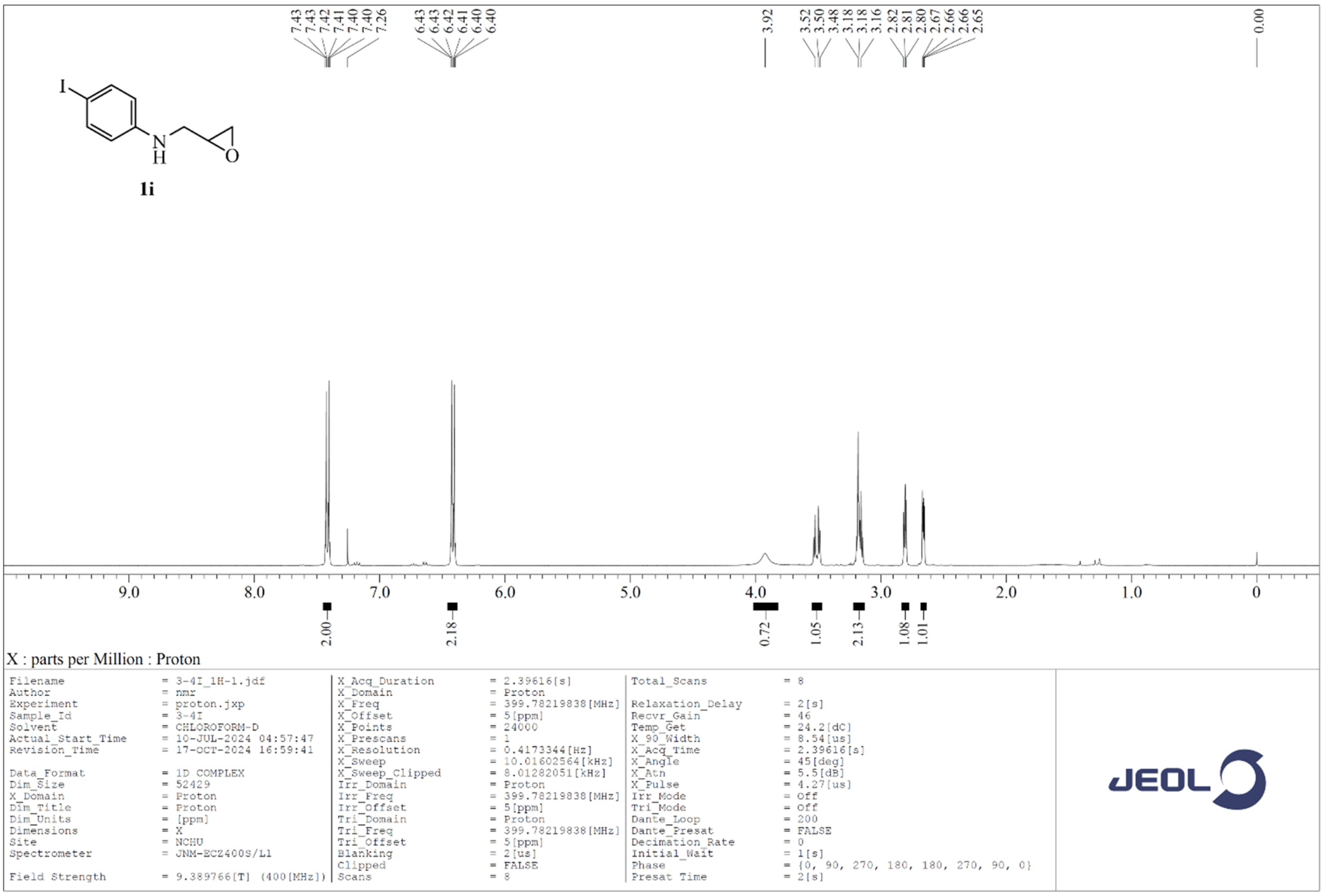


$^{1}$H NMR spectrum of compound **1i** (400 MHz, $CDCl_3$)

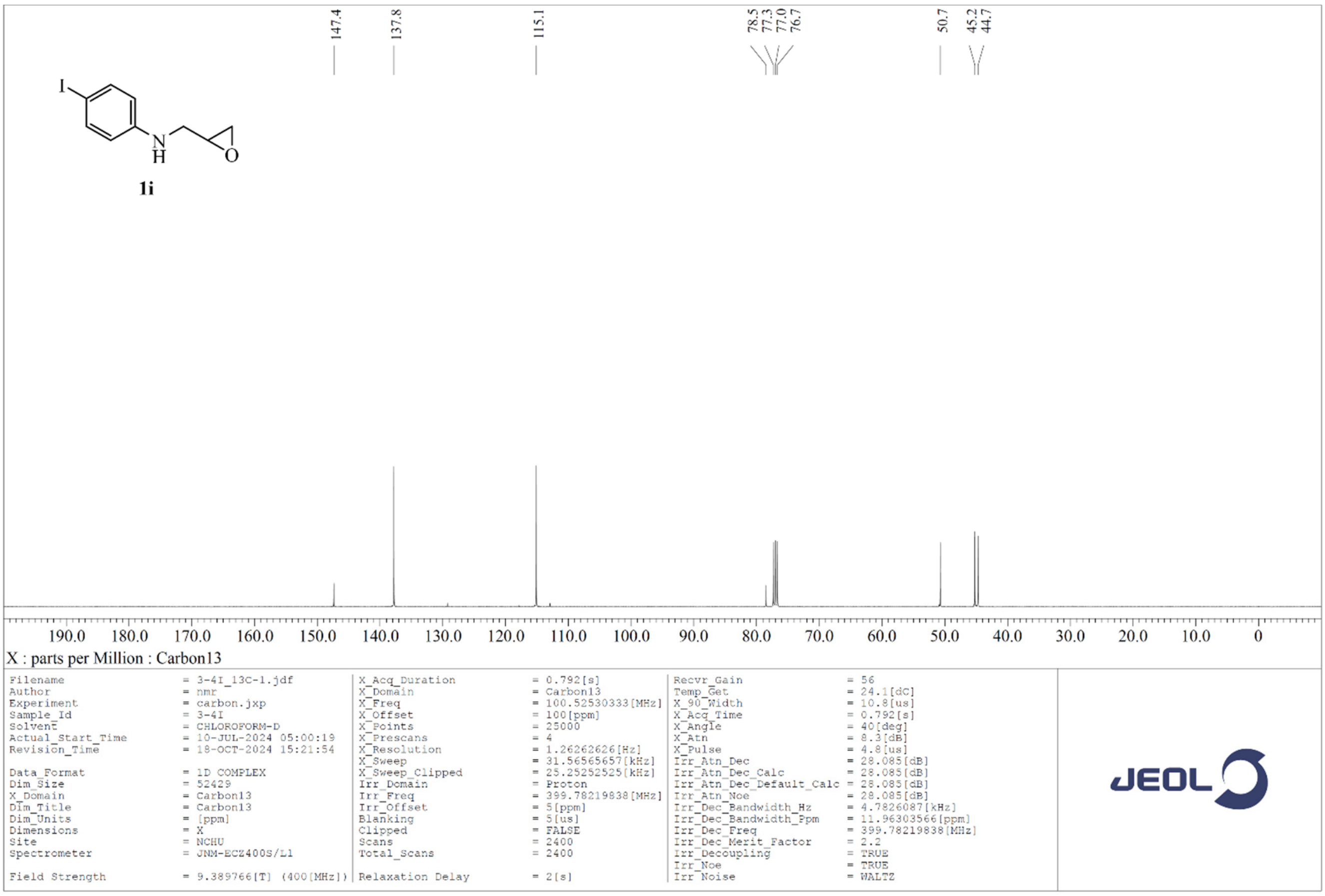


$^{13}$C NMR spectrum of compound **1i** (101 MHz, $CDCl_3$)

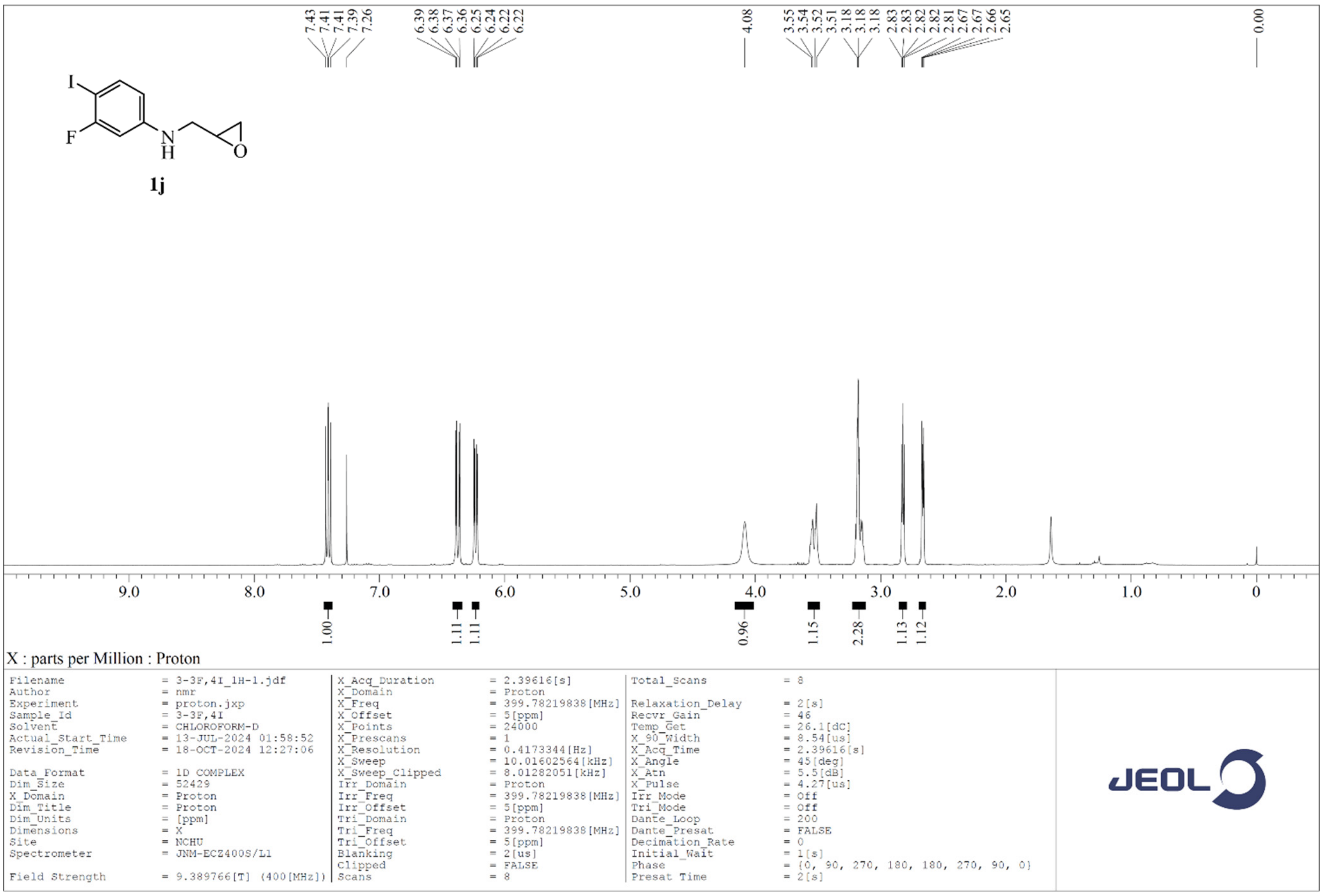


$^{1}$H NMR spectrum of compound **1j** (400 MHz, $CDCl_3$)

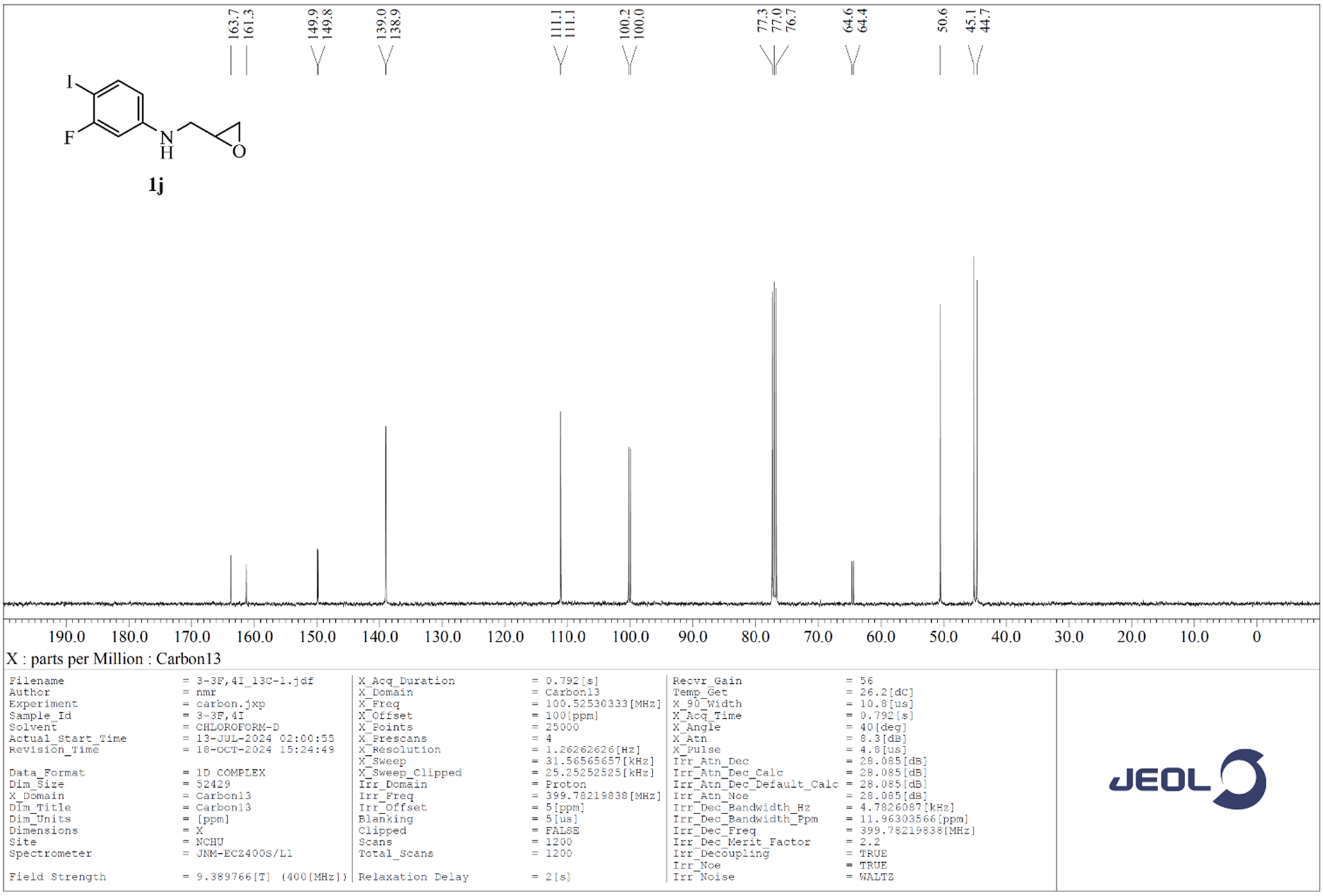


$^{13}C$ NMR spectrum of compound **1j** (101 MHz, $CDCl_3$)

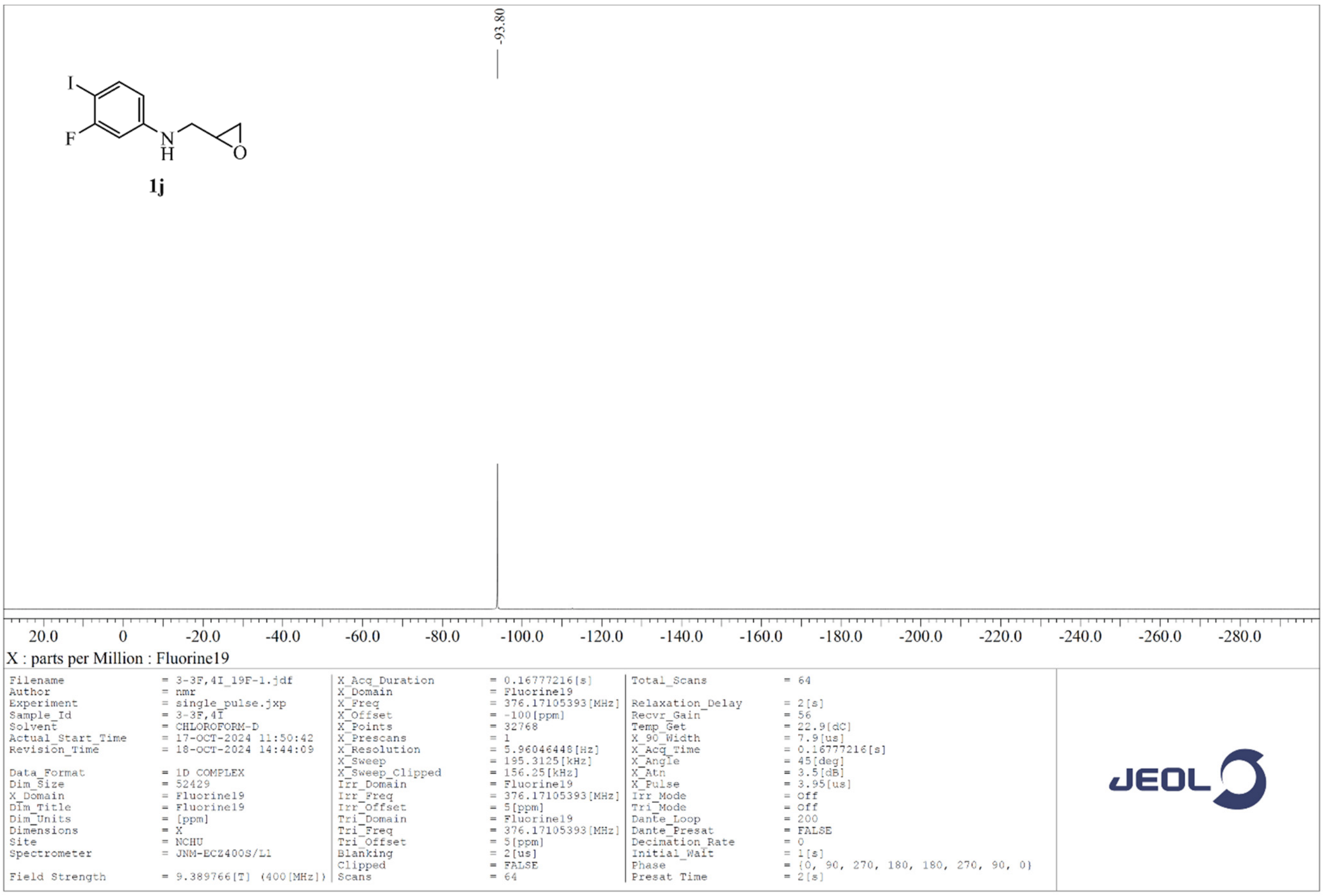


$^{19}F$ NMR spectrum of compound **1j** (376 MHz, $CDCl_3$)

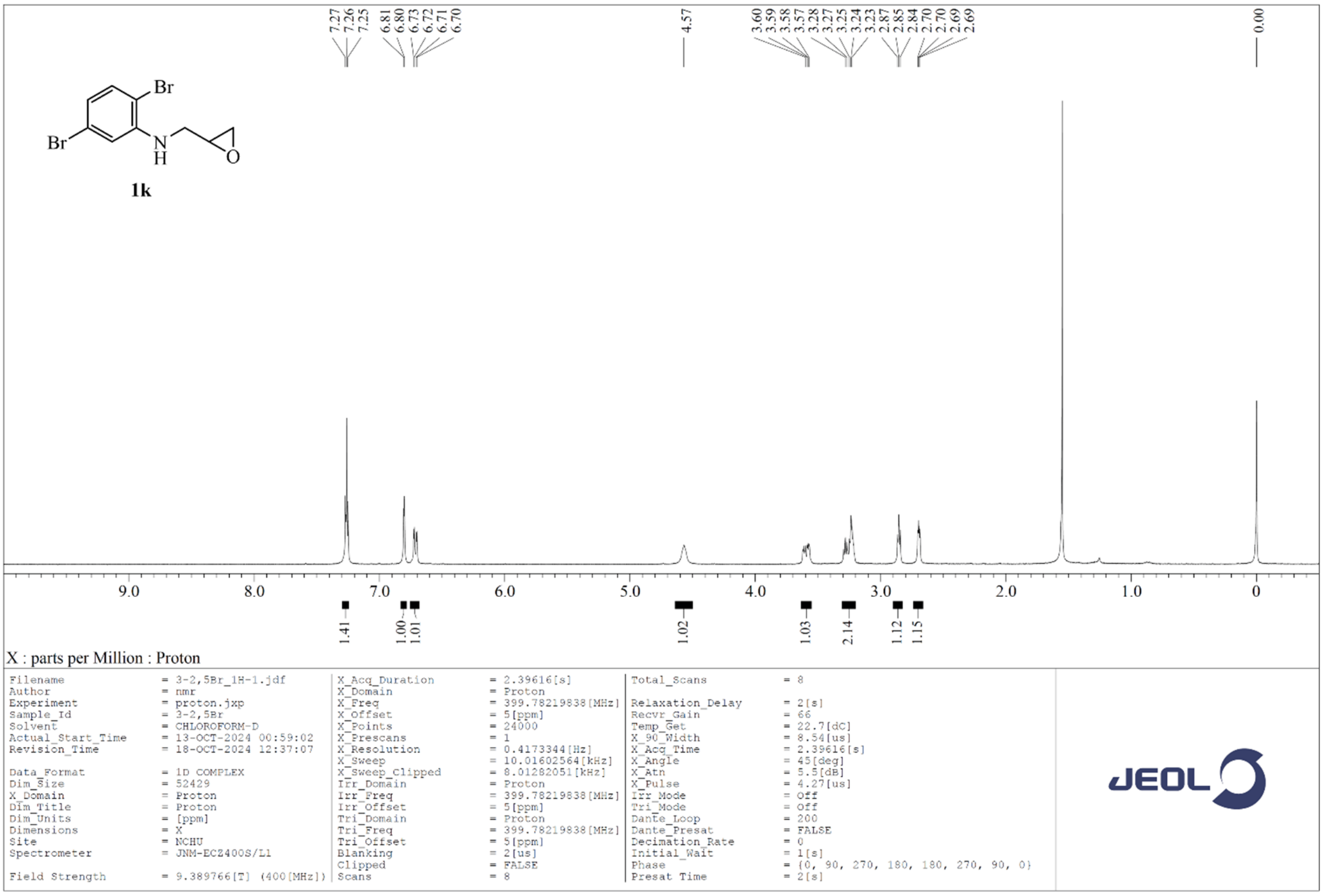


$^{1}$H NMR spectrum of compound **1k** (400 MHz, $CDCl_3$)

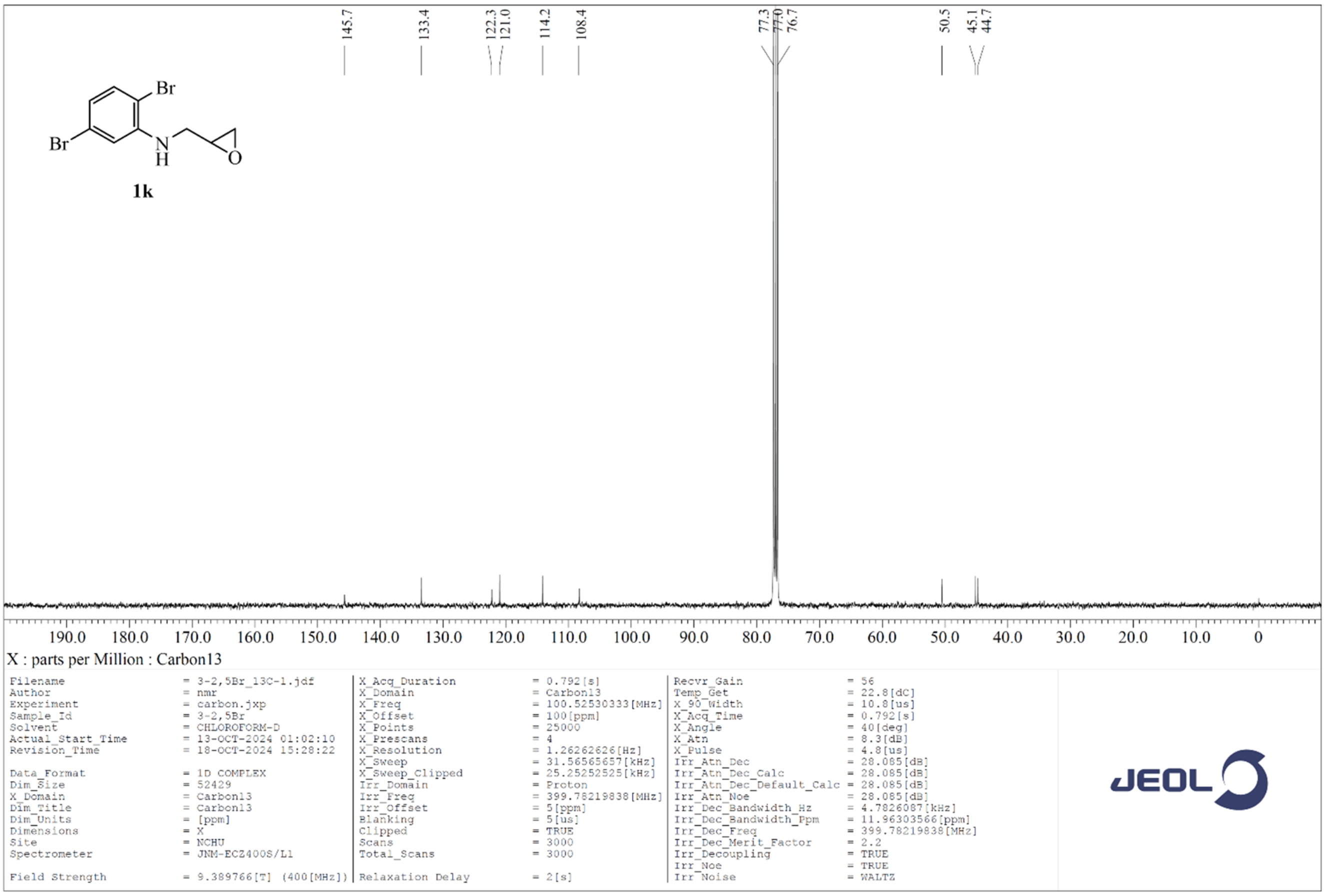


$^{13}$C NMR spectrum of compound **1k** (101 MHz, $CDCl_3$)

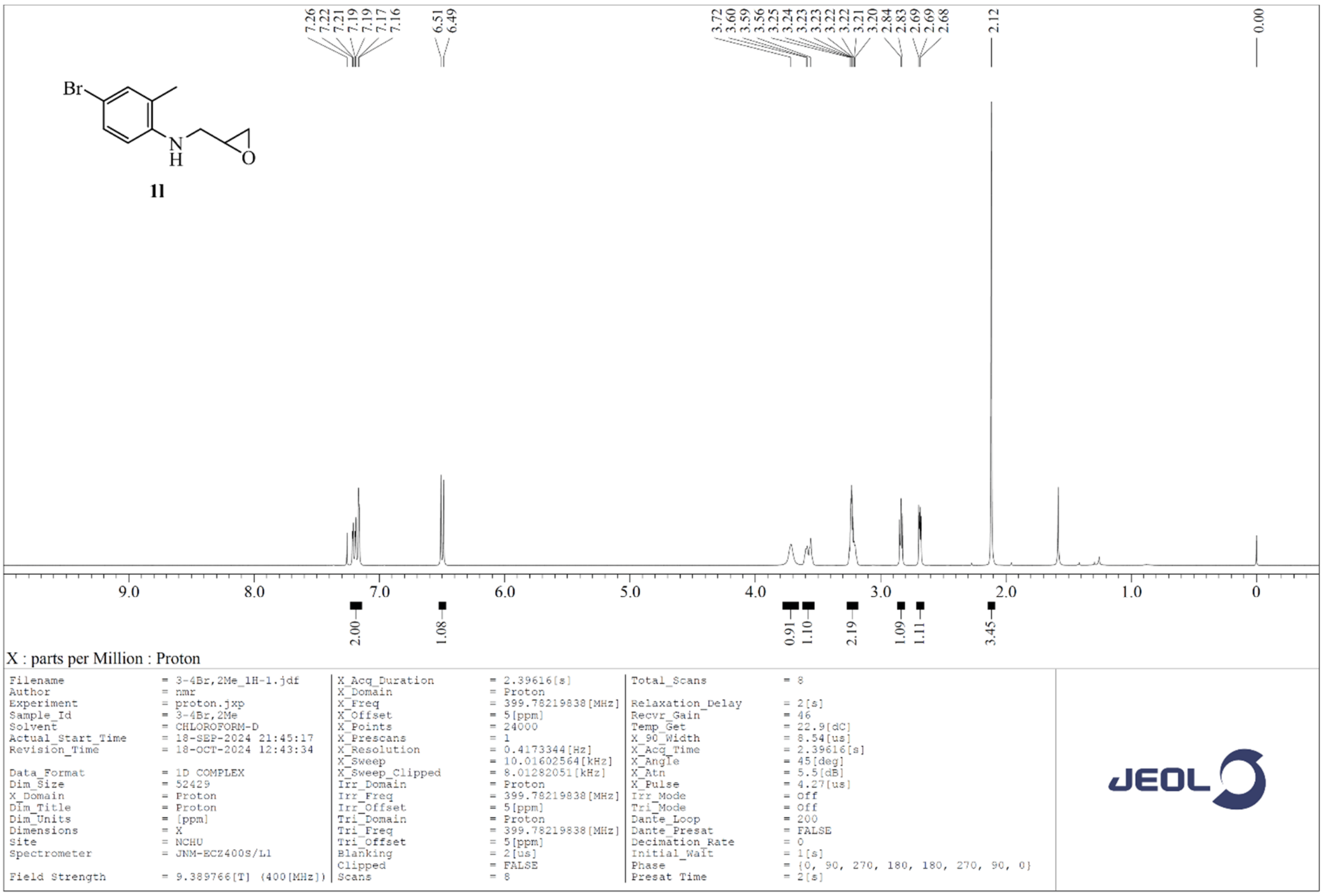


$^{1}H$ NMR spectrum of compound **1l** (400 MHz, $CDCl_3$)

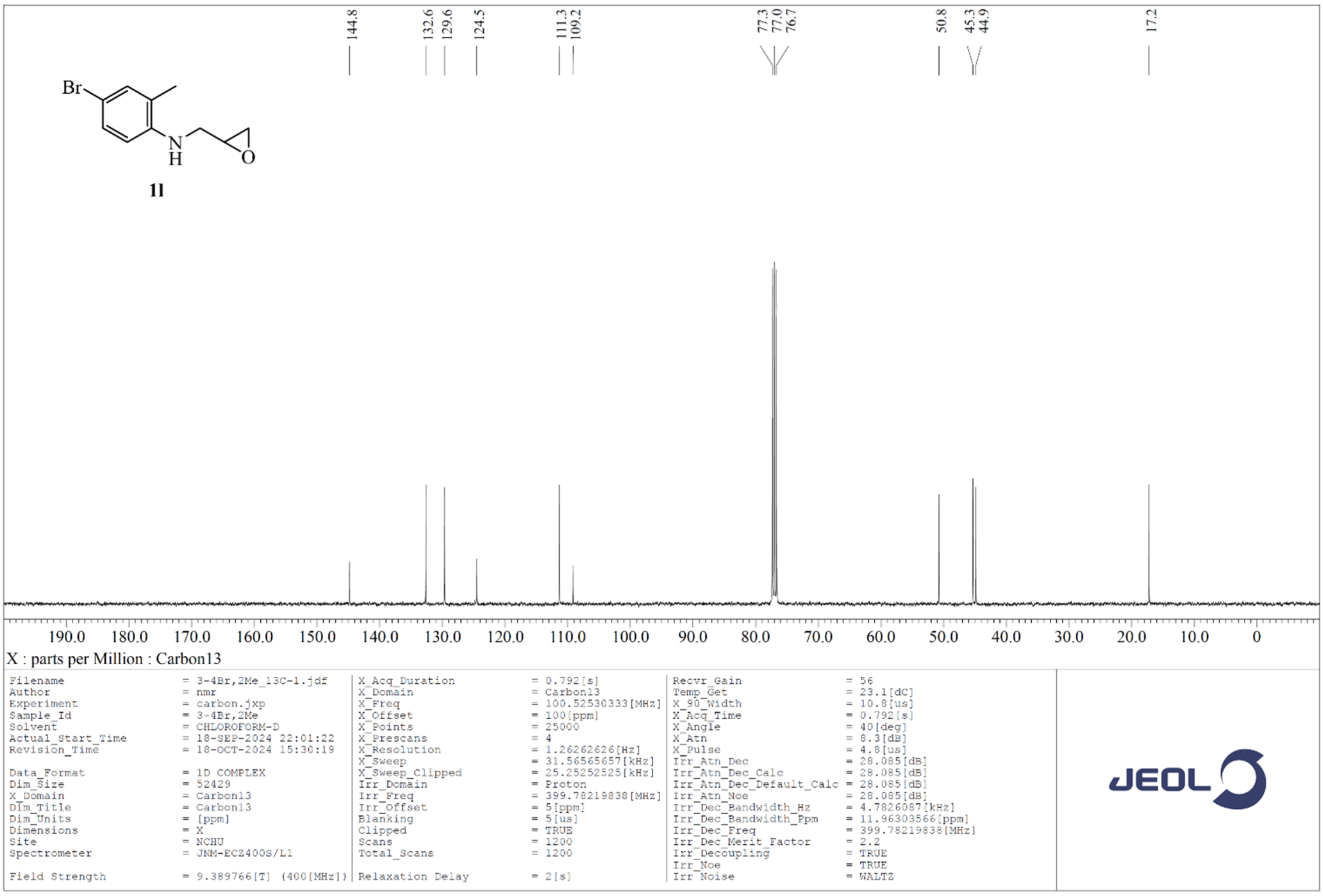


$^{13}C$ NMR spectrum of compound **1l** (101 MHz, $CDCl_3$)

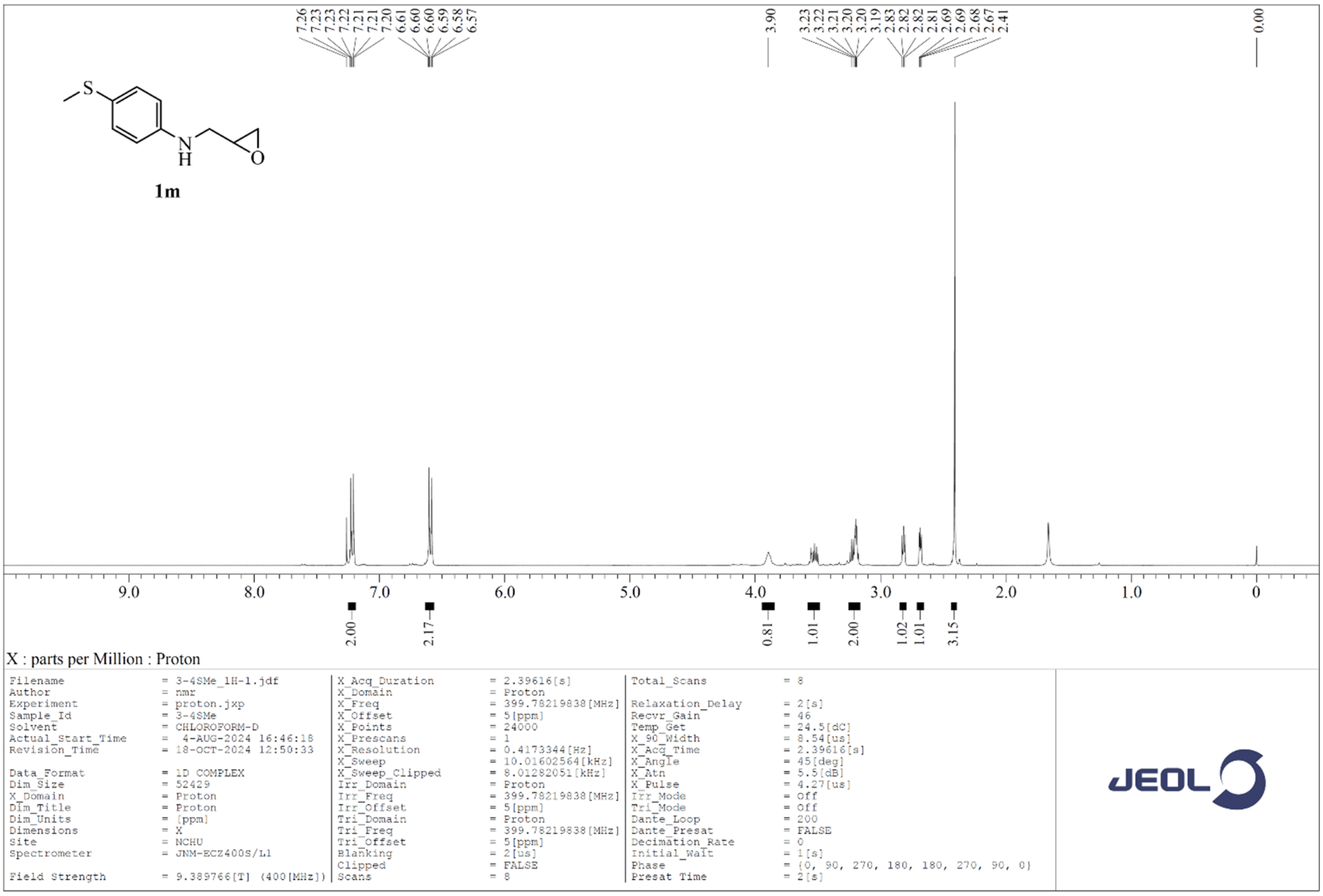


$^{1}H$ NMR spectrum of compound **1m** (400 MHz, $CDCl_3$)

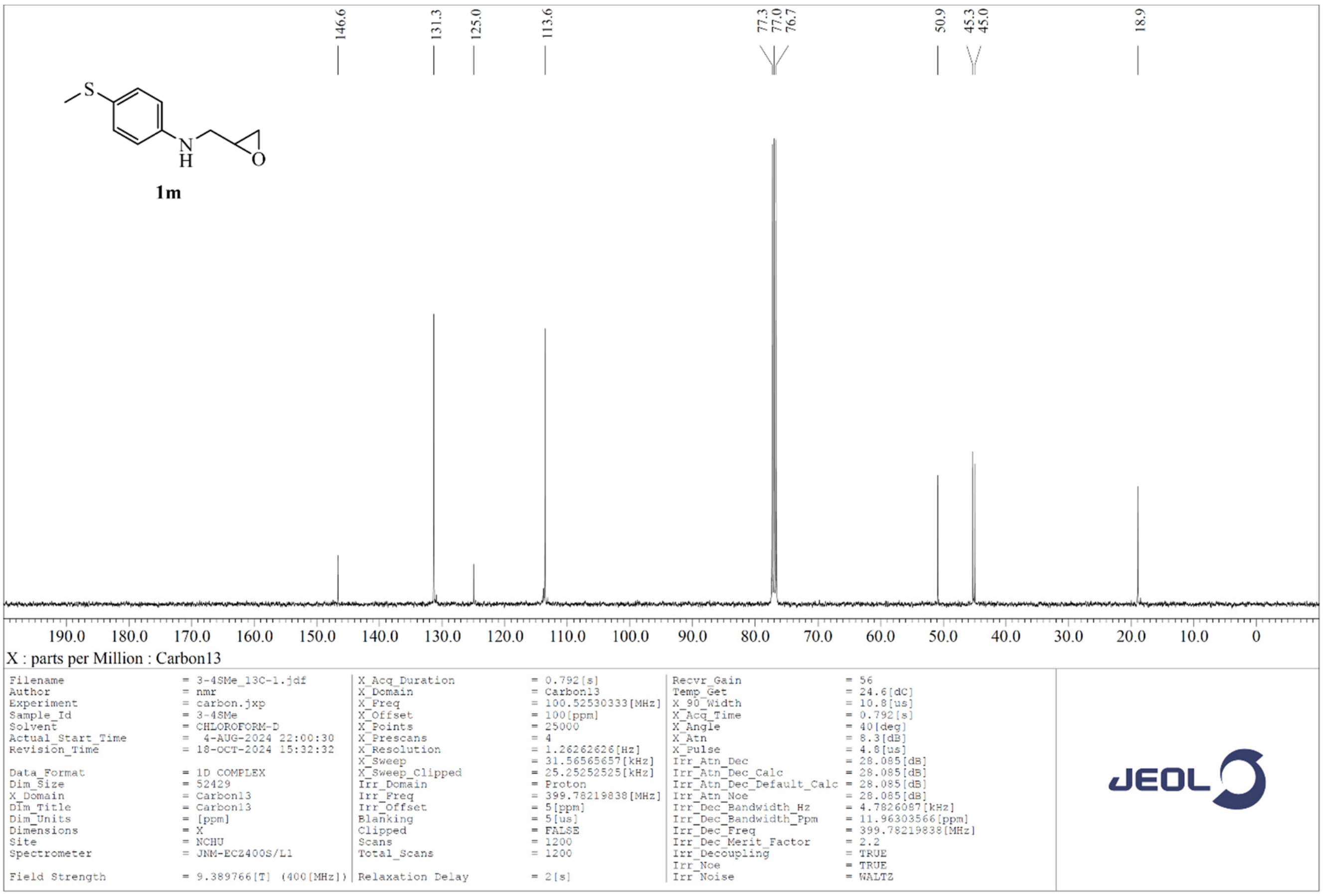


$^{13}C$ NMR spectrum of compound **1m** (101 MHz, $CDCl_3$)

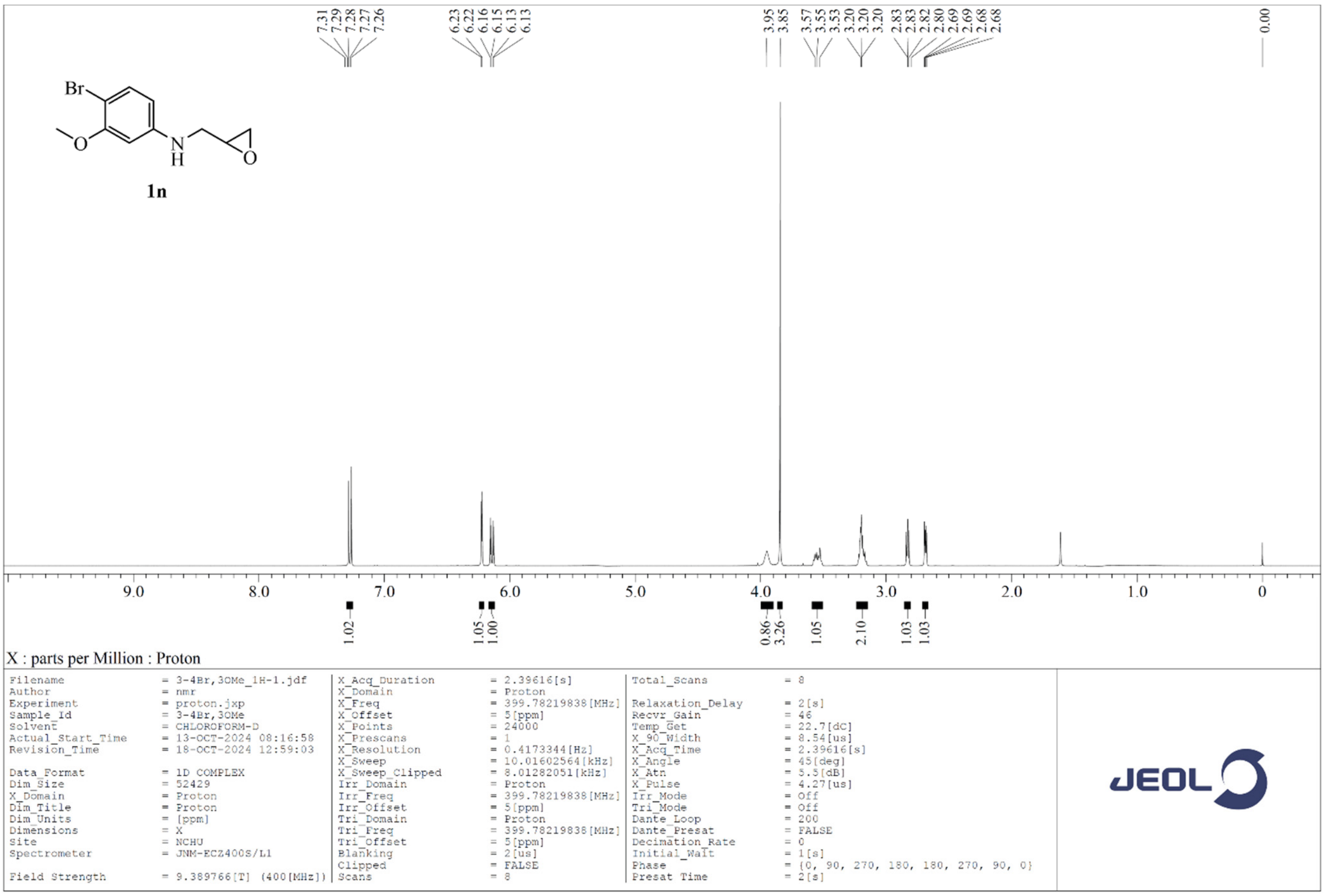


$^{1}$H NMR spectrum of compound **1n** (400 MHz, $CDCl_3$)

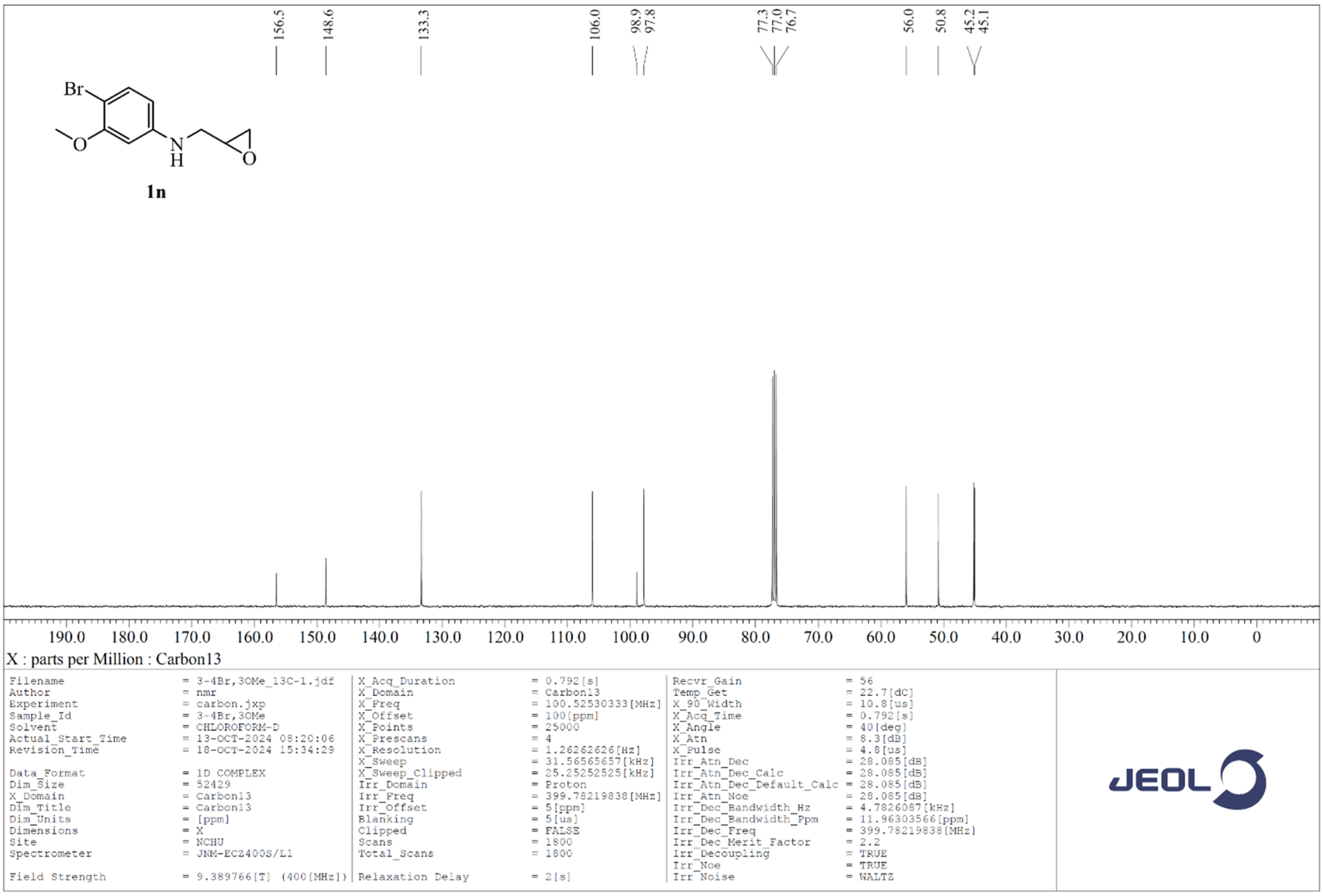


$^{13}$C NMR spectrum of compound **1n** (101 MHz, $CDCl_3$)

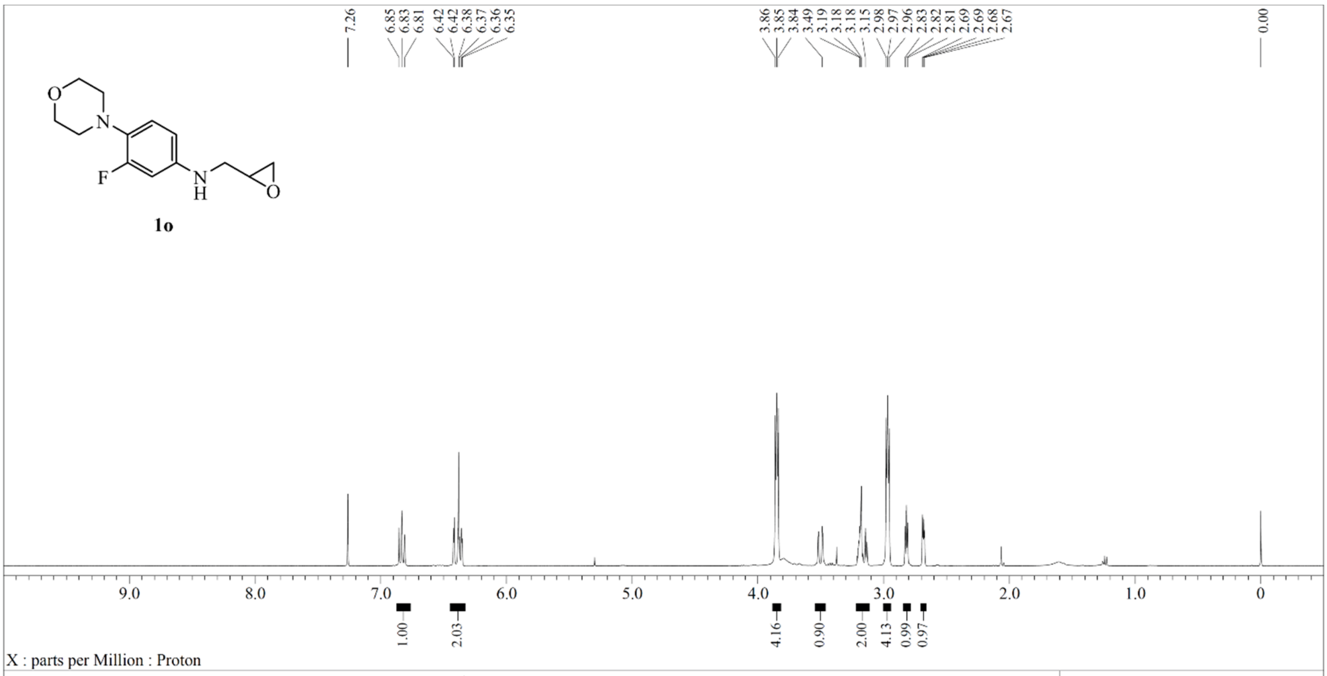


Filename = 321 morpholine-epichlorohy_proton-1-2.jdf
Author = nmr
Experiment = proton.jxp
Sample_Id = 321 morpholine-epichlorohy
Solvent = CHLOROFORM-D
Actual_Start_Time = 26-MAR-2023 19:59:52
Revision_Time = 18-OCT-2024 13:33:03

Data_Format = 1D COMPLEX
Dim_Size = 52429
X_Domain = Proton
Dim_Title = Proton
Dim_Units = [ppm]
Dimensions = X
Site = NCHU
Spectrometer = JNM-ECZ400S/L1

Field Strength = 9.389766[T] (400[MHz])

X_Acq_Duration = 2.39616[s]
X_Domain = Proton
X_Freq = 399.78219838[MHz]
X_Offset = 5[ppm]
X_Points = 24000
X_Prescans = 1
X_Resolution = 0.4173344[Hz]
X_Sweep = 10.01602564[kHz]
X_Sweep_Clipped = 8.01282051[kHz]
Irr_Domain = Proton
Irr_Freq = 399.78219838[MHz]
Irr_Offset = 5[ppm]
Tri_Domain = Proton
Tri_Freq = 399.78219838[MHz]
Tri_Offset = 5[ppm]
Blanking = 2[us]
Clipped = FALSE
Scans = 8

$^{1}$H NMR spectrum of compound **1o** (400 MHz, $CDCl_3$)

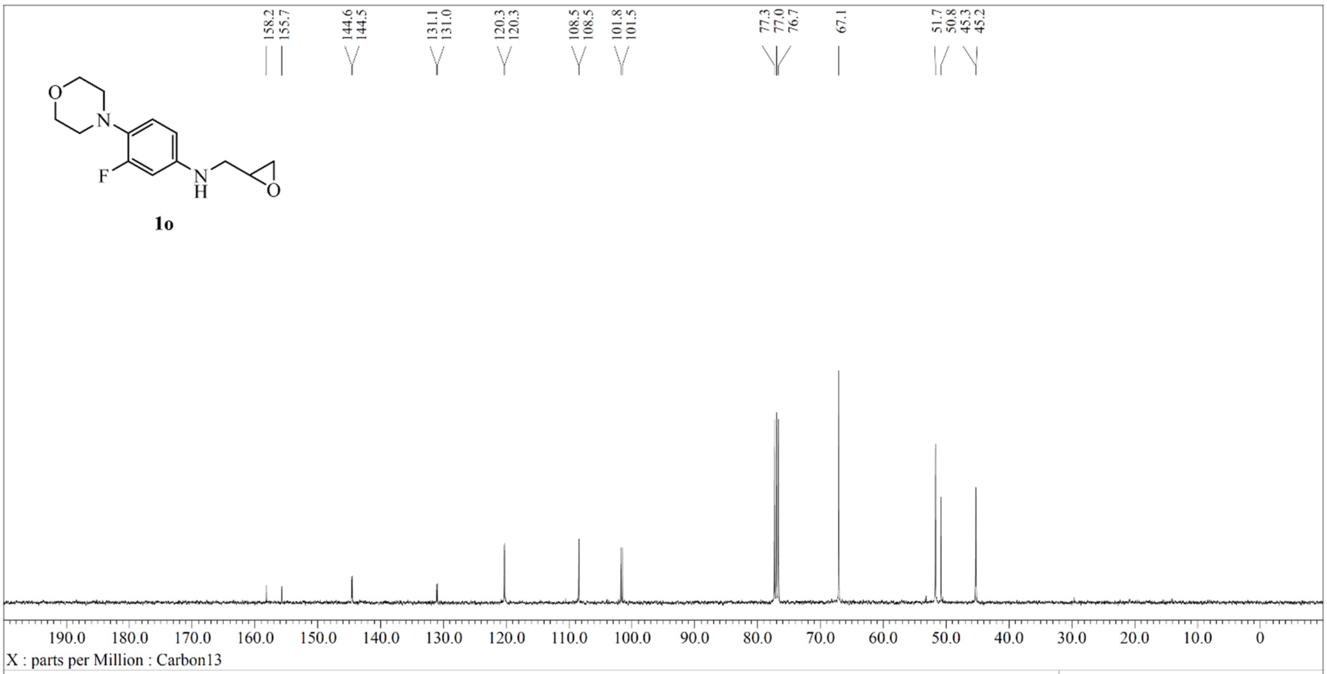


Filename = mor-epi (R) carbon_carbon-1-2.jdf
Author = nmr
Experiment = carbon.jxp
Sample_Id = mor-epi (R) carbon
Solvent = CHLOROFORM-D
Actual_Start_Time = 13-APR-2023 17:07:05
Revision_Time = 18-OCT-2024 16:09:26

Data_Format = 1D COMPLEX
Dim_Size = 52429
X_Domain = Carbon13
Dim_Title = Carbon13
Dim_Units = [ppm]
Dimensions = X
Site = NCHU
Spectrometer = JNM-ECZ400S/L1

Field Strength = 9.389766[T] (400[MHz])

X_Acq_Duration = 0.792[s]
X_Domain = Carbon13
X_Freq = 100.52530333[MHz]
X_Offset = 100[ppm]
X_Points = 25000
X_Prescans = 4
X_Resolution = 1.26262626[Hz]
X_Sweep = 31.56565657[kHz]
X_Sweep_Clipped = 25.25252525[kHz]
Irr_Domain = Proton
Irr_Freq = 399.78219838[MHz]
Irr_Offset = 5[ppm]
Blanking = 5[us]
Clipped = TRUE
Scans = 322
Total_Scans = 322

Relaxation Delay = 2[s]

$^{13}$C NMR spectrum of compound **1o** (101 MHz, $CDCl_3$)

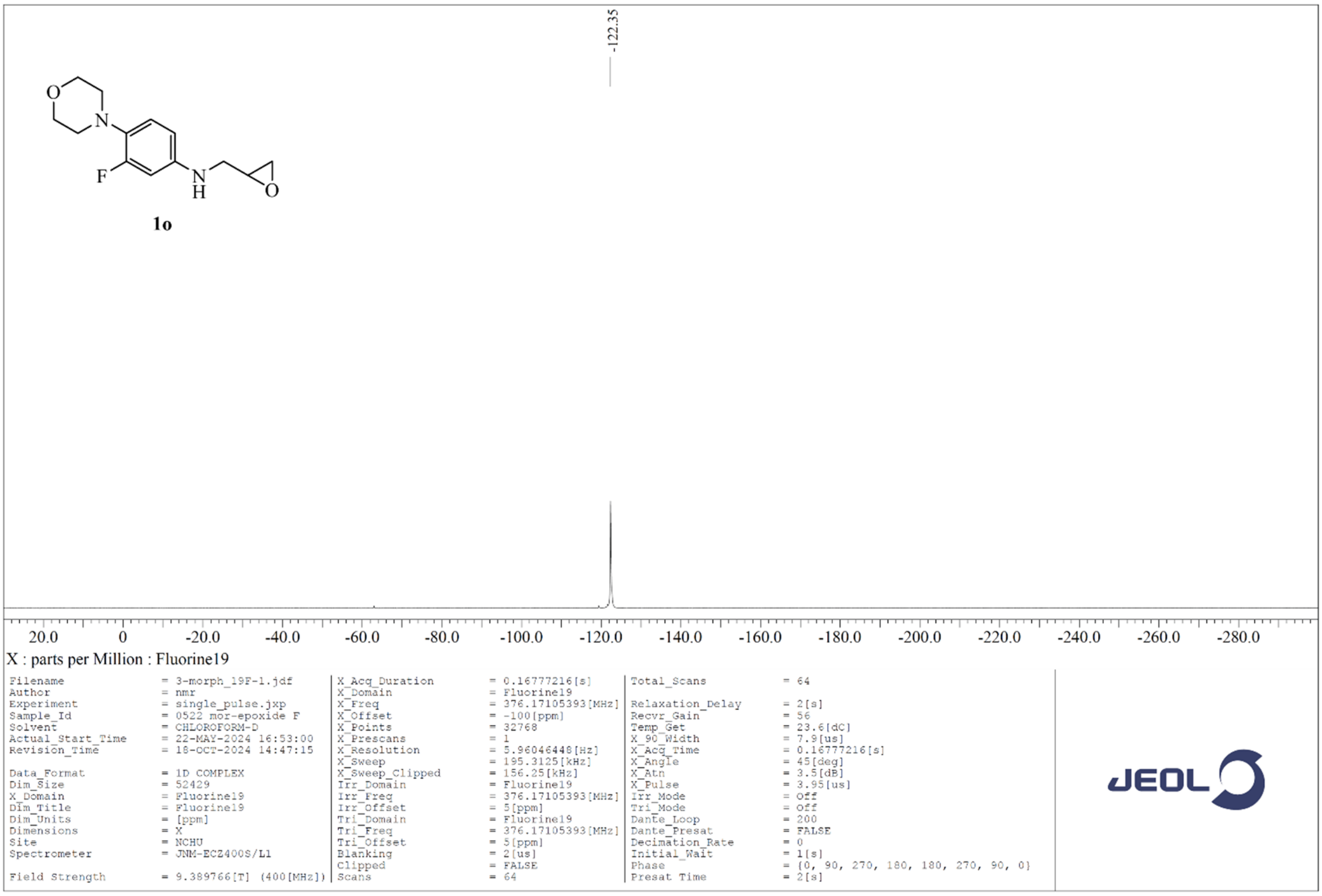


$^{19}F$ NMR spectrum of compound **1o** (376 MHz, $CDCl_3$)

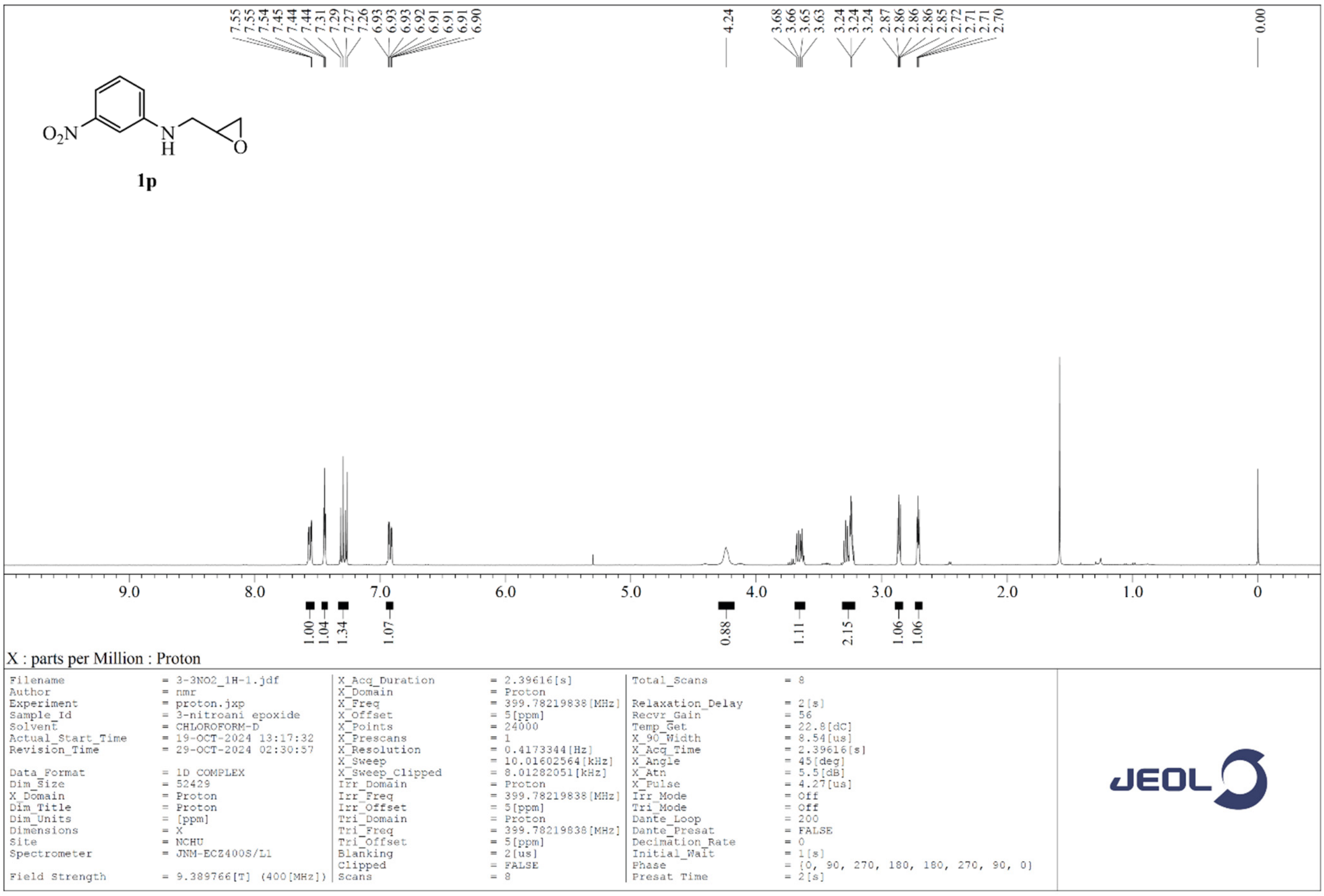


$^{1}H$ NMR spectrum of compound **1p** (400 MHz, $CDCl_3$)

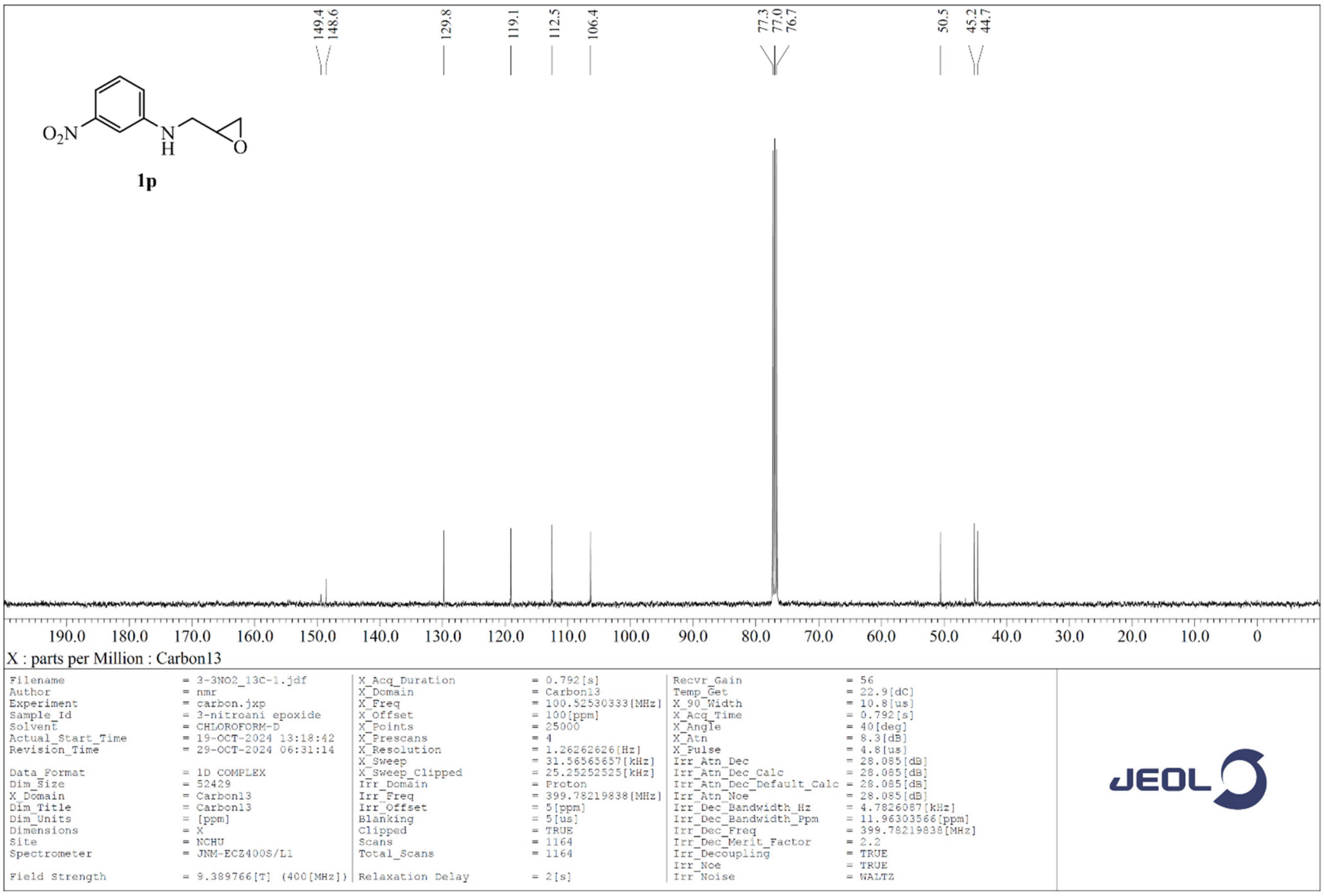


$^{13}$C NMR spectrum of compound **1p** (101 MHz, $CDCl_3$)

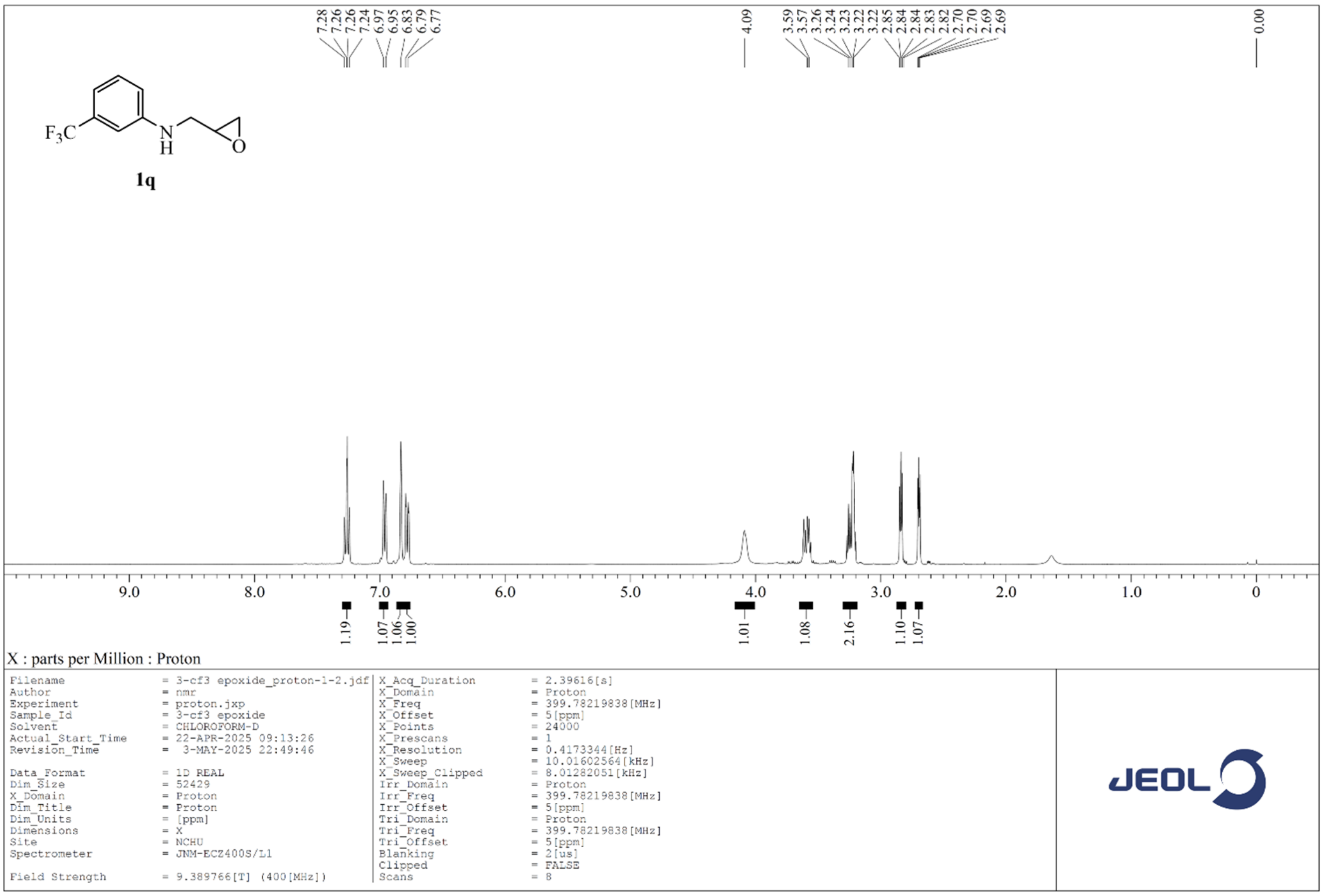


$^{1}$H NMR spectrum of compound **1q** (400 MHz, $CDCl_3$)

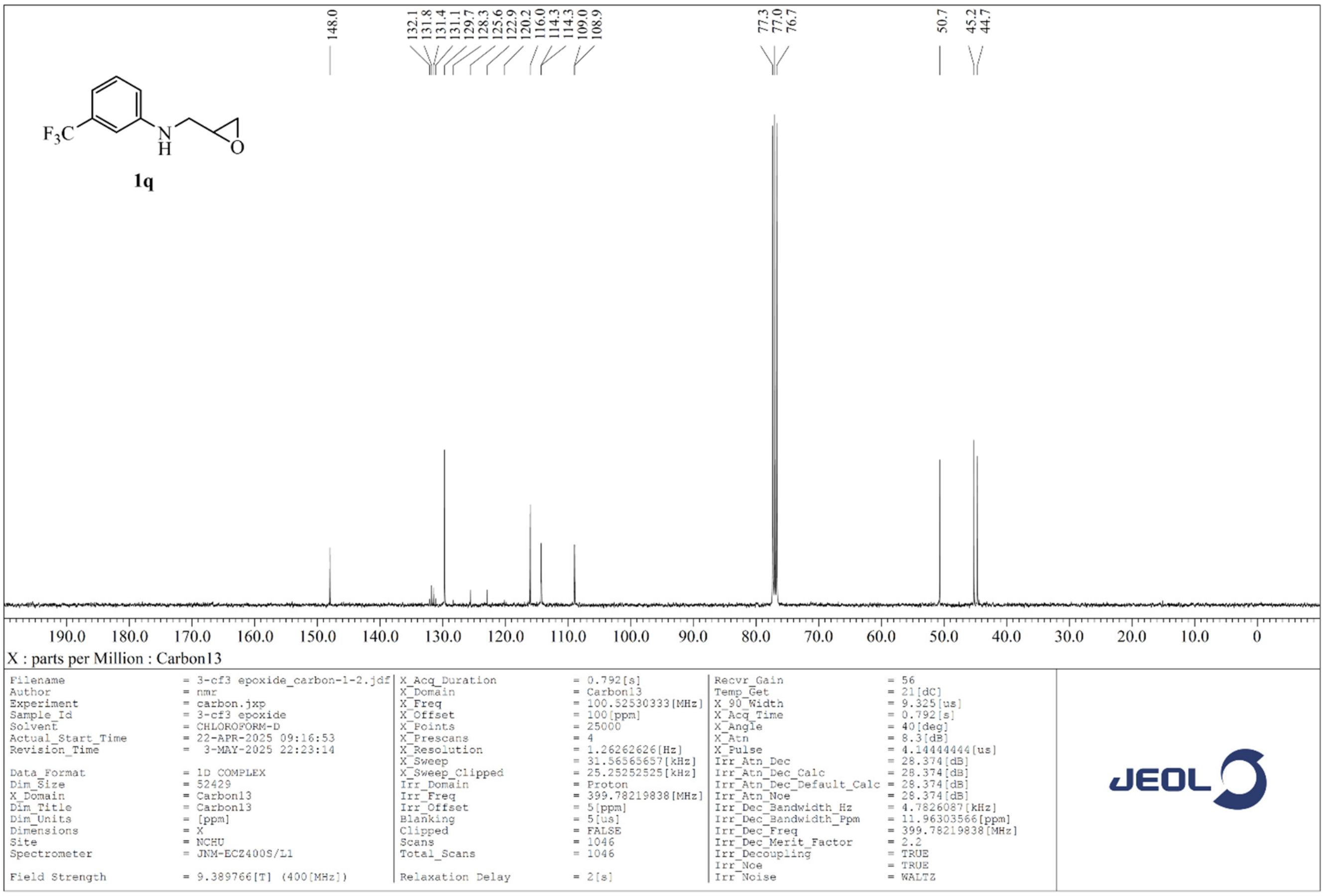


$^{13}C$ NMR spectrum of compound **1q** (101 MHz, $CDCl_3$)

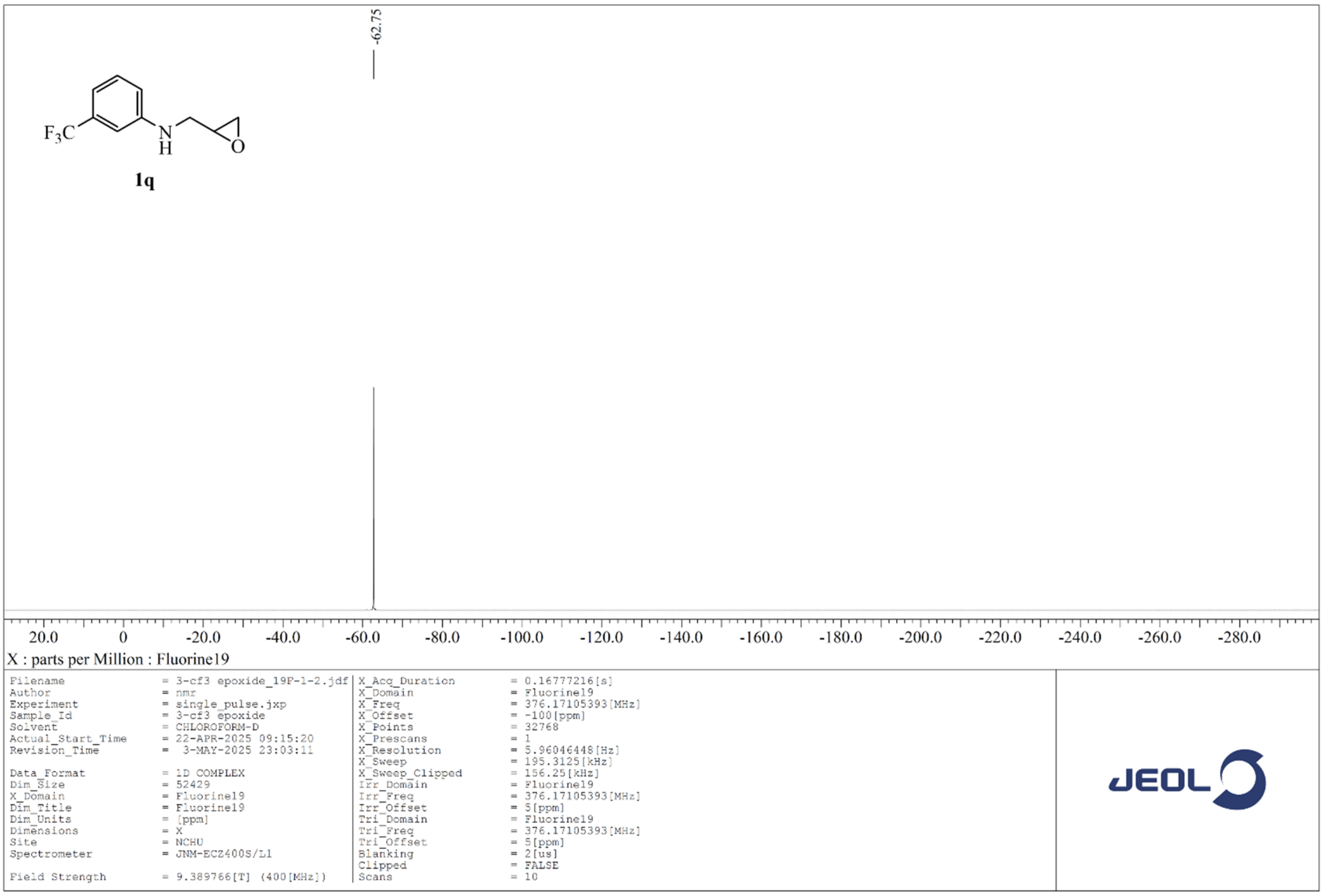


$^{19}F$ NMR spectrum of compound **1q** (376 MHz, $CDCl_3$)

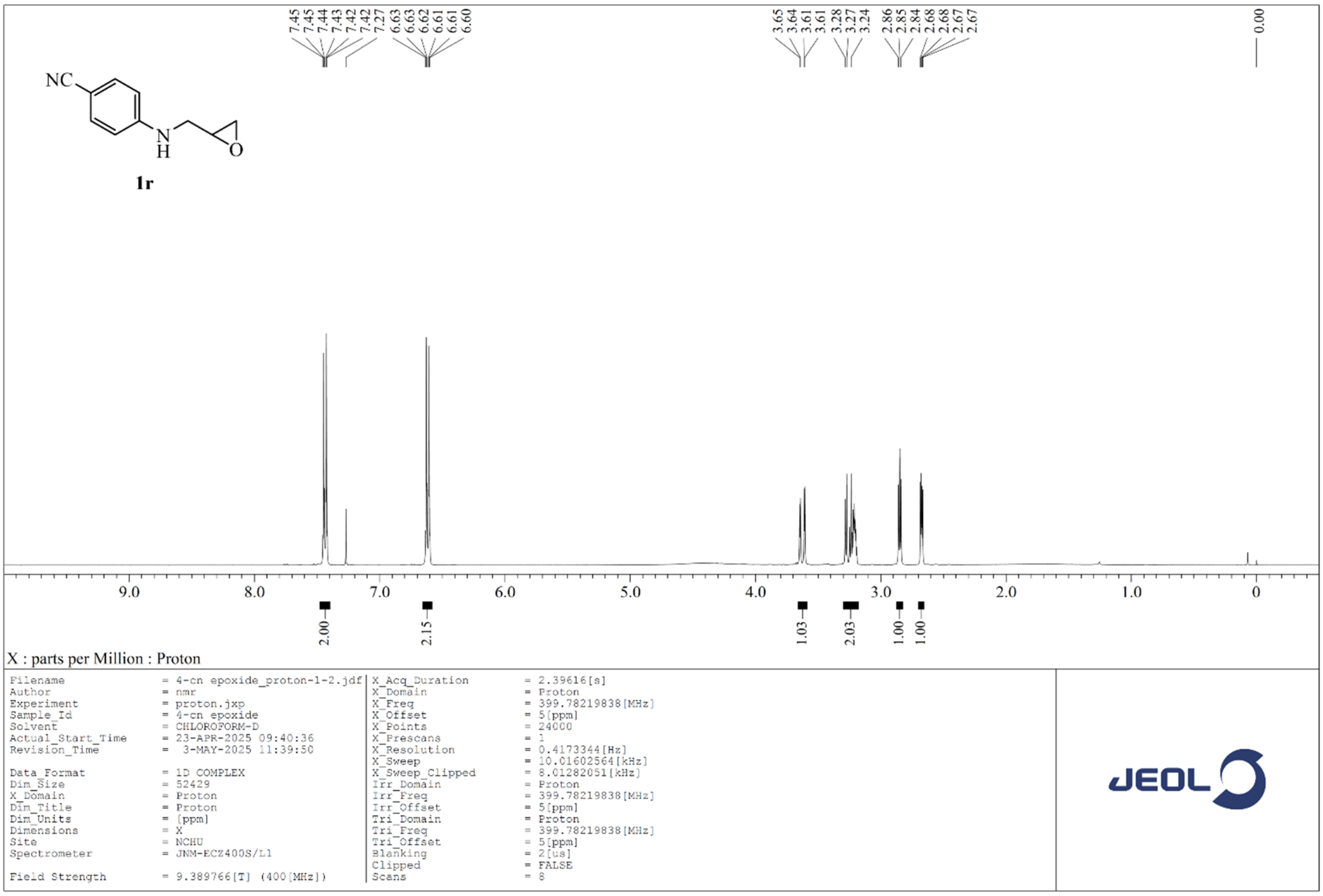


$^{1}H$ NMR spectrum of compound **1r** (400 MHz, $CDCl_3$)

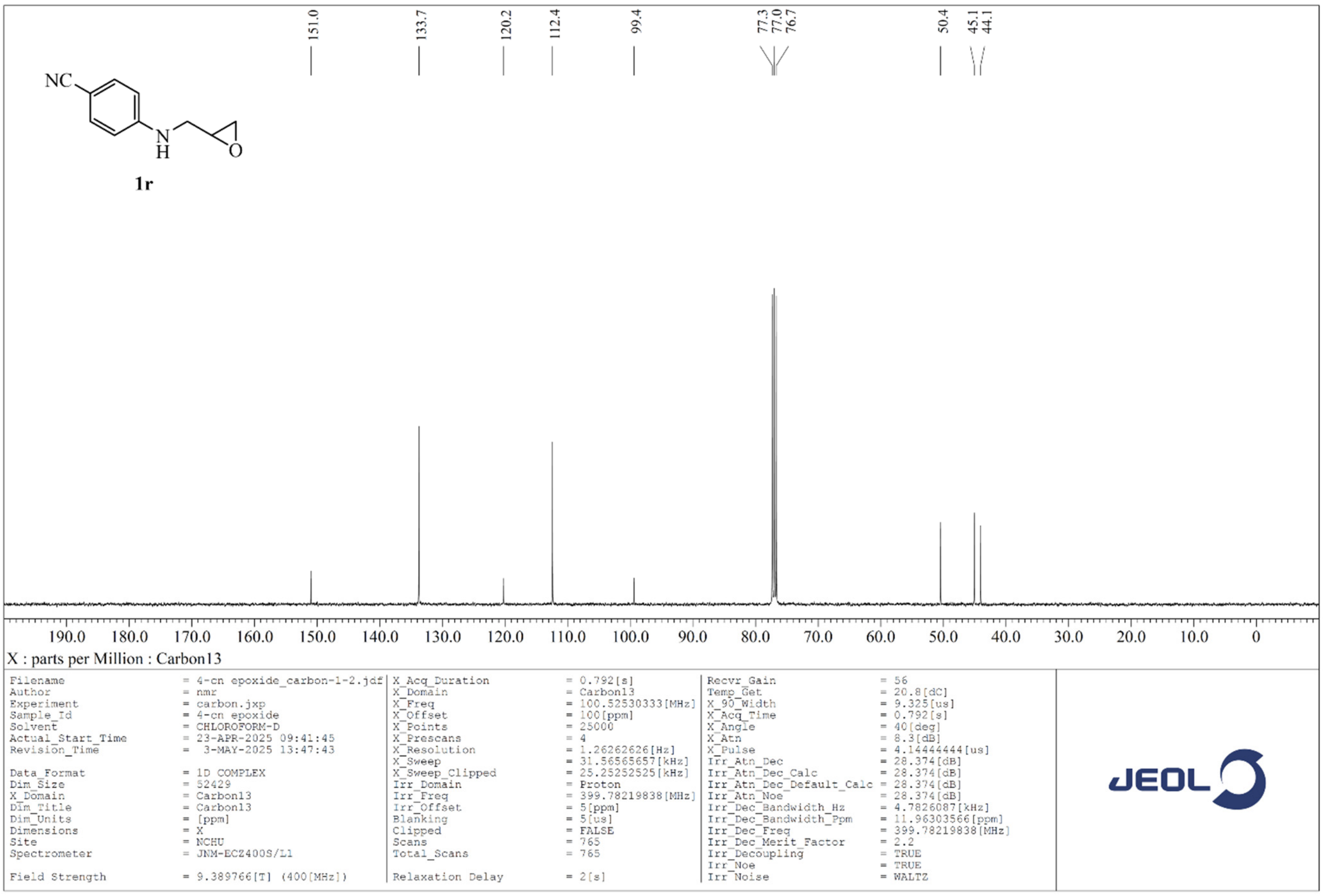


$^{13}C$ NMR spectrum of compound **1r** (101 MHz, $CDCl_3$)

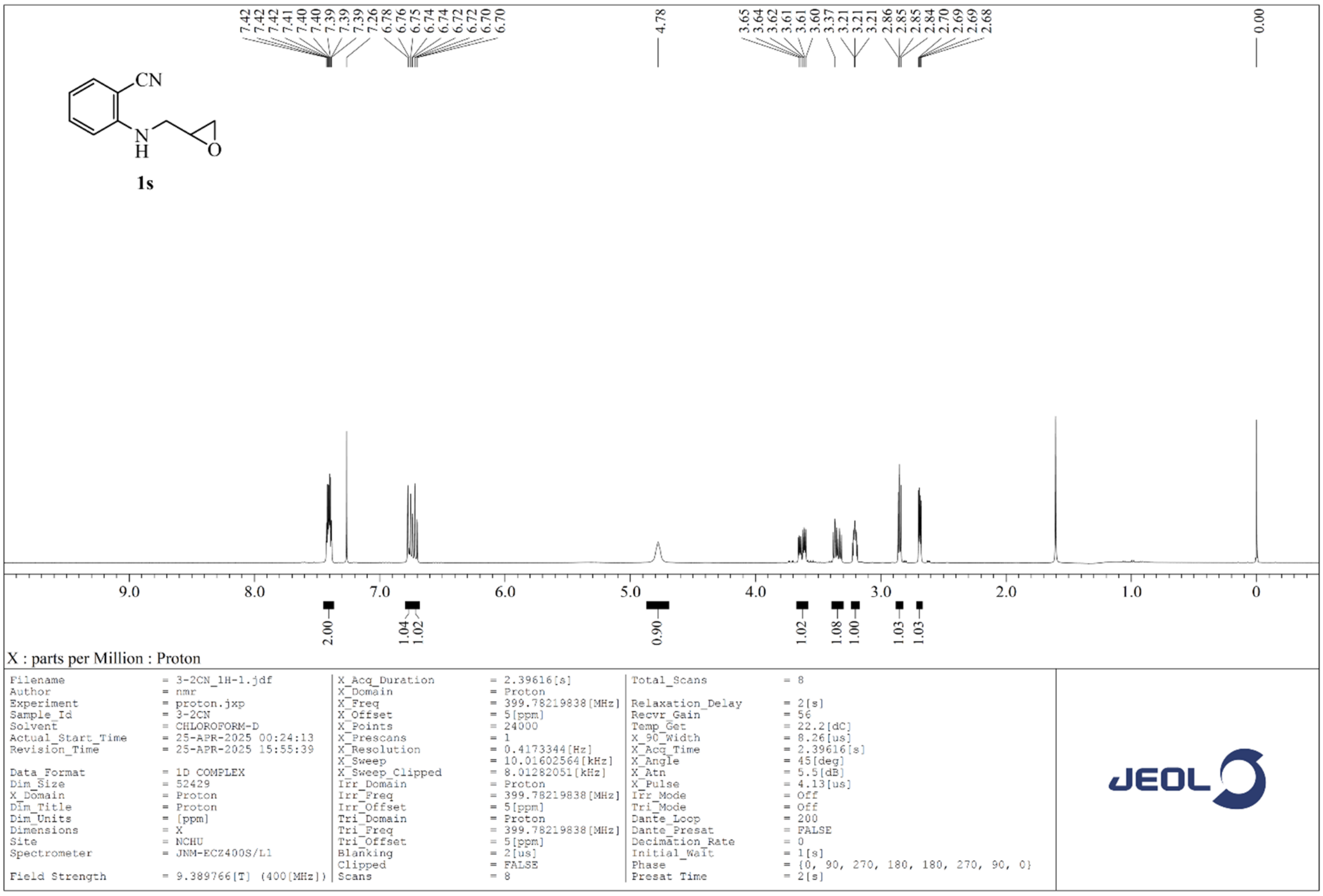


$^{1}$H NMR spectrum of compound **1s** (400 MHz, $CDCl_3$)

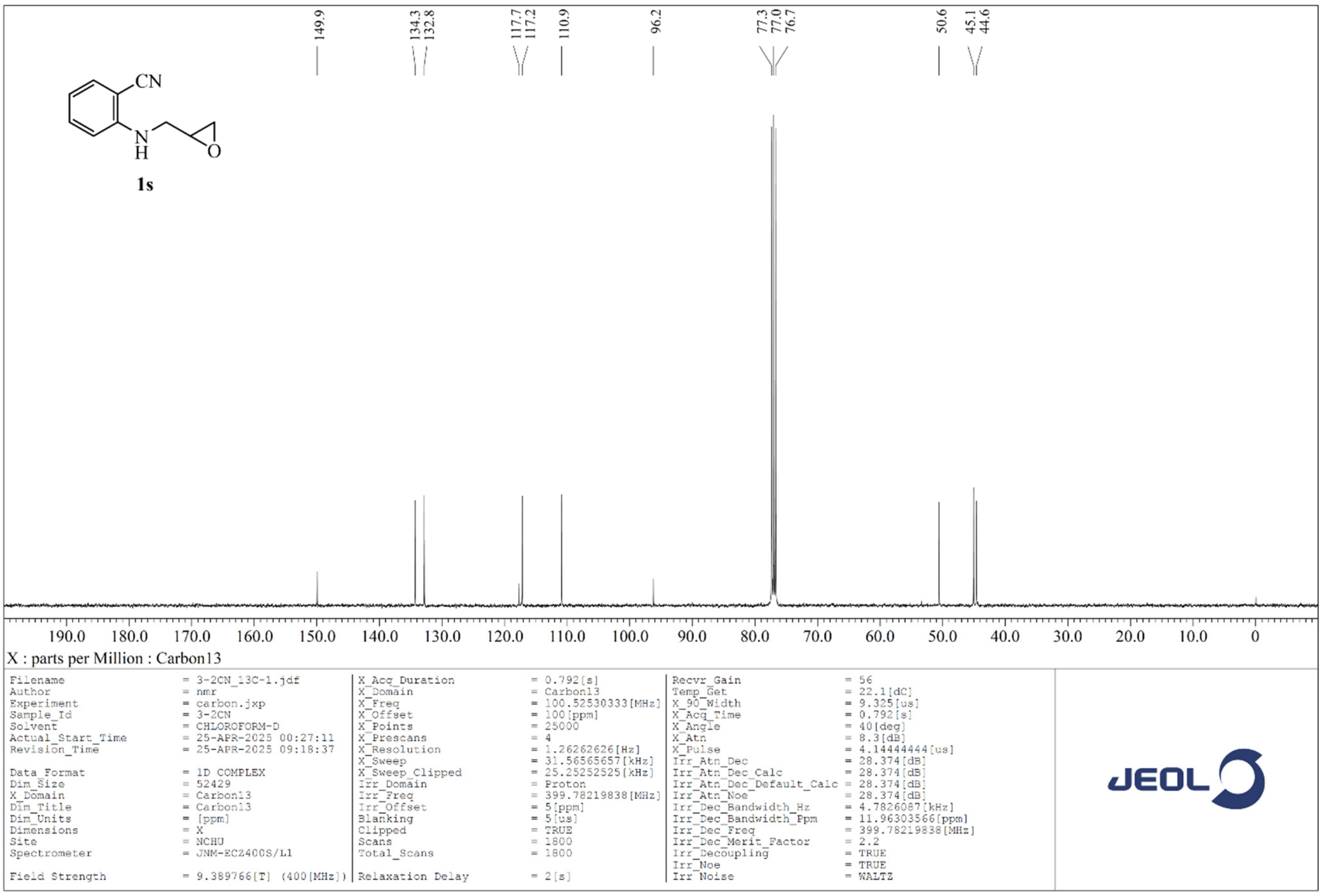


$^{13}$C NMR spectrum of compound **1s** (101 MHz, $CDCl_3$)

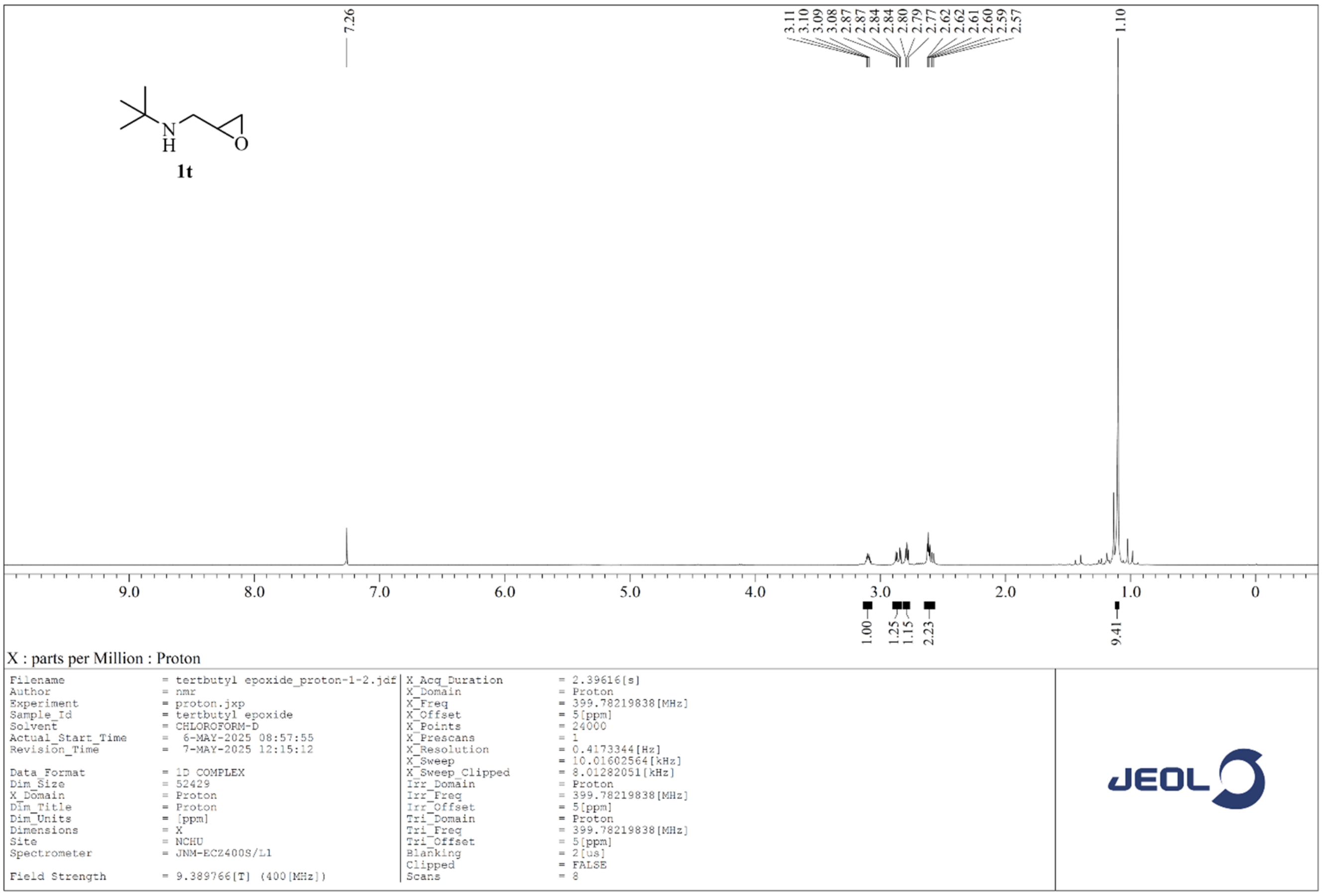


$^{1}$H NMR spectrum of compound **1t** (400 MHz, $CDCl_3$)

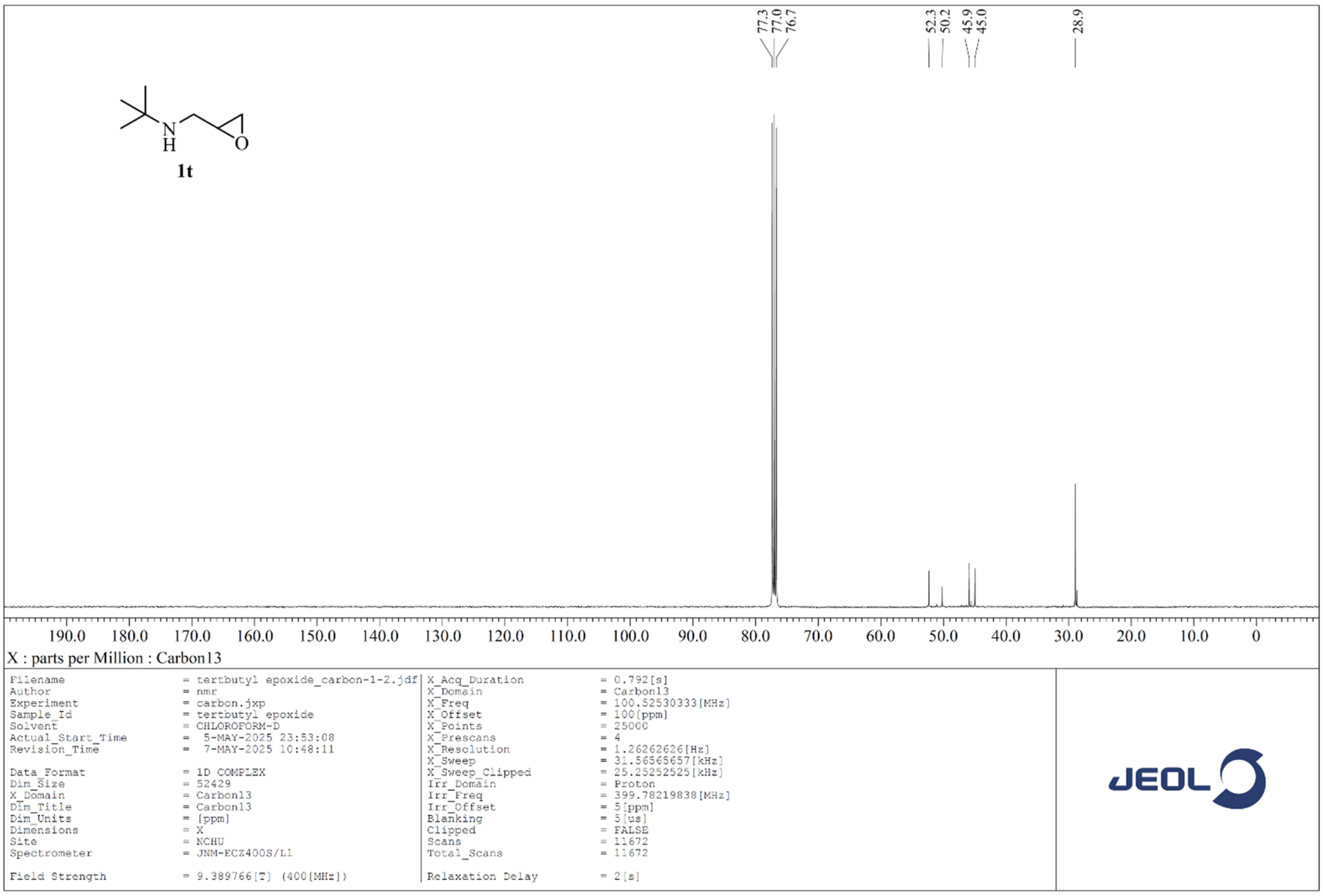


$^{13}$C NMR spectrum of compound **1t** (101 MHz, $CDCl_3$)

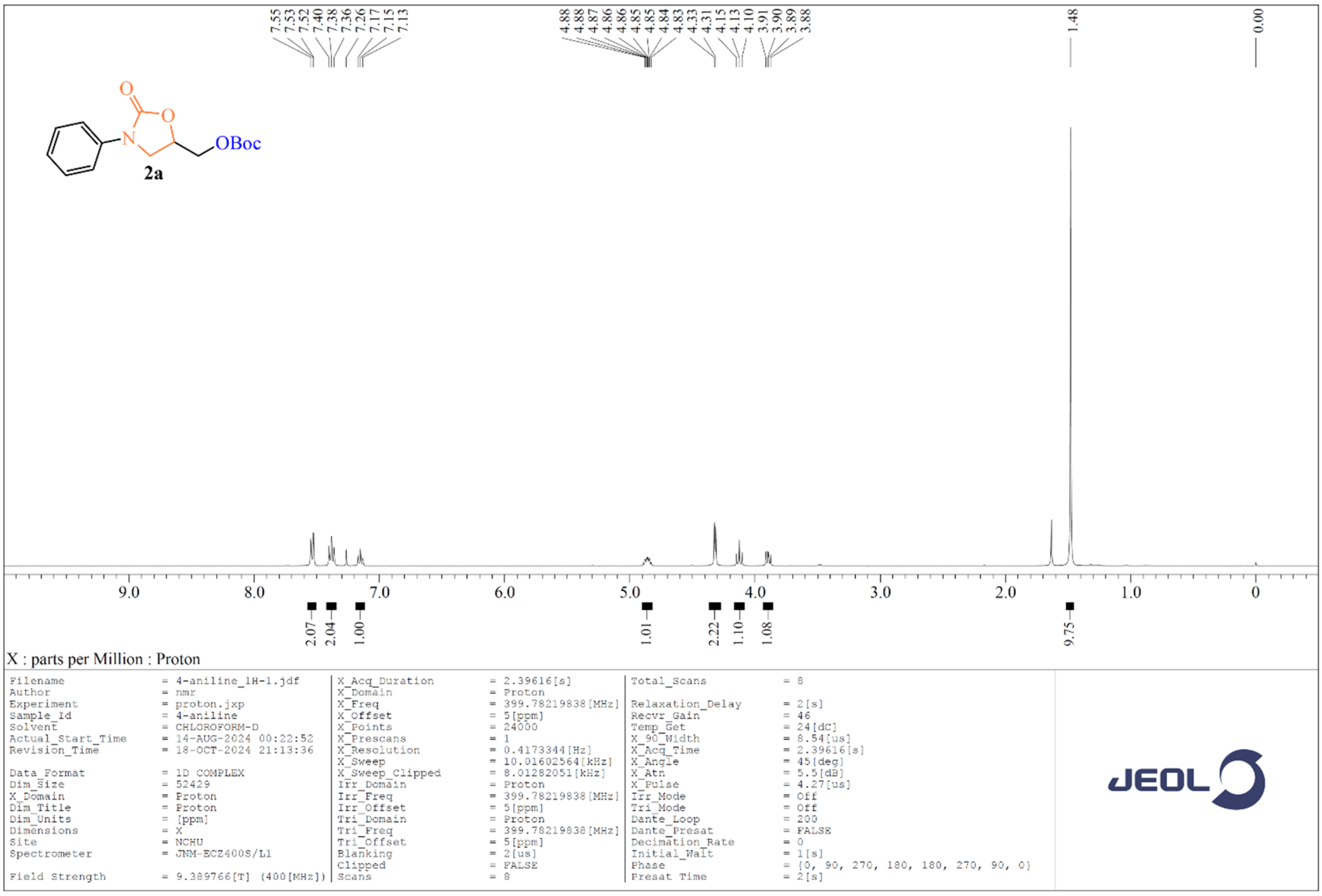

$^{1}H$ NMR spectrum of compound **2a** (400 MHz, $CDCl_3$)

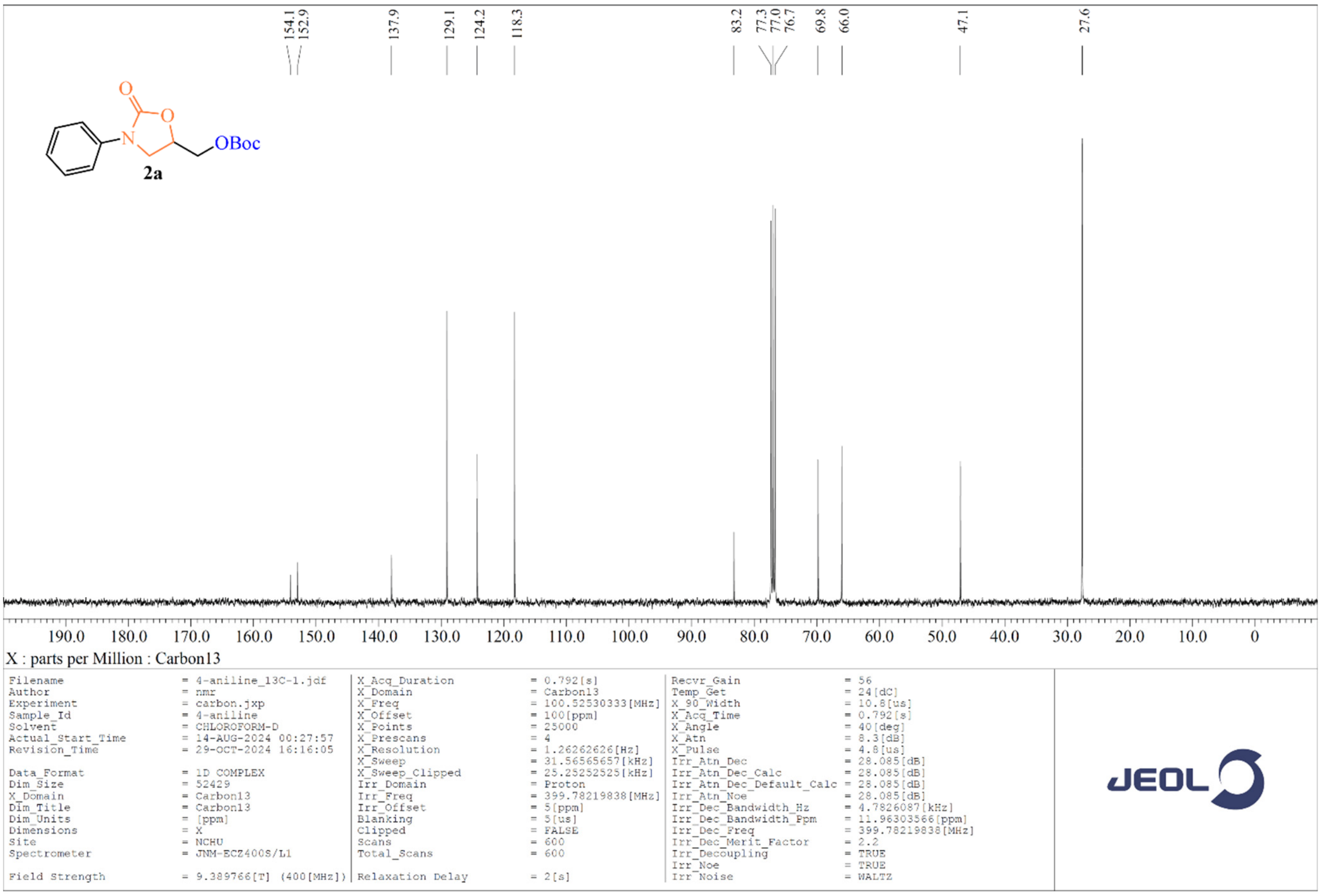

$^{13}C$ NMR spectrum of compound **2a** (101 MHz, $CDCl_3$)

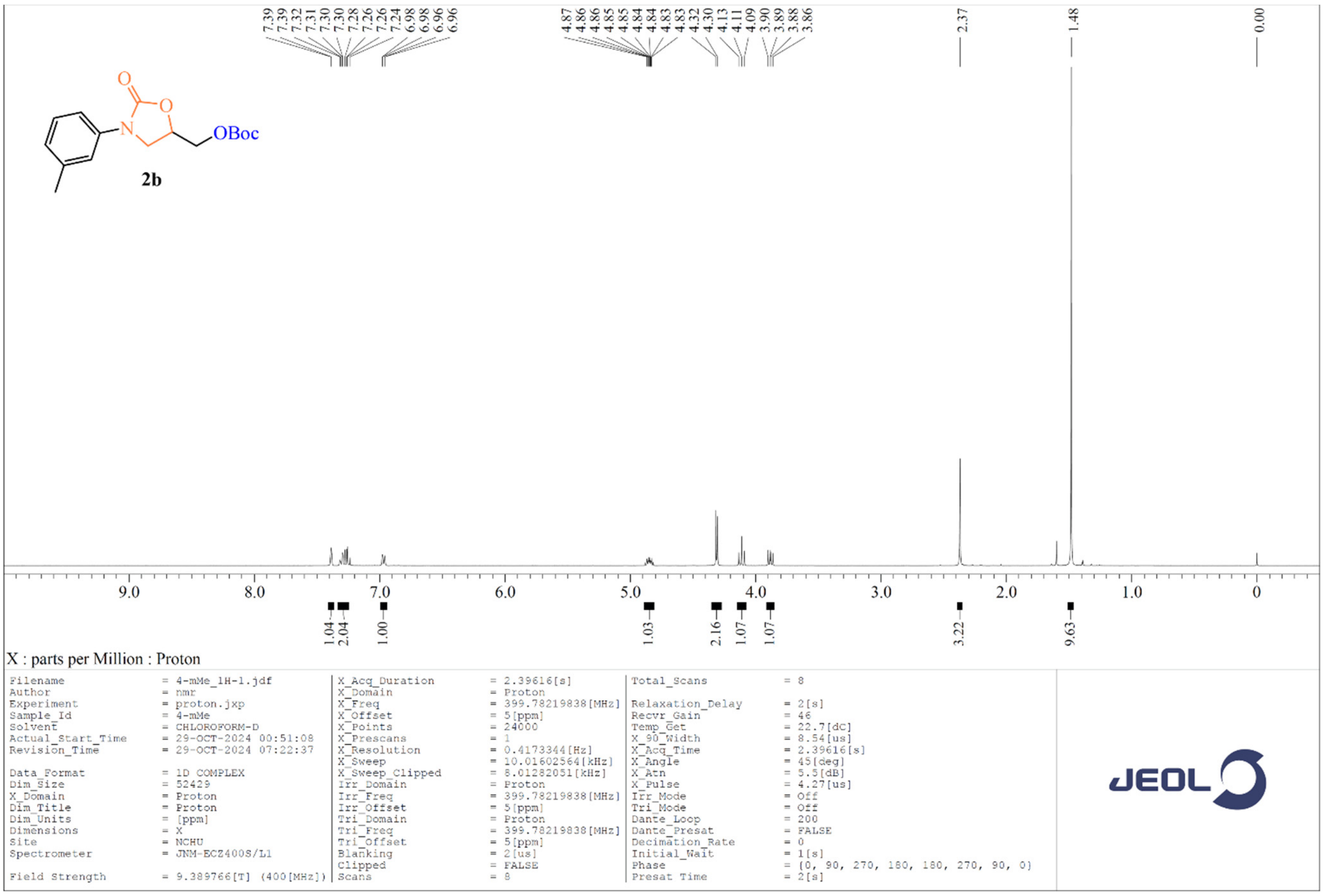


$^{1}H$ NMR spectrum of compound **2b** (400 MHz, $CDCl_3$)

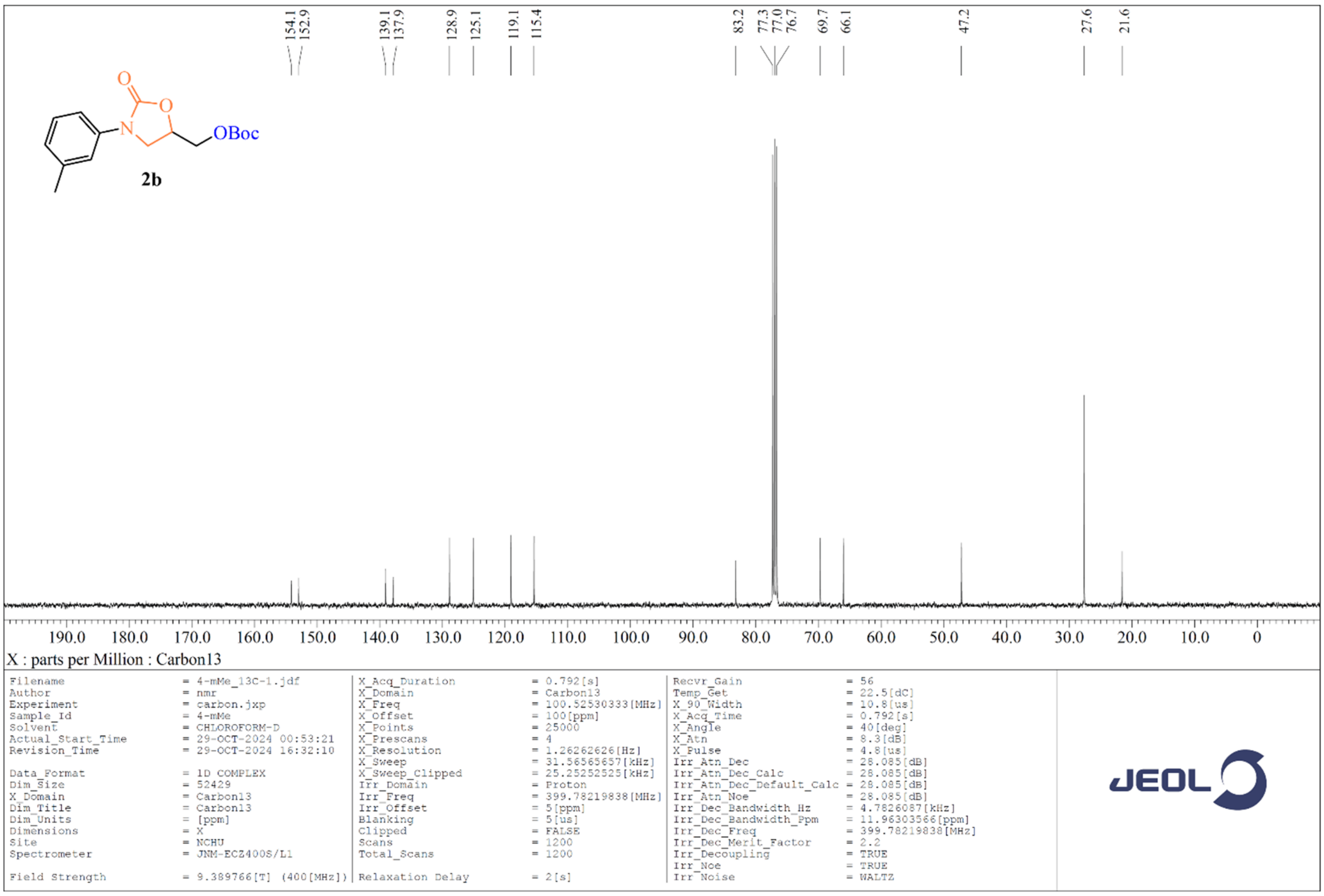


$^{13}C$ NMR spectrum of compound **2b** (101 MHz, $CDCl_3$)

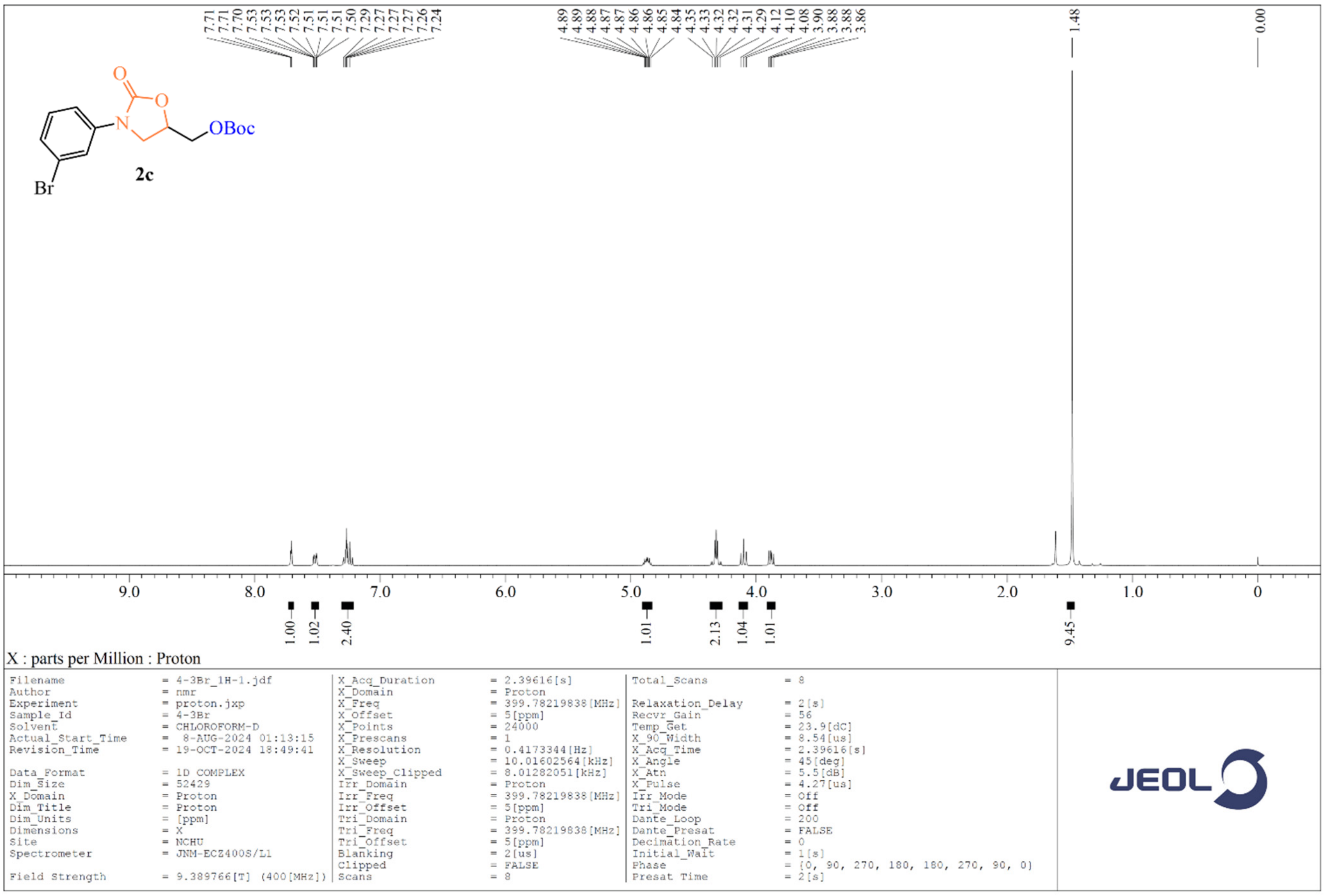


$^{1}$H NMR spectrum of compound **2c** (400 MHz, $CDCl_3$)

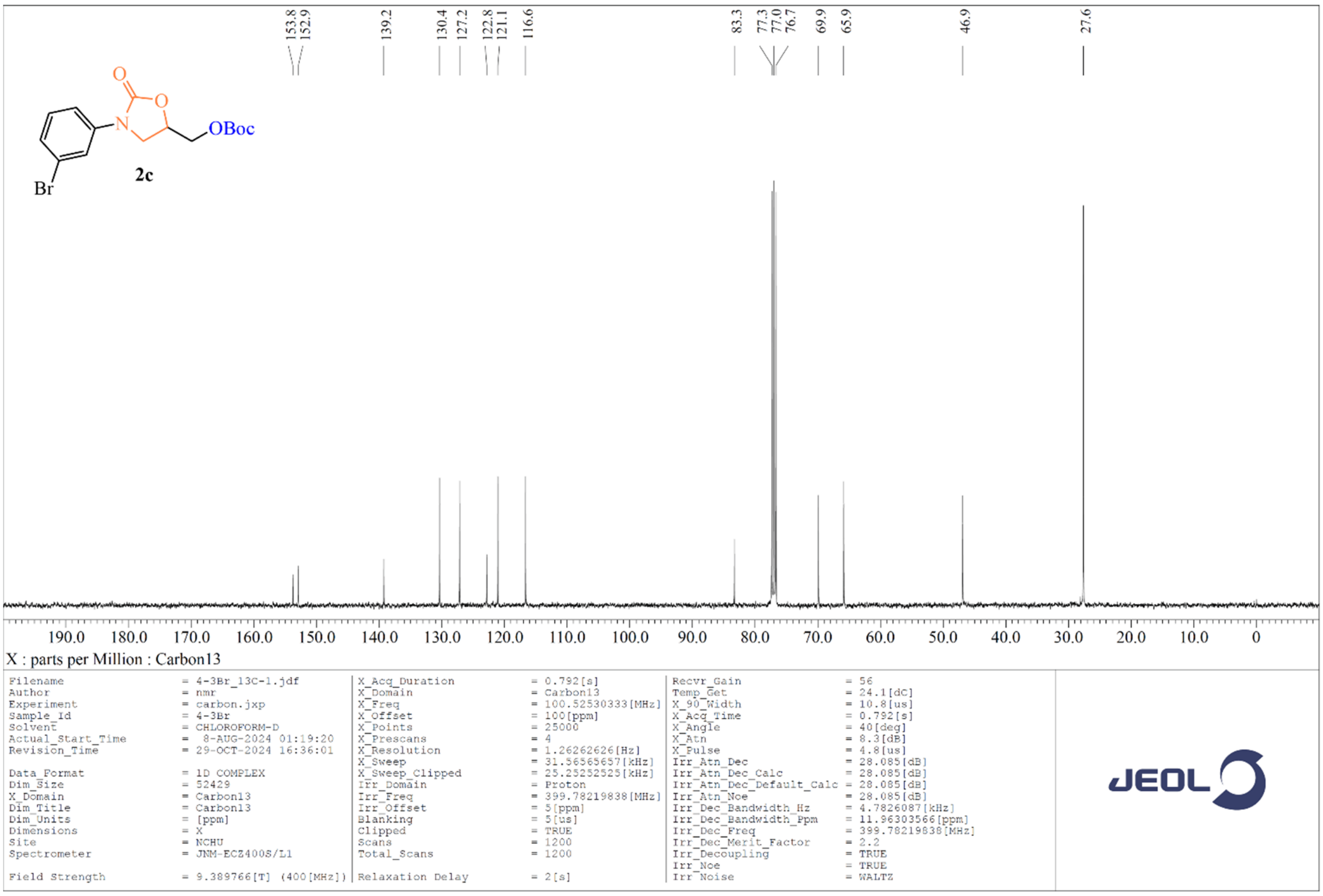


$^{13}$C NMR spectrum of compound **2c** (101 MHz, $CDCl_3$)

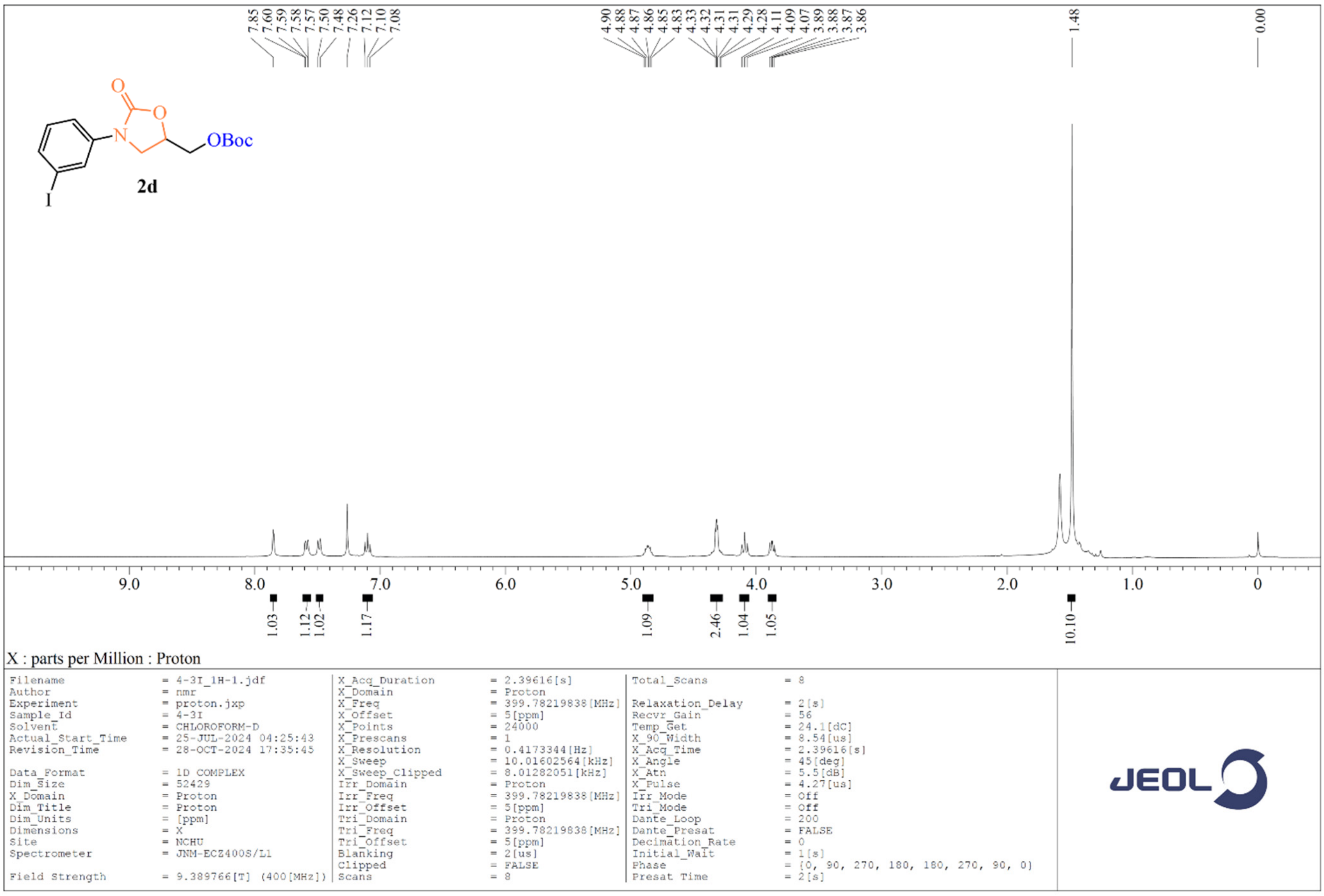


$^{1}$H NMR spectrum of compound **2d** (400 MHz, $CDCl_3$)

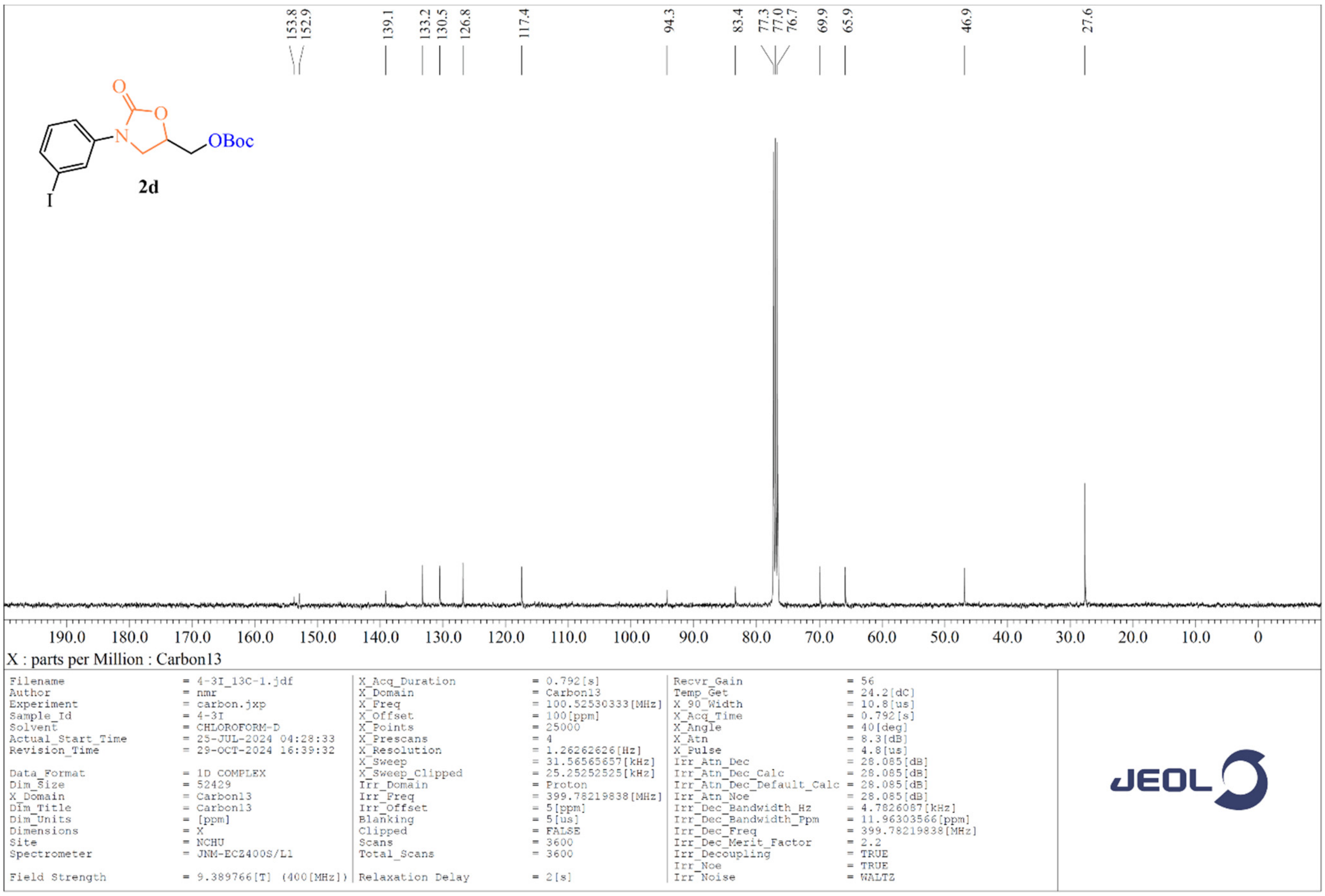


$^{13}$C NMR spectrum of compound **2d** (101 MHz, $CDCl_3$)

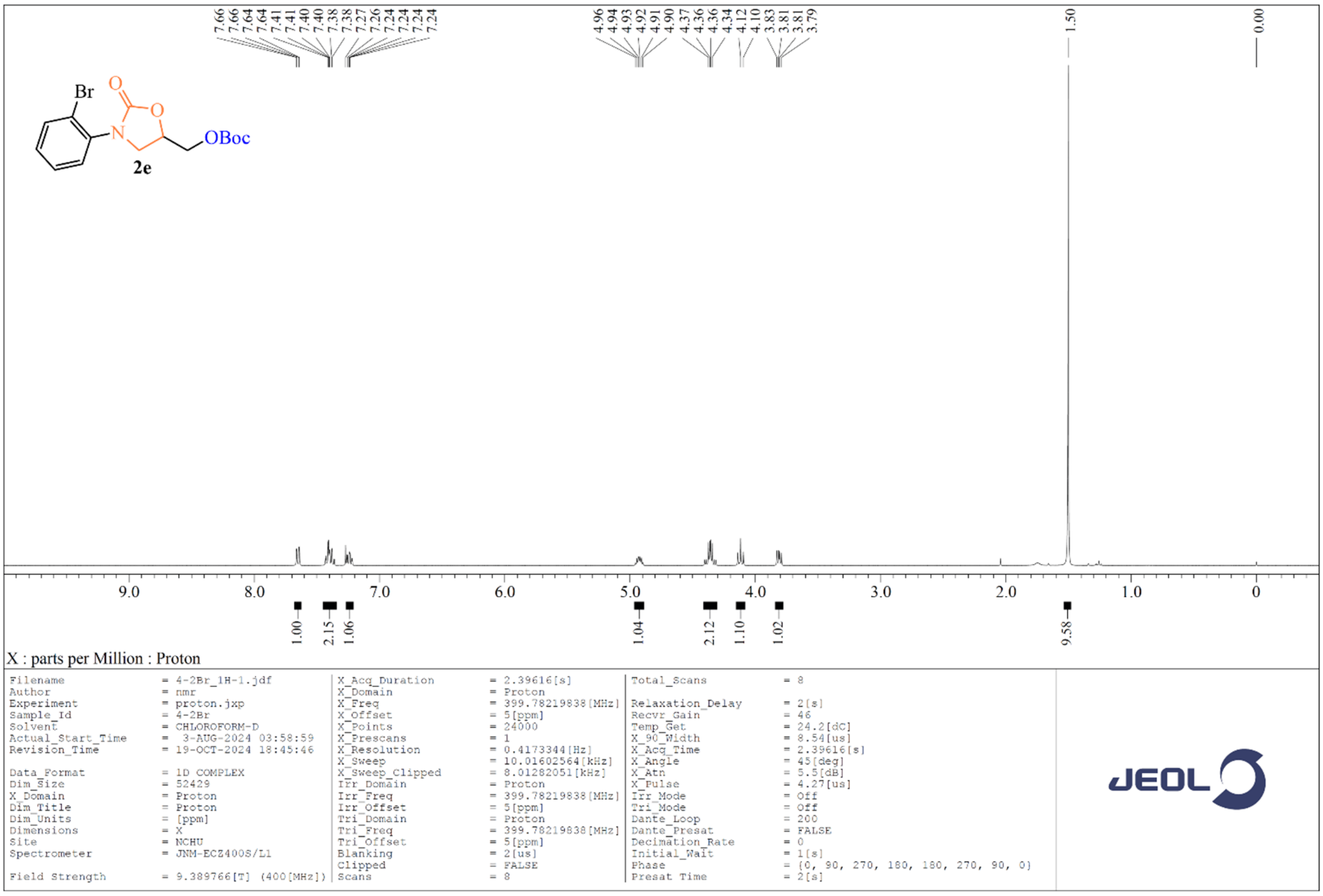


$^{1}$H NMR spectrum of compound **2e** (400 MHz, $CDCl_3$)

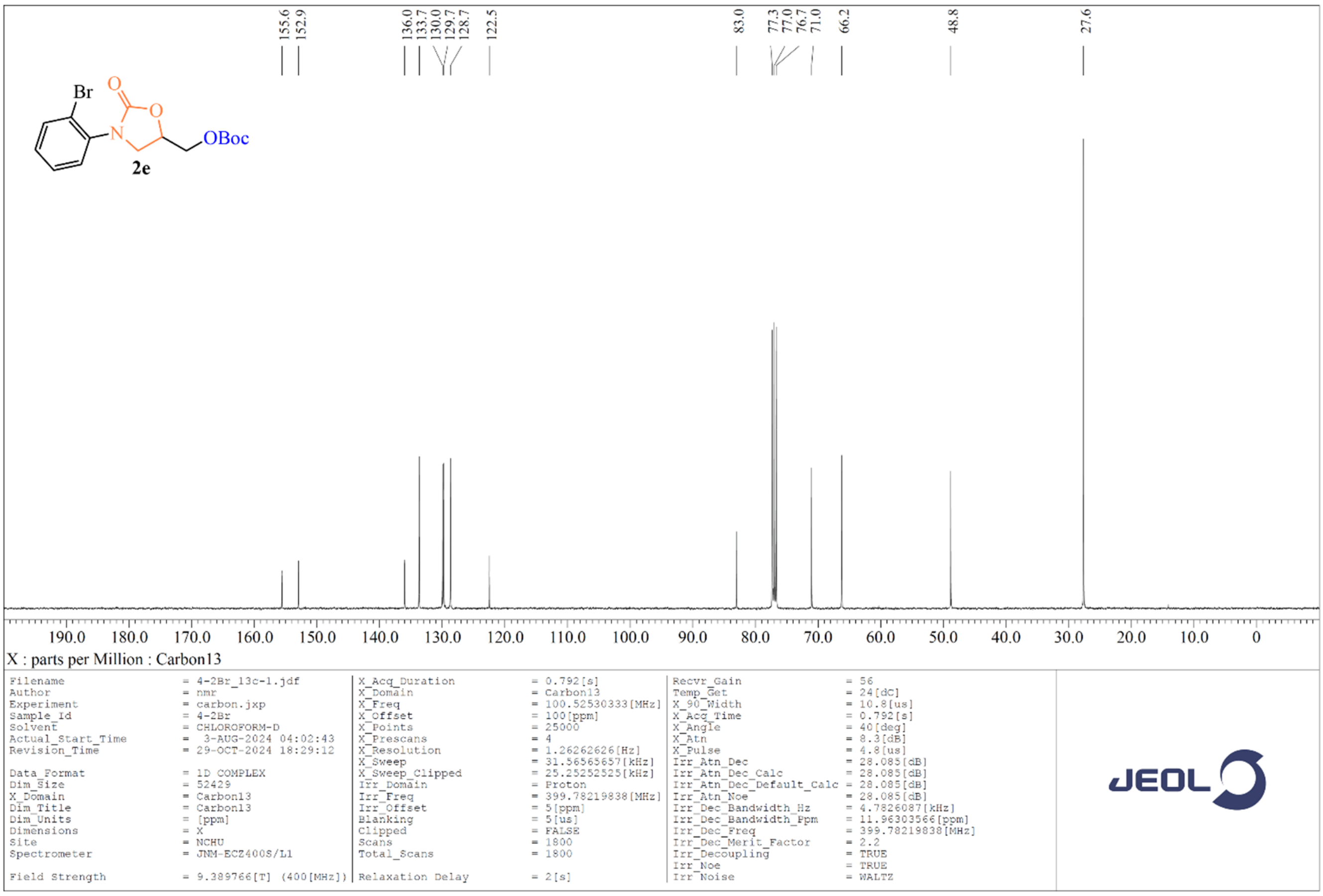


$^{13}$C NMR spectrum of compound **2e** (101 MHz, $CDCl_3$)

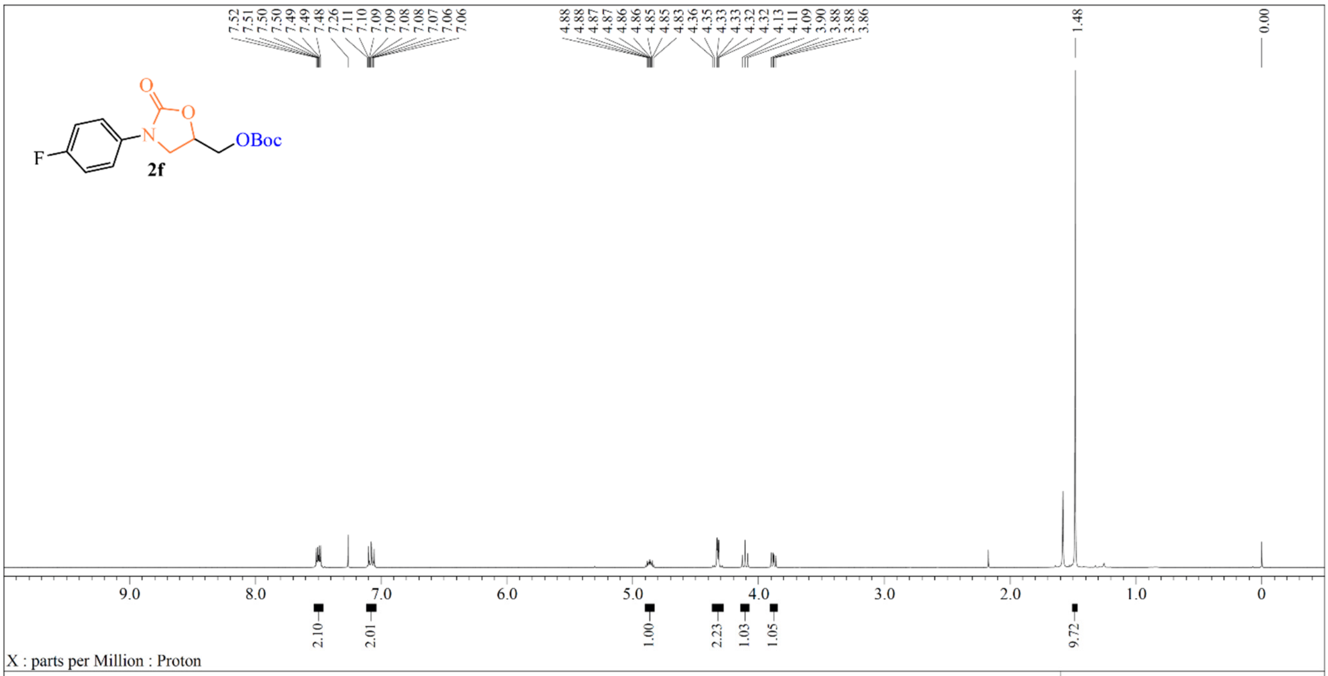


```
Filename            = 4-4F_1H-1.jdf            X_Acq_Duration     = 2.39616[s]           Total_Scans        = 8
Author              = nmr                      X_Domain           = Proton
Experiment          = proton.jxp               X_Freq             = 399.78219838[MHz]    Relaxation_Delay   = 2[s]
Sample_Id           = 4-4F                     X_Offset           = 5[ppm]               Recvr_Gain         = 56
Solvent             = CHLOROFORM-D             X_Points           = 24000                Temp_Get           = 22.7[dC]
Actual_Start_Time   = 11-OCT-2024 03:12:45     X_Prescans         = 1                    X_90_Width         = 8.54[us]
Revision_Time       = 29-OCT-2024 07:40:33     X_Resolution       = 0.4173344[Hz]        X_Acq_Time         = 2.39616[s]
                                               X_Sweep            = 10.01602564[kHz]     X_Angle            = 45[deg]
Data_Format         = 1D COMPLEX               X_Sweep_Clipped    = 8.01282051[kHz]      X_Atn              = 5.5[dB]
Dim_Size            = 52429                    Irr_Domain         = Proton               X_Pulse            = 4.27[us]
X_Domain            = Proton                   Irr_Freq           = 399.78219838[MHz]    Irr_Mode           = Off
Dim_Title           = Proton                   Irr_Offset         = 5[ppm]               Tri_Mode           = Off
Dim_Units           = [ppm]                    Tri_Domain         = Proton               Dante_Loop         = 200
Dimensions          = X                        Tri_Freq           = 399.78219838[MHz]    Dante_Presat       = FALSE
Site                = NCHU                     Tri_Offset         = 5[ppm]               Decimation_Rate    = 0
Spectrometer        = JNM-ECZ400S/L1           Blanking           = 2[us]                Initial_Wait       = 1[s]
                                               Clipped            = FALSE                Phase              = {0, 90, 270, 180, 180, 270, 90, 0}
Field Strength      = 9.389766[T] (400[MHz])   Scans              = 8                    Presat Time        = 2[s]
```

$^{1}$H NMR spectrum of compound **2f** (400 MHz, $CDCl_3$)

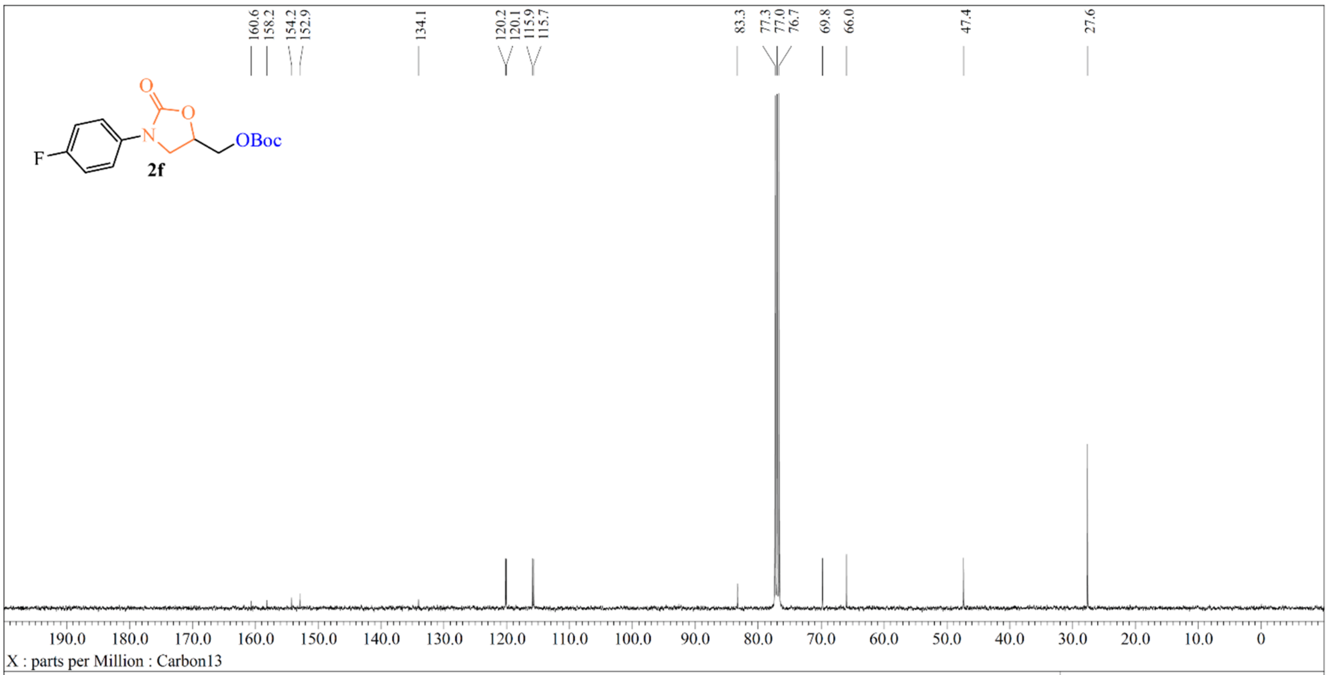


```
Filename            = 4-4F_13C-1.jdf           X_Acq_Duration     = 0.792[s]             Recvr_Gain                 = 56
Author              = nmr                      X_Domain           = Carbon13             Temp_Get                   = 22.7[dC]
Experiment          = carbon.jxp               X_Freq             = 100.52530333[MHz]    X_90_Width                 = 10.8[us]
Sample_Id           = 4-4F                     X_Offset           = 100[ppm]             X_Acq_Time                 = 0.792[s]
Solvent             = CHLOROFORM-D             X_Points           = 25000                X_Angle                    = 40[deg]
Actual_Start_Time   = 11-OCT-2024 03:18:29     X_Prescans         = 4                    X_Atn                      = 8.3[dB]
Revision_Time       = 29-OCT-2024 18:35:38     X_Resolution       = 1.26262626[Hz]       X_Pulse                    = 4.8[us]
                                               X_Sweep            = 31.56565657[kHz]     Irr_Atn_Dec                = 28.085[dB]
Data_Format         = 1D COMPLEX               X_Sweep_Clipped    = 25.25252525[kHz]     Irr_Atn_Dec_Calc           = 28.085[dB]
Dim_Size            = 52429                    Irr_Domain         = Proton               Irr_Atn_Dec_Default_Calc   = 28.085[dB]
X_Domain            = Carbon13                 Irr_Freq           = 399.78219838[MHz]    Irr_Atn_Noe                = 28.085[dB]
Dim_Title           = Carbon13                 Irr_Offset         = 5[ppm]               Irr_Dec_Bandwidth_Hz       = 4.7826087[kHz]
Dim_Units           = [ppm]                    Blanking           = 5[us]                Irr_Dec_Bandwidth_Ppm      = 11.96303566[ppm]
Dimensions          = X                        Clipped            = TRUE                 Irr_Dec_Freq               = 399.78219838[MHz]
Site                = NCHU                     Scans              = 1800                 Irr_Dec_Merit_Factor       = 2.2
Spectrometer        = JNM-ECZ400S/L1           Total_Scans        = 1800                 Irr_Decoupling             = TRUE
                                                                                         Irr_Noe                    = TRUE
Field Strength      = 9.389766[T] (400[MHz])   Relaxation Delay   = 2[s]                 Irr Noise                  = WALTZ
```

$^{13}$C NMR spectrum of compound **2f** (101 MHz, $CDCl_3$)

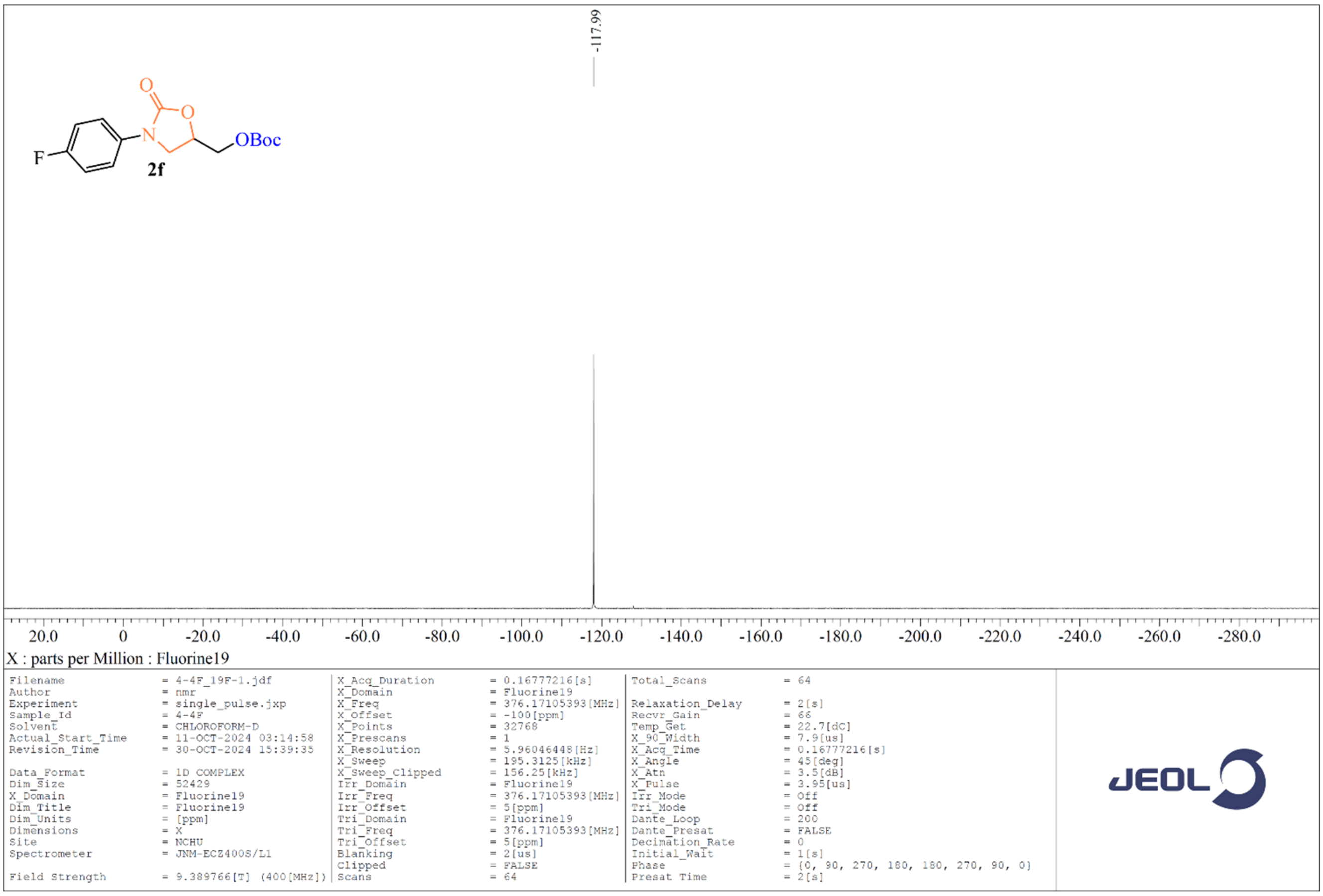


$^{19}$F NMR spectrum of compound **2f** (376 MHz, CDCl$_3$)

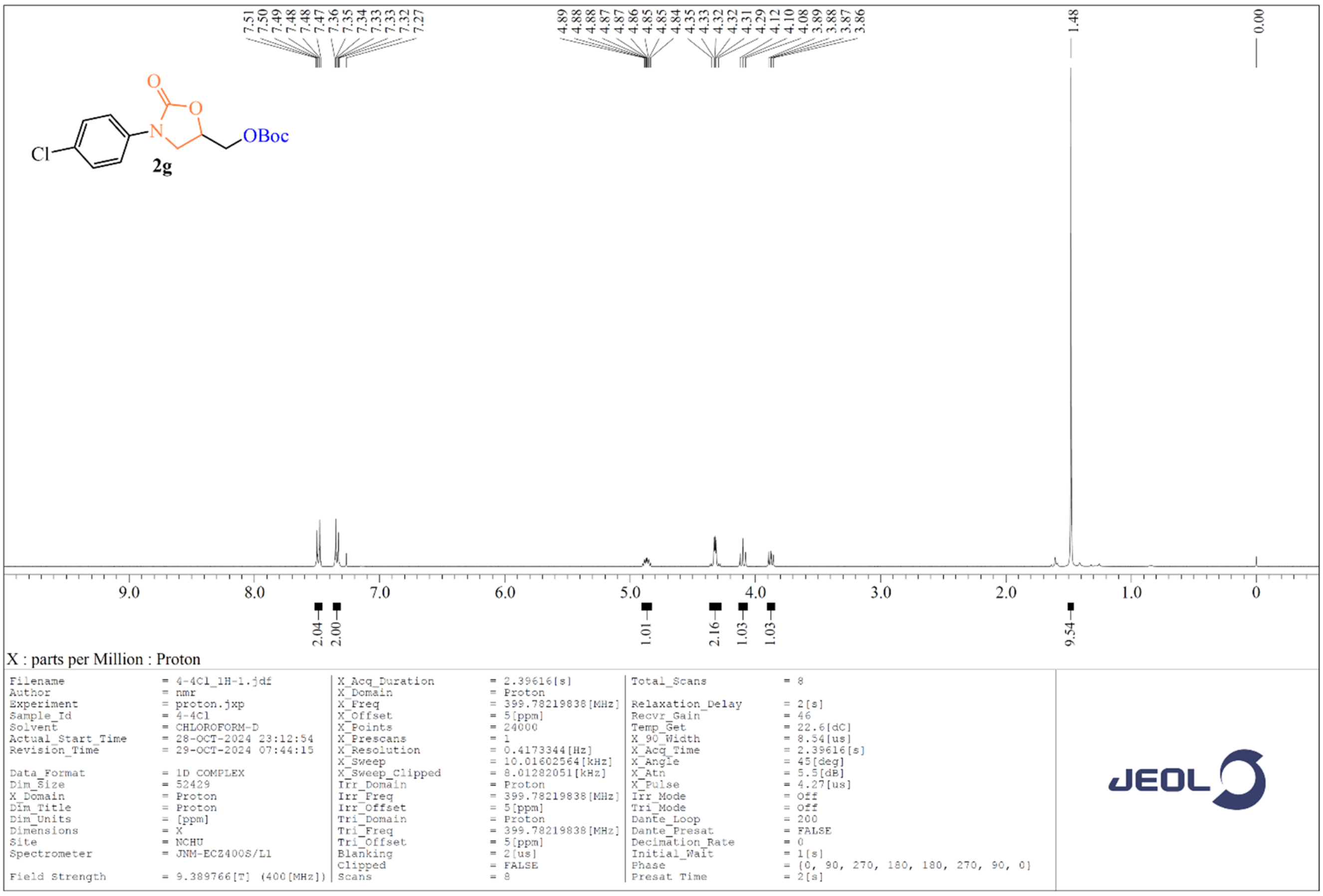


$^{1}$H NMR spectrum of compound **2g** (400 MHz, CDCl$_3$)

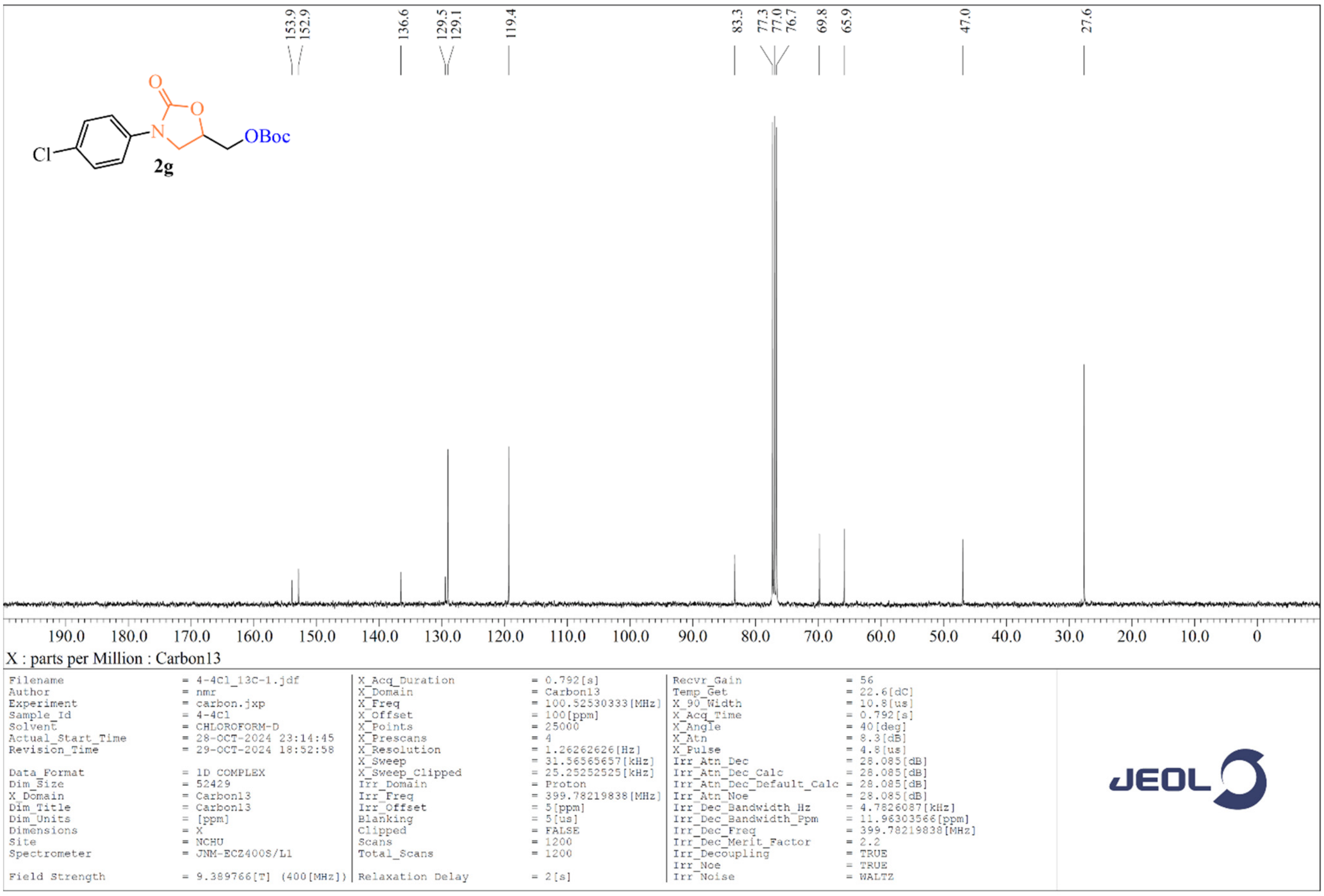


$^{13}$C NMR spectrum of compound **2g** (101 MHz, $CDCl_3$)

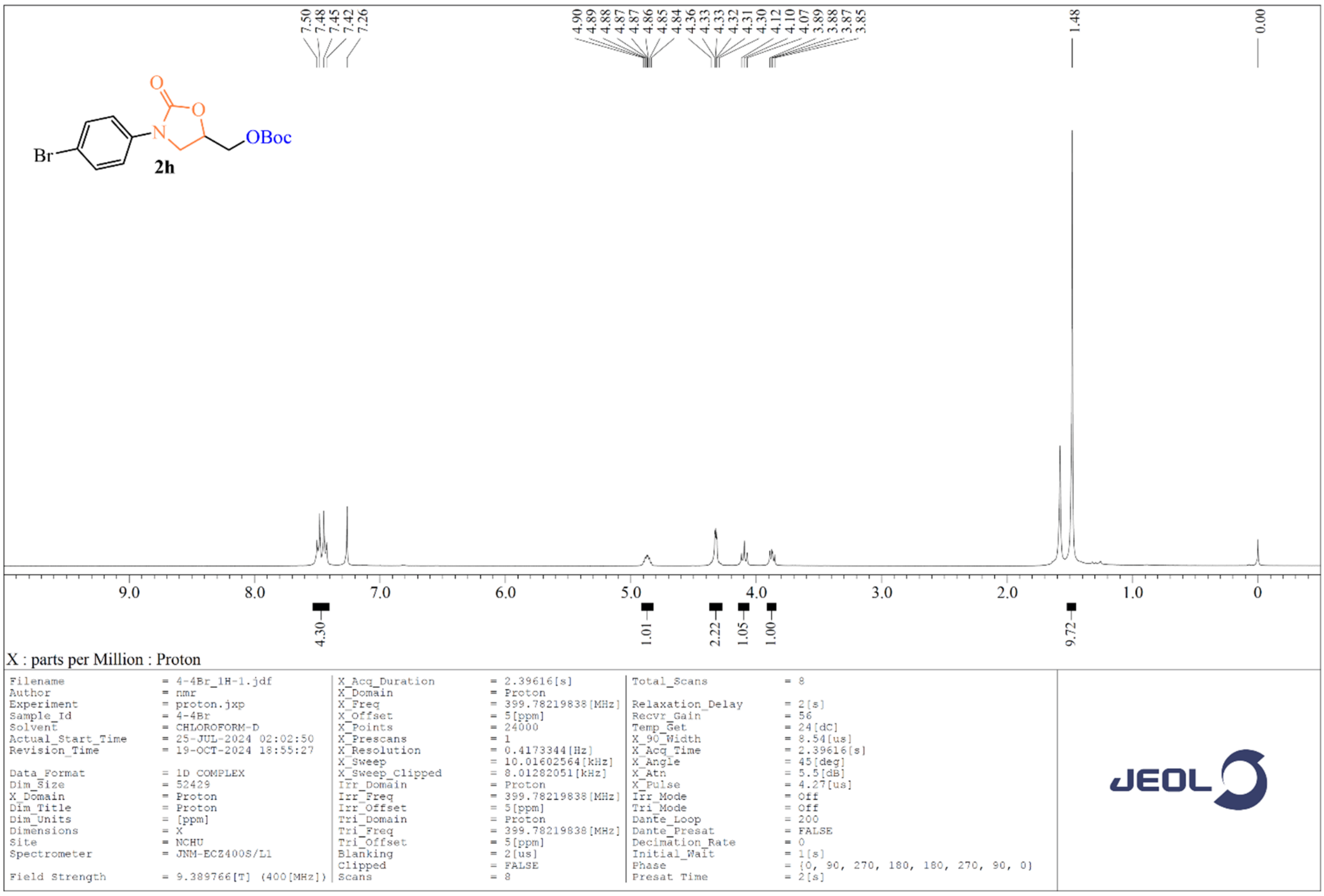


$^{1}$H NMR spectrum of compound **2h** (400 MHz, $CDCl_3$)

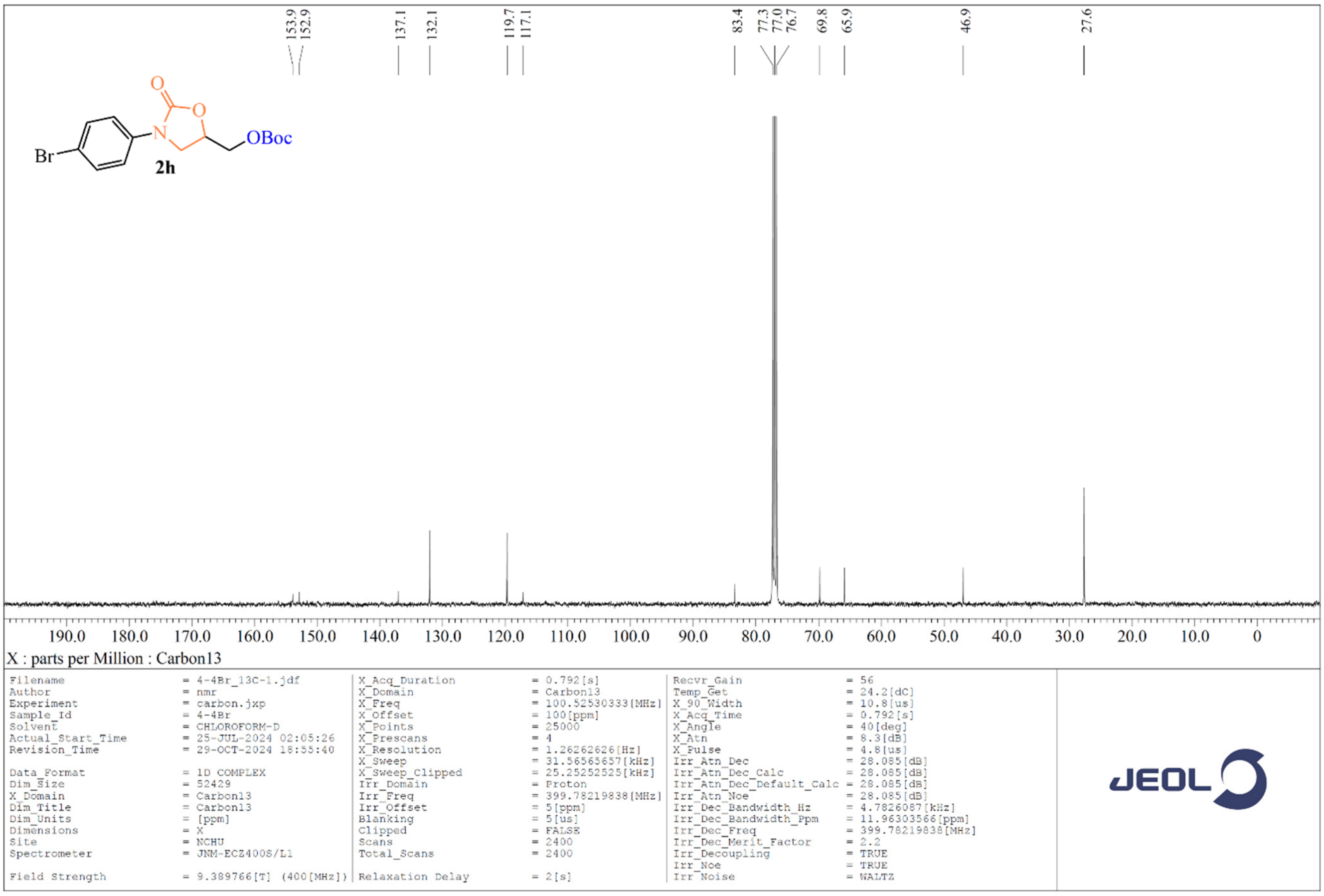


$^{13}$C NMR spectrum of compound **2h** (101 MHz, $CDCl_3$)

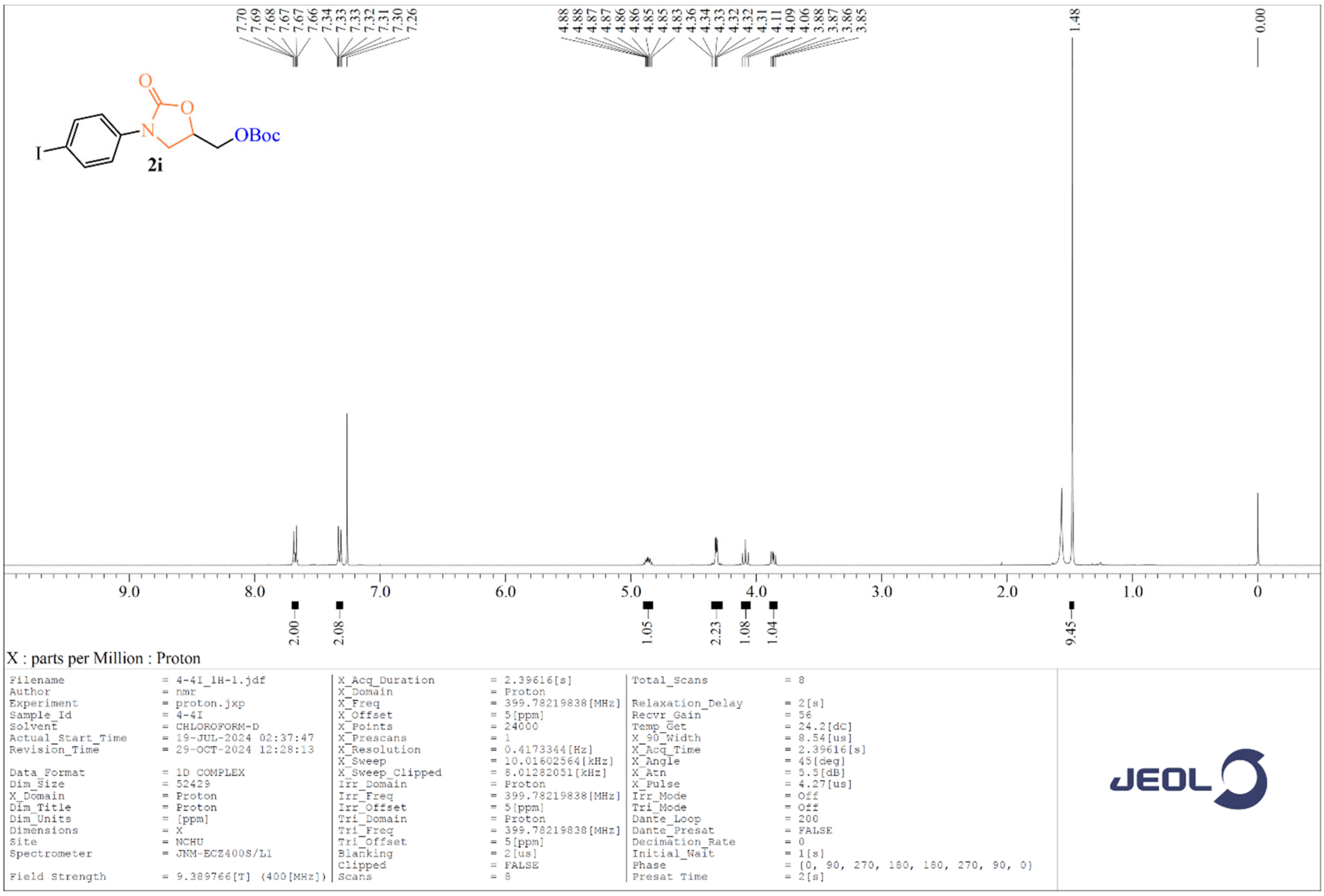


$^{1}$H NMR spectrum of compound **2i** (400 MHz, $CDCl_3$)

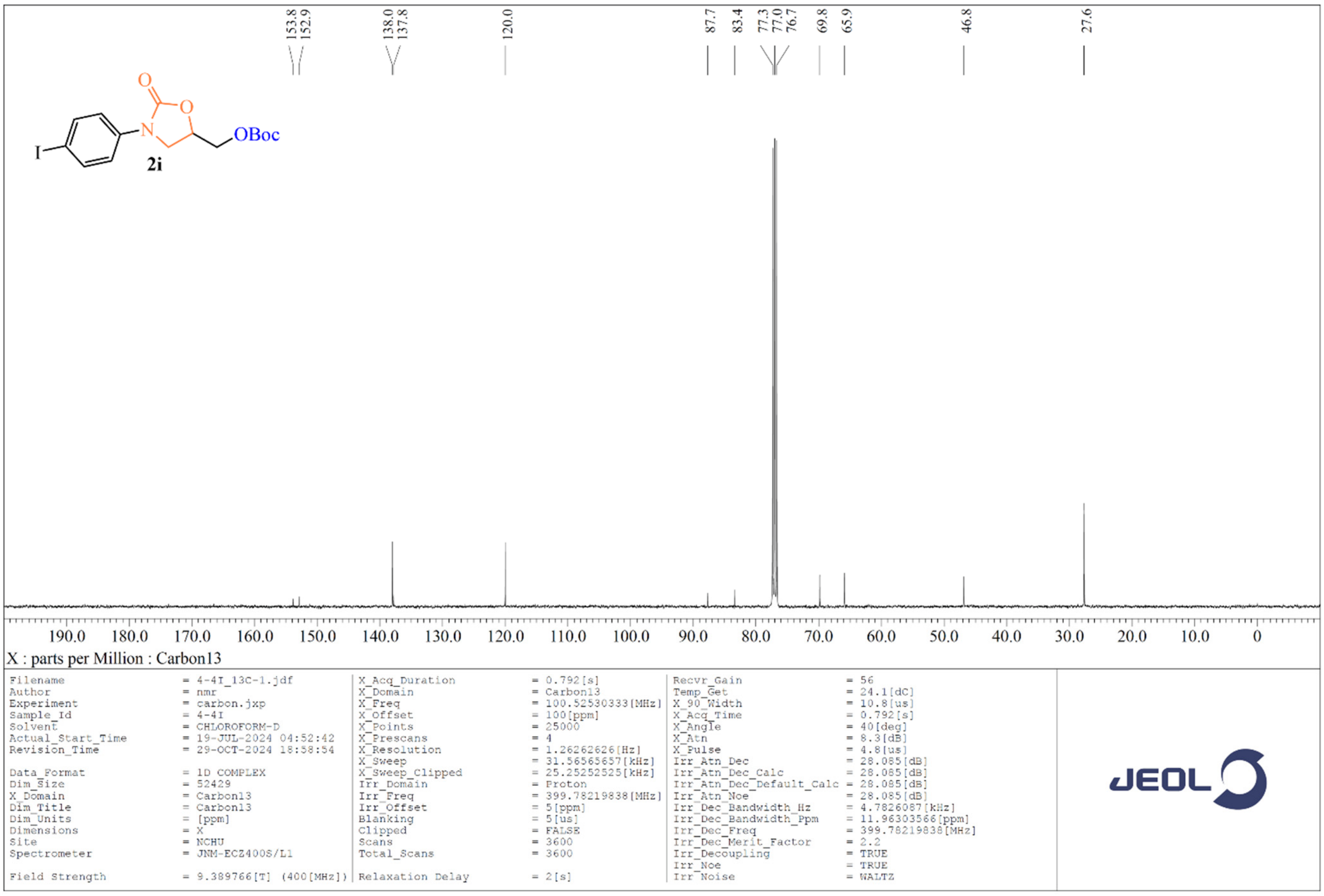


$^{13}$C NMR spectrum of compound **2i** (101 MHz, $CDCl_3$)

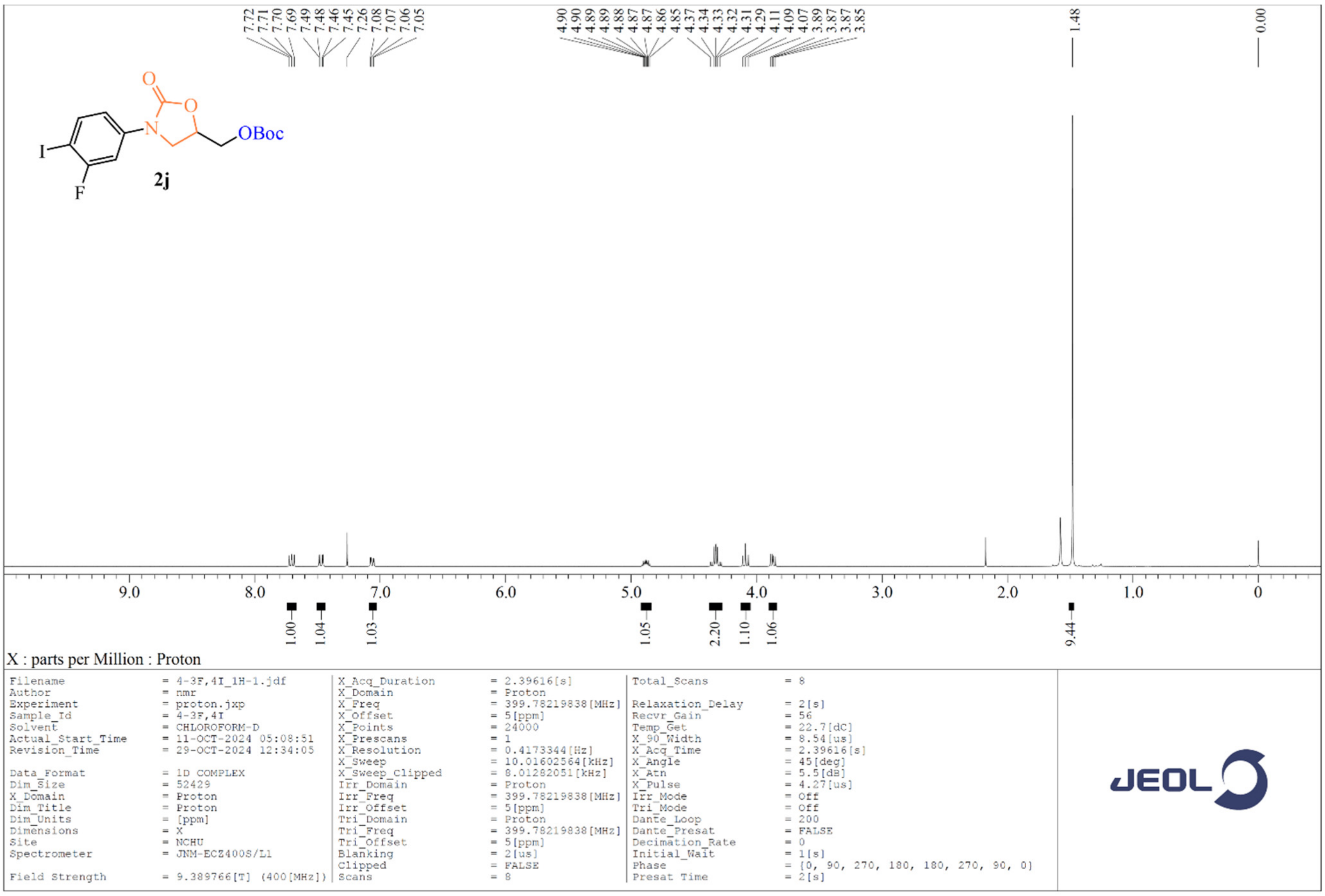


$^{1}$H NMR spectrum of compound **2j** (400 MHz, $CDCl_3$)

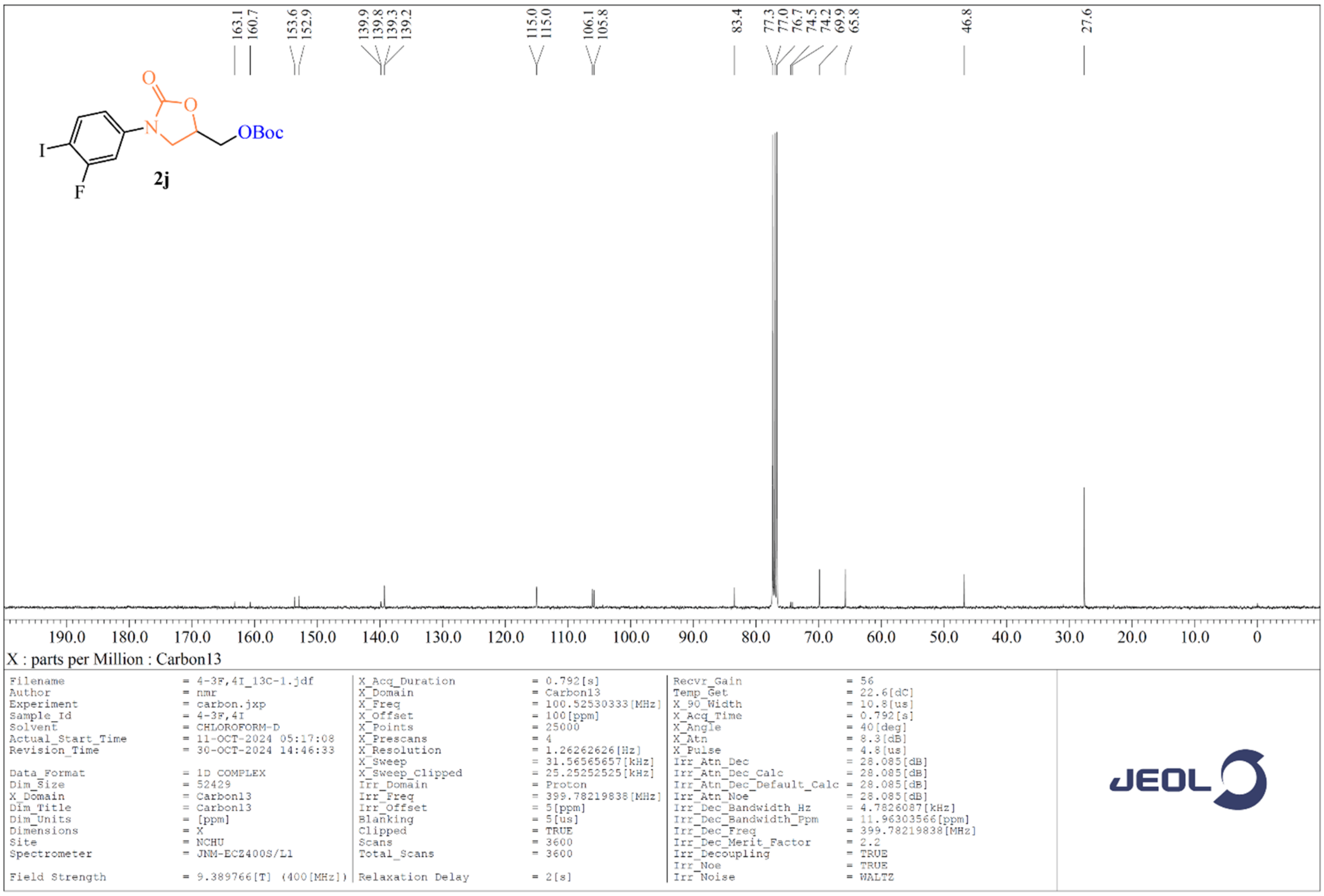


$^{13}C$ NMR spectrum of compound **2j** (101 MHz, $CDCl_3$)

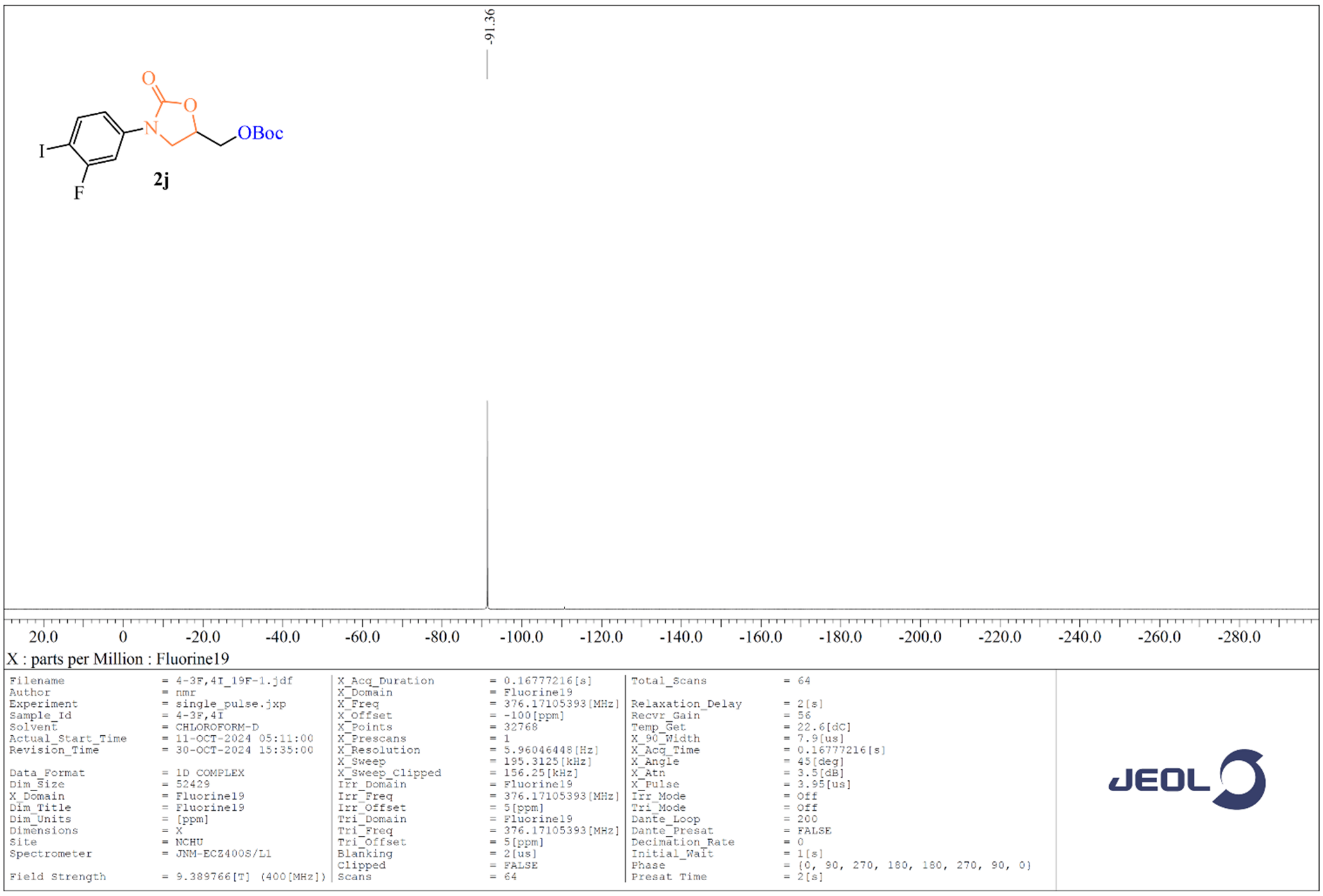


$^{19}F$ NMR spectrum of compound **2j** (376 MHz, $CDCl_3$)

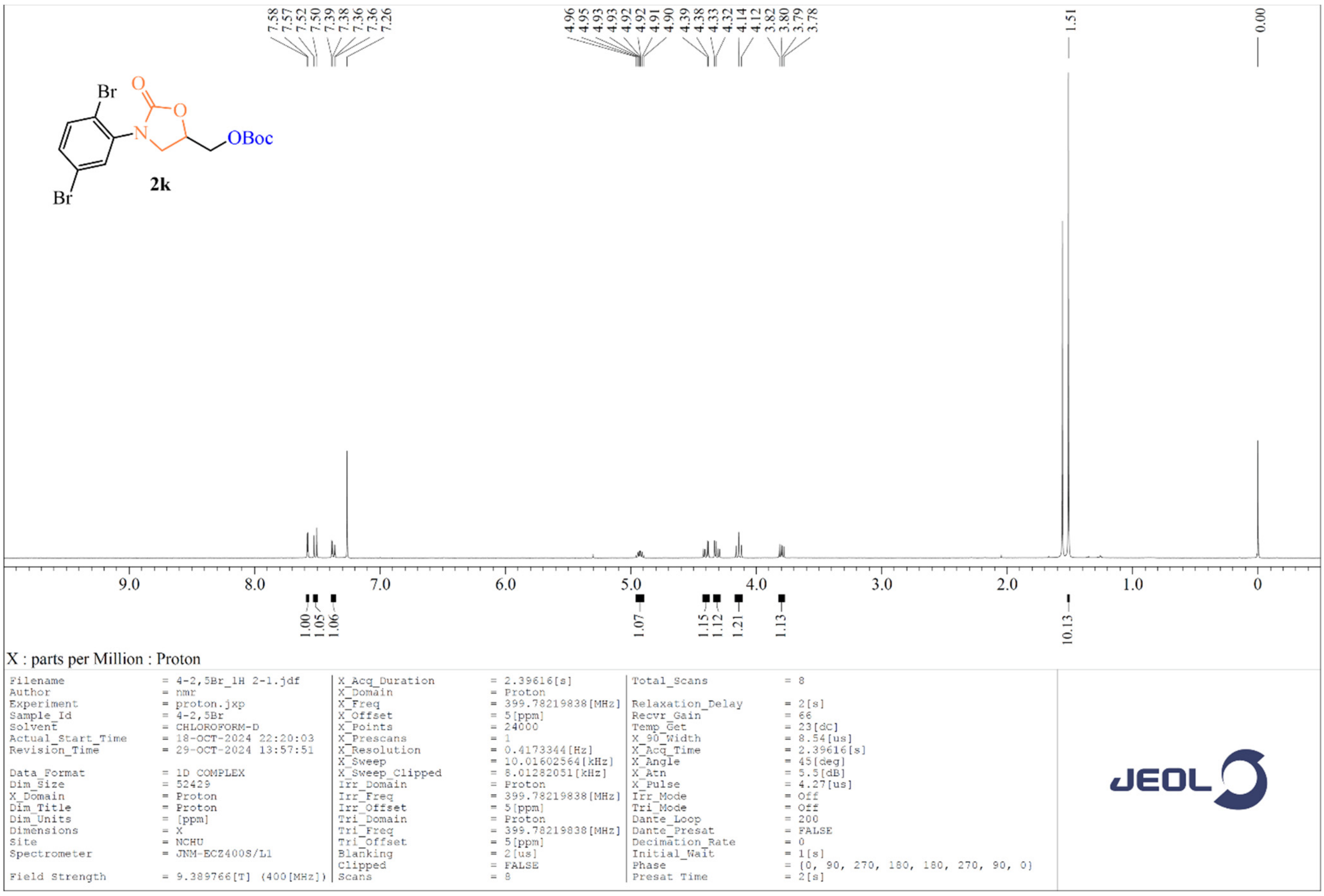


$^{1}$H NMR spectrum of compound **2k** (400 MHz, $CDCl_3$)

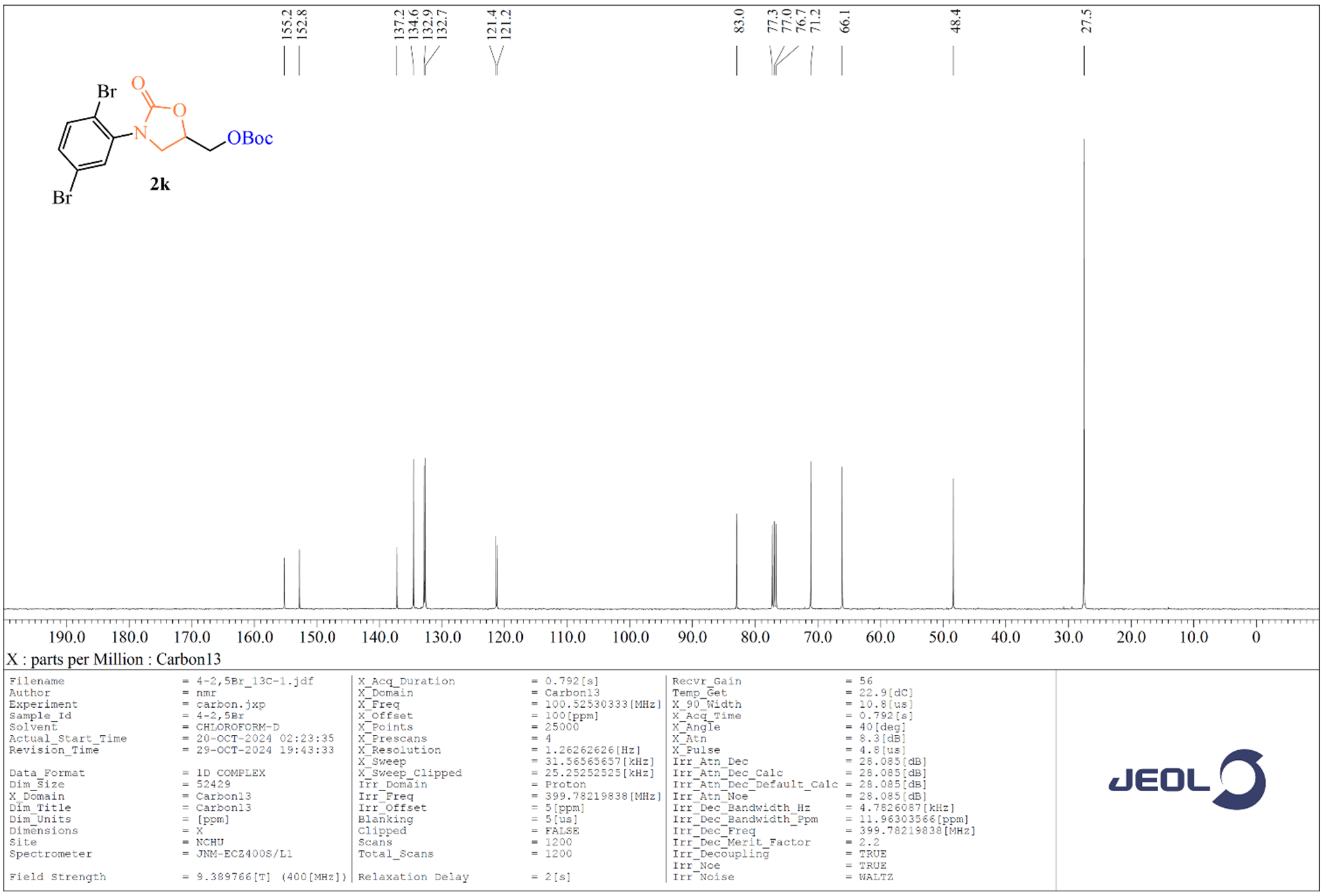


$^{13}$C NMR spectrum of compound **2k** (101 MHz, $CDCl_3$)

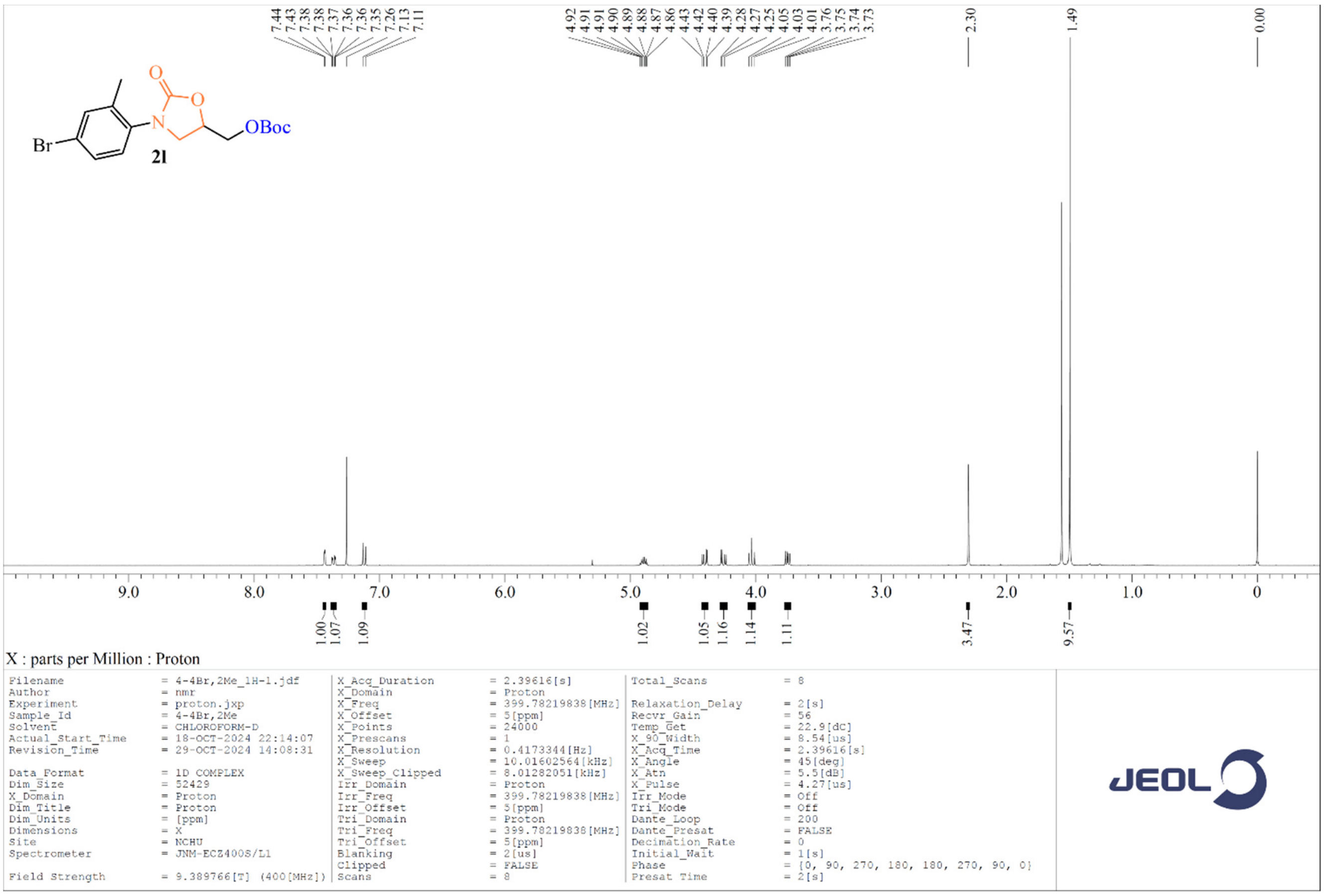


$^{1}$H NMR spectrum of compound **2l** (400 MHz, $CDCl_3$)

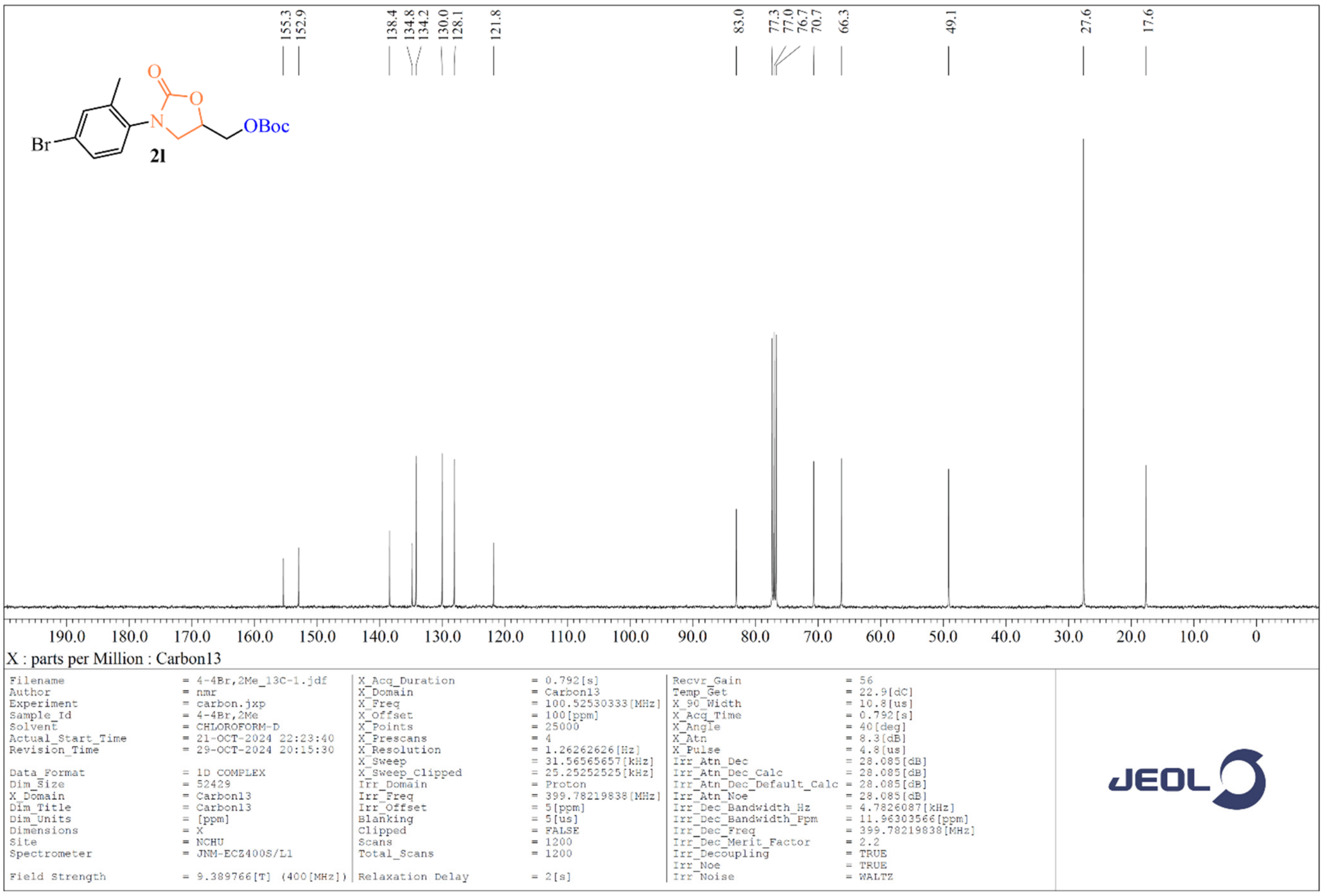


$^{13}$C NMR spectrum of compound **2l** (101 MHz, $CDCl_3$)

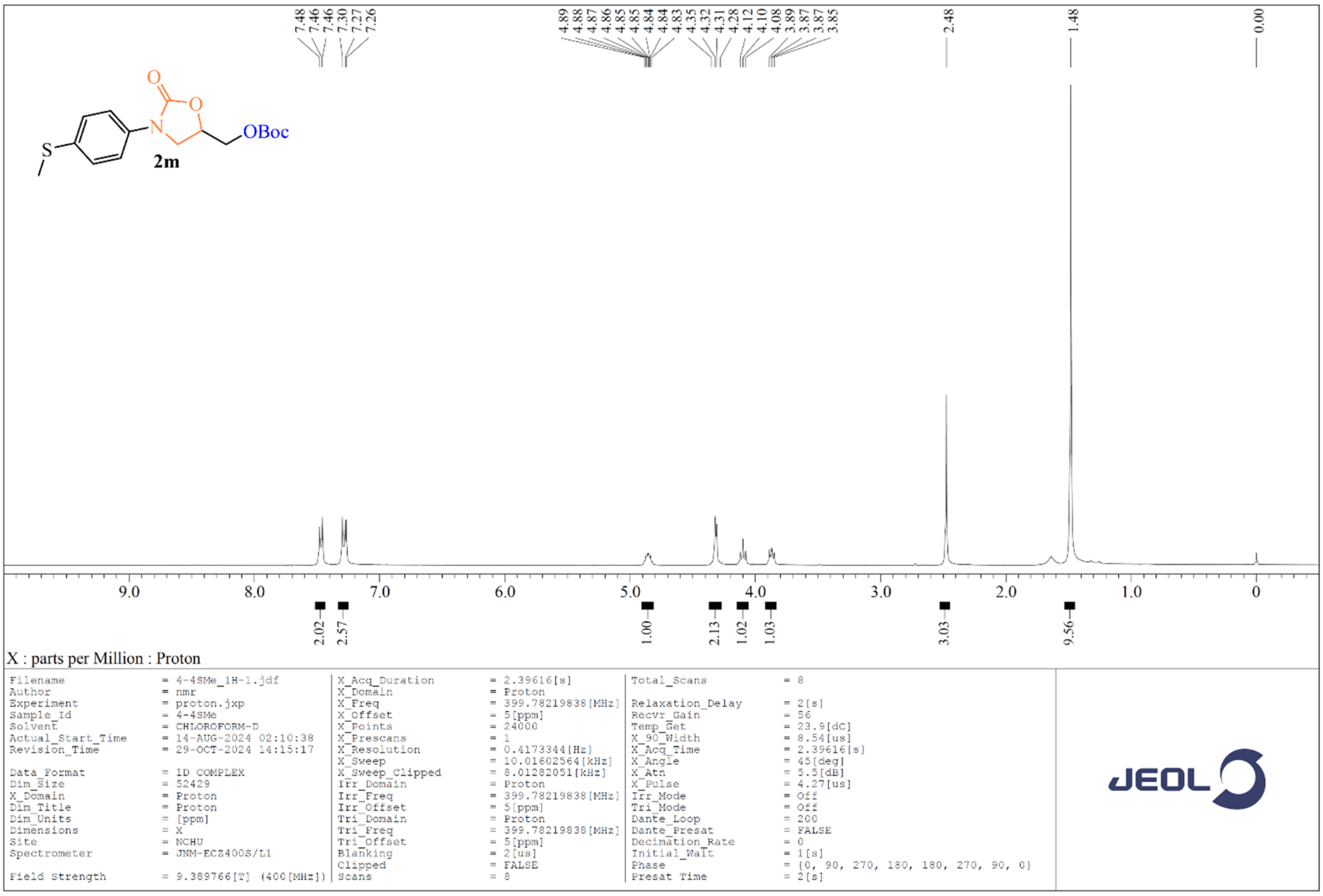


$^{1}H$ NMR spectrum of compound **2m** (400 MHz, $CDCl_3$)

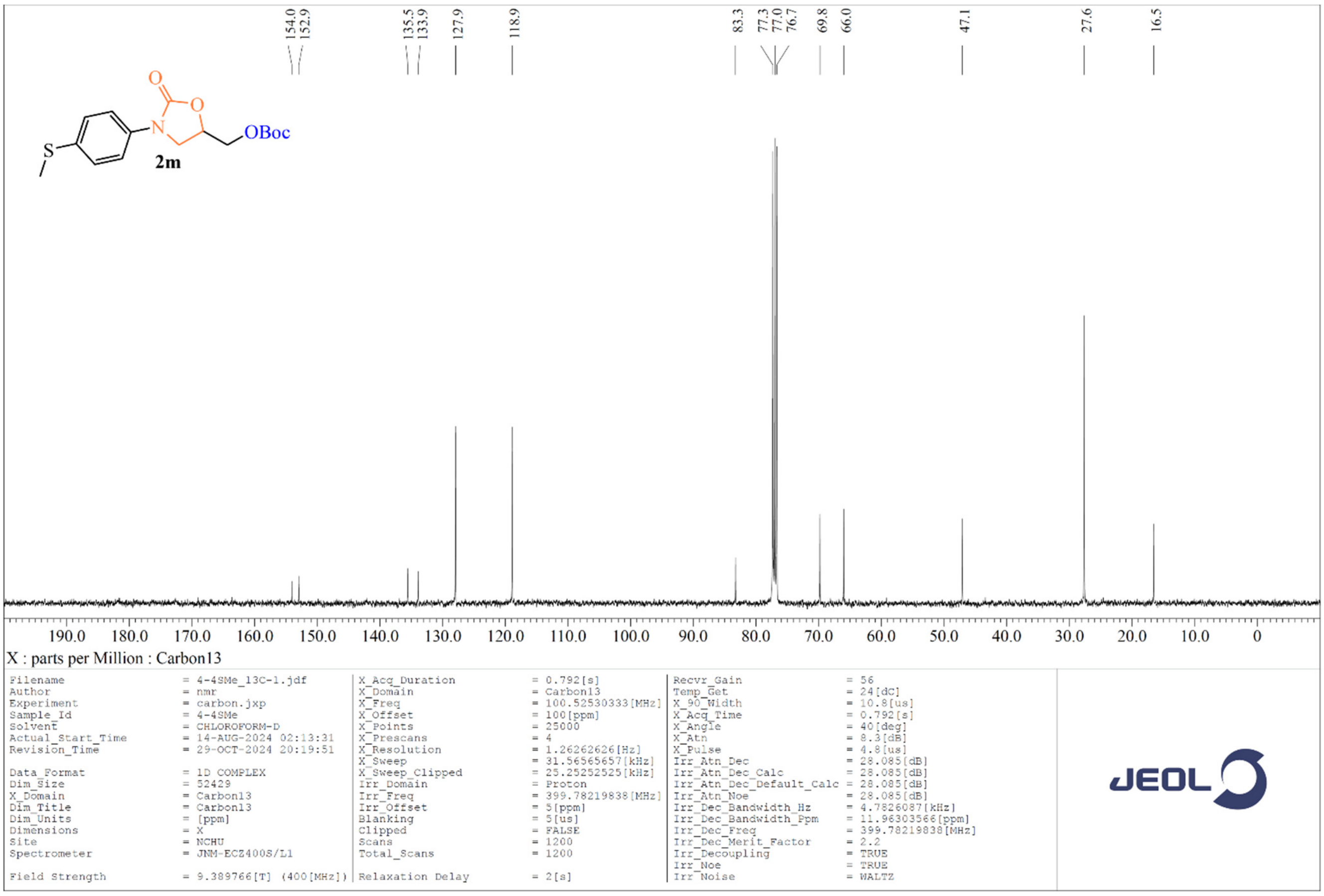


$^{13}C$ NMR spectrum of compound **2m** (101 MHz, $CDCl_3$)

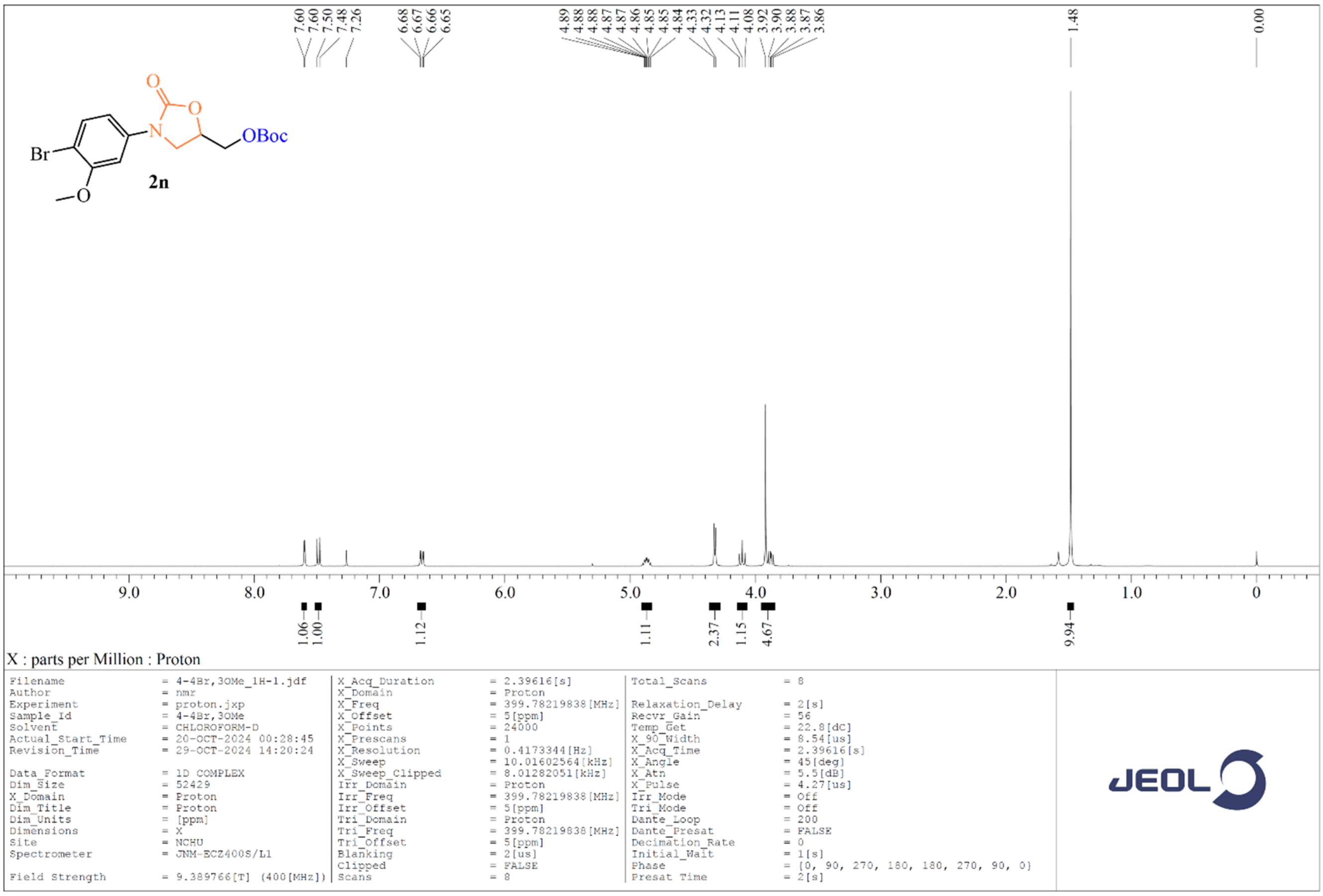


$^{1}$H NMR spectrum of compound **2n** (400 MHz, $CDCl_3$)

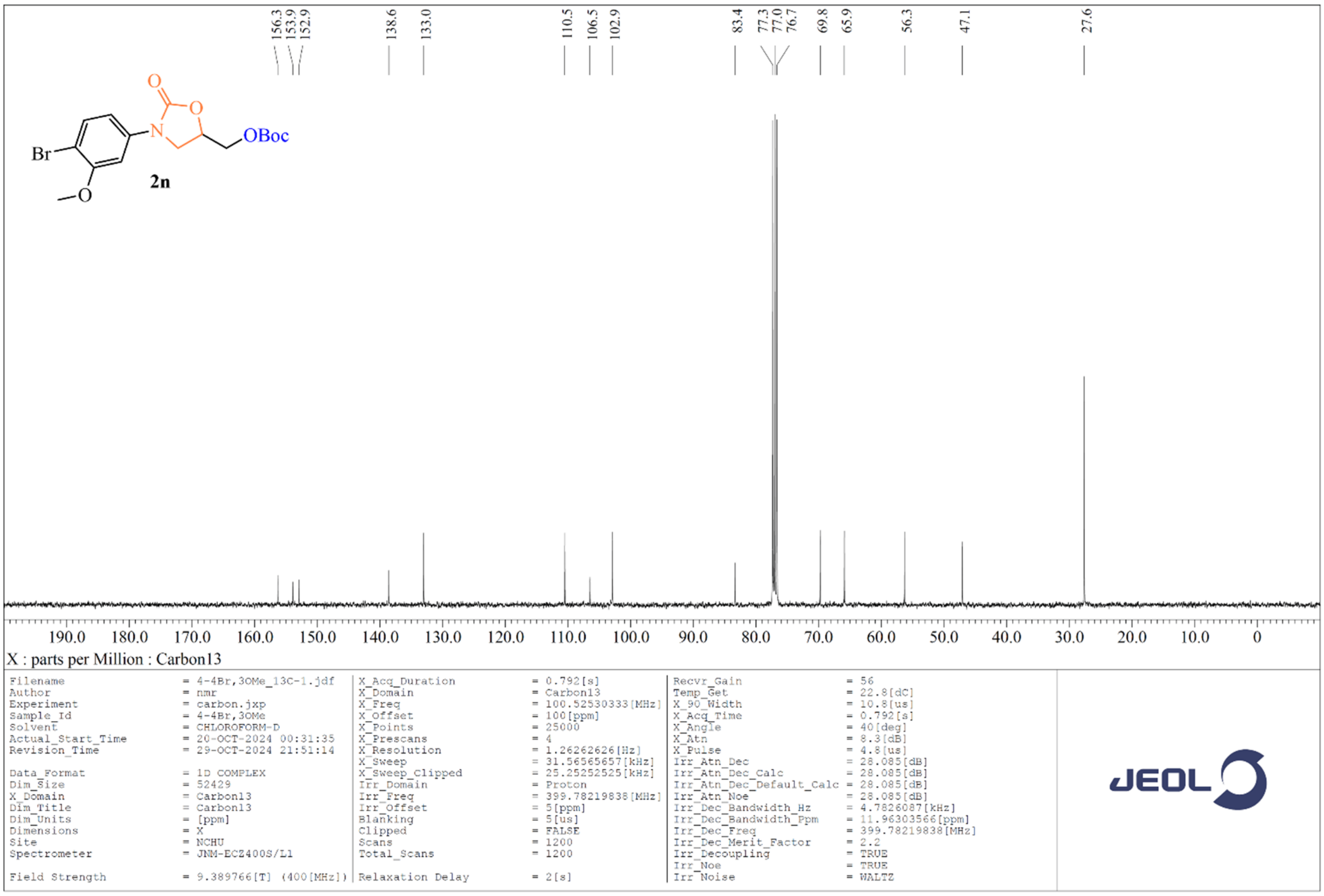


$^{13}$C NMR spectrum of compound **2n** (101 MHz, $CDCl_3$)

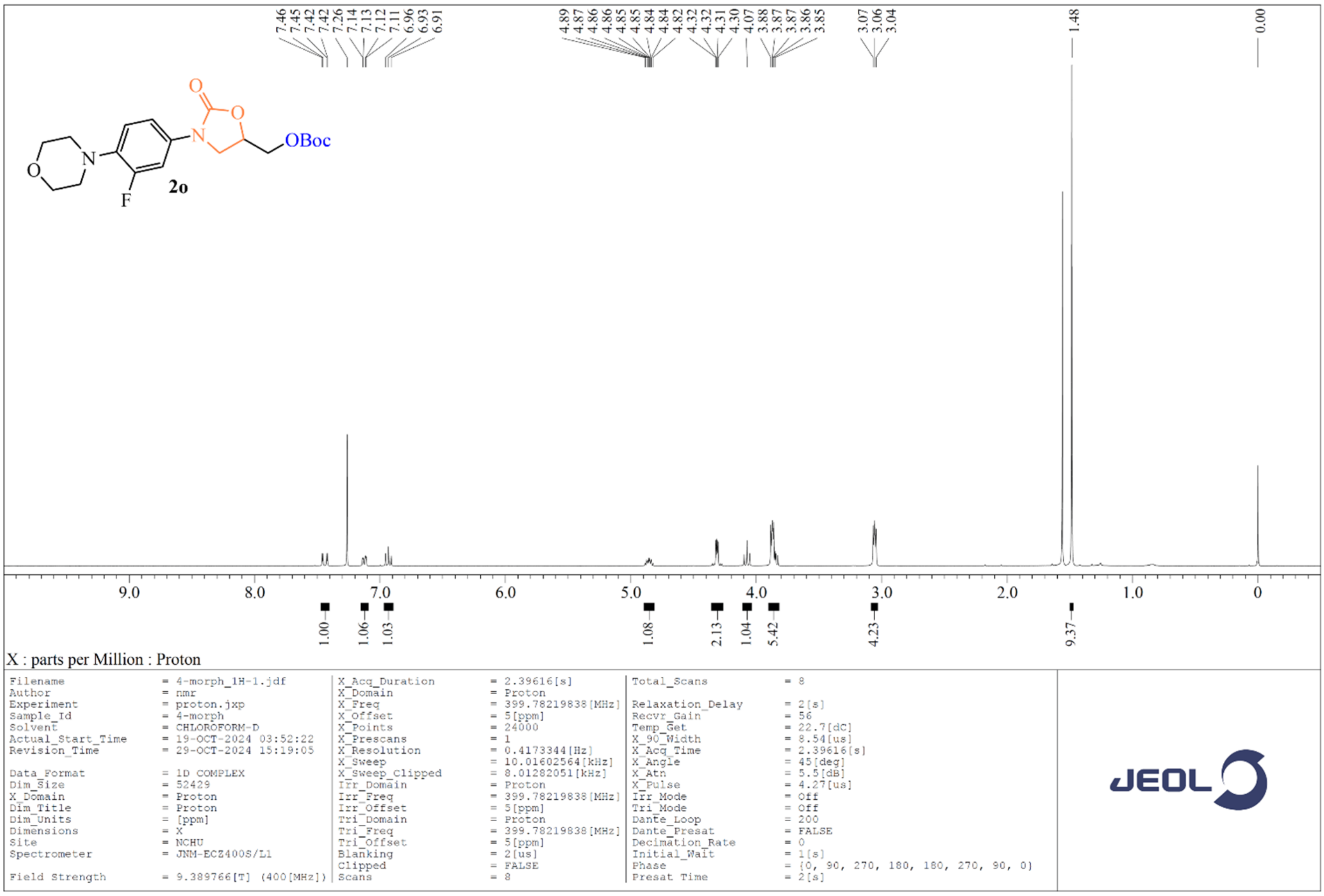


$^{1}$H NMR spectrum of compound **2o** (400 MHz, $CDCl_3$)

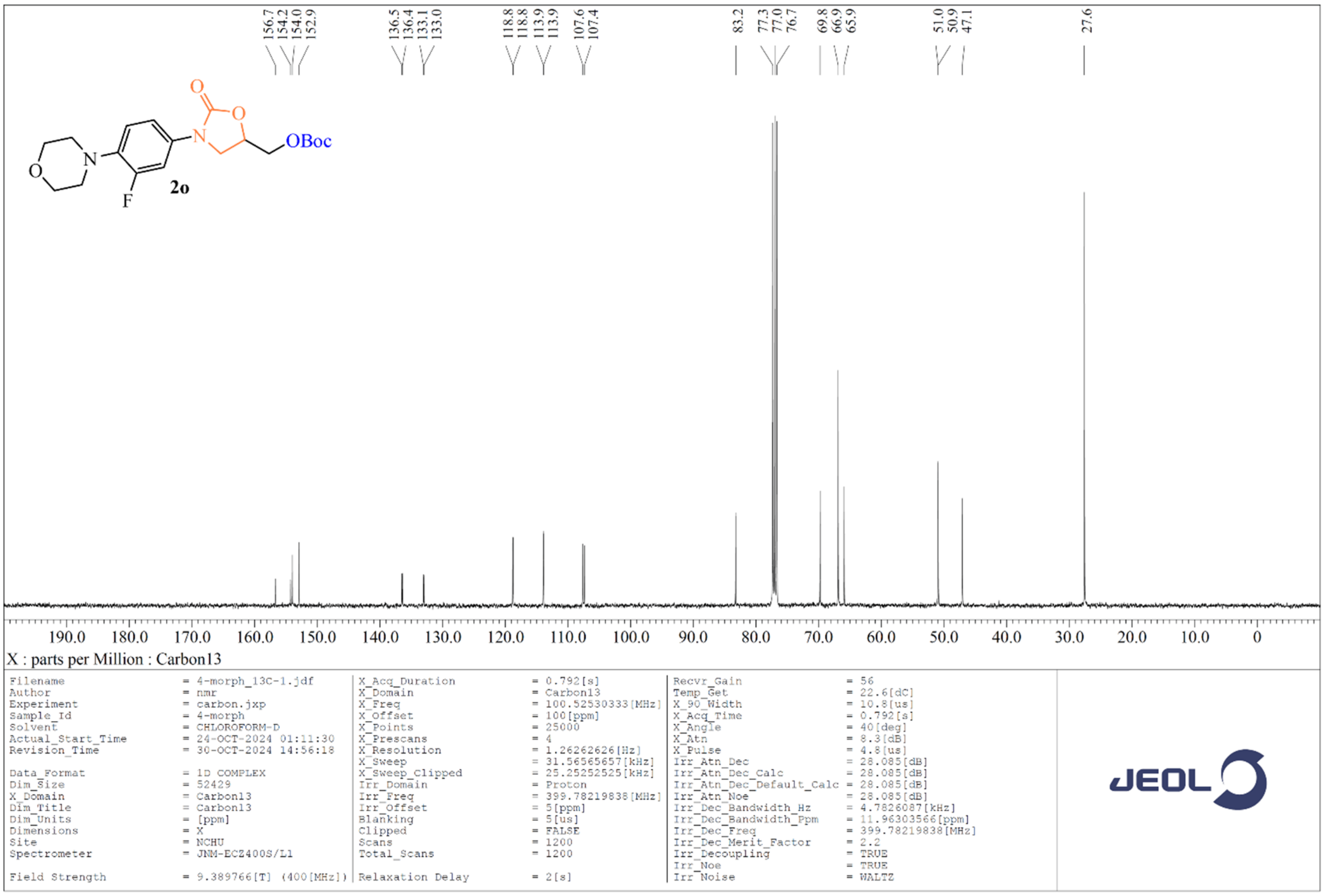


$^{13}$C NMR spectrum of compound **2o** (101 MHz, $CDCl_3$)

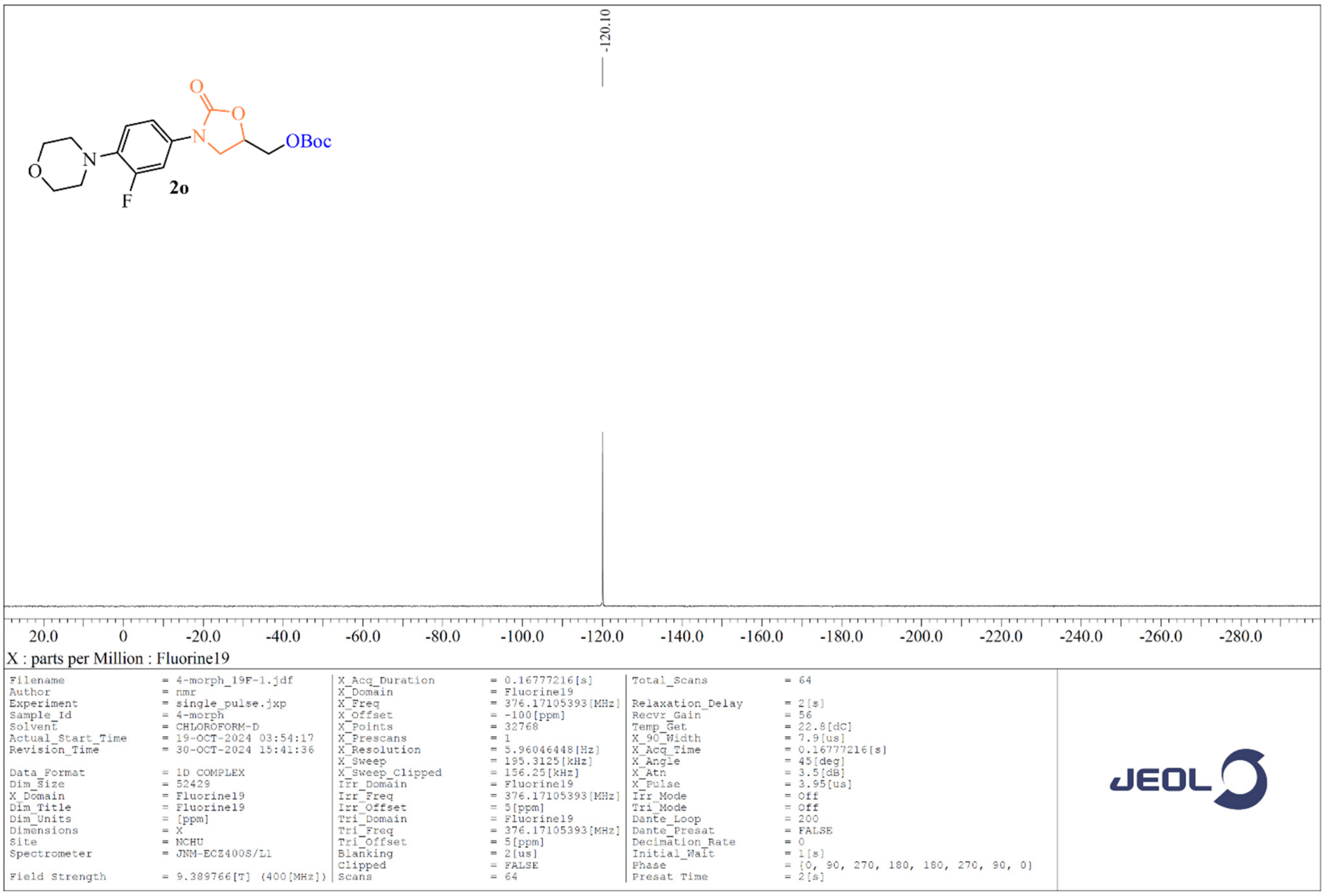


$^{19}F$ NMR spectrum of compound **2o** (376 MHz, $CDCl_3$)

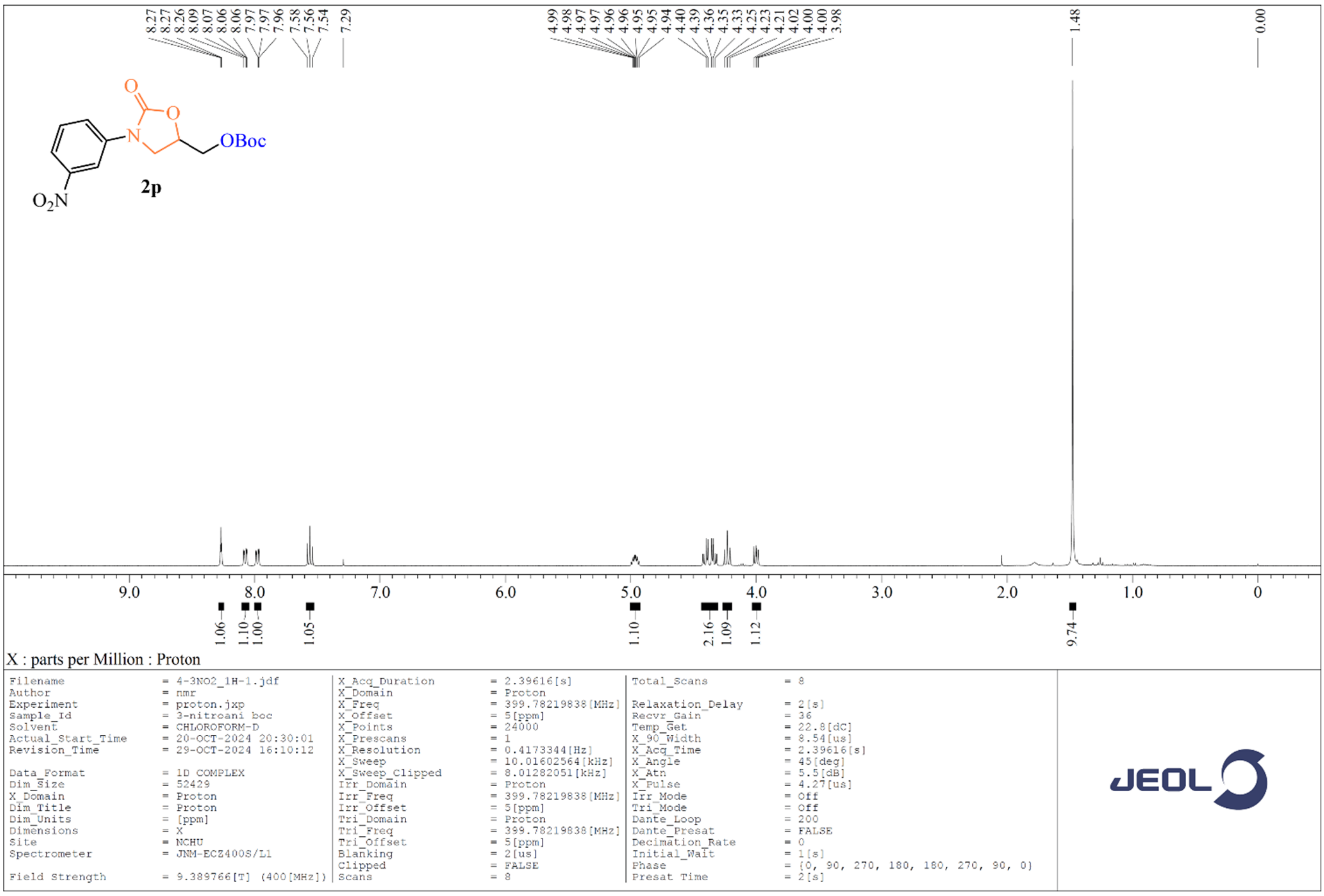


$^{1}H$ NMR spectrum of compound **2p** (400 MHz, $CDCl_3$)

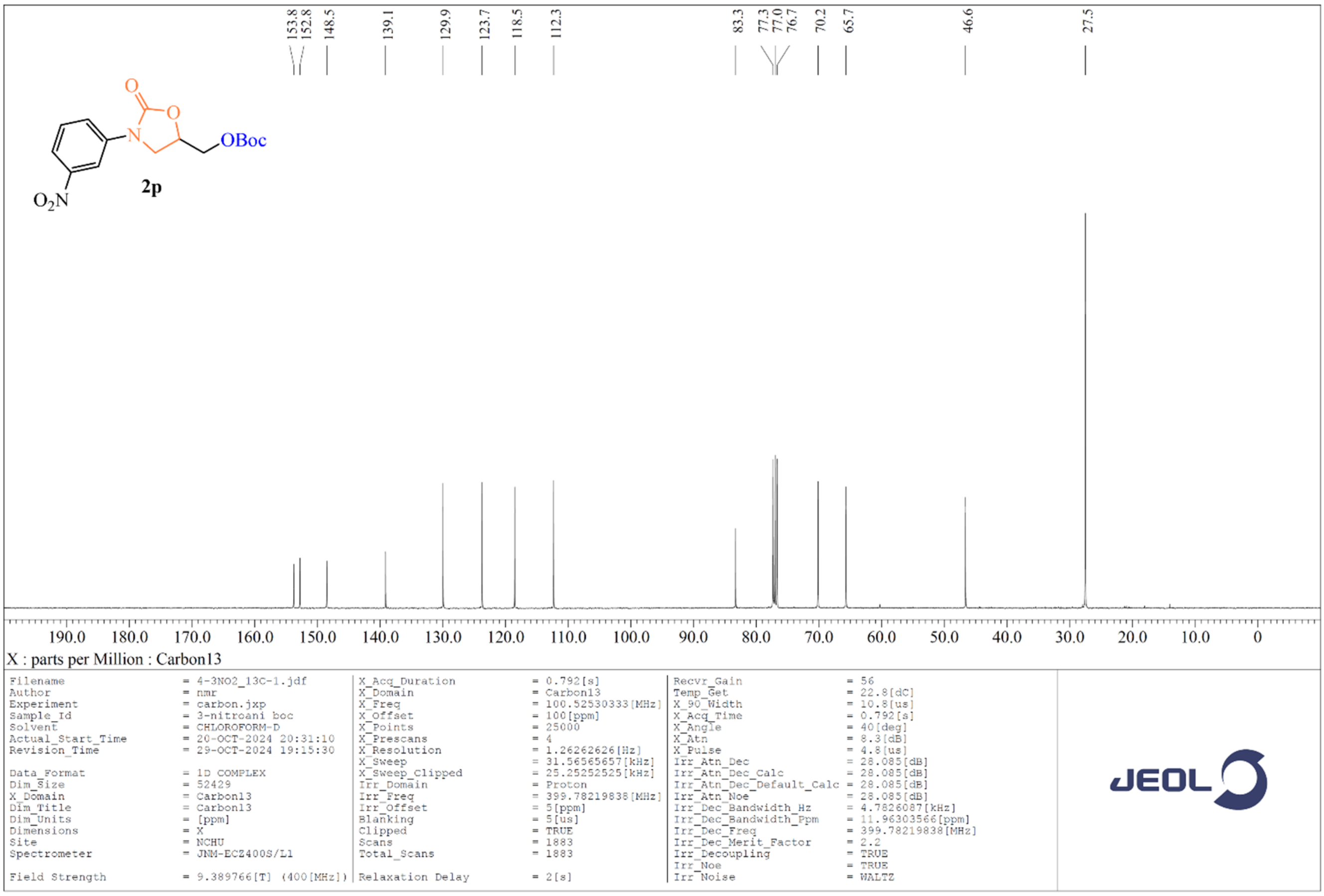


$^{13}C$ NMR spectrum of compound **2p** (101 MHz, $CDCl_3$)

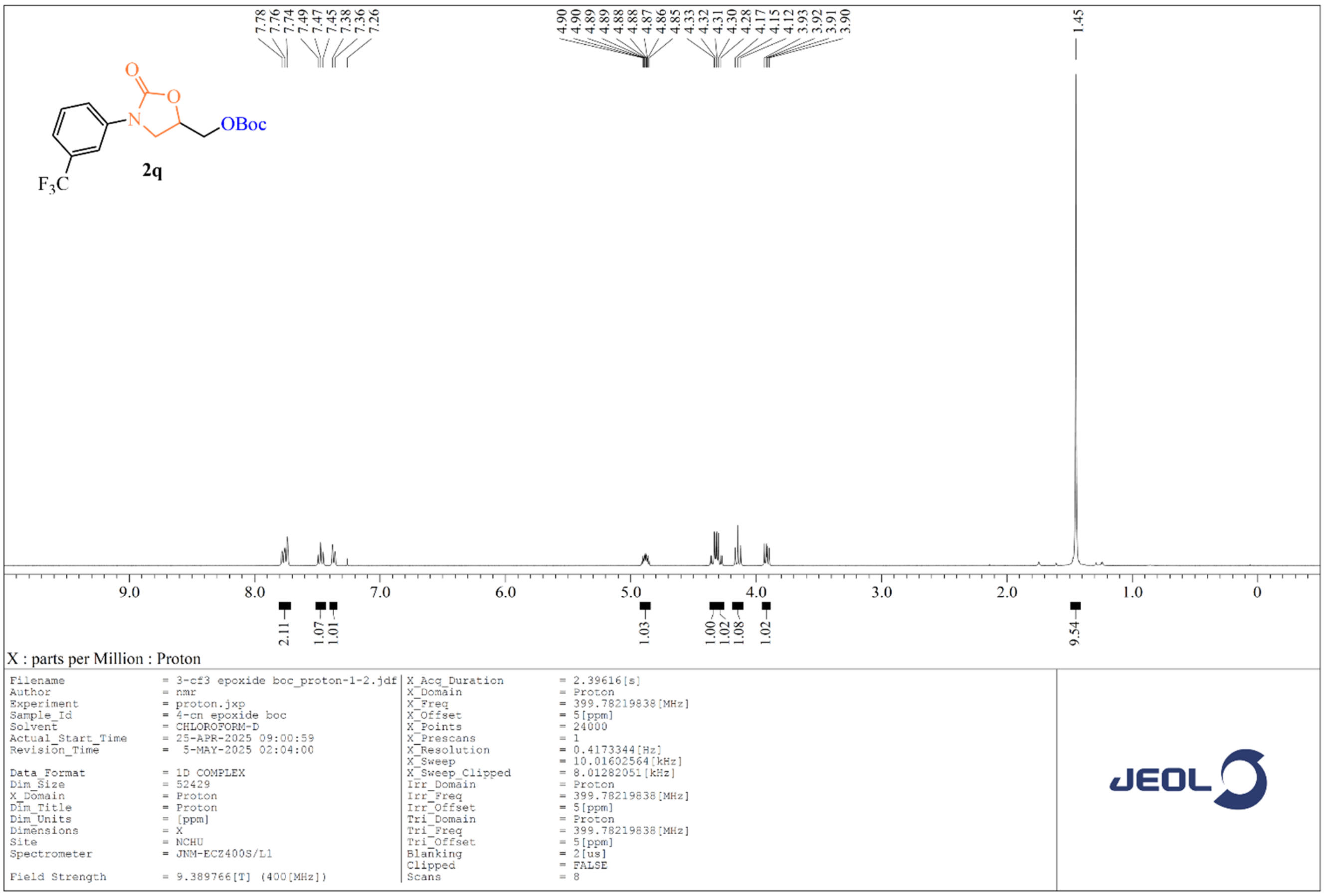


$^{1}H$ NMR spectrum of compound **2q** (400 MHz, $CDCl_3$)

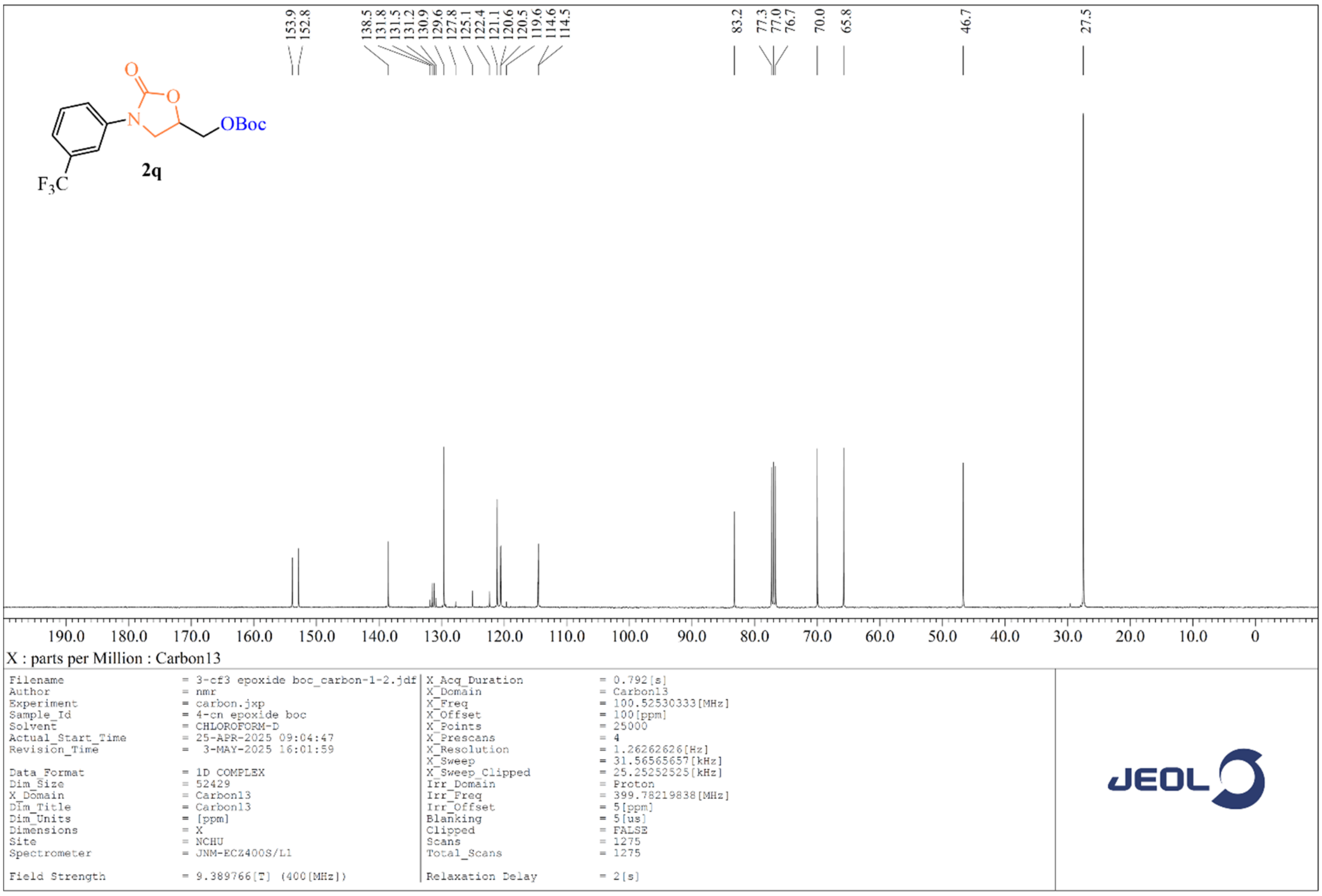


$^{13}$C NMR spectrum of compound **2q** (101 MHz, $CDCl_3$)

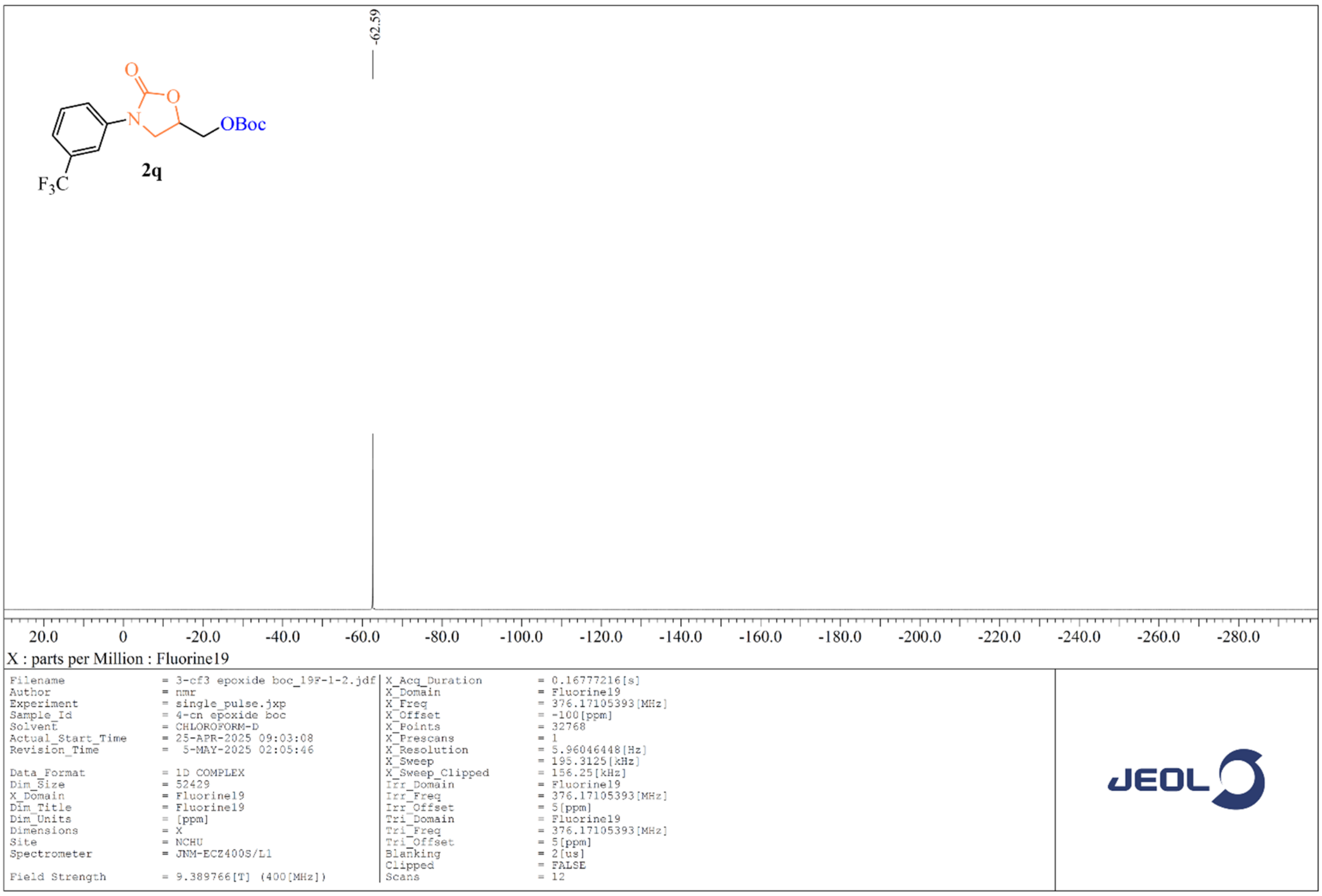


$^{19}$F NMR spectrum of compound **2q** (376 MHz, $CDCl_3$)

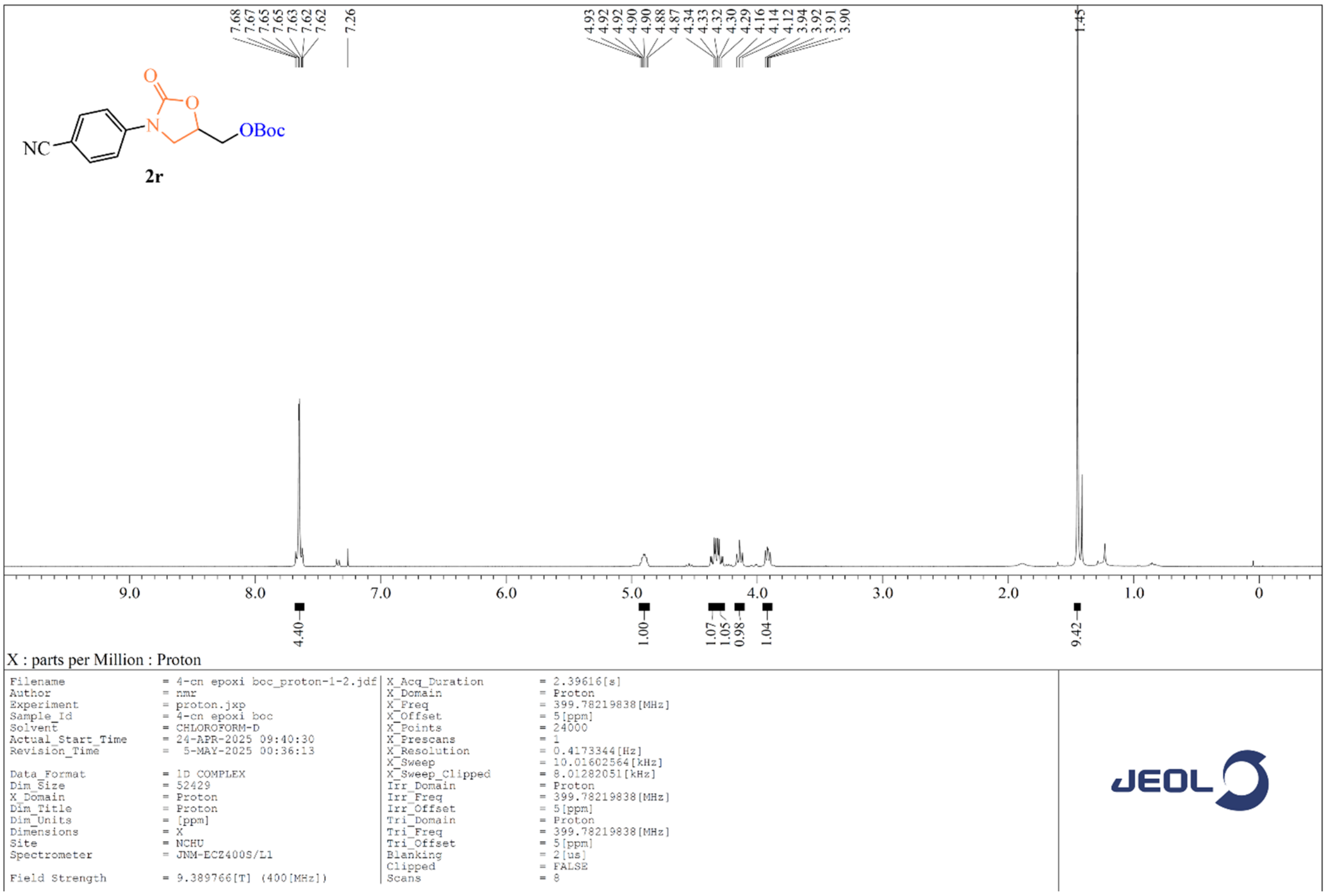


$^1$H NMR spectrum of compound **2r** (400 MHz, $CDCl_3$)

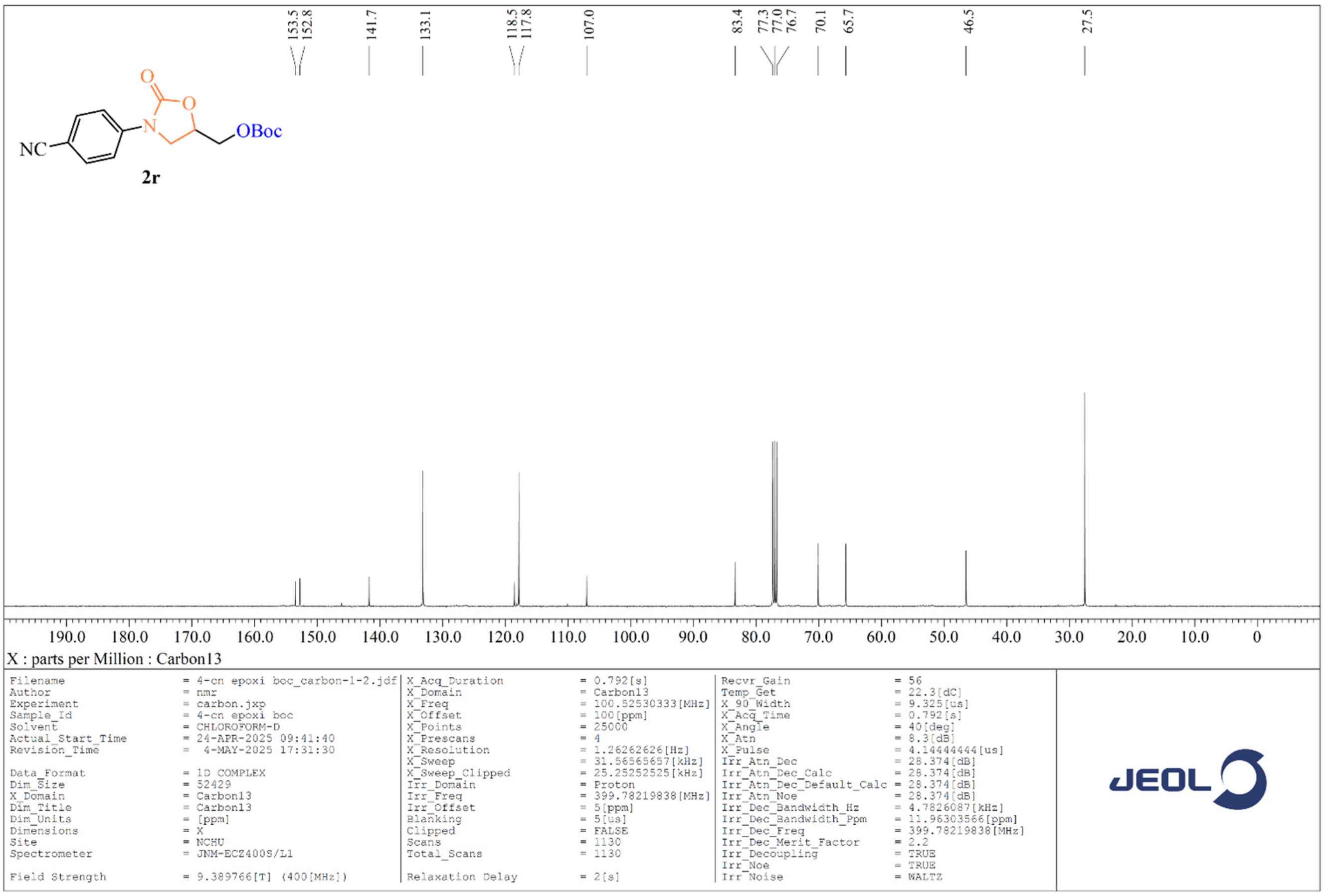


$^{13}$C NMR spectrum of compound **2r** (101 MHz, $CDCl_3$)

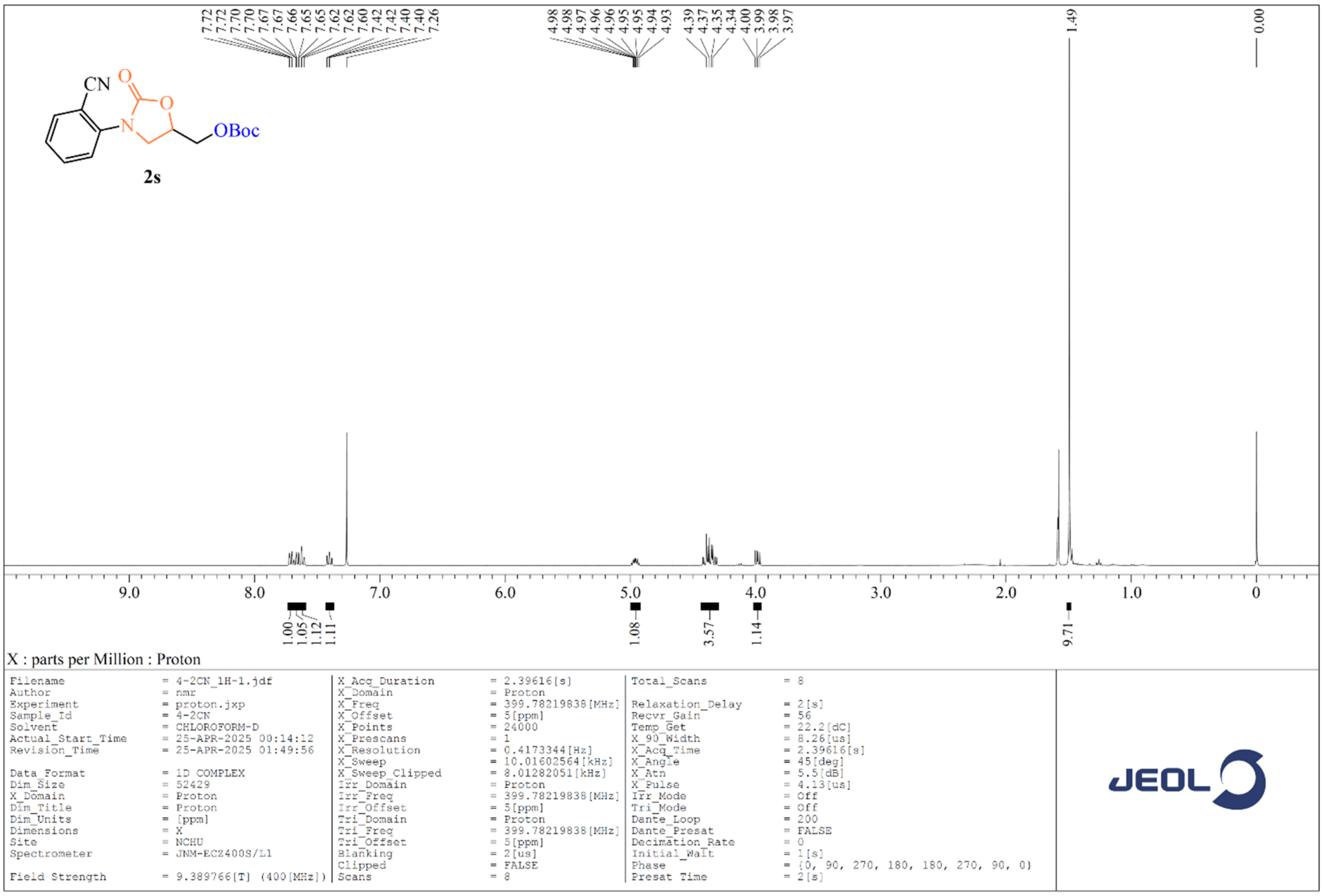


$^{1}$H NMR spectrum of compound **2s** (400 MHz, $CDCl_3$)

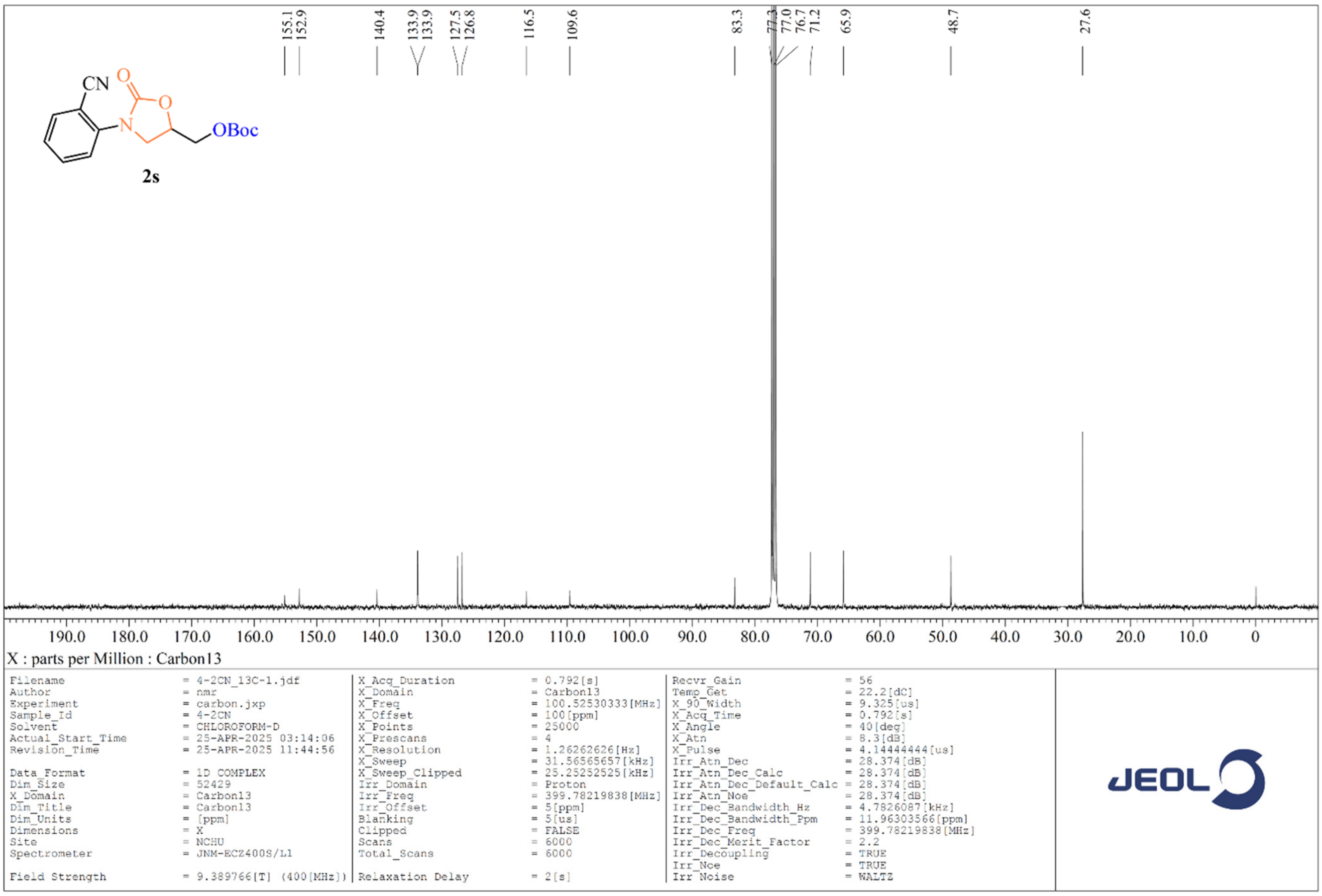


$^{13}$C NMR spectrum of compound **2s** (101 MHz, $CDCl_3$)

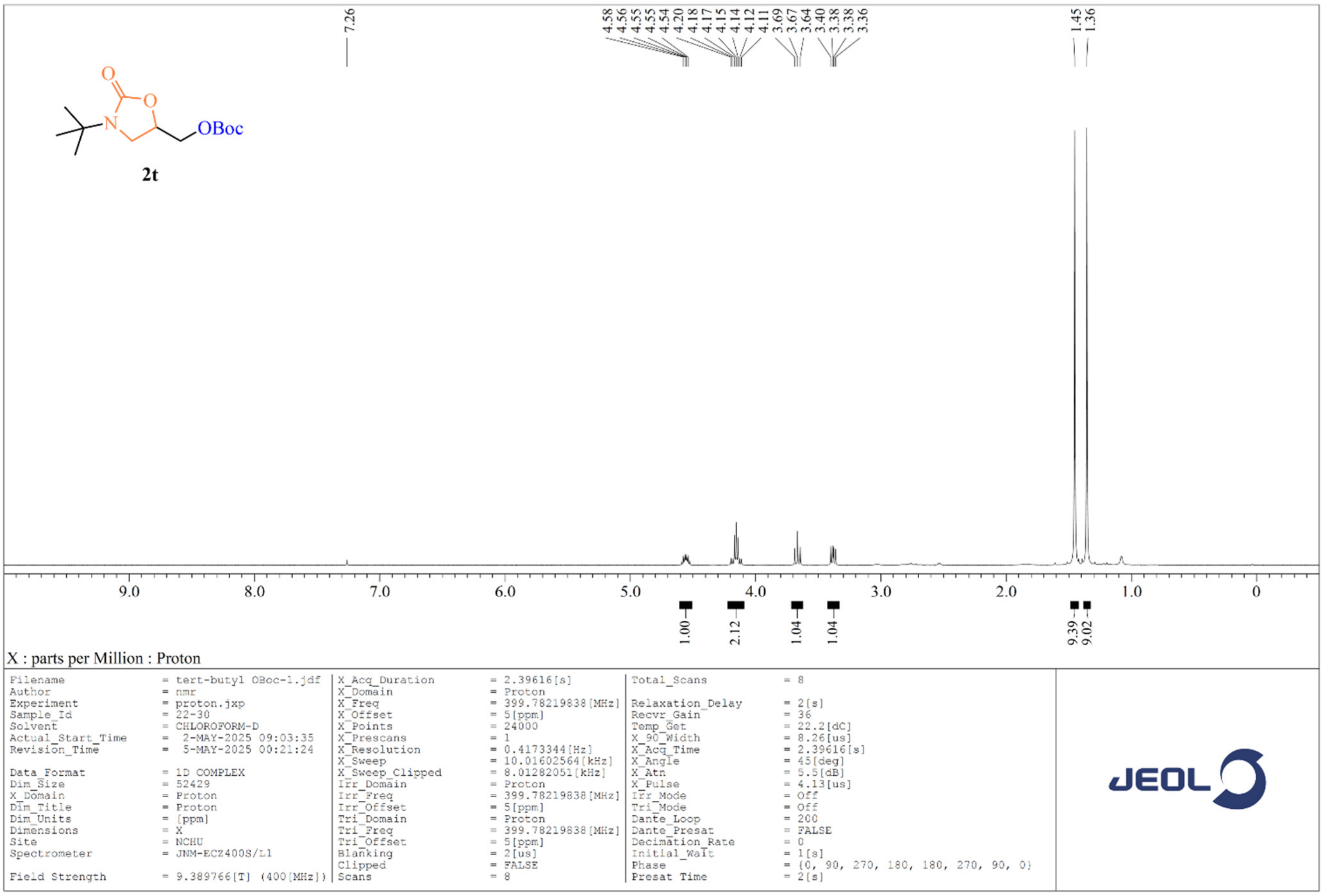


$^{1}H$ NMR spectrum of compound **2t** (400 MHz, $CDCl_3$)

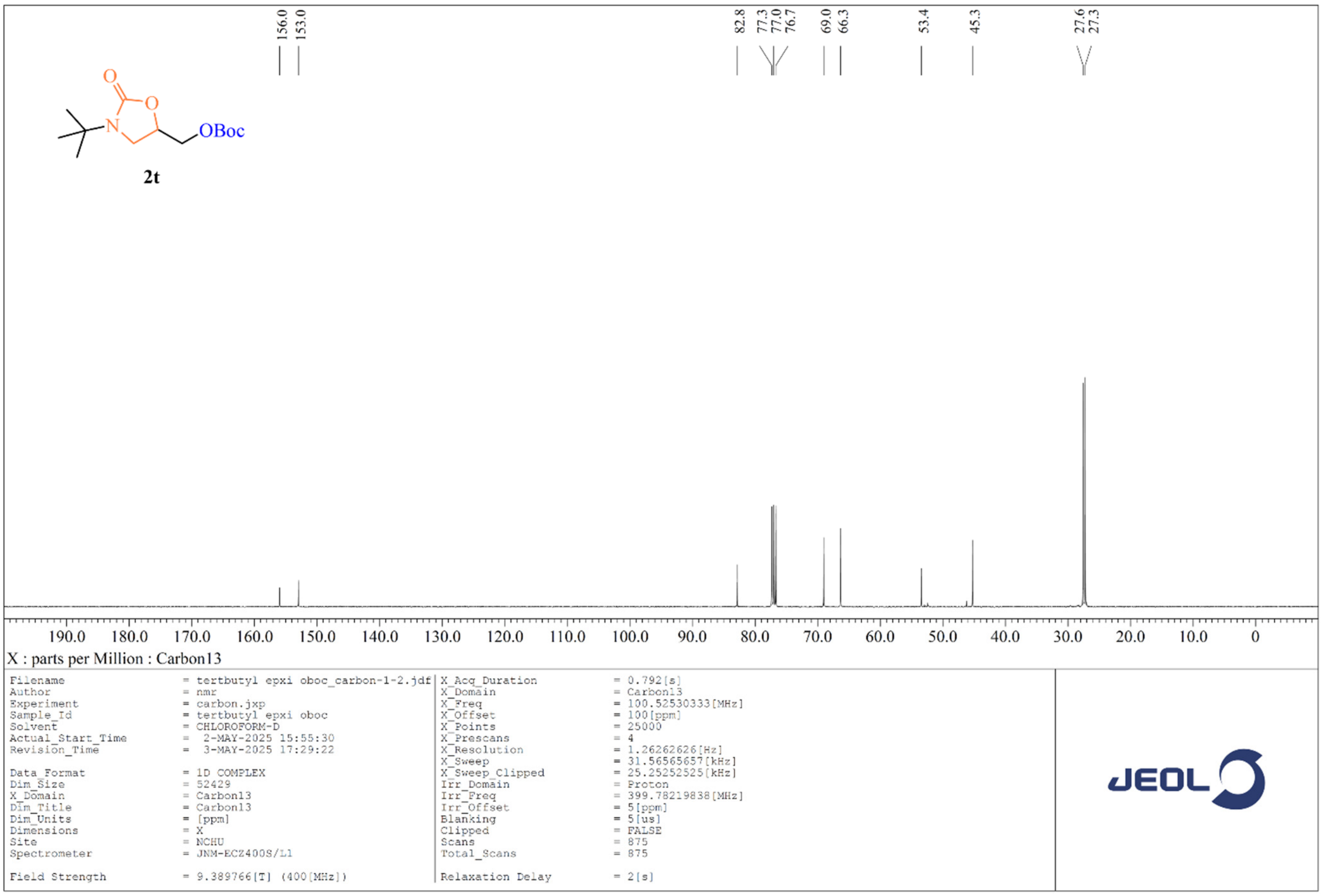


$^{13}C$ NMR spectrum of compound **2t** (101 MHz, $CDCl_3$)

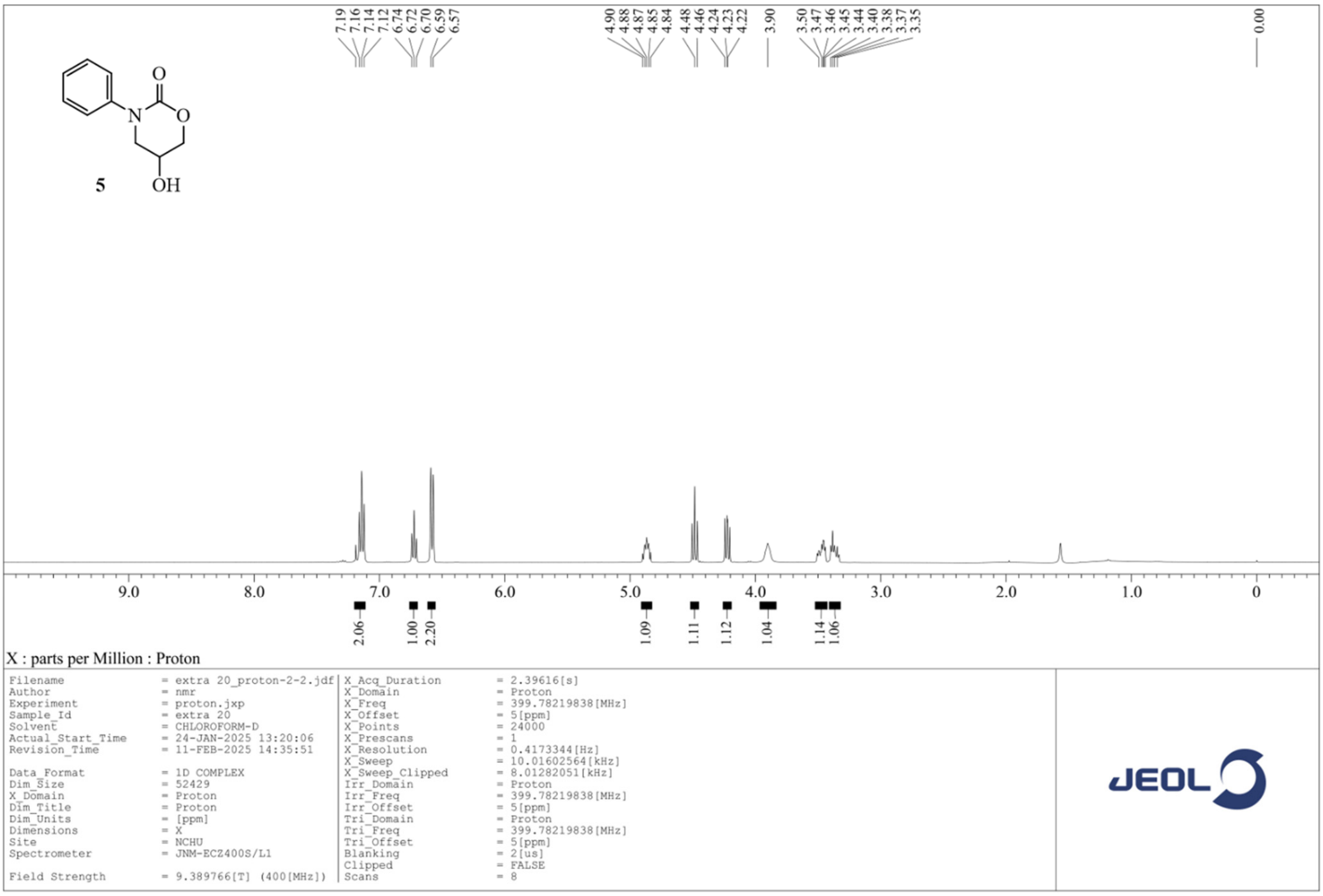


$^{1}H$ NMR spectrum of compound **5** (400 MHz, $CDCl_3$)

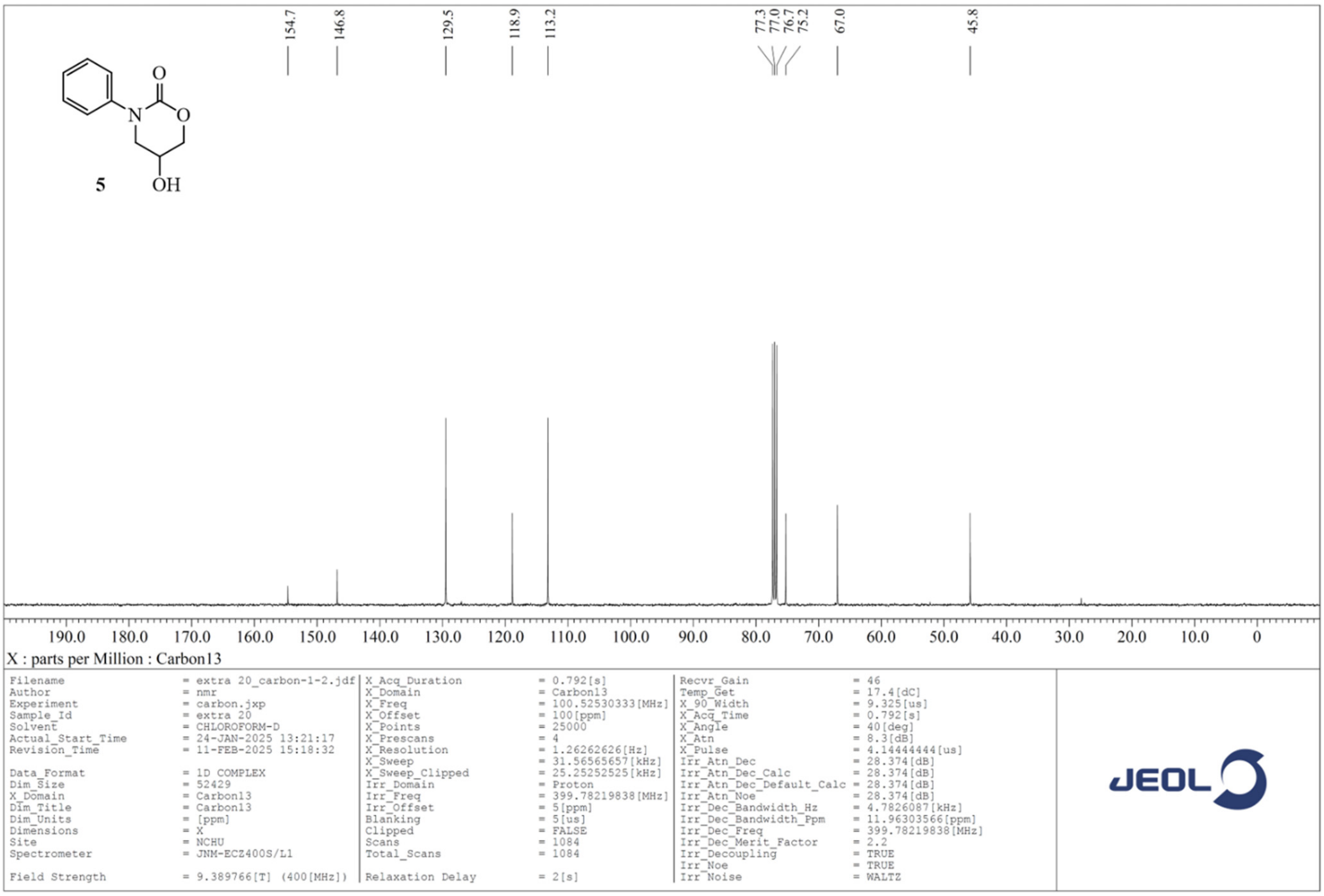


$^{13}C$ NMR spectrum of compound **5** (101 MHz, $CDCl_3$)

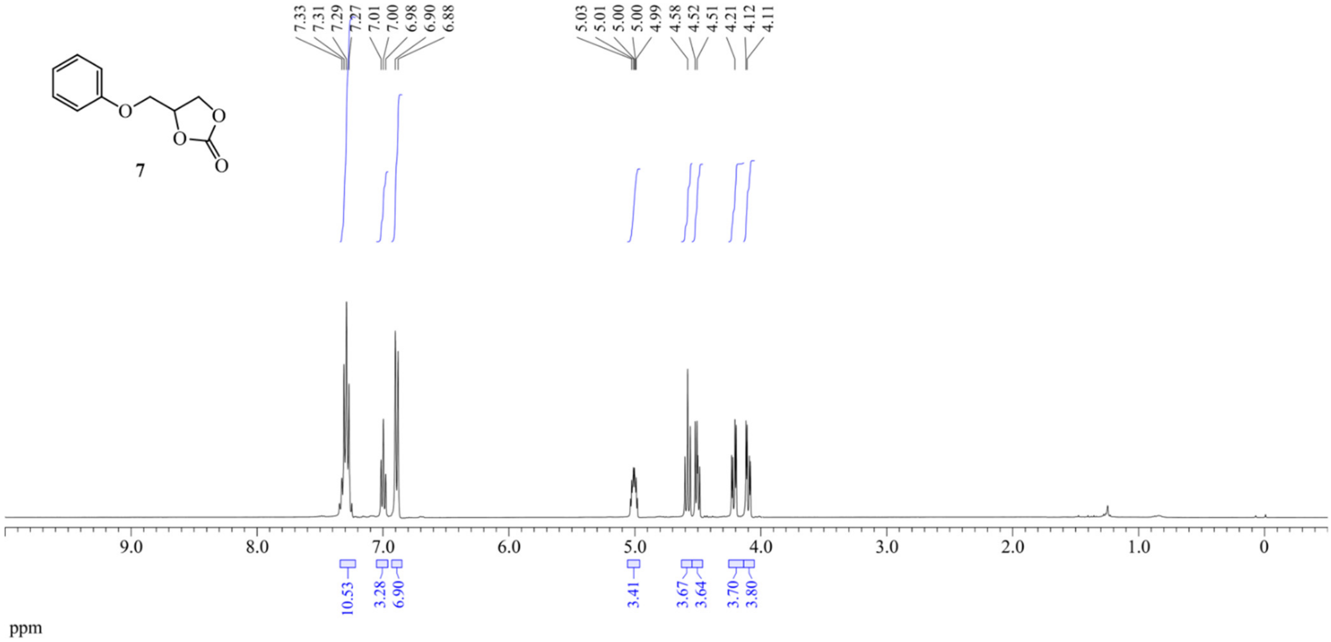


$^{1}$H NMR spectrum of compound **7** (400 MHz, $CDCl_3$)

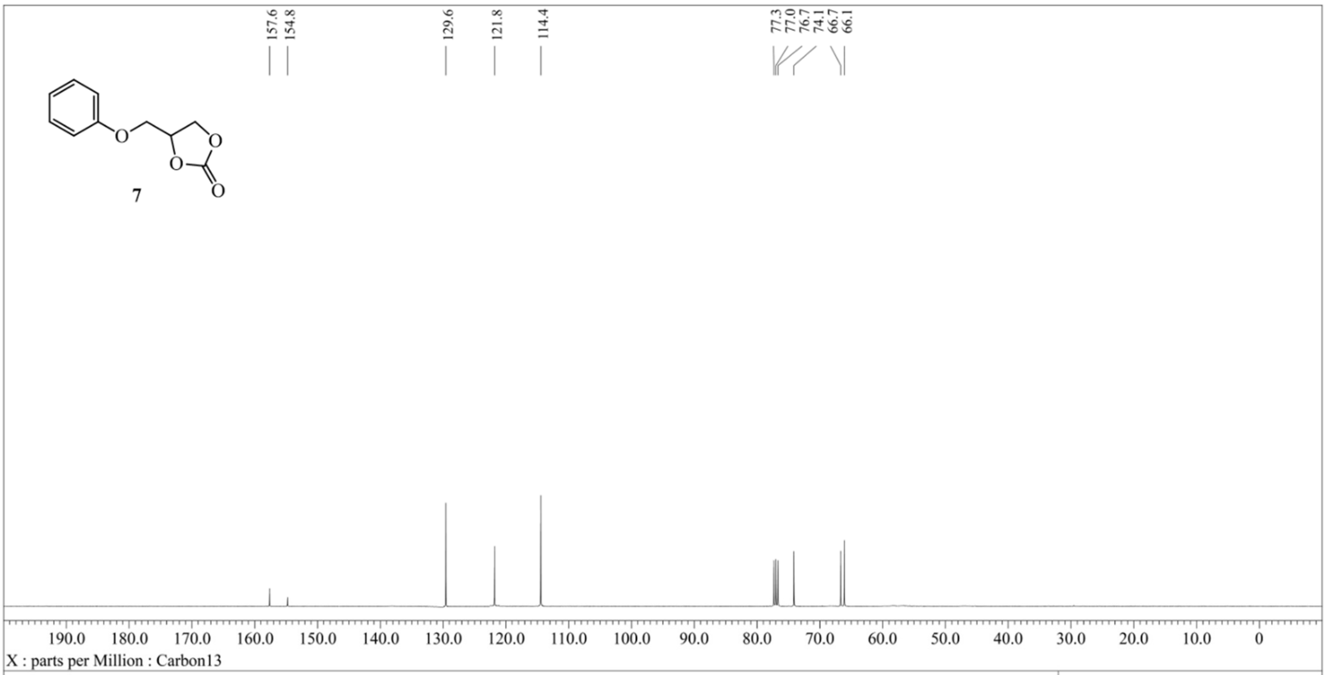


$^{13}$C NMR spectrum of compound **7** (101 MHz, $CDCl_3$)

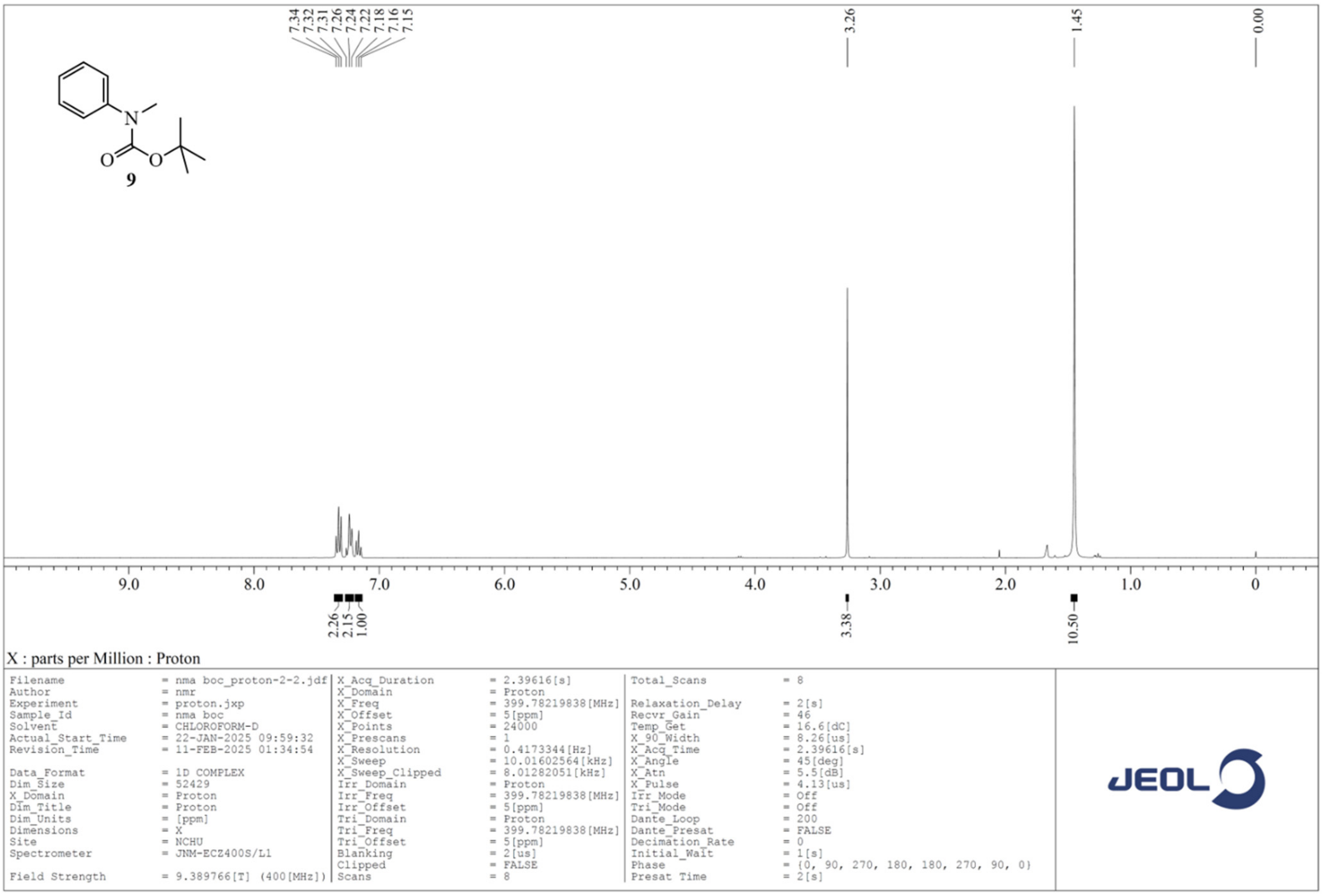


$^1$H NMR spectrum of compound **9** (400 MHz, $CDCl_3$)

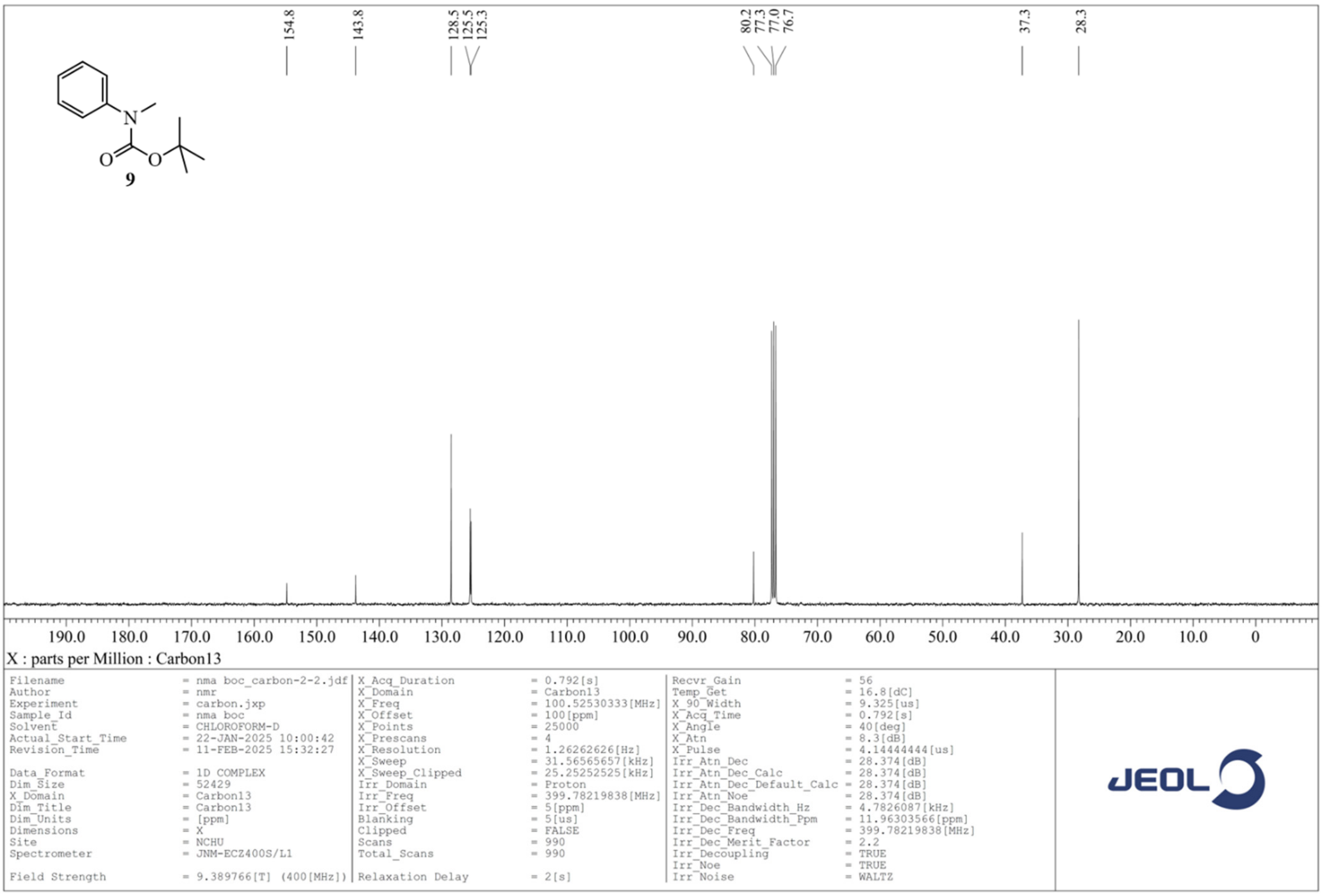


$^{13}$C NMR spectrum of compound **9** (101 MHz, $CDCl_3$)

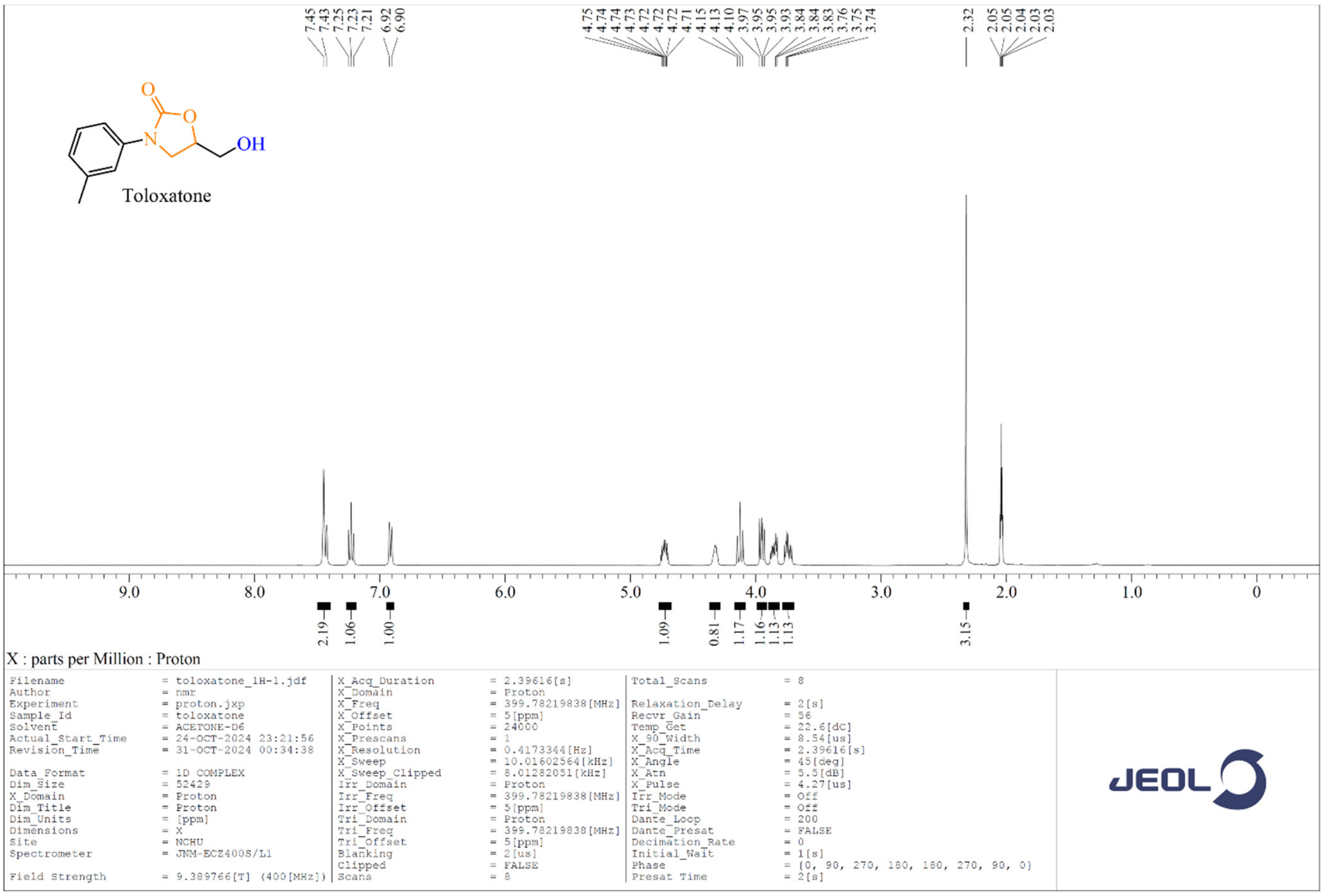


$^{1}H$ NMR spectrum of Toloxatone (400 MHz, acetone-$d_6$)

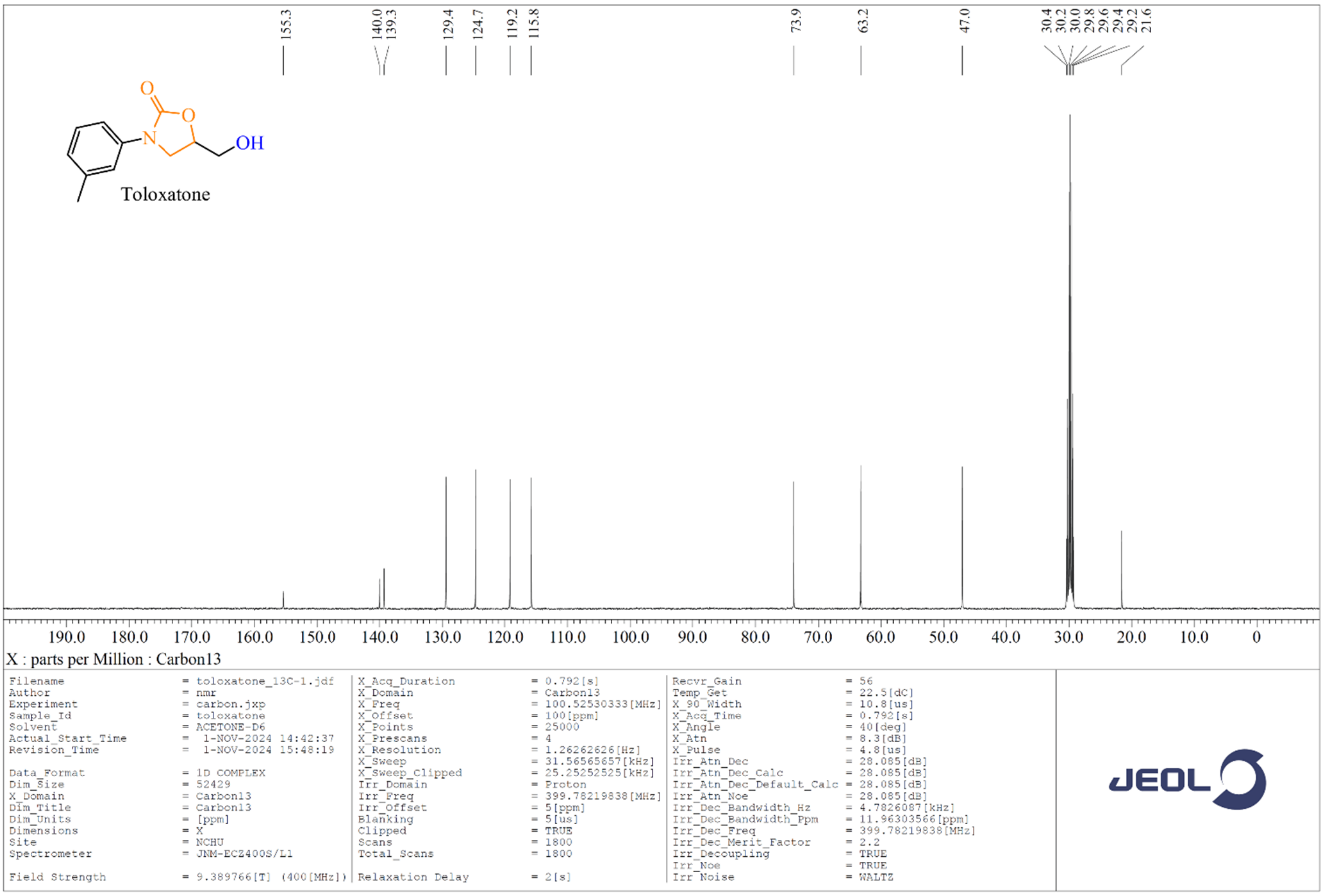


$^{13}C$ NMR spectrum of Toloxatone (101 MHz, acetone-$d_6$)

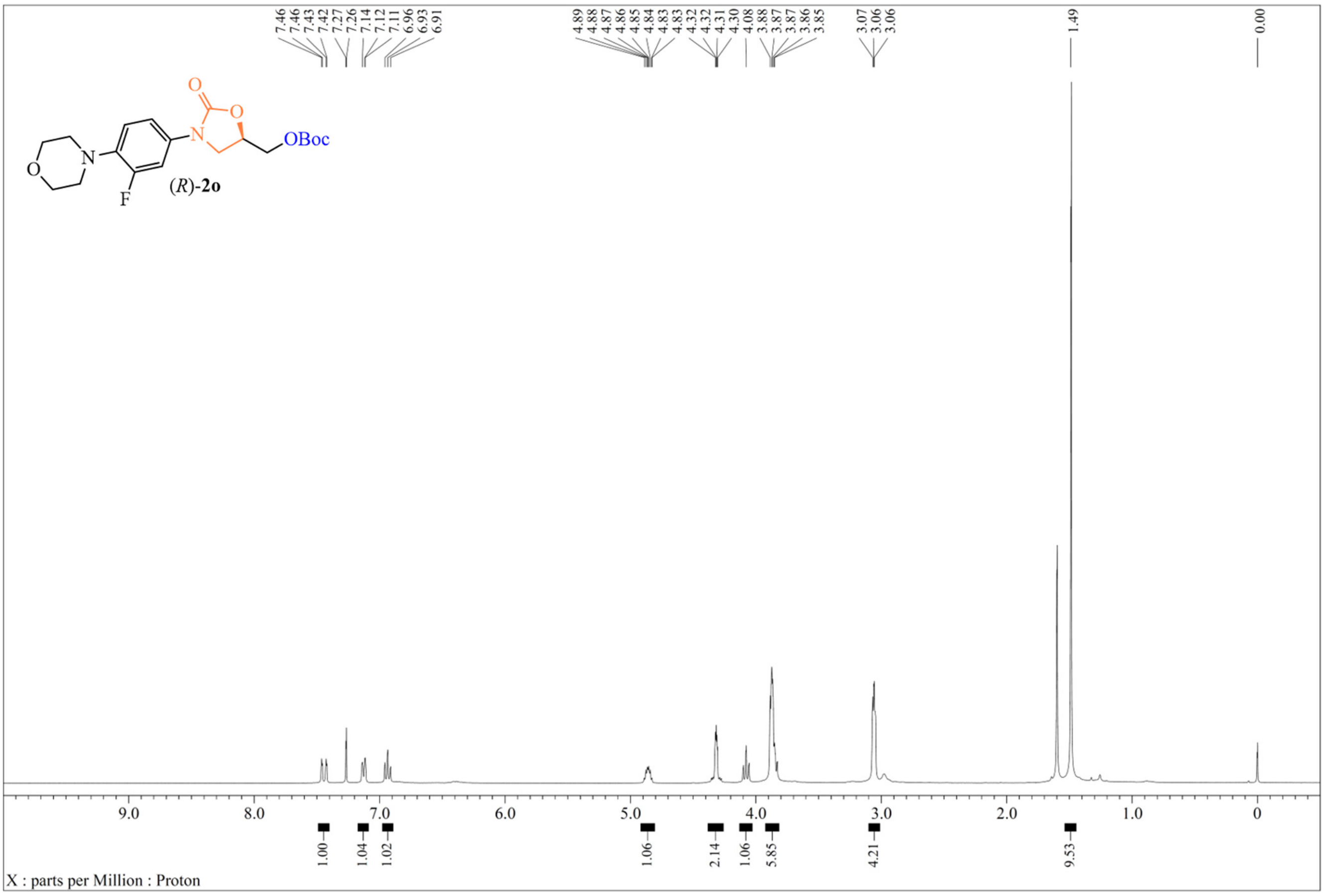

$^{1}$H NMR spectrum of compound (*R*)-**2o** (400 MHz, $CDCl_3$)

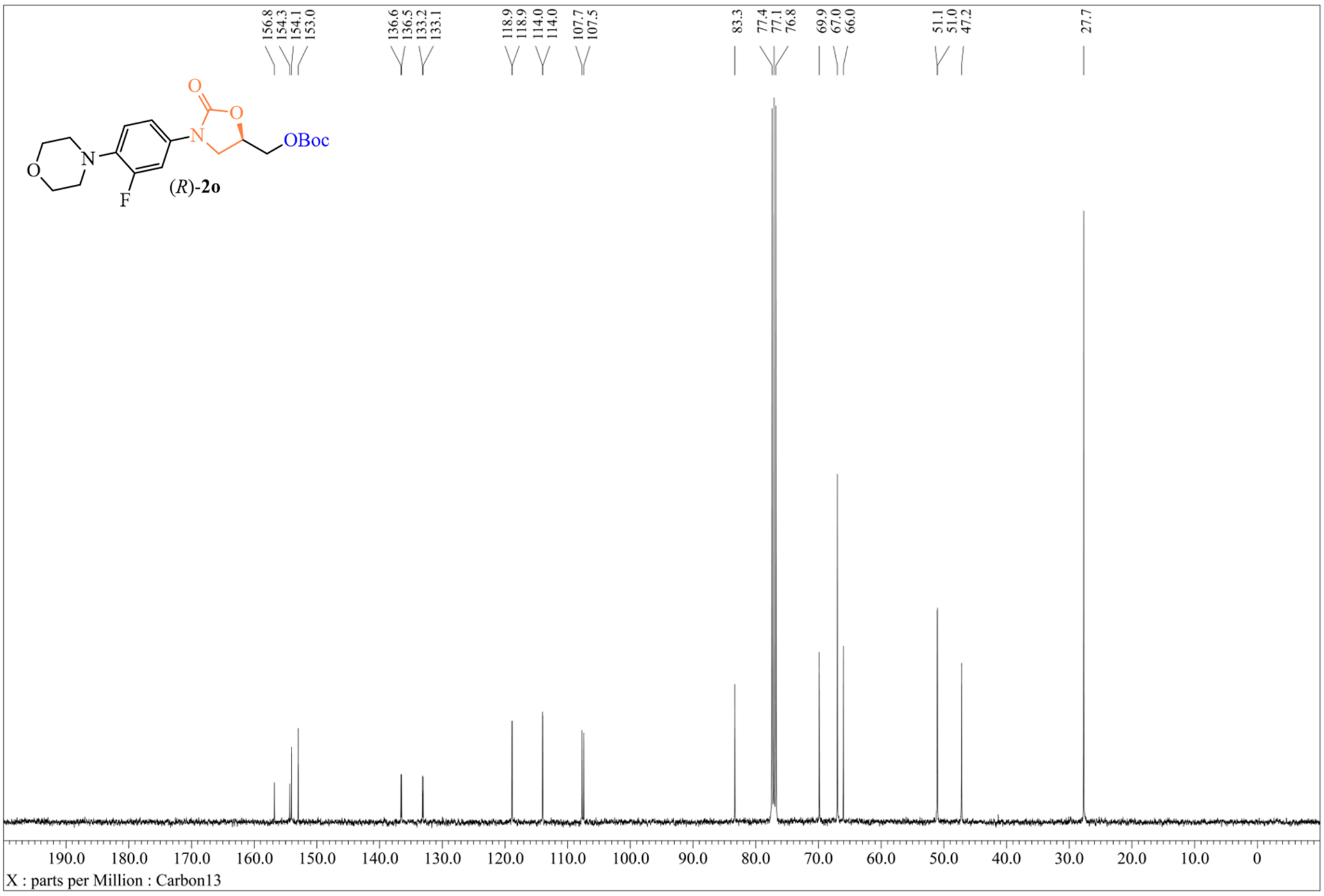

$^{13}$C NMR spectrum of compound (*R*)-**2o** (101 MHz, $CDCl_3$)

## References and Notes:


S1. Zanda, N.; Zhou, L.; Alza, E.; Kleij, A. W.; Pericàs, M. À. Continuous organocatalytic flow synthesis of 2-substituted oxazolidinones using carbon dioxide, *Green Chem.* **2022**, *24*, 4628–4633.

S2. Hong, H.; Johansson, M.; Li, K. Synthesizing method, partial intermediate products and final products of chiral beta-alkamine derivative, CN101717341B, **2013**.

S3. Toda, Y.; Shishido, M.; Aoki, T.; Sukegawa, K.; Suga, H. Switchable synthesis of cyclic carbamates by carbon dioxide fixation at atmospheric pressure, *Chem. Commun.* **2021**, *57*, 6672–6675.

S4. Toda, Y.; Iwasaki, M.; Suga, H. Base-mediated synthesis of cyclic dithiocarbamates from 1-amino-3-chloropropan-2-ol derivatives and carbon disulfide, *Org. Biomol. Chem.* **2023**, *21*, 6293–6297.

S5. Pace, V.; Cabrera, Á. C.; Fernández, M.; Sinisterra, J. V.; Alcántara, A. R. First General Route to Substituted α-Arylamino-α′-chloropropan-2-ones by Oxidation of *N*-Protected Aminohalohydrins: The Importance of Disrupting Hydrogen Bond Networks, *Synthesis*, **2010**, *20*, 3545–3555.

S6. Maiti, G.; Kundu, P.; Mallik, A. K. Mild and Efficient Synthesis of *β*-Amino Alcohols by Antimony Trichloride Catalyzed Opening of Epoxides, *J. Indian Chem. Soc.* **2008**, *85*, 412–416.

S7. Johncock, P.; Porecha, L.; Tudgey, G. F. The relative reactivity of primary and secondary amine hydrogen atoms of aromatic amines with epichlorohydrin and *N*- and *O*-glycidyl compounds, *J. Polym. Sci.* **1985**, *23*, 291–301.

S8. Lee, Y.; Choi, J.; Kim, H. Stereocontrolled, Divergent, Al(III)-Catalyzed Coupling of Chiral *N*-Aryl Epoxy Amines and $CO_2$, *Org. Lett.* **2018**, *20*, 5036−5039.

S9. Hu, Y.; Li, X.; Wan, B. NIS-mediated ring-closure/opening cascade reactions of allylamides: an expedient route to oxazolines, *Tetrahedron*, **2015**, *71*, 6935–6943.

S10. Rintjema, J.; Epping, R.; Fiorani, G.; Martín, E.; Escudero-Adán, E. C.; Kleij, A. W. Substrate-Controlled Product Divergence: Conversion of $CO_2$ into Heterocyclic Products, *Angew. Chem. Int. Ed.* **2016**, *55*, 3972–3976.

S11. Singh, R.; Sharma, A.; Khanna, M. S.; Prasad, M. A PROCESS FOR THE PREPARATION OF TEDIZOLID PHOSPHATE, WO2016088103A1, **2016**.

S12. Yu, T.; Weiss, R. G. Syntheses of cyclic carbonates with amidinium halide catalysts in reusable, reversible, room-temperature ionic liquids or acetonitrile, *Green Chem.* **2012**, *14*, 209–216.

S13. Basel, Y.; Hassner, A. Di-*tert*-butyl Dicarbonate and 4-(Dimethylamino)pyridine Revisited. Their Reactions with Amines and Alcohols, *J. Org. Chem.* **2000**, *65*, 6368–6380.

S14. Bhunia, S.; De, S.; Ma, D. Room Temperature Cu-Catalyzed *N*-Arylation of Oxazolidinones and Amides with (Hetero)Aryl Iodides, *Org. Lett.* **2022**, *24*, 1253−1257.
